# Can Side-Chain Conformation and Glycosylation Selectivity of Hexopyranosyl Donors Be Controlled with a Dummy Ligand?

**DOI:** 10.1021/acs.joc.2c02889

**Published:** 2023-03-06

**Authors:** Kapil Upadhyaya, Nicolas Osorio-Morales, David Crich

**Affiliations:** † Department of Pharmaceutical and Biomedical Sciences, University of Georgia, 250 West Green Street, Athens, Georgia 30602, United States; ‡ Department of Chemistry, University of Georgia, 302 East Campus Road, Athens, Georgia 30602, United States; § Complex Carbohydrate Research Center, University of Georgia, 315 Riverbend Road, Athens, Georgia 30602, United States

## Abstract

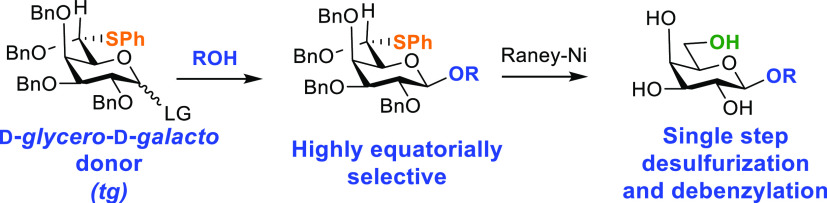

The use of a phenylthio group (SPh) as a dummy ligand at the 6-position to control the side-chain conformation of a series of hexopyranosyl donors is described. The SPh group limits side-chain conformation in a configuration-specific manner, which parallels that seen in the heptopyranosides, and so influences glycosylation selectivity. With both d- and l-glycero-d-galacto-configured donors, the equatorial products are highly favored as they are with an l-glycero-d-gluco donor. For the d-glycero-d-gluco donor, on the other hand, modest axial selectivity is observed. Selectivity patterns are discussed in terms of the side-chain conformation of the donors in combination with the electron-withdrawing effect of the thioacetal group. After glycosylation, removal of the thiophenyl moiety and hydrogenolytic deprotection is achieved in a single step with Raney nickel.

## Introduction

**Figure 1:**
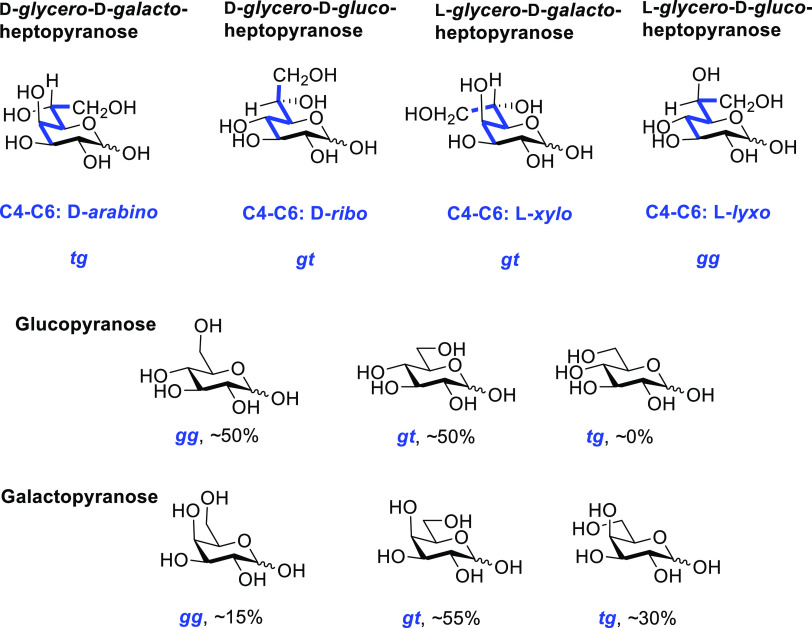
Influence of relative configuration on the side-chain conformation of heptopyranosides and hexopyranosides.

In the hepto- and higher carbon pyranosides (and hexo- and higher carbon furanosides), the conformation of the exocyclic C–C bond, hereinafter the side chain, is controlled by the relative configuration of the C4–C6 stereotriad (and of the C3–C5 triad in the furanosides).^1−3^ Compounds with the arabino configuration in this location predominantly take up the *tg* conformation,^4−7^ while those with the ribo and xylo configurations adopt the *gt* conformation; finally, compounds with the local lyxo configuration mainly populate the *gg* conformation (Figure 1). This pattern, which arises from a combination of steric, gauche, and dipolar interactions,^1,8,9^ differs substantially from that found in the simple hexopyranosides (and pentofuranosides),^5−7,10^ where the side-chain conformation is mainly dictated by the configuration at C4, with the *gluco* series adopting a near-equimolar mixture of the *gg* and *gt* conformations and the *galacto* series a 15:55:30 *tg*:*gt*:*gg* mixture (Figure 1).^5−7^

In typical glycosylation reactions in which an electrophilic glycosyl donor undergoes nucleophilic substitution of a leaving group by an acceptor alcohol,^11−13^ systems in which the donor side chain predominantly occupies the *tg* conformation are less reactive and more equatorially selective than those with a predominant *gg* conformation of the side chain, whereas those whose side chain mainly populates the *gt* conformation have intermediate reactivity and selectivity. This is because (i) the *tg* conformation maximizes the electron-withdrawing effect of the C6–O6 bond, thereby destabilizing nascent positive charge at the anomeric center, so favoring bimolecular mechanisms, and (ii) the *gg* conformation is best placed to electrostatically stabilize the developing positive charge at the anomeric center, while (iii) the *gt* conformation has intermediate behavior.^14,15^ Glycosylation reactivity and selectivity in the hepto- and higher carbon pyranosyl donors is therefore an inherent function of the relative configuration of C4–C6 stereotriad and of the so-imposed side-chain conformations. In the hexopyranosides, donor reactivity can be correspondingly influenced by locking the side-chain conformation with the aid of 4,6-*O*-benzylidene acetals or related cyclic protecting groups. However, as only one conformation is available in this manner for the gluco (*tg*) and galacto (*gg*) series, such options are limited.^16^

We now address the question of the control of side-chain conformation and glycosylation reactivity and selectivity in hexopyranosyl donors without the use of rigid bicyclic protecting groups at the 4,6-position. To this end, we envisaged that side-chain conformation in the hexopyranosides could be achieved by installation of a temporary or dummy ligand at the 6-position to mimic the C6–C7 bond in the hepto and higher carbon sugars. To be effective, such a ligand would need to be easily installed and removed, of comparable electronegativity and steric bulk to a methyl group, and stable under typical glycosylation conditions, causing us to settle on the thioether moiety.

## Results and Discussion

**Scheme 1:**
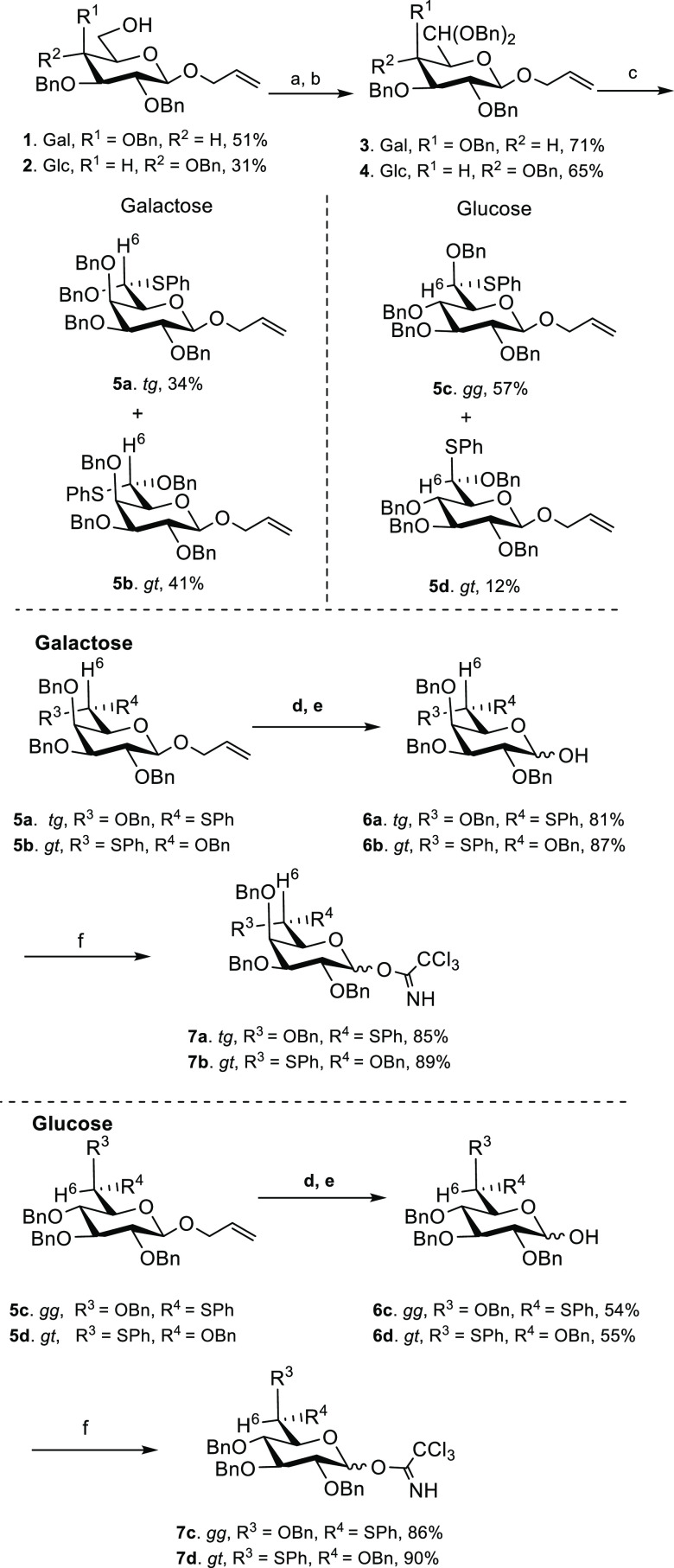
Synthesis of Trichloroacetimidate Donors [a] 1.5 equiv of Dess–Martin periodinane (DMP), CH_2_Cl_2_, r.t. [b] 2 equiv of CH(OBn)_3_, 0.2 equiv of *p*-TSA, Na_2_SO_4_, CH_3_CN, r.t. [c] 1.5 equiv of PhSH, 1.5 equiv of BF_3_·OEt_2_, toluene, −78 °C to −20 °C. [d] 10 mol % RuCl_2_(PPh_3_)_3_, 1 equiv of DBU, EtOH, reflux. [e] cat. OsO_4_, 3 equiv of NMO, dioxane:H_2_O 4:1, r.t. [f] 5 equiv of Cl_3_CCN, cat. DBU, CH_2_Cl_2_, 0 °C.

The preparation of the thioether derivatives **5a**–**d** began with the synthesis of alcohols **1** and **2**,^17−19^ which were oxidized to the corresponding aldehydes using the Dess–Martin periodinane^20,21^ followed by the formation of dibenzylacetals **3** and **4** with tribenzyl orthoformate and a catalytic amount of *p*-TSA in 71 and 65% yields, respectively.^22^ These acetals were treated with 1.5 equiv of BF_3_·OEt_2_ and 1.5 equiv of thiophenol at −78 to −20 °C to obtain the *S*-phenyl-*O*-benzyl monothioacetals **5a** and **5b** in the *galacto* series and **5c** and **5d** in the *gluco* series (Scheme 1). Provided the temperature is carefully controlled to avoid over-reaction and dithioacetal formation, the diastereomeric ratios in the formation of monothioacetals **5a** and **5b** and **5c** and **5d** were reproducible over multiple runs. At the present time, however, we do not have a rationale for the observed selectivities.

**Figure 2:**
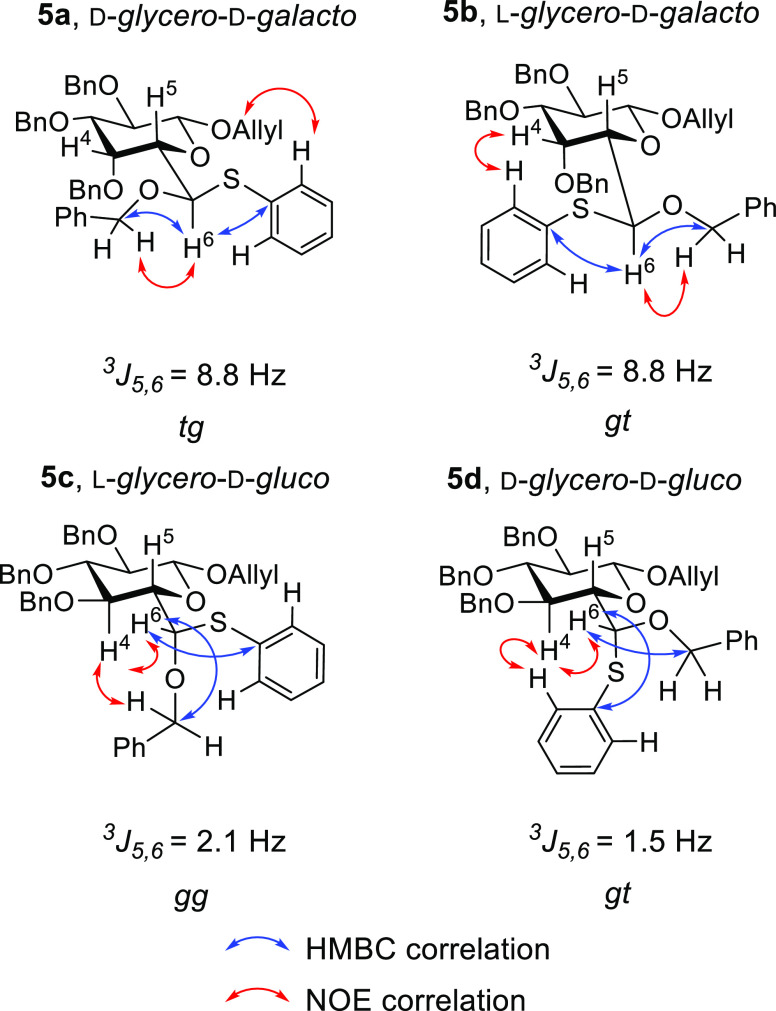
Key NOESY correlation. Red arrows: NOE correlation. Blue arrows: HMBC correlation. Nomenclature based on heptopyranoside series.

Both pairs of isomers were purified by silica gel chromatography and their structures were confirmed by NMR spectroscopy. For each isomer, the side-chain configurations and conformations were determined with a combination of coupling constant analysis and NOE measurements. For both **5a** and **5b**, a ^3^*J*_5,6_ coupling constant of 8.8 Hz requires an antiperiplanar relationship between H^5^ and H^6^ (Figure 2). The heteronuclear multiple bond coherence (HMBC) correlations of H^6^ located the corresponding methylene carbon signals for O^6^-CH_2_-Ph and the *ipso* carbons for the SPh groups in both compounds. An NOE correlation between the *ortho-*protons of the thiophenyl moiety and the methylene protons in O^1^-CH_2_CH=CH_2_ confirmed the d-*glycero*-d-*galacto* configuration^23^ and *tg* conformation of **5a**, for which additionally no measurable NOE correlation was found between H^4^ and the *ortho*-protons of the SPh group. For **5b**, an NOE correlation revealed the spatial proximity of H^4^ and the *ortho*-protons of the SPh moiety indicating a predominant *gt* conformation and the l-*glycero*-d-*galacto* configuration.

For both isomers in the *gluco* series, an NOE correlation linking H^4^ and H^6^ and the ^3^*J*_5,6_ coupling constants together require H^6^ to be predominantly antiperiplanar to O^5^. HBMC correlations with H^6^ identified the methylene carbon of the O^6^CH_2_Ph moiety whose attached protons showed an NOE correlation with H^4^, indicating the l-*glycero*-d-*gluco* configuration and a predominant *gg* conformation for **5c** (Figure 2). Finally, for **5d**, the *ipso* carbon of the SPh group, and so its respective *ortho*-protons, was identified by HMBC correlation to H^6^.

The NOE correlation between H^4^ and these *ortho*-SPh protons finally confirms the d-*glycero*-d-*gluco* configuration and *gt* conformation of **5d**. The predominant conformations assigned to **5a–d** are fully consistent with those found for the corresponding heptopyranosides (Figure 1) and support the central hypothesis of the use of a phenylthio group as a side-chain conformation-controlling dummy ligand in the hexopyranosides.

The allyl groups were removed from each of **5a**–**d** by isomerization with 10 mol % of RuCl_2_(PPh_3_)_3_ followed by treatment with catalytic OsO_4_ and *N*-methylmorpholine *N*-oxide to give the corresponding hemiacetals **6a**–**d** as α:β mixtures.^24,25^ Finally, the trichloroacetimidate donors **7a**–**d** were obtained by treatment with 5 equiv of Cl_3_CCN and catalytic amount of DBU at 0 °C in the form of α:β mixtures.^26−29^

Glycosylation reactions were carried out by stirring 0.15 M CH_2_Cl_2_ solutions of donors **7a**–**d** (1.0 equiv) with the acceptor (1.2 equiv) and 4 Å AWMS for 1 h, followed by cooling to −78 °C and stirring for another 0.5 h. TMSOTf (0.2 equiv) then was added to the mixture as a 10% solution in CH_2_Cl_2_.^11,30−32^ All glycosylations were maintained at −78 °C and quenched with 0.2 mL of triethylamine at that temperature before the mixtures were warmed to room temperature, filtered through celite, diluted with CH_2_Cl_2_, and washed with saturated aqueous NaHCO_3_. The resulting glycosides were then isolated by silica gel chromatography, leading to the results presented in Tables 1 and 2.

**Table 1:**
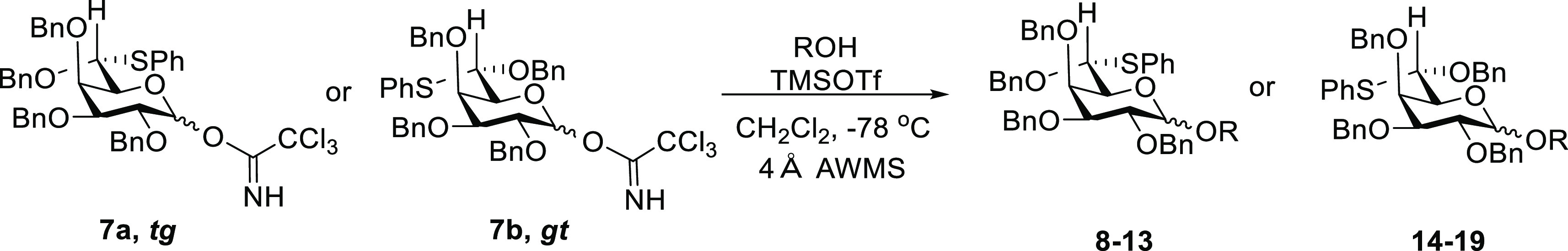

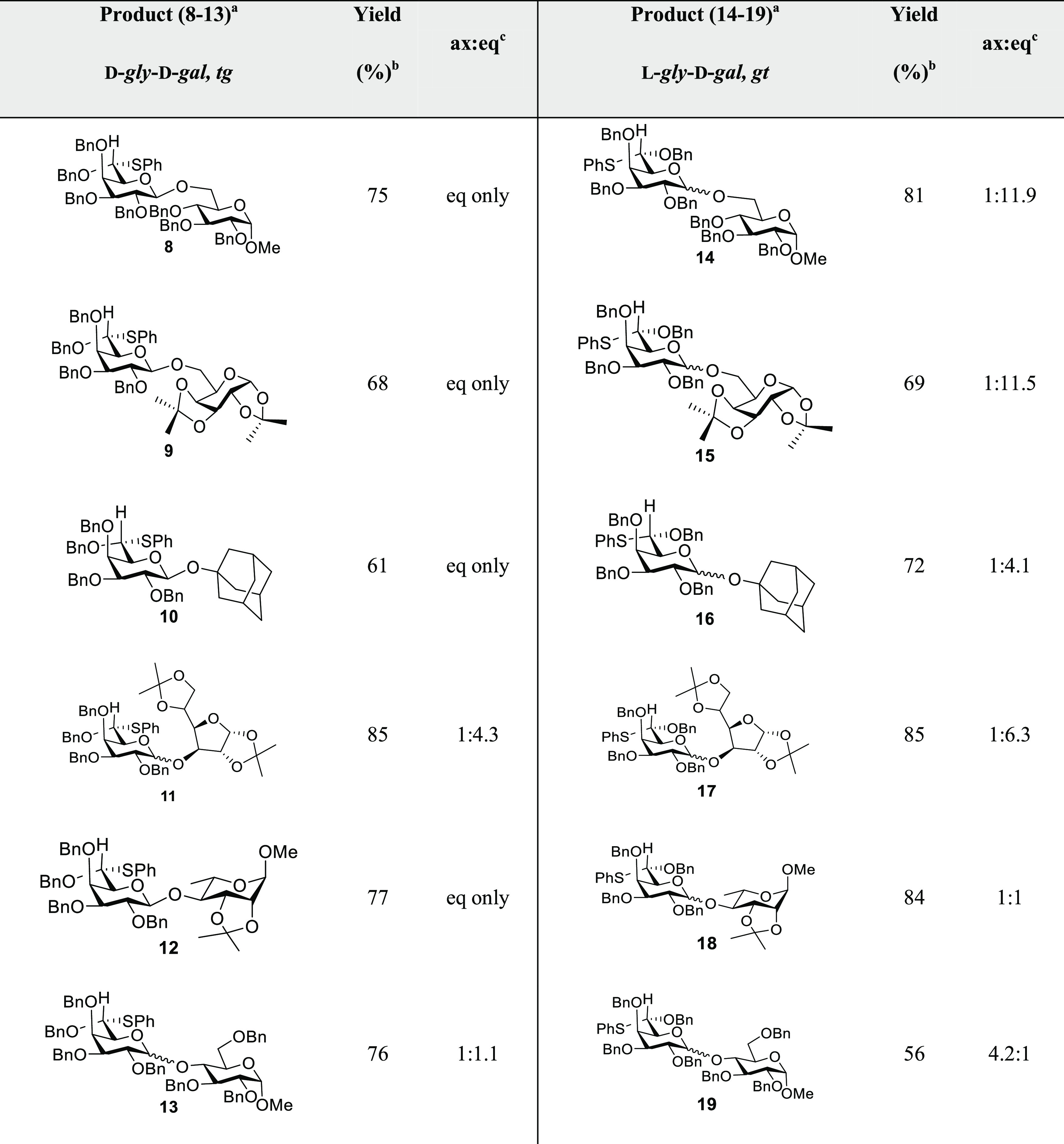
Glycosylation Reactions with d-*glycero*-d-*galacto* Donor **7a** and l-*glycero*-d-*galacto* Donor **7b**

a All reactions were carried out at −78 °C with activation by 0.2 equiv of TMSOTf. The donor-to-acceptor ratio is 1.0:1.2 with a 0.15 M concentration of donor.

b Isolated yield.

c Anomeric ratios were determined by integration of the anomeric signals in the ^1^H NMR spectra of the crude reaction mixtures.

**Table 2:**
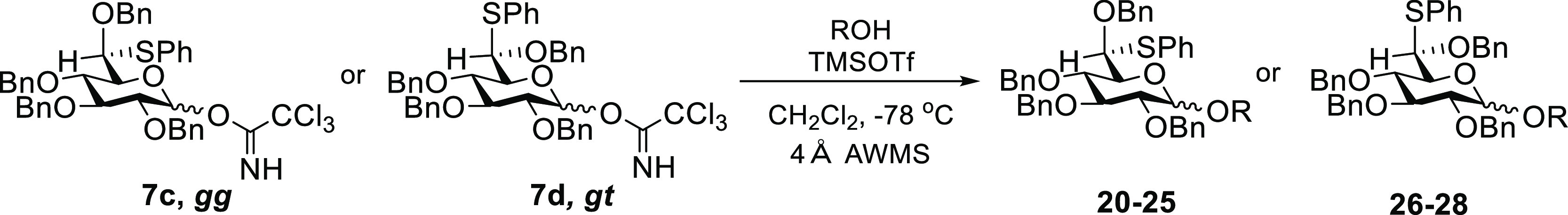

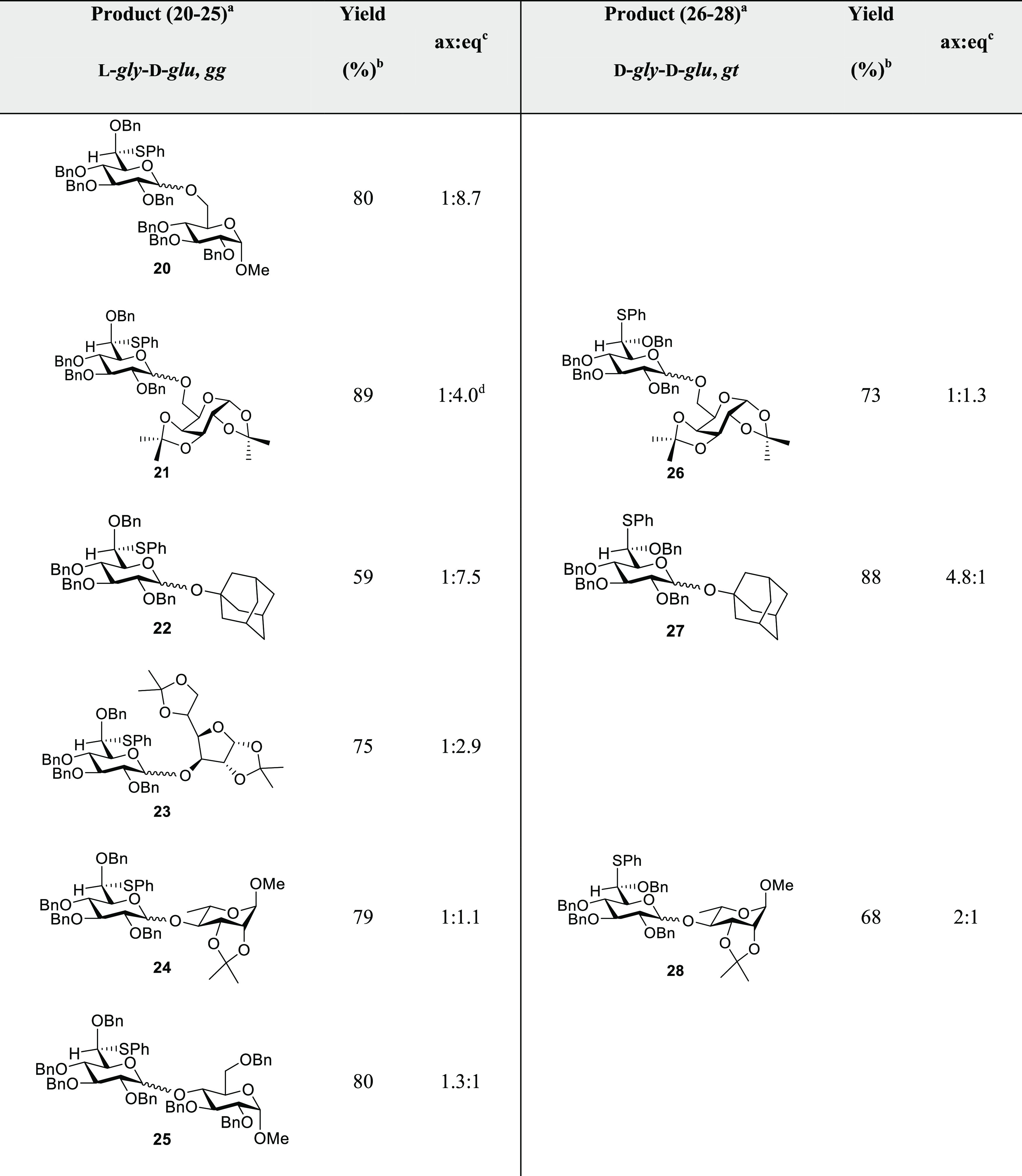
Glycosylation Reactions with l-*glycero*-d-*gluco* Donor **7c** and d-*glycero*-d-*gluco* Donor **7d**

a All reactions were carried out at −78 °C with activation by 0.2 equiv of TMSOTf. The donor-to-acceptor ratio is 1.0:1.2 with a 0.15 M concentration of donor.

b Isolated yield.

c Anomeric ratios were determined by integration of the anomeric signals in the ^1^H NMR spectra of the crude reaction mixtures.

d When this reaction was conducted on a scale of ∼1 g, **21** was obtained in a 92% isolated yield with an α:β ratio of 1:3.

Both the d-*glycero*-d-*galacto* and l-*glycero-*d-*galacto* donors (Table 1) showed good yields and high equatorial selectivity with primary alcohol acceptors. The 1-adamantyl glycosides were also formed with high equatorial selectivity in the case of **7a** with its *tg* conformation and modest equatorial selectivity for **7b** with the *gt* conformation. For such examples, this new method could be considered as complementary to the use of acetonitrile for the formation of β-galactopyranosides in the absence of neighboring group participation^33^ and also to the Kiso method using a 4,6-*O*-silylene acetal for the formation of α-galactopyranosides.^34^ Secondary alcohol acceptors showed good yields and typically modest equatorial selectivity, with the exception of the coupling of **7b** to the less reactive^35,36^ secondary alcohol methyl 2,3,6-tri-*O*-benzyl-α-d-glucopyranoside.

In the *gluco* series, equatorially selective couplings were observed for the l-*glycero*-d-*gluco* donor **7c** with the *gg* conformation with the exception of coupling to the less reactive methyl 2,3,6-tri-*O*-benzyl-α-d-glucopyranoside that shows modest axial selectivity. For the d-*glycero*-d-*gluco* configured donor **7d**, coupling to two of the three acceptors studied, 1-adamantanol and the relatively reactive alcohol methyl 2,3-*O*-isopropylidene-α-l-rhamnopyranoside showed modest axial selectivity, while the third example, with 1,2;3,4-di-*O*-isopropylidene-α-d-galactopyranose, displayed modest equatorial selectivity (Table 2).

**Figure 3:**
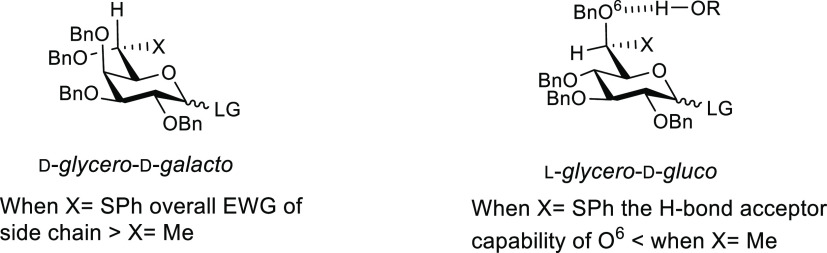
Influence of the side-chain conformation in the selectivity of glycosylations.

For the two galacto donors **7a** and **7b**, the observed selectivities are consistent with those found earlier with the corresponding 7-deoxy d- and l-*glycero*-d-*galactopyranosyl* donors,^1,3,37^ further reinforcing the design hypothesis of employing a phenylthio substituent as a surrogate for a methyl group. It is noteworthy, however, that for donor **7a**, with its *tg* side-chain conformation, the selectivities are better than those seen for the corresponding all-carbon donor. We attribute this phenomenon to a combination of the different coupling methods used and to the additional electron-withdrawing effect of the thioacetal moiety reinforcing that due to the imposition of the *tg* conformation (Figure 3).

In the gluco series, the selectivities observed with donors **7c** and **7d** were again grossly consistent with those observed previously with the corresponding d- and l-*glycero*-d-*gluco*-7-deoxy heptopyranosyl donors,^1^ again in line with the overall hypothesis. However, donor **7c** was somewhat less equatorially selective than its all-carbon analog. As with the galactopyranosides, the β-selective l-*glycero*-d-*gluco* donor **7c** can be considered as complementary to a simple per-*O*-benzyl glucopyranosyl trichloroacetimidate employed in the presence of acetonitrile.^33^

As we had attributed the high equatorial selectivity seen with the l-*glycero*-d-*gluco*-6-deoxy heptopyranosyl donor in the all-carbon series to stereodirecting hydrogen bonding between the *gg-*disposed *O*6 in the donor and the acceptor OH,^37^ the reduced selectivity seen with donor **7c** can again be ascribed to the presence of the electron-withdrawing hemiacetal, which reduces the H-bond acceptor capabilities of *O*6 (Figure 3).

Tables 3 and 4 provide illustrative examples of desulfurization with Raney nickel achieved concomitantly with hydrogenolysis of the benzyl groups.^38−41^ The reactions were carried out in ethanol under 1 atm of hydrogen at room temperature and gave the desired products in good yields.

**Table 3:**
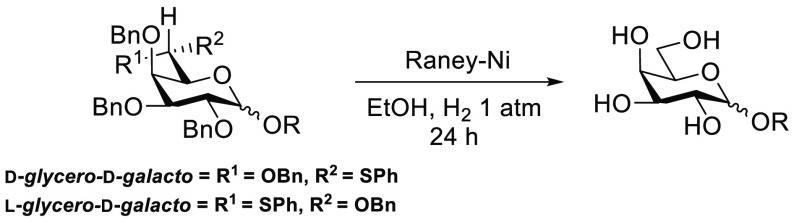

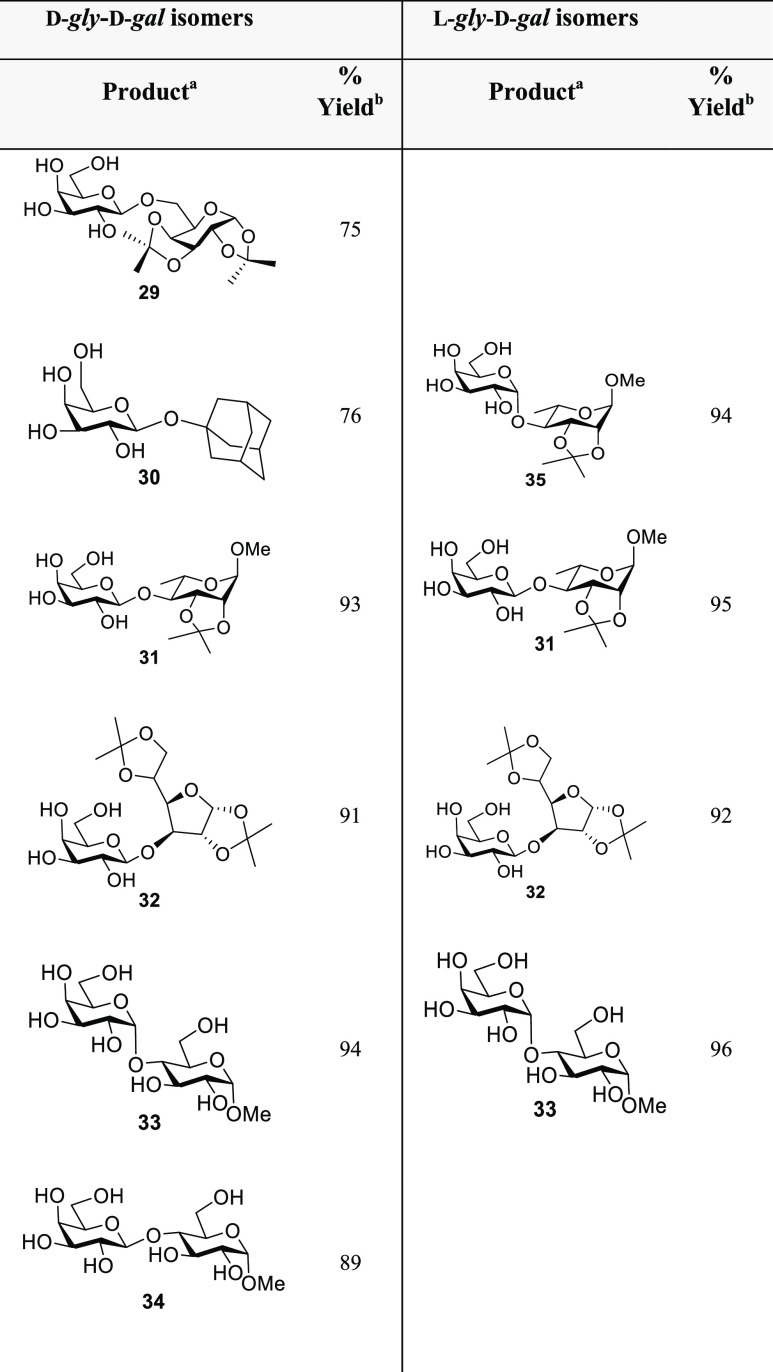
Desulfurization with Raney Nickel for the d-*glycero*-d-*galacto* and l-*glycero*-d-*galacto* Series

a All reactions were carried out at room temperature.

b Isolated yield.

**Table 4:**
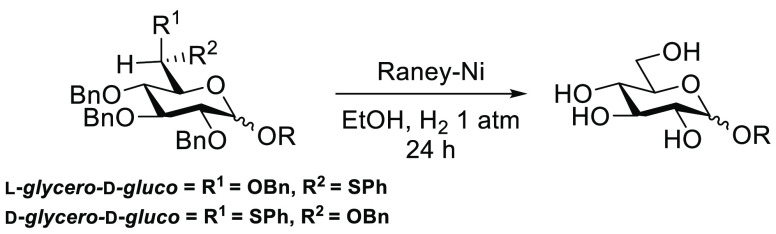

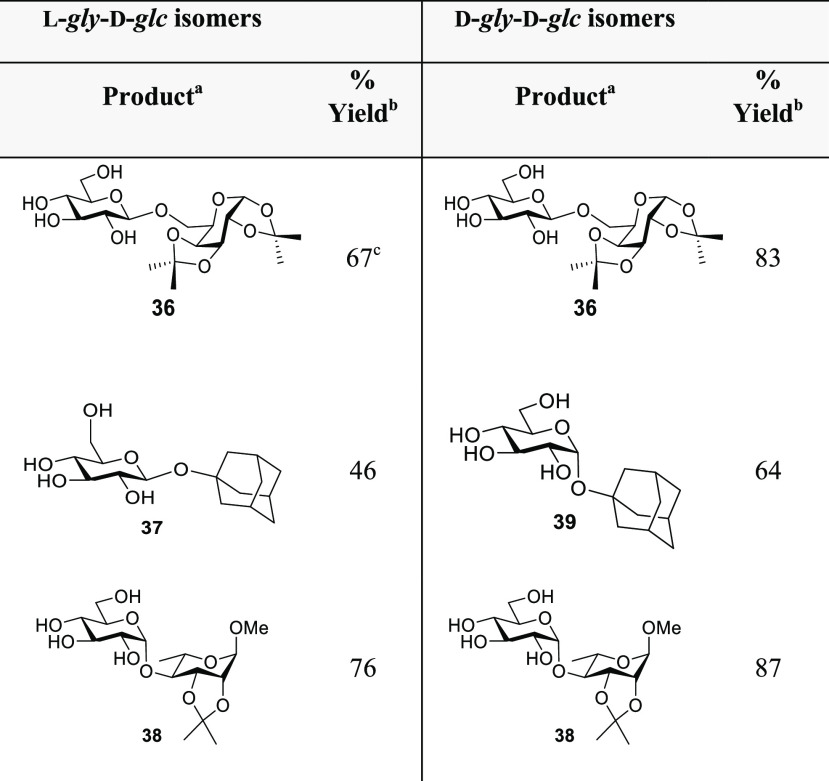
Desulfurization with Raney Nickel for the l-*glycero*-d-*gluco* and d-*glycero*-d-*gluco* Series

a All reactions were carried out at room temperature.

b Isolated yield.

c On a scale of 600 mg, desulfurization of **21β** gave 88% of **36**.

In these reactions, typically the thiophenyl ether moiety is cleaved rapidly while the *O*-benzyl groups are removed more slowly. Self-evidently, desulfurization and debenzylation of both side-chain isomers in either the *galacto* or *gluco* series of products give a single *galacto*- or *gluco*-pyranoside.

## Conclusions

Overall, the use of a dummy ligand is an effective strategy to bias the side-chain conformation of hexopyranosyl donors and provides a conceptually novel means of achieving moderate to good stereocontrol in the coupling of *galacto* and *gluco*pyranosyl donors to relatively reactive donors. Acetimidates **7a**–**d** are obtained in a minimum of steps from readily available allyl d-*galacto* and *gluco*pyranosides, and the final deprotection by desulfurization and debenzylation is achieved in a single step. Overall, the deprotected glycosides are obtained in eight steps from alcohols **1** and **2**.

## Experimental Section

### General Experimental Details

All reactions were carried out under argon unless otherwise stated. 1-Adamantanol and 1,2:5,6-di-*O*-isopropylidene-α-d-glucofuranose were purchased from commercial suppliers. Solvents used for column chromatography were of analytical grade and were purchased from commercial suppliers. Thin-layer chromatography was carried out with 250 μm glass-backed silica (XHL) plates. Detection of compounds was achieved by UV absorption (254 nm) and by staining with 10% sulfuric acid in ethanol. Purification of crude residues was performed by silica gel chromatography using a 230–400 mesh-grade 60 silica. Specific rotations [deg cm^3^ g^–1^ dm^–1^] were measured in chloroform on an automatic polarimeter with a path length of 10 cm. NMR spectra were recorded in C_6_D_6_, CD_2_Cl_2_, or CDCl_3_ at either 500, 600, or 900 MHz as indicated. High-resolution mass spectra (HRMS) were recorded in the electrospray mode using an orbitrap mass analyzer (Thermo Fisher ESI-Orbitrap). Chemical shifts (δ) are recorded in ppm, and multiplicities are abbreviated as follows: s (singlet), m (multiplet), br (broad), d (doublet), t (triplet), and q (quartet). Structural assignments were made with additional information from gCOSY, gHSQC, and gHMBC experiments. All manipulations and reactions using osmium tetroxide should be conducted in a well-ventilated fume hood.

### Preparation of Acceptors

#### Methyl 2,3,4-Tri-*O*-benzyl-α-d-glucopyranoside

This colorless oil was prepared according to the literature method and had spectral data consistent with the literature (1.4 g, 98%).^42−44^

#### 1,2:3,4-Di-*O*-isopropylidene-α-d-galactopyranose

This colorless syrup was prepared according to the literature method and had spectral data consistent with the literature (0.80 g, 74%).^45^

#### Methyl 2,3-*O*-Isopropylidene-α-l-rhamnopyranoside

This colorless oil was prepared according to the literature method and had spectral data consistent with the literature (1.1 g, 87%).^46,47^

#### Methyl 2,3,6-Tri-*O*-benzyl-α-d-glucopyranoside

This colorless syrup was prepared according to the literature method and had spectral data consistent with the literature (2.95 g, 98%).^42−44^

#### Allyl 2,3,4-Tri-*O*-benzyl-β*-*d-galactopyranoside (**1**)

This compound, a colorless oil, was prepared from β*-*d-galactose pentaacetate, starting with the synthesis of the allyl glycoside and deacetylation,^17^ followed by trityl group protection of O6, benzylation, and the final trityl group removal (3.67 g, 51%).^18,19^ The spectral data is consistent with the literature.^48^^**1**^**H NMR** (500 MHz, CDCl_3_) δ 7.40–7.26 (m, 15H, Ar-H), 6.04–5.90 (m, 1H, =CH), 5.33 (dq, *J* = 17.4, 1.7 Hz, 1H, =CH_2_), 5.19 (dq, *J* = 10.4, 1.5 Hz, 1H, =CH_2_), 5.02–4.92 (m, 2H, Ph-CH_2_-), 4.82 (d, *J* = 11.8 Hz, 1H, Ph-CH_2_-), 4.79 (d, *J* = 10.9 Hz, 1H, Ph-CH_2_-), 4.75 (d, *J* = 11.8 Hz, 1H, Ph-CH_2_-), 4.67 (d, *J* = 11.8 Hz, 1H, Ph-CH_2_-), 4.47–4.35 (m, 2H, CH), 4.14 (ddt, *J* = 13.0, 5.9, 1.5 Hz, 1H, -OCH_2a_-), 3.88 (dd, *J* = 9.8, 7.7 Hz, 1H, H2), 3.80–3.73 (m, 2H, CH), 3.57–3.47 (m, 2H, CH), 3.37 (dd, *J* = 6.8, 5.6 Hz, 1H, H5), 1.64 (s, 1H, OH). ^**13**^**C{**^**1**^**H} NMR** (126 MHz, CDCl_3_) δ 138.7 (C), 138.5 (C), 138.35 (C), 134.3 (CH), 128.7 (Ar), 128.5 (Ar), 128.4 (Ar), 128.3 (Ar), 128.0 (Ar), 127.8 (Ar), 127.7 (Ar), 127.6 (Ar), 117.2 (=CH_2_), 103.2 (CH1), 82.3 (CH3), 79.7 (CH2), 75.3 (CH5), 74.6 (CH6), 74.2 (CH4), 73.5 (CH_2_), 73.0 (CH_2_), 70.4 (OCH_2_), 62.1 (CH_2_). **HRMS (**ESI) *m*/*z*: [M + Na]^+^ calcd for C_30_H_34_O_6_Na 513.2247; found 513.2242.

#### Allyl 2,3,4-Tri-*O-*benzyl*-*β*-*d-glucopyranoside (**2**)

This compound, an amorphous white solid, was prepared from β*-*d-glucose pentaacetate, starting with the synthesis of the allyl glycoside and deacetylation,^17^ followed by trityl group protection of O6, benzylation, and the final trityl group removal (3.52 g, 31%).^18,19^ The spectral data is consistent with the literature.^49^^**1**^**H NMR** (500 MHz, CDCl_3_) δ 7.36–7.24 (m, 15H; Ar-H), 5.95 (ddt, *J* = 17.2, 10.5, 5.6 Hz, 1H; =CH), 5.34 (dq, *J* = 17.2, 1.6 Hz, 1H; =CH_2a_), 5.21 (dq, *J* = 10.5, 1.4 Hz, 1H; =CH_2b_), 4.98–4.89 (m, 2H; Ph-CH_2_-), 4.86 (d, *J* = 10.9 Hz, 1H; Ph-CH_2_-), 4.80 (d, *J* = 10.9 Hz, 1H; Ph-CH_2_-), 4.72 (d, *J* = 10.9 Hz, 1H; Ph-CH_2_-), 4.63 (d, *J* = 10.9 Hz, 1H; Ph-CH_2_-), 4.49 (d, *J* = 7.8 Hz; 1H, H1), 4.39 (ddt, *J* = 12.9, 5.6, 1.6 Hz, 1H; -OCH_2a_-), 4.15 (ddt, *J* = 12.9, 5.6, 1.4 Hz, 1H; -OCH_2b_-), 3.86 (d, *J* = 12.0 Hz, 1H; H-6a), 3.70 (d, *J* = 12.0 Hz, 1H; H-6b), 3.66 (t, *J* = 9.1 Hz, 1H; H3), 3.56 (app t, *J* = 9.1 Hz, 1H; H4), 3.44 (dd, *J* = 9.1, 7.8 Hz, 1H; H2), 3.35 (ddd, *J* = 9.1, 4.7, 2.8 Hz, 1H; H5), 1.87 (s, 1H; OH). ^**13**^**C{**^**1**^**H} NMR** (126 MHz, CDCl_3_) δ 138.6 (Ar-H), 138.4 (Ar-H), 138.1 (Ar-H), 134.0 (=CH), 128.6 (Ar-H), 128.5 (Ar-H), 128.3 (Ar-H), 128.2 (Ar-H), 128.0 (Ar-H), 127.97 (Ar-H), 127.8 (Ar-H), 127.7 (Ar-H), 117.6 (CH_2_), 102.9 (CH1), 84.6 (CH4), 82.4 (CH3), 77.7 (CH2), 75.8 (CH5), 75.2 (CH_2_-6), 75.12 (CH_2_), 75.07 (CH_2_), 70.8 (CH_2_), 62.2 (CH_2_). **HRMS (**ESI) *m*/*z*: [M + Na]^+^ calcd for C_30_H_34_O_6_Na 513.2247; found 513.2240.

### General Procedure for the Syntheses of Dibenzylacetals, (**GP1**)

To a stirred solution of alcohol (**1** or **2**, 1 equiv, 0.5 M) in anhydrous CH_2_Cl_2_ was added Dess–Martin periodinane (1.5 equiv). Stirring was continued for 1 h at room temperature. After the completion of the reaction as observed by thin-layer chromatography (TLC), saturated aqueous Na_2_S_2_O_3_ (50 mL) and saturated aqueous NaHCO_3_ (50 mL) were added to the reaction mixture, which was stirred for another 0.25 h. The organic layer was separated, and the aqueous layer was extracted with CH_2_Cl_2_ (3 × 50 mL). The combined organic layers were collected, dried over anhydrous Na_2_SO_4_, and concentrated under reduced pressure to afford a crude residue, which was used immediately without further purification.

A 0.5 M solution of crude aldehyde (1 equiv) in anhydrous acetonitrile was stirred with Na_2_SO_4_ (0.2 g/mmol) for 5 min before the addition of *p-*TSA (0.2 equiv) and subsequently the addition of CH(OBn)_3_ (2 equiv) in a dropwise manner. After complete addition, the reaction mixture was stirred at room temperature overnight; then, it was quenched with Et_3_N (2.0 mL), filtered, and concentrated under reduced pressure to afford a crude residue. Purification of this crude product by silica gel chromatography using 100% hexane to 5% ethyl acetate in hexane gave the dibenzylacetals.

#### Allyl 2,3,4,6,6-Penta-*O*-benzyl-β-d-galactopyranoside (**3**)

Prepared from compound **1** (3.67 g, 7.34 mmol) following general procedure **GP1**, eluting from silica gel with 5% ethyl acetate in hexanes. White amorphous solid (3.6 g, 71%) [α]_D_^21^ = +27.7 (*c* = 1.0, CHCl_3_). ^**1**^**H NMR** (500 MHz, CDCl_3_) δ 7.41–7.19 (m, 25H, ArH), 5.94 (ddt, *J* = 16.4, 10.9, 5.6 Hz, 1H, =CH), 5.29 (dq, *J* = 17.2, 1.8 Hz, 1H, =CH_2a_), 5.17 (dq, *J* = 10.5, 1.5 Hz, 1H, =CH_2b_), 5.02 (d, *J* = 7.4 Hz, 1H, H6), 5.00–4.92 (m, 2H, CH_2_), 4.82–4.73 (m, 3H, CH_2_), 4.74–4.63 (m, 3H, CH_2_), 4.48 (d, *J* = 11.5 Hz, 1H, CH_2_), 4.44–4.36 (m, 2H, H1, -OCH_2a_-), 4.26 (d, *J* = 11.3 Hz, 1H, CH_2_), 4.11 (dd, *J* = 13.0, 6.0 Hz, 1H, -OCH_2b_-), 4.03 (d, *J* = 2.9 Hz, 1H, H4), 3.88 (dd, *J* = 9.7, 7.7 Hz, 1H, H2), 3.52 (dd, *J* = 9.7, 2.9 Hz, 1H, H3), 3.49 (d, *J* = 7.3 Hz, 1H, H5); ^**13**^**C{**^**1**^**H} NMR** (126 MHz, CDCl_3_) δ 138.9 (C), 138.8 (C), 138.6 (C), 138.3 (C), 137.6 (C), 134.2 (=CH), 128.5 (Ar), 128.44 (Ar), 128.4 (Ar), 128.2 (Ar), 128.1 (Ar), 128.0 (Ar), 127.9 (Ar), 127.83 (Ar), 127.8 (Ar), 127.65 (Ar), 127.6 (Ar), 127.5 (Ar), 117.3 (=CH_2_), 103.2 (C1), 100.5 (C6), 82.4 (C3), 79.5 (C2), 75.7 (CH_2_), 75.3 (C5), 74.7 (CH_2_), 74.5 (C4), 73.3 (CH_2_), 70.3 (CH_2_), 69.7 (OCH_2_), 69.4 (CH_2_). **HRMS** (ESI) *m*/*z*: [M + Na]^+^ calcd for C_44_H_46_O_7_Na 709.3136; found, 709.3118.

#### Allyl 2,3,4,6,6-Penta-*O-*benzyl*-*β*-*d-glucopyranoside (**4**)

Prepared from compound **2** (3.52 g, 7.18 mmol) following general procedure **GP1**, eluting from silica gel with 5% ethyl acetate in hexanes. White amorphous solid (3.2 g, 65%) [α]_D_^21^ = +3.9 (*c* = 0.1, CHCl_3_). ^**1**^**H NMR** (500 MHz, CDCl_3_) δ 7.43–7.16 (m, 23H; Ar-H), 7.09 (dd, *J* = 7.0, 2.7 Hz, 2H; Ar-H), 5.96 (ddt, *J* = 17.3, 10.2, 5.5 Hz, 1H; =CH), 5.34 (dq, *J* = 17.3, 1.7 Hz, 1H; =CH_2a_), 5.20 (dq, *J* = 10.2, 1.7 Hz, 1H; =CH_2b_), 5.04 (d, *J* = 1.9 Hz, 1H; H6), 5.00 (d, *J* = 11.0 Hz, 1H; Ph-CH_2_-), 4.94 (d, *J* = 11.0 Hz, 1H; Ph-CH_2_-), 4.87–4.71 (m, 6H; Ph-CH_2_-), 4.59 (d, *J* = 12.0 Hz, 1H; Ph-CH_2_-), 4.47 (d, *J* = 7.7 Hz, 1H; H1), 4.43 (ddt, *J* = 13.1, 5.5, 1.7 Hz, 1H; -OCH_2a_-), 4.34 (d, *J* = 10.8 Hz, 1H; Ph-CH_2_-), 4.12 (ddt, *J* = 13.1, 5.5, 1.7 Hz, 1H; -OCH_2b_-), 3.73 (app t, *J* = 8.9 Hz, 1H; H4), 3.68 (t, *J* = 8.9 Hz, 1H; H3), 3.63 (dd, *J* = 8.9, 1.9 Hz, 1H; H5), 3.57 (dd, *J* = 8.9, 7.7 Hz, 1H; H2). ^**13**^**C{**^**1**^**H} NMR** (126 MHz, CDCl_3_) δ 138.46 (C), 138.4 (C), 138.22 (C), 138.2 (C), 137.5 (C), 134.0 (=CH), 128.34 (Ar), 128.30 (Ar), 128.24 (Ar), 128.2 (Ar), 128.10 (Ar), 128.07 (Ar), 127.77 (Ar), 127.7 (Ar), 127.59 (Ar), 127.56 (Ar), 127.54 (Ar), 127.5 (Ar), 127.4 (Ar), 117.2 (CH_2_), 102.7 (C1), 98.3 (C6), 84.6 (C3), 81.9 (C2), 78.1 (C4), 76.0 (C5), 75.6 (CH_2_), 74.6 (CH_2_), 74.4 (CH_2_), 70.1 (CH_2_), 68.8 (CH_2_), 68.4 (CH_2_). **HRMS (**ESI) *m*/*z*: [M + Na]^+^ calcd for C_44_H_46_O_7_Na 709.3136; found 709.3137.

### General Procedure for the Syntheses of Monothioacetals **5a**–**d**, (**GP2**)

Dibenzylacetal (**3** or **4**, 1 equiv) was coevaporated with toluene and dried overnight under high vacuum. The residue was dissolved in anhydrous toluene to give a 0.2 M solution and cooled down to −78 °C. Thiophenol (1.5 equiv) was added at the same temperature before. BF_3_·OEt_2_ (1.5 equiv) was added in a dropwise manner. After complete addition, the reaction mixture was stirred for 10 min at the same temperature and then gradually brought to −20 °C over a period of 3 h. The reaction mixture was stirred at −20 °C for 0.5 h and then quenched with saturated aqueous NaHCO_3_ (20 mL) and extracted with EtOAc (3 × 20 mL). The combined organic layers were collected, dried over anhydrous sodium sulfate, and concentrated under reduced pressure to afford a crude residue, which was purified by silica gel chromatography using 5–10% ethyl acetate and hexane as eluents to afford the corresponding monothioacetals.

#### Allyl (6*R*)-6-Phenylthio-2,3,4,6-tetra-*O-*benzyl-β-d-galactopyranoside **5a**

Prepared from compound **3** (2.0 g, 2.91 mmol) following general procedure **GP2**, eluting from silica gel with 5% ethyl acetate in hexanes. White amorphous solid (0.68 g, 34%) [α]_D_^23^ = −32.1 (*c* = 1.0, CHCl_3_). ^**1**^**H NMR** (500 MHz, CD_2_Cl_2_) δ 7.57–7.48 (m, 2H, H*o*-SPh), 7.39–7.19 (m, 24H, Ar-H), 6.02 (dddd, *J* = 17.1, 10.7, 6.0, 5.0 Hz, 1H, =CH), 5.37 (dq, *J* = 17.1, 1.7 Hz, 1H, =CH_2a (trans)_), 5.21 (dq, *J* = 10.7, 1.5 Hz, 1H, =CH_2a (cis)_), 5.14 (d, *J* = 11.0 Hz, 1H, CH_2_-O^6^), 5.00 (d, *J* = 8.9 Hz, 1H, H6), 4.95 (d, *J* = 11.0 Hz, 1H, CH_2_-O^4^), 4.88 (d, *J* = 11.0 Hz, 1H, CH_2_-O^3^), 4.71 (d, *J* = 11.0 Hz, 1H, CH_2_-O^3^), 4.68 (s, 2H, CH_2_-O^2^), 4.49 (ddt, *J* = 13.0, 5.0, 1.7 Hz, 1H, -O-CH_2a_-), 4.40 (d, *J* = 11.0 Hz, 1H, CH_2_-O^4^), 4.32 (d, *J* = 11.0 Hz, 1H, CH_2_-O^6^), 4.26 (d, *J* = 7.7 Hz, 1H, H1), 4.18 (ddt, *J* = 13.0, 6.0, 1.5 Hz, 1H, -O-CH_2b_-), 4.06 (dd, *J* = 3.0, 1.0 Hz, 1H, H4), 3.73 (dd, *J* = 9.7, 7.7 Hz, 1H, H2), 3.41 (dd, *J* = 9.7, 2.9 Hz, 1H, H3), 3.19 (dd, *J* = 8.9, 1.1 Hz, 1H, H5). ^**13**^**C {**^**1**^**H} NMR (126 MHz, CD**_**2**_**Cl**_**2**_**)** δ 139.1 (C), 139.0 (C), 138.7 (C), 137.2 (C), 134.7 (CH_*o-*SPh_), 134.5 (=CH), 131.8 (C), 128.9 (Ar), 128.5 (Ar), 128.4 (Ar), 128.28 (Ar), 128.26 (Ar), 128.19 (Ar), 128.18 (Ar), 128.08 (Ar), 128.01 (Ar), 127.6 (Ar), 127.54 (Ar), 127.50 (Ar), 127.4 (Ar), 116.8 (=CH_2_), 102.8 (C1), 87.0 (C6), 82.3 (C3), 80.0 (C2), 75.0 (CH_2_), 74.8 (CH_2_), 74.5 (C4), 73.4 (CH_2_), 73.0 (C5), 70.3 (CH_2_), 70.2 (OCH_2_). **HRMS** (ESI) *m*/*z*: [M + Na]^+^ calcd for C_43_H_44_O_6_SNa 711.2750; found 711.2734.

#### Allyl (6*S*)-6-Phenylthio-2,3,4,6-tetra-*O-*benzyl-β-d-galactopyranoside **5b**

Prepared from compound **3** (2.0 g, 2.91 mmol) following general procedure **GP2**, eluting from silica gel with 5% ethyl acetate in hexanes. White amorphous solid (0.82 g, 41%). [α]_D_^23^ = +22.8 (*c* = 2.0, CHCl_3_) ^**1**^**H NMR** (500 MHz, C_6_D_6_) δ 7.47–7.41 (m, 2H, *o*-SPh), 7.37–7.31 (m, 2H, ArH), 7.32–7.21 (m, 6H, ArH), 7.15–6.97 (m, 12H, ArH), 6.96–6.85 (m, 3H, *m,p*-SPh), 5.87–5.70 (m, 1H, =CH), 5.37–5.27 (m, 2H, H6, CH2), 5.19 (dq, *J* = 17.4, 1.8 Hz, 1H, =CH_2a_), 4.98–4.89 (m, 2H, CH_2_, =CH_2b_), 4.77 (d, *J* = 11.7 Hz, 1H, CH_2_), 4.73 (d, *J* = 10.9 Hz, 1H, CH_2_), 4.65 (dd, *J* = 11.8, 5.3 Hz, 2H, CH_2_), 4.54 (d, *J* = 12.2 Hz, 1H, CH_2_), 4.43 (d, *J* = 11.7 Hz, 1H, CH_2_), 4.38 (d, *J* = 3.0 Hz, 1H, H4), 4.36–4.30 (m, 1H, -OCH_2a_-), 4.17 (d, *J* = 7.7 Hz, 1H, H1), 4.07 (dd, *J* = 9.7, 7.6 Hz, 1H, H2), 3.95 (ddt, *J* = 13.2, 5.9, 1.7 Hz, 1H, -OCH_2b_-), 3.41 (d, *J* = 8.8 Hz, 1H, H5), 3.27 (dd, *J* = 9.7, 2.9 Hz, 1H, H3). ^**13**^**C{**^**1**^**H} NMR** (126 MHz, C_6_D_6_) δ 139.5 (C), 139.3 (C), 139.0 (C), 138.0 (C), 134.6 (=CH), 133.5 (Ar), 132.7 (Ar), 128.9 (Ar), 128.4 (Ar), 128.29 (Ar), 128.2 (Ar), 128.1 (Ar), 128.0 (Ar), 127.97 (Ar), 127.91 (Ar), 127.86 (Ar), 127.78 (Ar), 127.73 (Ar), 127.68 (Ar), 127.6 (Ar), 127.5 (Ar), 127.4 (Ar), 127.2 (Ar), 116.3 (=CH_2_), 103.3 (C1), 87.2 (C6), 82.2 (C3), 79.7 (C2), 76.1 (C4), 76.0 (C5), 75.3 (CH_2_), 74.7 (CH_2_), 73.3 (CH_2_), 70.7 (CH_2_), 69.7 (OCH_2_). **HRMS** (ESI) *m*/*z*: [M + Na]^+^ calcd for C_43_H_44_O_6_SNa 711.2750; found 711.2750.

#### Allyl (6*S*)-6-Phenylthio-2,3,4,6-tetra-*O-*benzyl-β-d-glucopyranoside **5c**

Prepared from compound **4** (1.6 g, 2.33 mmol) following general procedure **GP2**, eluting from silica gel with 5% ethyl acetate. Colorless syrup (0.923 g, 57%). [α]_D_^20^ = +6.3 (*c* = 2.0, CHCl_3_). ^**1**^**H NMR** (500 MHz, C_6_D_6_) δ 7.48–7.44 (m, 2H, H*o-*SPh), 7.37–7.27 (m, 2H, Ar-H), 7.26–7.20 (m, 2H, Ar-H), 7.09–6.84 (m, 19H, Ar-H), 5.81 (ddt, *J* = 17.1, 10.7, 6.1, 5.0 Hz, 1H, =CH), 5.59 (d, *J* = 2.1 Hz, 1H, H6), 5.27 (dd, *J* = 17.1, 1.5 Hz, 1H, =CH_2a_), 5.03–4.98 (m, 2H, =CH_2b_, CH_2_), 4.93 (m, 2H, CH_2_), 4.83 (d, *J* = 11.3 Hz, 1H, CH_2_), 4.72 (d, *J* = 11.3 Hz, 1H, CH_2_), 4.66 (d, *J* = 11.3 Hz, 1H, CH_2_), 4.53 (d, *J* = 11.6 Hz, 1H, CH_2_), 4.47 (d, *J* = 11.6 Hz, 1H, CH_2_), 4.42 (d, *J* = 7.2 Hz, 1H, H1), 4.36 (ddt, *J* = 13.2, 5.0, 1.5 Hz, 1H, -OCH_2a_-), 4.04 (ddt, *J* = 13.2, 6.1, 1.5 Hz, 1H, -OCH_2b_-), 3.94 (dd, *J* = 9.7, 8.4 Hz, 1H, H4), 3.75 (dd, *J* = 9.7, 2.1 Hz, 1H, H5), 3.62 (m, 2H, H2, H3). ^**13**^**C{**^**1**^**H} NMR** (126 MHz, C_6_D_6_) δ 139.17 (C), 139.10 (C), 139.0 (C), 137.6 (C), 136.9 (C), 134.3 (=CH), 131.3 (SPh-*C*H_*o*_), 129.0 (Ar), 128.26 (Ar), 128.21 (Ar), 128.18 (Ar), 128.0 (Ar), 127.9 (Ar), 127.7 (Ar), 127.5 (Ar), 127.4 (Ar), 127.3 (Ar), 126.7 (Ar), 116.8 (=CH_2_), 103.3 (C1), 88.4 (C6), 84.8 (C3), 82.4 (C2), 79.6 (C5), 78.5 (C4), 75.3 (CH_2_), 74.6 (CH_2_), 74.5 (CH_2_), 69.9 (CH_2_), 69.8 (-OCH_2_-). **HRMS (**ESI) *m*/*z*: [M + Na]^+^ calcd for C_43_H_44_O_6_NaS 711.2751; found 711.2741.

#### Allyl (6*R*)-6-Phenylthio-2,3,4,6-tetra-*O-*benzyl-β-d-glucopyranoside **5d**

Prepared from compound **4** (1.6 g, 2.33 mmol) following general procedure **GP2**, eluting from silica gel with 5% ethyl acetate in hexanes. White amorphous solid (0.192 g, 12%). [α]_D_^20^ = +6.6 (*c* = 0.2, CHCl_3_). ^**1**^**H NMR** (500 MHz, C_6_D_6_) δ 7.43–7.37 (m, 2H, H*o-*SPh), 7.31–7.28 (m, 2H, Ar-H), 7.26–7.21 (m, 2H, Ar-H), 7.18–7.12 (m, 2H, Ar-H), 7.12–6.91 (m, 14H, Ar-H), 6.90–6.81 (m, 3H, Ar-H), 5.74 (ddt, *J* = 16.4, 10.7, 5.4 Hz, 1H, =C-H), 5.62 (d, *J* = 1.5 Hz, 1H, H6), 5.17 (dq, *J* = 16.4, 1.8 Hz, 1H, =CH_2a_), 5.00–4.93 (m, 3H, =CH_2b_, CH_2_), 4.89 (d, *J* = 11.4 Hz, 1H, CH_2_), 4.79 (d, *J* = 11.9 Hz, 1H, CH_2_), 4.71–4.68 (m, 2H, CH_2_), 4.64 (d, *J* = 11.4 Hz, 1H, CH_2_), 4.45 (d, *J* = 11.4 Hz, 1H, CH_2_), 4.38 (d, *J* = 7.5 Hz, 1H, H1), 4.25 (ddt, *J* = 13.0, 5.4, 1.8 Hz, 1H, -OCH_2a_-), 4.01 (dd, *J* = 9.6, 8.5 Hz, 1H, H4), 3.94–3.85 (m, 2H, H5, -OCH_2b_-), 3.67 (dd, *J* = 9.1, 8.5 Hz, 1H, H3), 3.62 (d, *J* = 9.1, 7.5 Hz, 1H, H2). ^**13**^**C{**^**1**^**H} NMR** (126 MHz, C_6_D_6_) δ 139.09 (C), 139.03 (C), 138.7 (C), 138.0 (C), 136.5 (C_*i*_-SPh), 134.3 (=CH-), 131.4 (C_*o*_H-SPh), 128.9 (Ar-H), 128.3 (Ar), 128.24 (Ar), 128.21 (Ar), 128.0 (Ar), 127.8 (Ar), 127.6 (Ar), 127.5 (Ar), 127.45 (Ar), 127.42(Ar), 127.37 (Ar), 127.31 (Ar), 126.6 (Ar), 116.6 (=CH_2_), 102.8 (C1), 88.9 (C6), 84.6 (C3), 82.2 (C2), 79.0 (C4), 78.7 (C5), 75.1 (CH_2_), 74.6 (CH_2_), 74.5 (CH_2_), 69.8 (CH_2_), 69.5 (CH_2_). **HRMS (**ESI) *m*/*z*: [M + Na]^+^ calcd for C_43_H_44_O_6_NaS 711.2751; found 711.2741.

### General Procedure for Deallylation, **GP3**

To a stirred solution of allyl glycoside (1.0 equiv) and RuCl_2_(PPh_3_)_3_ (0.1 equiv) in ethyl alcohol (0.1 M) was added DBU (1.0 equiv). The resulting mixture was heated to reflux for 5 h before the solvent was evaporated under reduced pressure to afford a crude residue that was taken up in CH_2_Cl_2_ (50 mL) and washed with water (50 mL). The organic layer was separated, and the aqueous layer was extracted with CH_2_Cl_2_ (2 × 50 mL). The organic layers were combined, dried over Na_2_SO_4_ addition, filtered, and concentrated under reduced pressure to give a crude mixture, which was purified with silica gel column chromatography using 10% ethyl acetate in hexane as an eluent to afford a colorless syrup.

A stirred 0.1 M solution of the compound prepared in the previous step in 1,4-dioxane/water (4:1) was treated with NMO (3.0 equiv) and OsO_4_ (2% in *t-*butanol, 2 drops) and stirred until completion (monitored by TLC) at room temperature. The reaction was quenched with saturated aqueous Na_2_S_2_O_3_, diluted with ethyl acetate, washed with brine, and dried over Na_2_SO_4_. The organic layer was filtered and concentrated under reduced pressure to afford a crude residue that was purified by silica gel column chromatography using 15–25% ethyl acetate in hexane as an eluent to afford a colorless syrup as a mixture of anomers.

#### (6*R*)-6-Phenylthio-2,3,4,6-tetra-*O-*benzyl-α,β-d-galactopyranose (**6a**)

Prepared using general protocol **GP3** from **5a** (0.7 g, 1.02 mmol) as a mixture of 2.4:1, *ax:eq* anomers after eluting from silica gel with 15–25% gradient of ethyl acetate in hexanes (0.53 g, 81%).

Mixture of anomers: colorless syrup. ^**1**^**H NMR** (500 MHz, CDCl_3_) δ 7.67–7.58 (m, 7H), 7.40–7.26 (m, 35H), 5.39 (d, *J* = 3.7 Hz, 2.4H), 5.19–5.12 (m, 3.5H), 5.09–4.99 (m, 7.1H), 4.93 (d, *J* = 11.0 Hz, 1H), 4.85–4.76 (m, 6.1H), 4.75–4.68 (m, 7.1H), 4.56 (dd, *J* = 7.4, 4.1 Hz, 1H), 4.48–4.40 (m, 3.5H), 4.32–4.26 (m, 3.5H), 4.17 (d, *J* = 2.7 Hz, 2.4H), 4.10–4.05 (m, 3.5H), 3.91 (d, *J* = 9.0 Hz, 2.4H), 3.88 (dd, *J* = 9.9, 2.7 Hz, 2.4H), 3.74 (d, *J* = 6.2 Hz, 1H), 3.45 (dd, *J* = 9.6, 2.8 Hz, 1H), 3.20 (d, *J* = 8.8 Hz, 1H), 2.77 (d, *J* = 2.5 Hz, 2.2H). ^**13**^**C{**^**1**^**H} NMR** (126 MHz, CDCl_3_) δ 139.10, 138.9, 138.8, 138.7, 138.5, 138.4, 137.3, 137.1, 135.4, 135.0, 131.9, 131.2, 129.0, 128.8, 128.68, 128.65, 128.5, 128.48, 128.47, 128.40, 128.38, 128.37, 128.35, 128.30, 128.16, 128.13, 128.10, 127.88, 127.8, 127.68, 127.66, 127.64, 127.61, 127.57, 127.55, 127.53, 127.51, 127.49, 97.9, 92.0, 87.1, 86.8, 82.6, 81.0, 79.2, 76.6, 75.4, 75.2, 74.8, 74.7, 74.3, 73.8, 73.5, 73.0, 70.4, 70.3, 70.1. **HRMS** (ESI) *m*/*z*: [M + Na]^+^ calcd for C_40_H_40_O_6_SNa 671.2438; found 671.2427.

#### (6*S*)-6-Phenylthio-2,3,4,6-tetra-*O-*benzyl-α,β-d-galactopyranose (**6b**)

Prepared using general protocol **GP3** from **5b** (0.9 g, 1.31 mmol) as a mixture of 2.4:1, *ax:eq* anomers after eluting from silica gel with 15–25% gradient of ethyl acetate in hexanes (0.74 g, 87%).

Mixture of anomers: colorless syrup. ^**1**^**H NMR** (500 MHz, CDCl_3_) δ 7.73–6.65 (m, 85H), 5.33 (t, *J* = 3.1 Hz, 2H), 5.18–5.09 (m, 6.5H), 4.99 (d, *J* = 11.6 Hz, 1H), 4.96–4.91 (m, 3.2H), 4.87 (d, *J* = 11.9 Hz, 2.3H), 4.85–4.59 (m, 16.9H), 4.48–4.42 (m, 3H), 4.35 (d, *J* = 2.8 Hz, 1H), 4.21 (d, *J* = 5.5 Hz, 1H), 4.14–4.03 (m, 4.2H), 3.88 (dd, *J* = 10.0, 2.7 Hz, 2H), 3.80 (dd, *J* = 9.7, 7.5 Hz, 1H), 3.65 (d, *J* = 2.8 Hz, 2H), 3.38 (d, *J* = 9.0 Hz, 1H), 3.33 (dd, *J* = 9.7, 2.8 Hz, 1H). ^13^C NMR (126 MHz, CDCl_3_) δ 138.99, 138.91, 138.90, 138.8, 138.6, 138.5, 137.2, 137.1, 133.6, 133.0, 132.7, 131.8, 129.17, 129.16, 128.58, 128.57, 128.56, 128.50, 128.49, 128.47, 128.46, 128.38, 128.33, 128.31, 128.2, 128.1, 128.04, 128.00, 127.9, 127.88, 127.86, 127.81, 127.79, 127.75, 127.74, 127.6, 127.53, 127.51, 127.0, 98.0, 91.9, 86.7, 86.1, 82.2, 80.42, 79.2, 76.3, 76.1, 75.6, 75.0, 74.97, 74.95, 74.8, 73.35, 73.33, 73.2, 71.5, 70.4, 69.9. **HRMS** (ESI) *m*/*z*: [M + Na]^+^ calcd for C_40_H_40_O_6_SNa 671.2438; found 671.2430.

#### (6*S*)-6-Phenylthio-2,3,4,6-tetra-*O-*benzyl-α,β-d-glucopyranose **6c**

Prepared using general protocol **GP3** from **5c** (0.9 g, 1.30 mmol) as a mixture of 1.5:1, *ax:eq* anomers after eluting from silica gel with 15–25% gradient of ethyl acetate in hexanes (0.46 g, 54%).

Mixture of anomers: colorless syrup. ^**1**^**H NMR** (500 MHz, CDCl_3_) δ 7.55–7.49 (m, 5H), 7.39–7.33 (m, 7H), 7.32–7.08 (m, 40H), 7.06–7.01 (m, 5H), 5.35–5.31 (m, 3H), 5.30 (t, *J* = 2.9 Hz, 1.5H, H1 α anomer), 5.00–4.87 (m, 5H), 4.86–4.67 (m, 12.5H), 4.54 (d, *J* = 11.8 Hz, 1H, CH_2_), 4.43 (d, *J* = 11.8 Hz, 1.5H, CH_2_), 4.39 (d, *J* = 9.9 Hz, 1.5H, CH), 4.21 (d, *J* = 11.0 Hz, 1H, CH_2_), 4.17 (d, *J* = 11.3 Hz, 1.5H, CH_2_), 4.13 (t, *J* = 7.1 Hz, 1H, H1 β anomer), 4.02 (t, *J* = 9.3 Hz, 1.5H, CH), 3.83 (dd, *J* = 9.7, 1.7 Hz, 1H, CH), 3.75 (t, *J* = 9.2 Hz, 1H, CH), 3.70 (d, *J* = 9.5 Hz, 1H, CH), 3.66 (t, *J* = 9.2 Hz, 1.5H, CH), 3.61 (dd, *J* = 9.6, 3.5 Hz, 1.5H, CH), 3.45 (t, *J* = 8.6 Hz, 1.5H, CH), 3.37 (d, *J* = 5.2 Hz, 1.5H, CH), 3.12 (d, *J* = 2.9 Hz, 1.5H, CH). ^**13**^**C{**^**1**^**H} NMR** (126 MHz, CDCl_3_) δ 138.6 (C), 138.5 (C), 138.4 (C), 138.3 (C), 138.0 (C), 137.0 (C), 136.9 (C), 136.1 (C), 135.7 (C), 132.5 (Ar), 132.4 (Ar), 129.22 (Ar), 129.18 (Ar), 128.7 (Ar), 128.65 (Ar), 128.61 (Ar), 128.55 (Ar), 128.50 (Ar), 128.46 (Ar), 128.4 (Ar), 128.3 (Ar), 128.2 (Ar), 128.1 (Ar), 128.0 (Ar), 127.8 (Ar), 127.68 (Ar), 127.6 (Ar), 127.5 (Ar), 98.1 (C1 β anomer), 91.5 (C1 α anomer), 88.2 (CH), 87.2 (CH), 84.7 (CH), 83.2 (CH), 81.9 (CH), 80.0 (CH), 79.5 (CH), 78.4 (CH), 78.2 (CH), 76.0 (CH_2_), 75.8 (CH_2_), 75.3 (CH_2_), 74.8 (CH_2_), 74.7 (CH_2_), 73.4 (CH), 70.6 (CH_2_), 70.2 (CH_2_). **HRMS (**ESI) *m*/*z*: [M + Na]^+^ calcd for C_40_H_40_O_6_NaS 671.2438; found 671.2432.

#### (6*R*)-6-Phenylthio-2,3,4,6-tetra-*O-*benzyl-α,β-d-glucopyranose **6d**

Prepared using general protocol **GP3** from **5d** (0.192 g, 0.28 mmol) as a mixture of 9.8:1, *ax:eq* anomers after eluting from silica gel with 15–25% gradient of ethyl acetate in hexanes (0.099 g, 55%).

Mixture of anomers: colorless syrup. ^**1**^**H NMR** (500 MHz, CDCl_3_) δ 7.41–7.13 (m, 23H, Ar-H), 7.01–6.98 (m, 2H, Ar-H), 5.38 (d, *J* = 1.4 Hz, 1H, H6), 5.24 (t, *J* = 3.2 Hz, 1H, H1), 4.97 (d, *J* = 11.0 Hz, 1H, CH_2_), 4.86 (d, *J* = 11.0 Hz, 1H, CH_2_), 4.83–4.74 (m, 3H, CH_2_), 4.71 (d, *J* = 12.0 Hz, 1H), 4.63 (d, *J* = 12.0 Hz, 1H, CH_2_), 4.43–4.36 (m, 2H, CH_2_, H5), 4.03 (app t, *J* = 9.2 Hz, 1H, H4), 3.79 (t, *J* = 9.2 Hz, 1H, H3), 3.60 (dd, *J* = 9.2, 3.2 Hz, 1H, H2), 2.91 (d, *J* = 3.2 Hz, 1H, OH). ^**13**^**C{**^**1**^**H} NMR** (126 MHz, CDCl_3_) δ 138.6 (C), 138.2 (C), 137.9 (C), 137.5 (C), 135.6 (C), 131.9 (Ar), 129.1 (Ar), 128.6 (Ar), 128.5 (Ar), 128.4 (Ar), 128.4 (Ar), 128.11 (Ar), 128.10 (Ar), 128.08 (Ar), 128.0 (Ar), 127.8 (Ar), 127.6 (Ar), 127.5 (Ar), 127.2 (Ar), 91.3 (C1), 89.0 (C6), 81.6 (C4), 80.2 (C2), 79.1 (C3), 75.7 (CH_2_), 74.9 (CH_2_), 74.1 (CH_2_), 73.3 (C5), 70.0 (CH_2_). **HRMS (**ESI) *m*/*z*: [M + Na]^+^ calcd for C_40_H_40_O_6_NaS 671.2438 found 671.2424.

### General Procedure for Trichloroacetimidate Preparation, **GP4**

The hemiacetal (1.0 equiv) was dissolved in CH_2_Cl_2_ (0.2 M) and cooled down to 0 °C before trichloroacetonitrile (5.0 equiv) and then DBU (1 drop) were added, and the reaction mixture was stirred for 0.5 h at the same temperature. After completion, the reaction mixture was diluted with dichloromethane and washed with water. The aqueous layer was extracted with CH_2_Cl_2_ (2 × 20 mL). The organic layers combined were dried over Na_2_SO_4_ and concentrated under reduced pressure. The crude product was passed through a silica gel column, eluting with 10–20% ethyl acetate in hexane to give the trichloroacetimidate, which was used in the next step without further purification.

#### (6*R*)-6-Phenylthio-2,3,4,6-tetra-*O*-benzyl-α,β-d-galactopyranosyl Trichloroacetimidate (**7a**)

Prepared (0.55 g, 85%) by following the general protocol **GP4** from hemiacetal **6a** (0.53 g, 0.82 mmol) eluting with 10–20% ethyl acetate in hexane to give the trichloroacetimidate. Colorless syrup. **HRMS (**ESI) *m*/*z*: [M + Na]^+^ calcd for C_42_H_40_O_6_NCl_3_NaS 814.1534; found 814.1517.

#### (6*S*)-6-Phenylthio-2,3,4,6-tetra-*O*-benzyl-α,β-d-galactopyranosyl Trichloroacetimidate (**7b**)

Prepared (0.80 g, 89%) by following the general protocol **GP4** from hemiacetal **6b** (0.74 g, 1.14 mmol) eluting with 10–20% ethyl acetate in hexane to give the trichloroacetimidate. Colorless syrup. **HRMS (**ESI) *m*/*z*: [M + Na]^+^ calcd for C_42_H_40_O_6_NCl_3_NaS 814.1534; found 814.1506.

#### (6*S*)-6-Phenylthio-2,3,4,6-tetra-*O*-benzyl-α,β-d-glucopyranosyl Trichloroacetimidate (**7c**)

Prepared (0.48 g, 86%) by following the general protocol **GP4** from hemiacetal **6c** (0.46 g, 0.71 mmol) eluting with 10–20% ethyl acetate in hexane to give the trichloroacetimidate. Colorless syrup. **HRMS (**ESI) *m*/*z*: [M + Na]^+^ calcd for C_42_H_40_O_6_NCl_3_NaS 814.1534; found 814.1529.

#### (6*R*)-6-Phenylthio-2,3,4,6-tetra-*O*-benzyl-α,β-d-glucopyranosyl Trichloroacetimidate (**7d**)

Prepared (0.108 g, 90%) by following the general protocol **GP4** from hemiacetal **6d** (0.099 g, 0.15 mmol) eluting with 10–20% ethyl acetate in hexane to give the trichloroacetimidate. Colorless syrup. **HRMS (**ESI) *m*/*z*: [M + Na]^+^ calcd for C_42_H_40_O_6_NCl_3_NaS 814.1534; found 814.1511.

### General Procedure for Glycosylation Reaction, **GP5**

A mixture of the donor (1.0 equiv) and acceptor (1.2 equiv) was coevaporated with toluene twice, then taken up in anhydrous CH_2_Cl_2_ (0.15 M) and stirred for 1 h with activated 4 Å AWMS (2 g/mmol of the donor) at room temperature under argon before cooling to −78 °C. After 0.5 h of continuous stirring, the reaction mixture was treated with TMSOTf (0.2 equiv) and stirred for 4–5 h at −78 °C before it was quenched with triethylamine (0.2 mL). The reaction mixture was diluted with dichloromethane (10 mL), filtered through a pad of celite, and washed with saturated aqueous NaHCO_3_. The organic layer was separated, dried over Na_2_SO_4_, filtered, and concentrated under reduced pressure. Purification by flash column chromatography on silica gel (hexane/ethyl acetate) afforded the corresponding α/β-glycopyranosides. The anomeric ratio of the products was determined by integration of the anomeric signals in the^1^H NMR spectra of the crude product mixtures unless otherwise stated.

#### Methyl (6*R*)-6-Phenylthio-2,3,4,6-tetra-*O-*benzyl-β-d-galactopyranosyl-(1→6)-2,3,4-tri-*O*-benzyl-α-d-glucopyranoside (**8**)

Compound **8β** was obtained (31.1 mg, 75%) as a single isomer from the reaction of donor **7a** (30.0 mg, 37.8 μmol) and acceptor (21.1 mg, 45.4 μmol) by following the general procedure **GP5** for glycosylation (eluting with 10% ethyl acetate in hexanes).

Colorless syrup. [α]_D_^20^ = −14.0 (*c* = 1.9, CHCl_3_). ^**1**^**H NMR** (500 MHz, CDCl_3_) δ 7.50 (d, *J* = 7.5 Hz, 2H, ArH), 7.39–7.21 (m, 31H, ArH), 7.21–7.14 (m, 7H, ArH), 5.12 (d, *J* = 11.1 Hz, 1H, -CH_2_), 5.03–4.94 (m, 3H, H6′, -CH_2_), 4.91 (d, *J* = 10.9 Hz, 1H, CH_2_), 4.80 (m, 2H, CH_2_), 4.77–4.70 (m, 2H, CH_2_), 4.70–4.66 (m, 2H, CH_2_), 4.63 (d, *J* = 3.6 Hz, 1H, H1), 4.54 (d, *J* = 11.0 Hz, 1H, CH_2_), 4.40 (d, *J* = 11.4 Hz, 1H, CH_2_), 4.34 (dd, *J* = 10.9, 2.2 Hz, 1H, H6b), 4.25 (d, *J* = 11.0 Hz, 1H, CH_2_), 4.18 (d, *J* = 7.7 Hz, 1H, H1′), 4.05–3.99 (m, 2H, CH), 3.97–3.91 (m, 1H, CH), 3.87 (dd, *J* = 9.7, 7.7 Hz, 1H, H2′), 3.73 (dd, *J* = 10.9, 5.2 Hz, 1H, H6a), 3.58–3.50 (m, 2H, H2, CH), 3.38 (dd, *J* = 9.7, 2.8 Hz, 1H, H3′), 3.32 (s, 3H, OCH_3_), 3.13 (d, *J* = 8.8 Hz, 1H, H5′). ^**13**^**C{**^**1**^**H} NMR** (126 MHz, CDCl_3_) δ 139.04 (C), 139.02 (C), 138.8 (C), 138.7 (C), 138.5 (C), 138.4 (C), 138.3 (C), 137.1 (C), 134.8 (Ar), 132.5 (Ar), 131.9 (Ar), 128.8 (Ar), 128.6 (Ar), 128.5 (Ar), 128.45 (Ar), 128.43 (Ar), 128.40 (Ar), 128.3 (Ar), 128.28 (Ar), 128.24 (Ar), 128.13 (Ar), 128.11 (Ar), 128.08 (Ar), 128.04 (Ar), 128.00 (Ar), 127.9 (Ar), 127.65 (Ar), 127.60 (Ar), 127.58 (Ar), 127.53 (Ar), 127.51 (Ar), 127.46 (Ar), 127.40 (Ar), 104.3 (C1′), 98.1 (C1), 87.0 (C6′), 82.7 (CH), 82.2 (CH), 80.0 (CH), 79.4 (CH), 78.4 (CH), 75.8 (CH_2_), 75.3 (CH_2_), 75.1 (CH_2_), 74.7 (CH_2_), 74.3 (CH), 73.5 (CH), 73.4 (CH_2_), 73.0 (CH_2_), 70.3 (CH_2_), 70.0 (CH), 68.9 (C6), 55.3 (OCH_3_). **HRMS** (ESI) *m*/*z*: [M + Na]^+^ calcd for C_68_H_70_O_11_SNa 1117.4531; found 1117.4495.

#### (6*R*)-6-Phenylthio-2,3,4,6-tetra-*O*-benzyl-β-d-galactopyranosyl-(1→6)-1,2:3,4-*O*-diisopropylidene-α-d-galactopyranose (**9**)

Compound **9β** was obtained (34.4 mg, 68%) as a single isomer from the reaction of donor **7a** (45.0 mg, 56.7 μmol) and acceptor (17.7 mg, 68.0 μmol) by following the general procedure **GP5** for glycosylation (eluting with 10% ethyl acetate in hexanes).

Colorless syrup. [α]_D_^21^ = −45.9 (*c* = 0.5, CHCl_3_). ^**1**^**H NMR** (500 MHz, CDCl_3_) δ 7.56–7.51 (m, 2H, ArH), 7.42–7.36 (m, 2H, ArH), 7.35–7.16 (m, 21H, ArH), 5.57 (d, *J* = 5.0 Hz, 1H, H1), 5.12 (d, *J* = 11.0 Hz, 1H, -CH_2_), 5.02–4.97 (m, 2H, -CH_2_, H6′), 4.94 (d, *J* = 11.4 Hz, 1H, -CH_2_), 4.74–4.66 (m, 2H, CH_2_), 4.65–4.57 (m, 2H, H3, -CH_2_), 4.40 (d, *J* = 11.4 Hz, 1H, CH_2_), 4.31 (dd, *J* = 5.0, 2.4 Hz, 1H, H2), 4.29–4.21 (m, 3H, CH_2_, CH), 4.19 (d, *J* = 7.4 Hz, 1H, H1′), 4.11 (dt, *J* = 7.4, 2.8 Hz, 1H, CH), 4.01 (d, *J* = 2.8 Hz, 1H, H4′), 3.79 (dd, *J* = 9.7, 7.4 Hz, 1H, H2′), 3.73 (dd, *J* = 10.9, 7.7 Hz, 1H, CH), 3.35 (dd, *J* = 9.7, 2.8 Hz, 1H, H3′), 3.08 (d, *J* = 8.8 Hz, 1H, H5), 1.50–1.46 (m, 6H, 2xCH_3_), 1.34 (s, 3H, CH_3_), 1.30 (s, 3H, CH_3_); ^**13**^**C{**^**1**^**H} NMR** (126 MHz, CDCl_3_) δ 139.06 (C), 139.0 (C), 138.7 (C), 137.1 (C), 135.2 (Ar), 131.4 (Ar), 128.8 (Ar), 128.66 (Ar), 128.60 (Ar), 128.4 (Ar), 128.3 (Ar), 128.26 (Ar), 128.20 (Ar), 128.1 (Ar), 128.0 (Ar), 127.8 (Ar), 127.46 (Ar), 127.40 (Ar), 127.38 (Ar), 109.4 (CMe_2_), 108.7 (CMe_2_), 104.7 (C1′), 96.5 (C1), 86.7 (C6′), 82.3 (C3′), 79.1 (C2′), 74.71 (CH_2_), 74.68 (CH_2_), 74.4 (C4′), 73.2 (C5), 71.6 (CH), 70.9 (CH), 70.6 (CH), 70.2 (C6), 70.1 (CH_2_), 67.8 (CH), 26.16 (CH_3_), 26.12 (CH_3_), 25.2 (CH_3_), 24.6 (CH_3_). **HRMS** (ESI) *m*/*z*: [M + Na]^+^ calcd for C_52_H_58_O_11_SNa 913.3592; found 913.3571.

#### Adamantyl (6*R*)-6-Phenylthio-2,3,4,6-tetra-*O*-benzyl-β-d-galactopyranoside (**10**)

Compound **10β** was obtained (24.1 mg, 61%) as a single isomer from the reaction of donor **7a** (40.0 mg, 50.4 μmol) and acceptor (9.2 mg, 60.5 μmol) by following the general procedure **GP5** for glycosylation (eluting with 10% ethyl acetate in hexanes).

Colorless syrup. [α]_D_^21^ = −6.0 (*c* = 0.7, CHCl_3_). ^**1**^**H NMR** (500 MHz, CDCl_3_) δ 7.55–7.48 (m, 2H, ArH), 7.35–7.17 (m, 23H, ArH), 5.08–5.02 (m, 2H, H6, -CH_2_), 4.98 (d, *J* = 11.4 Hz, 1H, -CH_2_), 4.94 (d, *J* = 11.0 Hz, 1H, -CH_2_), 4.74–4.69 (m, 2H, CH_2_), 4.66 (d, *J* = 11.8 Hz, 1H, -CH_2_), 4.56 (d, *J* = 7.7 Hz, 1H, H1), 4.44 (d, *J* = 11.4 Hz, 1H, -CH_2_), 4.19 (d, *J* = 10.9 Hz, 1H, -CH_2_), 4.07 (dd, *J* = 2.8, 1.1 Hz, 1H, H4), 3.79 (dd, *J* = 9.7, 7.7 Hz, 1H, H2), 3.47–3.31 (m, 2H, H3, H5), 2.13 (dq, *J* = 6.4, 3.5, 3.0 Hz, 3H, Ada), 1.95–1.88 (m, 3H, Ada), 1.88–1.79 (m, 3H, Ada), 1.67–1.55 (m, 6H, Ada). ^**13**^**C{**^**1**^**H} NMR** (126 MHz, CDCl_3_) δ 139.1 (C), 138.9 (C), 138.7 (C), 137.1 (C), 134.0 (Ar), 132.7 (Ar), 128.7 (Ar), 128.5 (Ar), 128.35 (Ar), 128.30 (Ar), 128.27 (Ar), 128.21 (Ar), 128.1 (Ar), 127.9 (Ar), 127.6 (Ar), 127.5 (Ar), 127.48 (Ar), 127.45 (Ar), 127.29 (Ar), 127.23 (Ar), 96.67 (C1), 87.87 (C6), 82.93 (C3), 79.63 (C2), 75.27 (CH_2_), 75.23 (CH_2_), 74.73 (C4), 74.69 (CH_2_), 74.09 (C5), 73.20 (CH_2_), 70.37 (CH_2_), 42.79 (Ada), 42.36 (Ada), 36.43 (Ada), 30.82 (Ada), 30.74 (Ada). **HRMS** (ESI) *m*/*z*: [M + Na]^+^ calcd for C_50_H_54_O_6_SNa 805.3533; found 805.3537.

#### (6*R*)-6-Phenylthio-2,3,4,6-tetra-*O*-benzyl-α-d-galactopyranosyl-(1→3)-1,2:5,6-di-*O*-isopropylidene-α-d-glucofuranose (**11**)

Compounds **11α** and **11β** were obtained as a mixture of anomers (38.2 mg, 85%, α/β = 1:4.3) from the reaction of donor **7a** (40.0 mg, 50.4 μmol) and acceptor (15.7 mg, 60.5 μmol) by following the general procedure **GP5** for glycosylation (eluting with 10% ethyl acetate in hexanes). Repeated column chromatography gave pure samples of α- and β-isomers for full characterization.

#### α-Isomer

Colorless syrup. [α]_D_^20^ = +12.5 (*c* = 0.5, CHCl_3_). ^**1**^**H NMR** (500 MHz, CDCl_3_) δ 7.57–7.52 (m, 2H. ArH), 7.99–7.06 (m, 23H, ArH), 5.86 (d, *J* = 3.3 Hz, 1H, H1), 5.25 (d, *J* = 3.7 Hz, 1H, H1′), 5.03 (d, *J* = 9.11H, H6′), 4.99 (d, *J* = 11.0 Hz, 2H, -CH_2_), 4.94 (d, *J* = 3.6 Hz, 1H, H2), 4.83–4.74 (m, 2H, CH_2_), 4.73–4.66 (m, 2H, CH_2_), 4.50–4.42 (m, 1H, CH), 4.38 (d, *J* = 11.3 Hz, 1H, -CH_2_), 4.24 (d, *J* = 2.4 Hz, 1H, H4′), 4.20–4.14 (m, 4H, CH, CH_2_), 4.13–4.05 (m, 1H, H2′), 4.02–3.94 (m, 3H, CH, CH_2_), 3.89–3.83 (m, 1H, H3′), 1.47 (s, 3H, CH3), 1.40 (s, 3H, CH3), 1.24 (s, 3H, CH3), 1.22 (s, 3H, CH_3_). ^**13**^**C{**^**1**^**H} NMR** (126 MHz, CDCl_3_) δ 139.0 (C), 138.7 (C), 138.6 (C), 137.0 (C), 135.1 (C), 132.5 (Ar), 129.1 (Ar), 128.5 (Ar), 128.4 (Ar), 128.38 (Ar), 128.28 (Ar), 128.22 (Ar), 128.1 (Ar), 127.6 (Ar), 127.59 (Ar), 127.57 (Ar), 127.46 (Ar), 127.4 (Ar), 127.3 (Ar), 111.8 (CMe_2_), 108.9(CMe_2_), 105.3 (C1), 99.3 (C1′), 90.1 (C6′), 83.3 (C2), 82.1 (C4), 81.3 (CH), 78.7 (CH), 76.5 (CH), 75.6 (CH), 74.7 (CH_2_), 73.5 (CH_2_), 73.14 (CH_2_), 73.1 (CH), 72.6 (CH), 70.7 (CH_2_), 66.9 (CH_2_), 26.94 (CH_3_), 26.9 (CH_3_), 26.4 (CH_3_), 25.4 (CH_3_). **HRMS** (ESI) *m*/*z*: [M + Na]^+^ calcd for C_52_H_58_O_11_SNa 913.3592; found 913.3566.

#### β-Isomer

Colorless syrup. [α]_D_^21^ = −22.9 (*c* = 0.4, CHCl_3_). ^**1**^**H NMR** (500 MHz, CDCl_3_) δ 7.54–7.49 (m, 2H, ArH), 7.35–7.21 (m, 23H, ArH), 5.79 (d, *J* = 3.8 Hz, 1H, H1), 5.12 (d, *J* = 11.0 Hz, 1H, -CH_2_), 5.01 (d, *J* = 8.8 Hz, 1H, H6′), 4.96 (d, *J* = 11.5 Hz, 1H, -CH_2_), 4.73 (d, *J* = 11.0 Hz, 1H, -CH_2_), 4.67–4.62 (m, 3H, CH_2_), 4.51–4.45 (m, 3H, H2, -CH_2_), 4.42–4.38 (m, 2H, CH, CH_2_), 4.25 (d, *J* = 11.0 Hz, 1H, -CH_2_), 4.19 (d, *J* = 7.7 Hz, 1H, H1′), 4.11–4.05 (m, 2H, CH_2_), 4.03 (dd, *J* = 3.0, 1.1 Hz, 1H, H4′), 3.69 (dd, *J* = 9.7, 7.7 Hz, 1H, H2′), 3.37 (dd, *J* = 9.7, 2.9 Hz, 1H, H3′), 3.11 (dd, *J* = 8.9, 1.1 Hz, 1H, H5′), 1.51 (s, 3H, CH_3_), 1.45 (s, 3H, CH_3_), 1.37 (s, 3H, CH_3_), 1.25 (s, 3H, CH_3_). ^**13**^**C{**^**1**^**H} NMR** (126 MHz, CDCl_3_) δ 138.9 (C), 138.39 (C), 138.3 (C), 137.0 (C), 134.5 (Ar), 132.1 (Ar), 129.0 (Ar), 128.6 (Ar), 128.51 (Ar), 128.50 (Ar), 128.48 (Ar), 128.45 (Ar), 128.3 (Ar), 128.18 (Ar), 128.17 (Ar), 127.84 (Ar), 127.8 (Ar), 127.7 (Ar), 127.5 (Ar), 127.3 (Ar), 111.8 (CMe_2_), 108.3(CMe_2_), 105.2 (C1), 101.4 (C1′), 86.9 (C6′), 82.6 (C3′), 82.3 (CH), 80.4 (CH), 80.2 (CH), 79.4 (C2′), 75.4 (CH_2_), 74.7 (CH_2_), 74.3 (C4′), 74.0 (CH), 73.9 (C5′), 73.0 (CH_2_), 70.4 (CH_2_), 65.4 (CH_2_), 26.9 (CH_3_), 26.6 (CH_3_), 26.3 (CH_3_), 25.5 (CH_3_). **HRMS** (ESI) *m*/*z*: [M + Na]^+^ calcd for C_52_H_58_O_11_SNa 913.3592; found 913.3564.

#### Methyl (6*R*)-6-Phenylthio-2,3,4,6-tetra-*O*-benzyl-β-d-galactopyranosyl-(1→4)-2,3-*O*-isopropylidene-α-l-rhamnopyranoside (**12**)

Compound **12β** was obtained (31.3 mg, 77%) as a single isomer from the reaction of donor **7a** (38.0 mg, 47.9 μmol) and acceptor (12.5 mg, 57.5 μmol) by following the general procedure **GP5** for glycosylation (eluting with 10% ethyl acetate in hexanes).

Colorless syrup. [α]_D_^21^ = −26.6 (*c* = 1.0, CHCl_3_). ^**1**^**H NMR** (500 MHz^,^ CDCl_3_) δ 7.56–7.49 (m, 2H, ArH), 7.39 (dd, *J* = 7.9, 1.7 Hz, 2H, ArH), 7.34–7.17 (m, 21H, ArH), 5.09–5.03 (m, 2H, -CH_2_, H6′), 4.99 (d, *J* = 11.5 Hz, 1H, -CH_2_), 4.92 (d, *J* = 10.9 Hz, 1H, -CH_2_), 4.86 (s, 1H, H1), 4.78 (d, *J* = 7.7 Hz, 1H, H1′), 4.74–4.69 (m, 2H, -CH_2_), 4.66 (d, *J* = 11.8 Hz, 1H, -CH_2_), 4.42 (d, *J* = 11.5 Hz, 1H, -CH_2_), 4.26 (t, *J* = 6.0 Hz, 1H, CH), 4.20 (d, *J* = 11.0 Hz, 1H, CH_2_), 4.11 (dd, *J* = 5.9, 0.8 Hz, 1H, CH), 4.04 (dd, *J* = 3.1, 1.1 Hz, 1H, H4′), 3.77 (dd, *J* = 9.7, 7.7 Hz, 1H, H2′), 3.67 (m, 2H, H5), 3.49 (dd, *J* = 9.7, 2.9 Hz, 1H, H3′), 3.39 (s, 3H, OMe), 3.38–3.31 (m, 1H, CH), 1.54 (s, 3H, CH_3_), 1.40 (d, *J* = 5.7 Hz, 3H, CH_3_), 1.34 (s, 3H, CH_3_). ^**13**^**C{**^**1**^**H} NMR** (126 MHz, CDCl_3_) δ 139.2 (C), 138.9 (C), 138.7 (C), 137.1 (C), 133.6 (Ar), 133.5 (Ar), 133.2 (Ar), 128.9 (Ar), 128.5 (Ar), 128.3 (Ar), 128.29 (Ar), 128.25 (Ar), 128.0 (Ar), 127.6 (Ar), 127.57 (Ar), 127.53 (Ar), 127.52 (Ar), 127.47 (Ar), 127.42 (Ar), 127.37 (Ar), 127.34 (Ar), 109.3 (CMe2), 102.9 (C1′), 98.2 (C1), 88.0 (C6′), 82.6 (C3′), 82.4 (CH), 80.2 (CH), 79.8 (CH), 78.4 (CH), 75.9 (CH), 75.2 (CH_2_), 74.8 (C4′), 74.6 (CH_2_), 74.0 (CH), 73.2 (CH_2_), 70.3 (CH_2_), 64.5 (CH_2_), 54.9 (OCH_3_), 28.2 (CH_3_), 26.2 (CH_3_), 18.1 (CH_3_). **HRMS** (ESI) *m*/*z*: [M + Na]^+^ calcd for C_50_H_56_O_10_SNa 871.3486; found 871.3470.

#### Methyl (6*R*)-6-Phenylthio-2,3,4,6-tetra-*O*-benzyl-α/β-d-galactopyranosyl-(1→4)-2,3,4-tri-*O*-benzyl-α-d-glucopyranoside (**13**)

Compounds **13α** and **13β** were obtained as a mixture of anomers (47.9 mg, 76%, α/β = 1:1.1) from the reaction of donor **7a** (50.0 mg, 63.0 μmol) and acceptor (35.1 mg, 76.6 μmol) by following the general procedure **GP5** for glycosylation (eluting with 10% ethyl acetate in hexanes, 1:9). Repeated column chromatography gave pure samples of α- and β-isomers for full characterization.

#### α-Isomer

Colorless syrup. [α]_D_^21^ = +18.0 (*c* = 1.0, CHCl_3_). ^**1**^**H NMR** (500 MHz, CDCl_3_) δ 7.59–7.53 (m, 2H, ArH), 7.35–7.15 (m, 38H, ArH), 5.68 (d, *J* = 3.7 Hz, 1H, H1′), 5.07–4.99 (m, 2H, H6′, CH_2_), 4.96–4.89 (m, 2H, CH_2_), 4.82 (d, *J* = 11.4 Hz, 1H, CH_2_), 4.67 (d, *J* = 12.1 Hz, 1H, CH_2_), 4.65–4.61 (m, 2H, CH_2_), 4.61–4.55 (m, 3H, H1, CH_2_), 4.55–4.49 (m, 2H, CH_2_), 4.45 (d, *J* = 12.1 Hz, 1H, CH_2_), 4.37 (d, *J* = 11.2 Hz, 1H, CH_2_), 4.17 (d, *J* = 10.9 Hz, 1H, CH_2_), 4.07 (dd, *J* = 2.8, 1.4 Hz, 1H, H4′), 4.04–3.94 (m, 3H, H2′,CH), 3.90–3.81 (m, 2H, H5, CH), 3.76–3.68 (m, 2H, CH, H6b), 3.66 (dd, *J* = 10.4, 2.7 Hz, 1H, H6a), 3.55 (dd, *J* = 9.2, 3.6 Hz, 1H, CH), 3.38 (s, 3H, OCH_3_). ^**13**^**C{**^**1**^**H} NMR** (126 MHz, CDCl_3_) δ 139.2 (C), 139.0 (C), 138.8 (C), 138.4 (C), 138.2 (C), 138.2 (C), 137.4 (C), 135.6 (C), 132.7 (Ar), 129.0 (Ar), 128.5 (Ar), 128.4 (Ar), 128.37 (Ar), 128.35 (Ar), 128.32 (Ar), 128.31 (Ar), 128.29 (Ar), 128.27 (Ar), 127.99 (Ar), 127.96 (Ar), 127.94 (Ar), 127.7 (Ar), 127.59 (Ar), 127.53 (Ar), 127.4 (Ar), 127.3 (Ar), 127.2 (Ar), 127.1 (Ar), 127.0 (Ar), 97.8 (C1), 97.1 (C1′), 90.4 (C6′), 82.0 (CH), 79.9 (CH), 79.1 (CH), 76.0 (CH), 75.7 (C4′), 74.9 (CH_2_), 74.3 (CH_2_), 73.9 (CH), 73.5 (CH_2_), 73.4 (CH_2_), 73.1 (CH_2_), 72.6 (CH), 70.6 (CH_2_), 70.1 (C6), 69.6 (CH), 55.2 (OCH_3_). **HRMS** (ESI) *m*/*z*: [M + Na]^+^ calcd for C_68_H_70_O_11_SNa 1117.4531; found 1117.4503.

#### β-Isomer

Colorless syrup. [α]_D_^21^ = −22.0 (*c* = 1.0, CHCl_3_). ^**1**^**H NMR** (500 MHz, CDCl_3_) δ 7.59–7.55 (m, 2H, ArH), 7.47–7.41 (m, 2H, ArH), 7.34–7.16 (m, 30H, ArH), 7.16–7.06 (m, 4H, ArH), 7.05–6.98 (m, 2H, ArH), 5.17 (d, *J* = 11.0 Hz, 1H, -CH_2_), 5.03 (d, *J* = 11.0 Hz, 1H, -CH_2_), 4.99 (d, *J* = 11.3 Hz, 1H, -CH_2_), 4.84 (d, *J* = 12.2 Hz, 1H, -CH_2_), 4.80 (d, *J* = 8.8 Hz, 1H, H6′), 4.79–4.70 (m, 3H, -CH_2_), 4.69–4.64 (m, 3H, CH_2_), 4.57 (m, 2H, H1, -CH_2_), 4.38 (d, *J* = 11.3 Hz, 1H, -CH_2_), 4.33 (d, *J* = 12.2 Hz, 1H, -CH_2_), 4.21–4.14 (m, 2H, CH_2_), 4.07 (dd, *J* = 10.1, 9.0 Hz, 1H, CH), 4.04 (dd, *J* = 2.9, 1.1 Hz, 1H, H4′), 3.87 (t, *J* = 9.4 Hz, 1H, CH), 3.80–3.72 (m, 2H, H2′), 3.61 (dt, *J* = 10.0, 2.7 Hz, 1H, CH), 3.49 (dd, *J* = 9.7, 3.7 Hz, 1H, CH), 3.44 (dd, *J* = 10.8, 2.1 Hz, 1H, CH), 3.37 (s, 3H, OCH_3_), 3.26–3.14 (m, 2H, H3′, H5′). ^**13**^**C{**^**1**^**H} NMR** (126 MHz, CDCl_3_) δ 139.8 (C), 139.3 (C), 139.0 (C), 138.7 (C), 138.6 (C), 138.0 (C), 137.3 (C), 134.5 (Ar), 133.6 (Ar), 128.78 (Ar), 128.7 (Ar), 128.6 (Ar), 128.48, (Ar) 128.41 (Ar), 128.38 (Ar), 128.30 (Ar), 128.27 (Ar), 128.24 (Ar), 128.2 (Ar), 127.97 (Ar), 127.94 (Ar), 127.93 (Ar), 127.74 (Ar), 127.7 (Ar), 127.6 (Ar), 127.5 (Ar), 127.4 (Ar), 127.34 (Ar), 127.30 (Ar), 127.2 (Ar), 127.0 (Ar), 102.3 (C1′), 98.8 (C1), 88.8 (C6′), 82.6 (C3′), 80.6 (CH), 80.2 (CH), 78.5 (CH), 75.9 (CH), 75.7 (CH_2_), 75.2 (CH_2_), 74.7 (CH_2_), 74.5 (C4′), 74.4 (C5′), 73.8 (CH_2_), 73.5 (CH_2_), 72.8 (CH_2_), 70.26 (CH_2_), 70.2 (CH), 67.9 (CH_2_), 55.4 (OCH_3_). **HRMS** (ESI) *m*/*z*: [M + Na]^+^ calcd for C_68_H_70_O_11_SNa 1117.4531; found 1117.4529.

#### Methyl (6*S*)-6-Phenylthio-2,3,4,6-tetra-*O*-benzyl-α/β-d-galactopyranosyl-(1→6)-2,3,4-tri-*O*-benzyl-α-d-glucopyranoside (**14**)

Compounds **14α** and **14β** were obtained as a mixture of anomers (78.3 mg, 81%, α/β = 1:11.9) from the reaction of donor **7b** (70.0 mg, 88.2 μmol) and acceptor (49.2 mg, 105.9 μmol) by following the general procedure **GP5** for glycosylation (eluting with 10% ethyl acetate in hexanes). Repeated column chromatography gave pure samples of the α- and β-isomers for full characterization.

#### α-Isomer

Colorless syrup. [α]_D_^20^ = +23.2 (*c* = 2.0, CHCl_3_). ^**1**^**H NMR** (900 MHz, CDCl_3_) δ 7.42–7.12 (m, 40H, ArH), 5.14 (m, 2H, -CH_2_, H6′), 5.05 (d, *J* = 3.7 Hz, 1H, H1′), 4.95 (d, *J* = 11.0 Hz, 1H, -CH_2_), 4.85 (t, *J* = 11.2 Hz, 2H, -CH_2_), 4.82–4.67 (m, 6H, -CH_2_, -CH), 4.64–4.54 (m, 3H, -CH_2_), 4.49 (d, *J* = 11.1 Hz, 1H, -CH_2_), 4.45 (d, *J* = 3.5 Hz, 1H, H1′), 4.36 (s, 1H, H4′), 4.07 (d, *J* = 9.9 Hz, 1H, -CH), 3.94 (t, *J* = 9.5 Hz, 1H, -CH), 3.91 (d, *J* = 8.9 Hz, 1H, -CH), 3.84–3.81 (m, 1H, -CH), 3.71 (s, 3H), 3.50 (s, 1H, -CH), 3.38 (dd, *J* = 9.2, 4.0 Hz, 1H, -CH), 3.23 (s, 3H, -OCH_3_). ^**13**^**C{**^**1**^**H} NMR** (226 MHz, CDCl_3_) δ 138.9 (C), 138.8 (C), 138.4 (C), 138.2 (C), 137.9 (C), 132.9 (Ar), 128.9 (Ar), 128.4 (Ar), 128.36 (Ar), 128.34 (Ar), 128.3 (Ar), 128.19 (Ar), 128.13 (Ar), 128.0 (Ar), 127.97 (Ar), 127.94 (Ar), 127.9 (Ar), 127.8 (Ar), 127.6 (Ar), 127.58 (Ar), 127.53 (Ar), 127.50 (Ar), 127.37 (Ar), 127.34 (Ar), 97.7 (C1′), 97.6 (C1), 87.5 (C6′), 82.0 (CH), 80.1 (CH), 78.6 (CH), 78.1 (CH), 77.2 (CH), 76.36 (CH), 76.34 (CH), 75.7, 75.0, 74.9, 73.4 (CH_2_), 73.0 (CH_2_), 72.5 (CH_2_), 71.8 (CH_2_), 70.4 (CH_2_), 70.1 (CH_2_), 66.0 (CH_2_), 54.9 (OCH_3_). **HRMS** (ESI) *m*/*z*: [M + Na]^+^ calcd for C_68_H_70_O_11_SNa 1117.4531; found 1117.4532.

#### β-Isomer

Colorless syrup. [α]_D_^21^ = +27.4 (*c* = 1.0, CHCl_3_). ^**1**^**H NMR** (500 MHz, CDCl_3_) δ 7.39–7.15 (m, 38H, ArH), 7.11–7.06 (m, 2H, ArH), 5.14–5.06 (m, 2H, -CH_2_, H6′), 4.96–4.89 (m, 2H, -CH_2_), 4.84 (d, *J* = 11.6 Hz, 1H, -CH_2_), 4.80–4.69 (m, 5H, -CH_2_, -CH_2_), 4.68–4.55 (m, 5H, -CH_2_, H1), 4.45 (d, *J* = 11.1 Hz, 1H, -CH_2_), 4.29 (d, *J* = 2.8 Hz, 1H, H4′), 4.21–4.12 (m, 2H, H1′, H6b), 3.94 (t, *J* = 9.3 Hz, 1H, -CH), 3.86 (dd, *J* = 9.7, 7.7 Hz, 1H, H2′), 3.77 (ddd, *J* = 10.1, 4.5, 2.1 Hz, 1H, H5), 3.61 (dd, *J* = 11.0, 4.5 Hz, 1H, H6a), 3.54–3.44 (m, 2H, -CH, H2), 3.35 (dd, *J* = 9.7, 2.8 Hz, 1H, H3′), 3.33–3.27 (m, 4H, H5′,OCH_3_). ^**13**^**C{**^**1**^**H} NMR** (126 MHz, CDCl_3_) δ 139.0 (C), 138.9 (C), 138.8 (C), 138.52 (C), 138.5 (C), 138.3 (C), 137.5 (C), 133.7 (Ar), 131.9 (Ar), 129.0 (Ar), 128.53 (Ar), 128.50 (Ar), 128.41 (Ar), 128.39 (Ar), 128.34 (Ar), 128.31 (Ar), 128.23 (Ar), 128.19 (Ar), 128.03 (Ar), 128.01 (Ar), 127.99 (Ar), 127.97 (Ar), 127.96 (Ar), 127.8 (Ar), 127.78 (Ar), 127.74 (Ar), 127.71 (Ar), 127.57 (Ar), 127.52 (Ar), 127.44 (Ar), 127.41 (Ar), 104.2 (C1′), 98.1 (C1), 86.6 (C6′), 82.5 (C3′), 82.1 (-CH), 79.9 (CH), 79.2 (C2′), 77.9 (CH), 75.9 (C5′), 75.7 (CH_2_), 75.2 (C4′), 75.1 (CH_2_), 74.9 (CH_2_), 74.8 (-CH_2_), 73.4 (CH_2_), 73.3 (CH_2_), 71.0 (CH_2_), 69.9 (C5), 68.5 (C6), 55.2 (OCH_3_). **HRMS** (ESI) *m*/*z*: [M + Na]^+^ calcd for C_68_H_70_O_11_SNa 1117.4531; found 1117.4541.

#### (6*S*)-6-Phenylthio-2,3,4,6-tetra-*O*-benzyl-α/β-d-galactopyranosyl-(1→6)-1,2:3,4-*O*-diisopropylidene-α-d-galactopyranose (**15**)

Compounds **15α** and **15β** were obtained as a mixture of anomers (48.1 mg, 69%, α/β = 1:11.5) from the reaction of donor **7b** (62.0 mg, 78.2 μmol) and acceptor (24.4 mg, 93.8 μmol) by following the general procedure **GP5** for glycosylation (eluting with 10% ethyl acetate in hexanes, 1:9). The α-isomer was not obtained pure and was characterized in the mixture of anomers by the following diagnostic signals.

#### α-Isomer

^1^H NMR (500 MHz, CDCl_3_) δ 7.42–7.39 (m, 2H, ArH), 7.38–7.31 (m, 6H, ArH), 7.30–7.19 (m, 17H, ArH), 5.48 (d, *J* = 5.0 Hz, 1H, H1), 5.15–5.10 (m, 2H, H6′, -CH_2_), 5.07 (d, *J* = 10.8 Hz, 1H, -CH_2_), 4.88–4.82 (m, 2H, -CH_2_), 4.78–4.67 (m, 3H, -CH_2_), 4.63–4.57 (m, 2H, -CH_2_), 4.51 (dd, *J* = 7.9, 2.4 Hz, 1H, -CH), 4.39–4.35 (m, 1H, -CH), 4.27 (dd, *J* = 5.0, 2.4 Hz, 1H, H2), 4.14 (dd, *J* = 7.9, 1.9 Hz, 1H, -CH), 4.07 (dd, *J* = 10.0, 3.7 Hz, 1H, H6b), 3.99 (td, *J* = 6.9, 1.9 Hz, 1H, H5), 3.94–3.90 (m, 1H, -CH), 3.85 (dd, *J* = 10.1, 2.8 Hz, 1H, H2′), 3.77 (dd, *J* = 10.8, 7.1 Hz, 1H, H6a), 3.67 (dd, *J* = 10.8, 6.6 Hz, 1H, -CH), 1.51 (s, 3H, CH_3_), 1.37 (s, 3H, CH_3_), 1.31 (s, 3H, CH_3_), 1.25 (s, 3H, CH_3_).

Repeated column chromatography gave a pure sample of the β-isomer for full characterization.

#### β-Isomer

Colorless syrup. [α]_D_^21^ = +23.5 (*c* = 0.9, CHCl_3_). ^**1**^**H NMR** (500 MHz, CDCl_3_) δ 7.48–7.43 (m, 2H, ArH), 7.41–7.18 (m, 23H, ArH), 5.56 (d, *J* = 5.0 Hz, 1H, H1), 5.15–5.10 (m, 2H, -CH_2_, H6′), 5.05 (d, *J* = 11.2 Hz, 1H, -CH_2_), 4.91 (d, *J* = 11.4 Hz, 1H, -CH_2_), 4.86 (d, *J* = 12.1 Hz, 1H, -CH_2_), 4.75 (m, 2H, -CH_2_), 4.65 (m, 2H, -CH_2_), 4.51 (dd, *J* = 7.9, 2.4 Hz, 1H, H3), 4.34 (d, *J* = 7.7 Hz, 1H, H1′), 4.32 (d, *J* = 2.9 Hz, 1H, H4′), 4.29 (dd, *J* = 5.0, 2.4 Hz, 1H, H2), 4.18 (dd, *J* = 7.9, 1.9 Hz, 1H, -CH), 4.14 (dd, *J* = 10.8, 3.6 Hz, 1H, H6b), 4.09 (ddd, *J* = 7.4, 3.5, 1.8 Hz, 1H, H5), 3.86 (dd, *J* = 9.8, 7.6 Hz, 1H, H2′), 3.73 (dd, *J* = 10.8, 7.4 Hz, 1H, H6a), 3.40–3.34 (m, 2H, H3′, H5′), 1.50 (s, 3H, CH_3_), 1.41 (s, 3H, CH_3_), 1.32 (s, 3H, CH_3_), 1.29 (s, 3H, CH_3_). ^**13**^**C{**^**1**^**H} NMR** (126 MHz, CDCl_3_) δ 139.1 (C), 138.9 (C), 138.8 (C), 137.6 (C), 133.8 (Ar), 131.9 (Ar), 129.1 (Ar), 128.7 (Ar), 128.49 (Ar), 128.46 (Ar), 128.3 (Ar), 128.23 (Ar), 128.21 (Ar), 128.1 (Ar), 128.0 (Ar), 127.8 (Ar), 127.7 (Ar), 127.48 (Ar), 127.44 (Ar), 109.3 (CMe_2_), 108.7 (CMe_2_), 104.9 (C1′), 96.5 (C1), 86.9 (C6′), 82.0 (C3′), 78.9 (C2′), 75.8 (C5′), 75.3 (C4′), 75.0 (CH_2_), 74.6 (CH_2_), 73.5 (CH_2_), 71.5 (CH_2_), 71.1 (CH_2_), 70.8 (CH_2_), 70.6 (C2), 69.9 (C6), 67.7 (C5), 26.2 (CH_3_), 26.1 (CH_3_), 25.2 (CH_3_), 24.5 (CH_3_). **HRMS** (ESI) *m*/*z*: [M + Na]^+^ calcd for C_52_H_58_O_11_SNa 913.3592; found 913.3575.

#### Adamantyl (6*S*)-6-Phenylthio-2,3,4,6-tetra-*O*-benzyl-α-d-galactopyranoside (**16**)

Compounds **16α** and **16β** were obtained as a mixture of anomers (48.3 mg, 72%, α/β = 1:4.1) from the reaction of donor **7b** (68.0 mg, 85.7 μmol) and adamantanol (15.6 mg, 102.9 μmol) by following the general procedure **GP5** for glycosylation (eluting with 10% ethyl acetate in hexanes). Repeated column chromatography gave pure samples of α- and β-isomers for full characterization.

#### α-Isomer

Colorless syrup. [α]_D_^20^ = −6.6 (*c* = 0.6, CHCl_3_). ^**1**^**H NMR** (500 MHz, CDCl_3_) δ 7.46–7.42 (m, 2H, ArH), 7.41–7.38 (m, 2H, ArH), 7.38–7.33 (m, 4H, ArH), 7.32–7.19 (m, 17H, ArH), 5.33 (d, *J* = 3.8 Hz, 1H, H1), 5.13 (d, *J* = 10.9 Hz, 1H, CH_2_), 5.06 (d, *J* = 9.1 Hz, 1H, H6), 4.95 (d, *J* = 11.2 Hz, 1H, CH_2_), 4.86 (d, *J* = 11.7 Hz, 1H, CH_2_), 4.76 (d, *J* = 11.7 Hz, 1H, CH_2_), 4.72 (d, *J* = 12.0 Hz, 1H, CH_2_), 4.68 (d, *J* = 12.0 Hz, 1H, CH_2_), 4.61 (d, *J* = 10.9 Hz, 1H, CH_2_), 4.48 (d, *J* = 11.2 Hz, 1H, CH_2_), 4.42 (d, *J* = 2.8 Hz, 1H, H4), 4.08–4.00 (m, 2H, H2, H5), 3.85 (dd, *J* = 10.1, 2.8 Hz, 1H, H3), 2.00 (p, *J* = 3.3 Hz, 3H, Ada), 1.81–1.74 (m, 3H, Ada), 1.66 (dq, *J* = 11.7, 2.6 Hz, 3H, Ada), 1.59–1.52 (m, 3H, Ada), 1.51–1.45 (m, 3H, Ada). ^**13**^**C{**^**1**^**H} NMR** (126 MHz, CDCl_3_) δ 139.18 (C), 139.14 (C), 138.9 (C), 137.9 (C), 133.5 (Ar), 132.7 (Ar), 128.9 (Ar), 128.4 (Ar), 128.3 (Ar), 128.23 (Ar), 128.18 (Ar), 128.07 (Ar), 128.04 (Ar), 127.7 (Ar), 127.6 (Ar), 127.58 (Ar), 127.55 (Ar), 127.50 (Ar), 127.42 (Ar), 127.39 (Ar), 90.6 (C1), 87.9 (C6), 79.7 (C3), 77.3 (-CH_2_), 76.4 (C2), 76.3 (C4), 74.9 (CH_2_), 74.5 (CH_2_), 73.2 (CH_2_), 72.9 (CH_2_), 71.5 (CH_2_), 71.3 (C5), 42.4 (Ada), 36.3 (Ada), 30.7 (Ada). **HRMS** (ESI) *m*/*z*: [M + Na]^+^ calcd for C_50_H_54_O_6_SNa 805.3533; found 805.3520.

#### β-Isomer

Colorless syrup. [α]_D_^21^ = +21.6 (*c* = 1.0, CHCl_3_). ^**1**^**H NMR** (500 MHz, CDCl_3_) δ 7.40–7.18 (m, 25H, ArH), 5.15–5.08 (m, 2H, H6, CH_2_), 4.95 (d, *J* = 11.1 Hz, 1H, CH_2_), 4.87 (d, *J* = 11.0 Hz, 1H, CH_2_), 4.78 (d, *J* = 12.0 Hz, 1H, CH_2_), 4.74–4.68 (m, 2H, -CH_2_), 4.60 (d, *J* = 10.9 Hz, 1H, -CH_2_), 4.56 (d, *J* = 11.0 Hz, 1H, -CH_2_), 4.51 (d, *J* = 7.7 Hz, 1H, H1), 4.29 (dd, *J* = 2.9, 1.0 Hz, 1H, H4), 3.78 (dd, *J* = 9.8, 7.7 Hz, 1H, H2), 3.37 (dd, *J* = 9.8, 2.9 Hz, 1H, H3), 3.33 (dd, *J* = 8.8, 1.0 Hz, 1H, H5), 2.02 (t, *J* = 3.2 Hz, 3H, Ada), 1.85 (dq, *J* = 11.5, 2.4 Hz, 3H, Ada), 1.75 (dq, *J* = 11.6, 2.7 Hz, 3H, Ada), 1.61–1.47 (m, 6H, Ada). ^**13**^**C{**^**1**^**H} NMR** (126 MHz, CDCl_3_) δ 139.04 (C), 139.0 (C), 138.8 (C), 137.6 (C), 133.5 (Ar), 132.3 (Ar), 129.0 (Ar), 128.44 (Ar), 128.39 (Ar), 128.34 (Ar), 128.30 (Ar), 128.27 (Ar), 128.1 (Ar), 128.0 (Ar), 127.8 (Ar), 127.7 (Ar), 127.65 (Ar), 127.60 (Ar), 127.54 (Ar), 127.51 (Ar), 127.4 (Ar), 96.7 (C1), 87.3 (C6), 82.8 (C3), 79.4 (CH_2_), 75.9 (C5), 75.5 (C4), 75.16 (CH_2_), 75.1 (CH_2_), 74.9 (CH_2_), 73.5 (CH_2_), 71.5 (CH_2_), 42.8 (Ada), 36.3 (Ada), 30.7 (Ada). **HRMS** (ESI) *m*/*z*: [M + Na]^+^ calcd for C_50_H_54_O_6_SNa 805.3533; found 805.3525.

#### (6*S*)-6-Phenylthio-2,3,4,6-tetra-*O*-benzyl-α-d-galactopyranosyl-(1→3)-1,2:5,6-di-*O*-isopropylidene-α-d-glucofuranose (**17**)

Compounds **17α** and **17β** were obtained as a mixture of anomers (57.2 mg, 85%, α/β = 1:6.3) from the reaction of donor **7b** (60.0 mg, 75.6 μmol) and acceptor (23.7 mg, 90.8 μmol) by following the general procedure **GP5** for glycosylation (eluting with 10% ethyl acetate in hexanes). Repeated column chromatography gave pure samples of the α- and β-isomers for full characterization.

#### α-Isomer

Colorless syrup. [α]_D_^23^ = +36.3 (*c* = 1.0, CHCl_3_). ^**1**^**H NMR** (500 MHz, CDCl_3_) δ 7.43–7.20 (m, 26H, ArH), 5.20 (d, *J* = 3.5 Hz, 1H, H1), 5.13 (d, *J* = 10.9 Hz, 1H, -CH_2_), 5.08 (d, *J* = 3.8 Hz, 1H, H1), 5.01 (d, *J* = 11.6 Hz, 1H, -CH_2_), 4.96 (d, *J* = 9.3 Hz, 1H, H6′), 4.87 (d, *J* = 12.0 Hz, 1H, -CH_2_), 4.76–4.64 (m, 4H, -CH_2_), 4.62 (d, *J* = 11.6 Hz, 1H, -CH_2_), 4.53 (d, *J* = 3.5 Hz, 1H, H2), 4.44 (dd, *J* = 2.8, 1.1 Hz, 1H, H4′), 4.36 (td, *J* = 6.5, 5.2 Hz, 1H, -CH), 4.12 (dd, *J* = 6.5, 2.8 Hz, 1H), 4.05–3.97 (m, 3H, -CH_2_, CH), 3.89 (dd, *J* = 8.5, 5.3 Hz, 1H, -CH), 3.63 (dd, *J* = 10.2, 2.8 Hz, 1H, CH_2_), 3.57 (dd, *J* = 9.3, 1.1 Hz, 1H, CH), 1.39 (s, 3H, CH_3_), 1.38 (s, 3H, CH_3_), 1.19 (s, 3H, CH_3_), 0.86 (s, 3H, CH_3_). ^**13**^**C{**^**1**^**H} NMR** (126 MHz, CDCl_3_) δ 138.85 (C), 138.79 (C), 138.75 (C), 137.3 (C), 134.5 (C), 130.9 (Ar), 129.0 (Ar), 128.57 (Ar), 128.54 (Ar), 128.43 (Ar), 128.40 (Ar), 128.3 (Ar), 128.1 (Ar), 128.0 (Ar), 127.80 (Ar), 127.78 (Ar), 127.66 (Ar), 127.63 (Ar), 127.61 (Ar), 127.5 (Ar), 111.5 (CMe2), 108.8 (CMe_2_), 105.0 (C1), 99.7 (C1′), 86.2 (C6′), 82.8 (CH), 82.4 (CH), 80.9 (CH), 78.8 (CH), 76.6 (CH), 76.1 (CH), 75.1 (CH_2_), 73.5 (CH_2_), 73.2 (CH_2_), 72.8 (CH), 71.9 (CH_2_), 71.3 (CH_2_), 66.5 (C6), 26.9 (CH_3_), 26.8 (CH_3_), 25.8 (CH_3_), 25.3 (CH_3_). **HRMS** (ESI) *m*/*z*: [M + Na]^+^ calcd for C_52_H_58_O_11_SNa 913.3592; found 913.3596.

#### β-Isomer

Colorless syrup. [α]_D_^21^ = +26.1 (*c* = 1.0, CHCl_3_). ^**1**^**H NMR** (500 MHz, CDCl_3_) δ 7.38–7.23 (m, 23H, ArH), 7.23–7.18 (m, 2H, ArH), 5.72 (d, *J* = 3.8 Hz, 1H, H1), 5.10 (d, *J* = 11.1 Hz, 1H, CH_2_), 5.05 (d, *J* = 8.8 Hz, H6′), 4.85 (d, *J* = 11.5 Hz, 1H, -CH_2_), 4.79–4.72 (m, 3H, -CH_2_), 4.71–4.64 (m, 2H, -CH_2_), 4.61 (d, *J* = 11.2 Hz, 1H, -CH_2_), 4.45–4.37 (m, 2H, CH, H2), 4.33 (t, *J* = 3.7 Hz, 1H, CH), 4.31–4.25 (m, 3H, -CH, H1′), 4.04–3.97 (m, 2H, H6), 3.70 (dd, *J* = 9.7, 7.7 Hz, 1H, H2′), 3.35 (dd, *J* = 9.7, 2.8 Hz, 1H, H3′), 3.29 (dd, *J* = 8.8, 1.2 Hz, 1H, H5′), 1.44 (s, 3H, CH_3_), 1.36 (s, 3H, CH_3_), 1.26 (s, 3H, CH_3_), 1.18 (s, 3H, CH_3_). ^**13**^**C{**^**1**^**H} NMR** (126 MHz, CDCl_3_) δ 138.9 (C), 138.5 (C), 138.3 (C), 137.3 (C), 133.8 (Ar), 131.6 (Ar), 129.1 (Ar), 128.5 (Ar), 128.4 (Ar), 128.3 (Ar), 128.27 (Ar), 128.1 (Ar), 127.88 (Ar), 127.83 (Ar), 127.77 (Ar), 127.72 (Ar), 127.6 (Ar), 127.4 (Ar), 111.7 (CMe_2_), 108.4 (CMe2), 105.2 (C1), 101.7 (C1′), 86.2 (C6′), 82.8 (CH), 82.3 (C3′), 80.40 (CH), 80.3 (CH), 79.3 (C2′), 76.3 (C5′), 75.2(CH_2_), 75.1 (CH_2_), 74.8 (CH), 73.7 (CH), 73.3 (CH_2_), 71.3 (CH_2_), 65.7 (C6), 26.8 (CH_3_), 26.6 (CH_3_), 26.2 (CH_3_), 25.4 (CH_3_). **HRMS** (ESI) *m*/*z*: [M + Na]^+^ calcd for C_52_H_58_O_11_SNa 913.3592; found 913.3589.

#### Methyl (6*S*)-6-Phenylthio-2,3,4,6-tetra-*O*-benzyl-α/β-d-galactopyranosyl-(1→4)-2,3-*O*-isopropylidene-α-l-rhamnopyranoside (**18**)

Compounds **18α** and **18β** were obtained as a mixture of anomers (54.0 mg, 84%, α/β = 1:1.02) from the reaction of donor **7b** (60.0 mg, 75.6 μmol) and acceptor (19.8 mg, 90.8 μmol) by following the general procedure **GP5** for glycosylation (eluting with 10% ethyl acetate in hexanes). Repeated column chromatography gave pure samples of α- and β-isomers for full characterization.

#### α-Isomer

Colorless syrup. [α]_D_^21^ = +31.0 (*c* = 1.0, CHCl_3_). ^**1**^**H NMR** (500 MHz, CDCl_3_) δ 7.48–7.44 (m, 2H, ArH), 7.38–7.18 (m, 23H, ArH), 5.23 (d, *J* = 3.7 Hz, 1H, H_1_′), 5.15 (d, *J* = 7.4 Hz, 1H, H_6_′), 5.03 (d, *J* = 10.8 Hz, 1H, CH_2_), 4.84 (d, *J* = 11.5 Hz, 1H, CH_2_), 4.81–4.77 (m, 3H, CH_2_, H_1_), 4.74 (d, *J* = 11.8 Hz, 1H, CH_2_), 4.67 (m, 2H), 4.50 (d, *J* = 11.5 Hz, 1H, CH_2_), 4.37 (dd, *J* = 2.8, 1.3 Hz, 1H, H4′), 4.22–4.16 (m, 2H, H5′, H3), 4.13 (dd, *J* = 10.2, 3.6 Hz, 1H, H2′), 3.95 (d, *J* = 5.8 Hz, 1H, H2), 3.86 (dd, *J* = 10.2, 2.7 Hz, 1H, H3′), 3.62 (dq, *J* = 9.7, 6.3 Hz, 1H, H5), 3.37 (dd, *J* = 9.8, 7.1 Hz, 1H, H4), 3.33 (s, 3H, OCH_3_), 1.33 (s, 3H, CH_3_), 1.29 (d, *J* = 6.3 Hz, 3H, CH_3_), 1.13 (s, 3H, CH_3_). ^**13**^**C{**^**1**^**H} NMR** (126 MHz, CDCl_3_) δ 139.1 (C), 138.8 (C), 138.7 (C), 137.8 (C), 132.8 (Ar), 128.9 (Ar), 128.4 (Ar), 128.39 (Ar), 128.33 (Ar), 128.09 (Ar), 128.07 (Ar), 128.01 (Ar), 127.7 (Ar), 127.6 (Ar), 127.5 (Ar), 127.4 (Ar), 127.3 (Ar), 127.2 (Ar), 109.0 (CMe_2_), 98.3 (C1′), 98.1 (C1), 89.7 (C6′), 81.4 (C4), 79.3 (C3′), 77.0 (C3), 76.5 (C2′), 75.7 (C2), 75.4 (C4′), 74.6 (CH_2_), 73.8 (CH_2_), 72.9 (CH_2_), 71.5 (C5′), 71.2, 64.6 (C5), 54.8 (OCH_3_), 28.2 (CH_3_), 26.3 (CH_3_), 17.9 (CH_3_). **HRMS** (ESI) *m*/*z*: [M + Na]^+^ calcd for C_50_H_56_O_10_SNa 871.3486; found 871.3478.

#### β-Isomer

Colorless syrup. [α]_D_^21^ = +18.6 (*c* = 1.0, CHCl_3_). ^**1**^**H NMR** (500 MHz, CDCl_3_) δ 7.39–7.21 (m, 23H, ArH), 7.20–7.15 (m, 2H, ArH), 5.13–5.08 (m, 2H, H6′, CH_2_), 4.93–4.83 (m, 3H, H1, CH_2_), 4.80 (d, *J* = 12.1 Hz, 1H, CH_2_), 4.75 (dd, *J* = 7.8, 1.1 Hz, 1H, H1′), 4.73–4.67 (m, 3H, CH_2_), 4.61 (d, *J* = 11.1 Hz, 1H, CH_2_), 4.25 (d, *J* = 3.0 Hz, 1H, H4′), 4.17 (t, *J* = 6.4 Hz, 1H, H3), 4.04 (d, *J* = 5.7 Hz, 1H, H2), 3.78–3.70 (m, 2H, H2′, H4), 3.62 (m, 1H, H5), 3.42–3.36 (m, 4H, H3′, OCH_3_), 3.31–3.26 (m, 1H, H5′), 1.33 (d, *J* = 6.1 Hz, 3H, CH_3_), 1.29–1.22 (m, 6H, 2xCH_3_). ^**13**^**C{**^**1**^**H} NMR** (126 MHz, CDCl_3_) δ 139.2 (C), 138.8 (C), 137.4 (C), 133.6 (Ar), 132.1 (Ar), 128.9 (Ar), 128.5 (Ar), 128.26 (Ar), 128.24 (Ar), 128.21 (Ar), 128.1 (Ar), 127.9 (Ar), 127.86 (Ar), 127.80 (Ar), 127.73 (Ar), 127.69 (Ar), 127.46 (Ar), 127.41 (Ar), 109.3 (CMe_2_), 102.2 (C1′), 98.2 (C1), 86.3 (C6′), 82.5 (C3′), 79.7 (C4), 78.5 (C3), 78.1 (C2′), 76.0 (C2), 75.8 (C5′), 75.6 (C4′), 75.0 (CH_2_), 74.9 (CH_2_), 73.6 (CH_2_), 70.7 (CH_2_), 64.5 (C5), 54.9 (OCH_3_), 27.7 (CH_3_), 26.4 (CH_3_), 18.0 (CH_3_). **HRMS** (ESI) *m*/*z*: [M + Na]^+^ calcd for C_50_H_56_O_10_SNa 871.3486; found 871.3484.

#### Methyl (6*S*)-6-Phenylthio-2,3,4,6-tetra-*O*-benzyl-α/β-d-galactopyranosyl-(1→4)-2,3,4-tri-*O*-benzyl-α-d-glucopyranoside (**19**)

Compounds **19α** and **19β** were obtained as a mixture of anomers (47.9 mg, 56%, α/β = 4.2:1) from the reaction of donor **7b** (62.0 mg, 78.2 μmol) and acceptor (43.6 mg, 93.8 μmol) by following the general procedure **GP5** for glycosylation (eluting with 10% ethyl acetate in hexanes). Repeated chromatography gave a pure sample of the α-isomer for full characterization.

#### α-Isomer

Colorless syrup. [α]_D_^21^ = +35.8 (*c* = 0.8, CHCl_3_). ^1^H NMR (500 MHz, CDCl_3_) δ 7.44–7.40 (m, 2H, ArH), 7.25 (d, *J* = 2.1 Hz, 11H, ArH), 5.60 (d, *J* = 3.8 Hz, 1H, H1′), 5.10–5.03 (m, 2H, CH_2_, H6′), 4.90–4.84 (m, 2H, CH_2_), 4.82 (d, *J* = 11.4 Hz, 1H, CH_2_), 4.72 (d, *J* = 11.7 Hz, 1H, CH_2_), 4.68–4.61 (m, 3H, CH_2_), 4.59–4.54 (m, 2H, CH_2_), 4.53–4.49 (m, 2H, H1, CH), 4.47 (d, *J* = 11.3 Hz, 1H, CH_2_), 4.37–4.32 (m, 2H, CH, CH_2_), 4.22 (d, *J* = 12.0 Hz, 1H, CH_2_), 4.00 (dd, *J* = 10.3, 3.8 Hz, 1H, H2′), 3.94 (dd, *J* = 9.6, 8.1 Hz, 1H, CH), 3.82 (dd, *J* = 8.7, 1.3 Hz, 1H, H5′), 3.80–3.75 (m, 1H, CH), 3.76–3.70 (m, 2H, CH), 3.53 (dd, *J* = 10.9, 5.9 Hz, 1H, H6b), 3.43 (dd, *J* = 9.6, 3.5 Hz, 1H, CH), 3.37 (dd, *J* = 10.8, 2.1 Hz, 1H, H6a), 3.32 (s, 3H, OCH_3_). ^**13**^**C{**^**1**^**H} NMR** (126 MHz, CDCl_3_) δ 139.2 (C), 138.8 (C), 138.7 (C), 138.6 (C), 138.4 (C), 138.2 (C), 137.8 (C), 133.09 (Ar), 133.06 (C), 129.1 (Ar), 128.48 (Ar), 128.47 (Ar), 128.45 (Ar), 128.33 (Ar), 128.29 (Ar), 128.25 (Ar), 128.23 (Ar), 127.96 (Ar), 127.93 (Ar), 127.89 (Ar), 127.81 (Ar), 127.66 (Ar), 127.61 (Ar), 127.58 (Ar), 127.54 (Ar), 127.50 (Ar), 127.4 (Ar), 127.1 (Ar), 127.0 (Ar), 97.6 (C1′), 97.4 (C1), 88.3 (C6′), 81.9 (CH_2_), 80.0 (CH_2_), 79.2 (CH_2_), 76.2 (CH), 75.8 (CH_2_), 75.0 (CH_2_), 74.6 (CH_2_), 74.5 (CH_2_), 73.4 (CH_2_), 73.3 (CH_2_), 73.2 (CH_2_), 73.1 (CH_2_), 72.9 (C5′), 71.4 (CH), 70.2 (CH), 69.5 (C6), 55.0 (OCH_3_). **HRMS** (ESI) *m*/*z*: [M + Na]^+^ calcd for C_68_H_70_O_11_SNa 1117.4531; found 1117.4504.

The β-isomer was not obtained pure and was characterized in the mixture of anomers by the following diagnostic signals**:**

^**1**^**H NMR** (500 MHz, CDCl_3_) δ 4.94 (d, *J* = 8.7 Hz, 1H, H6′), 4.56 (d, *J* = 3.8 Hz, 1H, H1′), 3.25 (d, *J* = 8.7 Hz, 1H, H5′); ^13^C NMR (126 MHz, CDCl_3_) δ 139.4 (C), 139.1 (C), 139.0 (C), 138.69 (C), 138.67 (C), 138.2 (C), 137.9 (C), 133.1 (Ar), 132.8 (Ar), 129.0 (Ar), 128.97 (Ar), 128.91 (Ar), 128.49 (Ar), 128.48 (Ar), 128.45 (Ar), 128.43 (Ar), 128.33 (Ar), 128.30 (Ar), 128.26 (Ar), 128.23 (Ar), 128.1 (Ar), 128.09 (Ar), 128.06 (Ar), 127.97 (Ar), 127.94 (Ar), 127.91 (Ar), 127.90 (Ar), 127.7 (Ar), 127.69 (Ar), 127.67 (Ar), 127.64 (Ar), 127.63 (Ar), 127.59 (Ar), 127.56 (Ar), 127.55 (Ar), 127.51 (Ar), 127.4 (Ar), 127.2 (Ar), 127.1 (Ar), 127.07 (Ar), 127.01 (Ar), 102.4 (C1′), 98.7 (C1), 87.3 (C6′), 82.7 (CH), 80.2 (CH), 80.0 (CH), 79.1 (CH), 76.5 (CH_2_), 75.9 (CH), 75.7 (CH), 75.6 (CH_2_), 75.2 (CH_2_), 75.1 (CH_2_), 73.9 (CH_2_), 73.3 (CH_2_), 73.1 (CH_2_), 72.3 (CH_2_), 70.2 (CH_2_), 67.9 (C6), 55.3 (OCH_3_).

#### Methyl (6*S*)-6-Phenylthio-2,3,4,6-tetra-*O-*benzyl-α,β-d-glucopyranosyl-(1→6)-2,3,4-tri-*O*-benzyl-α-d-glucopyranoside (**20**)

Compounds **20α** and **20β** were obtained as a mixture of anomers (66.2 mg, 80%, α/β = 1:8.7) from the reaction of donor **7c** (60.0 mg, 75.0 μmol) and acceptor (42.2 mg, 90.0 μmol) by following the general procedure **GP5** for glycosylation (eluting with 10% ethyl acetate in hexanes). Repeated column chromatography gave a pure sample of β-isomer for full characterization.

#### β-Isomer

Colorless syrup. [α]_D_^20^ = +7.1 (*c* 0.3, CHCl_3_). ^**1**^**H NMR** (500 MHz, CDCl_3_) δ 7.48–7.45 (m, 2H, Ar-H), 7.34–7.14 (m, 36H, Ar-H), 7.06 (dd, *J* = 6.7, 2.9 Hz, 2H, CH_2_), 5.39 (d, *J* = 2.0 Hz, 1H, H6′), 4.97 (m, 2H, CH_2_), 4.89 (d, *J* = 11.0 Hz, 1H, CH_2_), 4.83 (d, *J* = 12.0 Hz, 1H, CH_2_), 4.78 (d, *J* = 11.0 Hz, 1H, CH_2_), 4.76–4.69 (m, 5H, CH_2_, CH), 4.64 (d, *J* = 12.0 Hz, 1H, CH_2_), 4.60 (d, *J* = 3.6 Hz, 1H, H1), 4.55–4.51 (m, 2H, CH_2_), 4.38–4.30 (m, 2H, H1′, CH_2_), 4.23 (dd, *J* = 10.9, 2.0 Hz, 1H, H5′), 4.00 (t, *J* = 9.2 Hz, 1H, CH), 3.86 (dd, *J* = 10.2, 5.0 Hz, 1H, H5), 3.75 (t, *J* = 9.4 Hz, 1H, CH), 3.71–3.64 (m, 2H, CH), 3.61 (t, *J* = 8.9 Hz, 1H, H3′), 3.54–3.45 (m, 3H, CH), 3.32 (s, 3H, OCH_3_). ^**13**^**C{**^**1**^**H} NMR** (126 MHz, CDCl_3_) δ 139.0 (C), 138.5 (C), 138.46 (C), 138.43 (C), 138.3 (C), 138.2 (C), 137.3 (C), 136.3 (C), 131.6 (Ar), 129.2 (Ar), 128.57 (Ar), 128.51 (Ar), 128.49 (Ar), 128.44 (Ar), 128.40 (Ar), 128.3 (Ar), 128.2 (Ar), 128.08 (Ar), 128.05 (Ar), 128.02 (Ar), 127.9 (Ar), 127.8 (Ar), 127.79 (Ar), 127.75 (Ar), 127.73 (Ar), 127.72 (Ar), 127.69 (Ar), 127.64 (Ar), 127.1 (Ar), 104.6 (C1′), 98.1 (C1), 88.0 (C6′), 84.8 (CH), 82.1 (CH), 82.0 (CH), 80.0 (CH), 79.4 (CH), 78.3 (CH), 78.2 (CH), 77.4 (CH_2_), 77.1 (CH_2_), 76.9 (CH_2_), 75.9 (CH_2_), 75.7 (CH), 75.0 (CH_2_), 74.9 (CH_2_), 74.9 (CH_2_), 73.4 (CH_2_), 70.0 (CH), 69.8 (CH_2_), 68.9, 55.3 (CH_3_). **HRMS (**ESI) *m*/*z*: [M + Na]^+^ calcd for C_68_H_70_O_11_NaS 1117.45310; found 1117.4521.

The α-isomer was identified in the mixture by the following diagnostic signals: ^**1**^**H NMR** (500 MHz, CDCl_3_) δ 5.31 (d, *J* = 1.7 Hz, 1H, H6′), 5.15 (d, *J* = 3.5 Hz, 1H, H1′), 3.48–3.42 (m, 1H), 3.38 (s, 3H, OCH_3_).

#### (6*S*)-6-Phenylthio-2,3,4,6-tetra-*O-*benzyl-α,β-d-glucopyranosyl-(1→6)-1,2:3,4-*O*-diisopropylidene-α-d-galactopyranose (**21**)

Compounds **21α** and **21β** were obtained as a mixture of anomers (57.0 mg, 89%, α/β = 1:4. Integration of H6′ from the donor moiety) from the reaction of donor **7c** (57.0 mg, 71.8 μmol) and acceptor (22.4 mg, 86.0 μmol) by following the general procedure **GP5** for glycosylation (eluting with 10% ethyl acetate in hexanes). Repeated column chromatography gave a pure sample of β-isomer for full characterization.

### Synthesis of **21α** and **21β** on mmol Scale

A mixture of 1.056 g of donor **7c** (1.33 mmol, 1.0 equiv) and 0.416 g of 1,2:3,4-di-*O*-isopropylidene-α-d-galactopyranose (1.60 mmol, 1.2 equiv) was coevaporated with toluene twice, then taken up in 8.8 mL of anhydrous CH_2_Cl_2_ (0.15 M), and stirred for 1 h with 2.6 g of activated 4 Å AWMS (2 g/mmol of the donor) at room temperature under argon before cooling to −78 °C. The reaction mixture was treated with 48 μL of TMSOTf (0.26 mmol, 0.2 equiv) and stirred for 2 h at −78 °C before it was quenched with triethylamine (0.4 mL). The reaction mixture was diluted with dichloromethane (50 mL), filtered through a pad of celite, and washed with 50 mL of saturated aqueous NaHCO_3_. The organic layer was separated, dried over Na_2_SO_4_, filtered, and concentrated under reduced pressure. Purification by flash column chromatography on silica gel eluting with 10% ethyl acetate in hexanes afforded 1.096 g of **21α** and **21β** in a 92% yield with a 1:3 α/β; 98 mg of **21α** (8%) and 602 mg of **21β** (51% yield) were isolated after repeated silica gel column chromatography eluting with 10% ethyl acetate in hexanes. The anomeric ratio of the products was determined by integration of the H6′ signals in the^1^H NMR spectra.

#### β-Isomer

Colorless syrup. [α]_D_^20^ = −16.8 (*c* 0.6, CHCl_3_). ^**1**^**H NMR** (500 MHz, CDCl_3_) δ 7.55–7.49 (m, 2H, Ar-H), 7.46–7.40 (m, 2H, Ar-H), 7.39–7.07 (m, 19H, Ar-H), 7.04–6.99 (m, 2H, Ar-H), 5.57 (d, *J* = 5.0 Hz, 1H, H1), 5.36 (d, *J* = 1.5 Hz, 1H, H6′), 5.07 (d, *J* = 11.2 Hz, 1H, CH_2_), 4.95 (d, *J* = 10.8 Hz, 1H, CH_2_), 4.86 (d, *J* = 11.9 Hz, 1H, CH_2_), 4.77–4.67 (m, 3H, CH_2_), 4.60 (dd, *J* = 7.9, 2.4 Hz, 1H, H3), 4.52 (d, *J* = 11.8 Hz, 1H, CH_2_), 4.47 (d, *J* = 7.8 Hz, 1H, H1′), 4.31 (dd, *J* = 5.0, 2.4 Hz, 1H, H2), 4.25 (dd, *J* = 7.9, 1.5 Hz, 1H, H4), 4.22 (d, *J* = 10.9 Hz, 1H, CH_2_), 4.15–4.04 (m, 2H, H5, H6a′), 3.74 (m, 3H, H4′, H5′, H6′b), 3.63 (t, *J* = 8.5 Hz, 1H, H3′), 3.49 (dd, *J* = 9.1, 7.8 Hz, 1H, H2′), 1.50 (s, 3H, CH_3_), 1.47 (s, 3H, CH_3_), 1.33 (s, 3H, CH_3_), 1.30 (s, 3H, CH_3_). ^**13**^**C{**^**1**^**H} NMR** (126 MHz, CDCl_3_) δ 138.8 (C), 138.6 (C), 138.3 (C), 137.1 (C), 136.3 (C), 131.9 (Ar), 129.2 (Ar), 128.78 (Ar), 128.74 (Ar), 128.5 (Ar), 128.4 (Ar), 128.39 (Ar), 128.36 (Ar), 128.30 (Ar), 128.0 (Ar), 127.9 (Ar), 127.8 (Ar), 127.68 (Ar), 127.66 (Ar), 127.5 (Ar), 127.2 (Ar), 109.4 (CMe_2_), 108.6 (CMe_2_), 105.0 (C1′), 96.5 (C1), 88.0 (C6), 84.7 (C3′), 81.5 (C2′), 79.3 (C4′), 78.2 (C5′), 75.8 (CH_2_), 74.7 (CH_2_), 74.3 (CH_2_), 71.5 (C4), 70.9 (CH_2_), 70.6 (C3), 70.1 (CH_2_), 69.9 (CH_2_), 67.6 (CH_3_), 26.17 (CH_3_), 26.14 (CH_3_), 24.5 (CH_3_). **HRMS (**ESI) *m*/*z*: [M + Na]^+^ calcd for C_52_H_58_O_11_NaS 913.3592; found 913.3573.

#### α-Isomer

Colorless syrup. [α]_D_^20^ = +2.1 (*c* 2.0, CHCl_3_). ^**1**^**H NMR** (500 MHz, CDCl_3_) δ 7.55–7.50 (m, 2H, Ar-H), 7.41–7.38 (m, 2H, Ar-H), 7.37–7.16 (m, 17H, Ar-H), 7.14–7.10 (m, 2H, Ar-H), 7.05 (dd, *J* = 6.4, 3.0 Hz, 2H, Ar-H), 5.56 (d, *J* = 5.1 Hz, 1H, H1), 5.37 (d, *J* = 1.7 Hz, 1H, H6′), 5.17 (d, *J* = 3.5 Hz, 1H, H1′), 5.01 (d, *J* = 10.7 Hz, 1H, CH_2_), 4.88–4.73 (m, 4H, CH_2_), 4.69 (d, *J* = 11.9 Hz, 1H, CH_2_), 4.62 (dd, *J* = 7.9, 2.4 Hz, 1H, H3), 4.44–4.37 (m, 2H, H4, CH_2_), 4.34 (dd, *J* = 5.1, 2.4 Hz, 1H, H2), 4.24–4.18 (m, 2H, H5′, CH_2_), 4.11 (ddd, *J* = 8.0, 6.2, 1.9 Hz, 1H, H5), 4.07–4.02 (m, 2H, H3′, H6), 3.84 (dd, *J* = 10.4, 7.9 Hz, 1H, H6), 3.72 (t, *J* = 9.4 Hz, 1H, H4′), 3.64 (dd, *J* = 9.6, 3.6 Hz, 1H, H2′), 1.54 (s, 3H, CH_3_), 1.44 (s, 3H, CH_3_), 1.32 (s, 3H, CH_3_), 1.30 (s, 3H, CH_3_). ^**13**^**C{**^**1**^**H} NMR** (126 MHz, CDCl_3_) δ 138.9 (C), 138.5 (C), 138.4 (C), 136.9 (C), 136.6 (C), 132.0 (Ar), 129.2 (Ar), 128.6 (Ar), 128.51 (Ar), 128.50 (Ar), 128.49 (Ar), 128.42 (Ar), 128.41 (Ar), 128.2 (Ar), 128.0 (Ar), 127.9 (Ar), 127.8 (Ar), 127.74 (Ar), 127.70 (Ar), 127.3 (Ar), 109.4 (C), 108.7 (C), 96.7 (C1′), 96.5 (C1), 88.5 (C6′), 82.1 (C3′), 79.8 (C2′), 78.4 (C4′), 75.9 (CH_2_), 75.5 (C5′) 74.8 (CH_2_), 72.2 (CH_2_), 70.9 (C4), 70.8 (C3), 70.78 (C2), 70.6 (CH_2_), 65.9 (C6), 65.4 (C5), 26.3 (CH_3_), 26.2 (CH_3_), 25.1 (CH_3_), 24.9 (CH_3_). **HRMS (**ESI) *m*/*z*: [M + Na]^+^ calcd for C_52_H_58_O_11_NaS 913.3592; found 913.3584.

#### Adamantyl (6*S*)-6-Phenylthio-2,3,4,6-tetra-*O-*benzyl-α,β-d-glucopyranoside (**22**)

Compounds **22α** and **22β** were obtained as a mixture of anomers (31.0 mg, 59%, α/β = 1:7.5) from the reaction of donor **7c** (53.0 mg, 66.8 μmol) and acceptor (12.2 mg, 80.0 μmol) by following the general procedure **GP5** for glycosylation (eluting with 10% ethyl acetate in hexanes). Repeated column chromatography gave pure samples of α- and β-isomers for full characterization.

#### α-Isomer

Colorless syrup. [α]_D_^20^ = +46.5 (*c* 0.1, CHCl_3_). ^**1**^**H NMR** (600 MHz, CDCl_3_) δ 7.55–7.50 (m, 2H, Ar-H), 7.41–7.15 (m, 18H, Ar-H), 7.11–7.07 (m, 2H, Ar-H), 7.05–7.01 (m, 2H, Ar-H), 5.42 (d, *J* = 3.5 Hz, 1H, H1′), 5.39 (d, *J* = 1.6 Hz, 1H, H6′), 5.00 (d, *J* = 10.7 Hz, 1H, CH_2_), 4.82–4.75 (m, 3H, CH_2_), 4.71 (m, 2H, CH_2_), 4.41 (d, *J* = 11.9 Hz, 1H, CH_2_), 4.37 (dd, *J* = 9.8, 1.6 Hz, 1H, H5′), 4.13 (d, *J* = 10.9 Hz, 1H, CH_2_), 4.06 (t, *J* = 9.8 Hz, 1H, H4′), 3.69 (t, *J* = 9.8 Hz, 1H, H3′), 3.58 (dd, *J* = 9.8, 3.5 Hz, 1H, H2′), 2.20–2.17 (m, 3H, CH), 2.02–1.98 (m, 3H, CH_2_), 1.88 (dd, *J* = 11.5, 3.0 Hz, 3H, CH_2_), 1.65 (d, *J* = 3.0 Hz, 6H, CH_2_). ^**13**^**C{**^**1**^**H} NMR** (156 MHz, CDCl_3_) δ 138.8 (C), 138.3 (C), 138.2 (C), 136.9 (C), 136.8 (C), 131.8 (Ar), 129.1 (Ar), 128.6 (Ar), 128.4 (Ar), 128.3 (Ar), 128.2 (Ar), 128.1 (Ar), 128.0 (Ar), 127.9 (Ar), 127.8 (Ar), 127.7 (Ar), 127.6 (Ar), 127.5 (Ar), 127.0 (Ar), 90.0 (C1′), 88.6 (C6′), 81.8 (C4′), 80.0 (C2′), 78.7 (C3′), 75.6 (CH_2_), 75.1 (C5′), 74.9 (CH_2_), 74.7 (CH_2_), 73.0 (CH_2_), 70.3 (CH_2_), 42.5 (CH_2_), 36.3 (CH_2_), 30.7 (CH). **HRMS (**ESI) *m*/*z*: [M + Na]^+^ calcd for C_50_H_54_O_6_NaS 805.3533; found 805.3537.

#### β-Isomer

Colorless syrup. [α]_D_^20^ = +5.7 (*c* 1.0, CHCl_3_). ^**1**^**H NMR** (500 MHz, CDCl_3_) δ 7.49 (dt, *J* = 8.1, 1.2 Hz, 2H, Ar-H), 7.37–7.33 (m, 2H, Ar-H), 7.32–7.11 (m, 19H, Ar-H), 7.05 (m, 2H, Ar-H), 5.43 (d, *J* = 1.3 Hz, 1H, H6′), 5.02 (d, *J* = 11.0 Hz, 1H, CH_2_), 4.90 (d, *J* = 11.0 Hz, 1H, CH_2_), 4.83 (d, *J* = 11.9 Hz, 1H, CH_2_), 4.77–4.66 (m, 4H, H1′, CH_2_), 4.56 (d, *J* = 11.9 Hz, 1H, CH_2_), 4.32 (d, *J* = 10.9 Hz, 1H, CH_2_), 3.78–3.69 (m, 2H, H4′, H5′), 3.63 (t, *J* = 8.6 Hz, 1H, H3′), 3.48 (dd, *J* = 8.6, 7.8, 1H, H2′), 2.22–2.16 (m, 3H, CH), 1.99 (dt, *J* = 11.6, 3.1 Hz, 3H, CH_2_), 1.91 (dt, *J* = 11.6, 3.1 Hz, 3H, CH_2_), 1.66 (d, *J* = 3.1 Hz, 6H, CH_2_). ^**13**^**C{**^**1**^**H} NMR** (126 MHz, CDCl_3_) δ 138.7 (C), 138.6 (C), 138.4 (C), 137.5 (C), 136.9 (C), 131.4 (Ar), 129.1 (Ar), 128.45 (Ar), 128.44 (Ar), 128.36 (Ar), 128.34 (Ar), 128.31 (Ar), 128.0 (Ar), 127.76 (Ar), 127.70 (Ar), 127.68 (Ar), 127.63 (Ar), 127.0 (Ar), 97.2 (C1′), 88.2 (C6′), 85.1 (C3′), 82.3 (C2′), 79.3 (C5′), 78.4 (C4′), 75.8 (CH_2_), 75.6 (C), 75.0 (CH_2_), 74.8 (CH_2_), 69.6 (CH_2_), 42.9 (CH_2_), 36.4 (CH_2_), 30.8 (CH). **HRMS (**ESI) *m*/*z*: [M + Na]^+^ calcd for C_50_H_54_O_6_NaS 805.3533; found 805.3537.

#### (6*S*)-6-Phenylthio-2,3,4,6-tetra-*O-*benzyl-α,β-d-glucopyranosyl-(1→3)-1,2:5,6-di-*O*-isopropylidene-α-d-glucofuranose (**23**)

Compounds **23α** and **23β** were obtained as a mixture of anomers (125.0 mg, 75%, α/β = 1:2.9) from the reaction of donor **7c** (148.0 mg, 186.0 μmol) and acceptor (58.3 mg, 223.0 μmol) by following the general procedure **GP5** for glycosylation (eluting with 10% ethyl acetate in hexanes). Repeated column chromatography gave pure samples of α- and β-isomers for full characterization.

#### α-Isomer

Colorless syrup. [α]_D_^20^ = +34.9 (*c* 0.9, CHCl_3_).^**1**^**H NMR** (500 MHz, CDCl_3_) δ 7.53–7.46 (m, 2H, Ar-H), 7.37–7.11 (m, 21H, Ar-H), 7.02–6.97 (m, 2H, Ar-H), 5.87 (d, *J* = 3.5 Hz, 1H, H1), 5.34–5.30 (m, 2H, H6′, H1′), 4.97–4.91 (m, 2H, CH_2_), 4.86 (d, *J* = 11.7 Hz, 1H, CH_2_), 4.76–4.72 (m, 3H, CH, CH_2_), 4.68 (d, *J* = 11.7 Hz, 1H, CH_2_), 4.47 (td, *J* = 8.4, 6.1, 5.1 Hz, 1H, H5), 4.42 (d, *J* = 11.7 Hz, 2H, CH_2_), 4.31 (d, *J* = 2.7 Hz, 1H, H3), 4.17 (dd, *J* = 8.4, 2.7 Hz, 1H, H4), 4.15–4.10 (m, 2H, CH), 4.06–4.04 (m, 2H, CH), 3.92 (t, *J* = 9.4 Hz, 1H, H4′), 3.68 (t, *J* = 9.4 Hz, 1H, H3′), 3.58 (dd, *J* = 9.4, 3.5 Hz, 1H, H2′), 1.49 (s, 3H, CH_3_), 1.41 (s, 3H, CH_3_), 1.25 (s, 6H, CH_3_). ^**13**^**C{**^**1**^**H} NMR** (126 MHz, CDCl_3_) δ 138.5 (C), 138.2 (C), 138.1 (C), 138.0 (C), 136.7 (C), 136.2 (C), 131.8 (Ar), 129.3 (Ar), 128.9 (Ar), 128.7 (Ar), 128.68 (Ar), 128.65 (Ar), 128.5 (Ar), 128.49 (Ar), 128.47 (Ar), 128.2 (Ar), 128.1 (Ar), 127.86 (Ar), 127.8 (Ar), 127.7 (Ar), 127.6 (Ar), 111.9 (CMe_2_), 109.1 (CMe_2_), 105.4 (C1), 98.4 (C1′), 88.5 (C6′), 83.7 (CH), 81.6 (CH), 81.4 (CH), 81.3 (CH), 80.0 (CH), 78.2 (CH), 76.3 (CH), 75.9 (CH_2_), 75.1 (CH_2_), 73.2 (CH_2_), 72.5 (CH), 70.7 (CH_2_), 67.2 (CH_2_), 27.1 (CH_3_), 26.9 (CH_3_), 26.3 (CH_3_), 25.6 (CH_3_). **HRMS (**ESI) *m*/*z*: [M + Na]^+^ calcd for C_52_H_58_O_11_NaS 913.3592; found 913.3596.

#### β-Isomer

Colorless syrup. [α]_D_^20^ = +6.2 (*c* 0.7, CHCl_3_).^**1**^**H NMR** (500 MHz, CDCl_3_) δ 7.50–7.44 (m, 2H, Ar-H), 7.35–7.18 (m, 21H, Ar-H), 7.13–7.06 (m, 2H, Ar-H), 5.78 (d, *J* = 3.7 Hz, 1H, H1), 5.40 (d, *J* = 1.7 Hz, 1H, H6′), 4.89 (d, 11.0 Hz, 1H, CH_2_), 4.84 (d, *J* = 11.7 Hz, 1H, CH_2_), 4.79–4.72 (m, 4H, CH_2_), 4.59 (d, *J* = 11.7 Hz, 1H, CH_2_), 4.48–4.45 (m, 3H, CH), 4.44–4.40 (m, 2H, CH), 4.27 (d, *J* = 3.2 Hz, 1H, H3), 4.06 (d, *J* = 6.3 Hz, 2H, CH), 3.80–3.70 (m, 2H, H4′, H5′), 3.63 (t, *J* = 8.6 Hz, 1H, H3′), 3.38 (dd, *J* = 8.6, 7.8 Hz, 1H, H2′), 1.50 (s, 3H, CH_3_), 1.45 (s, 3H, CH_3_), 1.28 (s, 3H, CH_3_), 1.25 (s, 3H, CH_3_). ^**13**^**C{**^**1**^**H} NMR** (126 MHz, CDCl_3_) δ 138.4 (C), 138.2 (C), 138.1 (C), 137.5 (C), 136.2 (C), 131.3 (Ar), 129.2 (Ar), 128.59 (Ar), 128.51 (Ar), 128.47 (Ar), 128.42 (Ar), 128.2 (Ar), 127.89 (Ar), 127.84 (Ar), 127.80 (Ar), 127.7 (Ar), 127.1 (Ar), 112.0 (CMe_2_), 108.4 (CMe_2_), 105.2 (C1), 102.0 (C1′), 87.5 (CH), 84.6 (CH), 82.8 (CH), 82.1 (CH), 80.8 (CH), 80.2 (CH), 79.6 (CH), 78.2 (CH), 77.3 (CH), 77.1 (CH), 76.8 (CH), 75.8 (CH_2_), 75.0 (CH_2_), 73.8 (CH), 69.4 (CH_2_), 65.6 (CH_2_), 26.6 (CH_3_), 26.2 (CH_3_), 25.2 (CH_3_). **HRMS (**ESI) *m*/*z*: [M + Na]^+^ calcd for C_52_H_58_O_11_NaS 913.3592; found 913.3596.

#### Methyl (6*S*)-6-Phenylthio-2,3,4,6-tetra-*O-*benzyl-α,β-d-glucopyranosyl-(1→4)-2,3-*O*-isopropylidene-α-l-rhamnopyranoside (**24**)

Compounds **24α** and **24β** were obtained as a mixture of anomers (53.7 mg, 79%, α/β = 1:1.1) from the reaction of donor **7c** (63.0 mg, 79.4 μmol) and acceptor (20.8 mg, 95.3 μmol) by following the general procedure **GP5** for glycosylation (eluting with 10% ethyl acetate in hexanes). Repeated column chromatography gave pure samples of α- and β-isomers for full characterization.

#### α-Isomer

Colorless syrup. [α]_D_^20^ = +20.6 (*c* 1.5, CHCl_3_). ^**1**^**H NMR** (500 MHz, CDCl_3_) δ 7.54–7.48 (m, 2H, Ar-H), 7.35–7.12 (m, 19H, Ar-H), 7.11–7.06 (m, 2H, Ar-H), 7.05–6.98 (m, 2H, Ar-H), 5.31 (d, *J* = 1.8 Hz, 1H, H6′), 5.28 (d, *J* = 3.4 Hz, 1H, H1′), 4.92 (d, *J* = 10.8 Hz, 1H, CH_2_), 4.83–4.70 (m, 5H, H1, CH_2_), 4.65 (d, *J* = 11.8 Hz, 1H, CH_2_), 4.48 (t, *J* = 6.4 Hz, 1H, H4), 4.38 (d, *J* = 11.8 Hz, 1H, CH_2_), 4.32 (dd, *J* = 9.8, 1.8 Hz, 1H, H5′), 4.19 (d, *J* = 10.8 Hz, 1H, CH_2_), 4.10 (dd, *J* = 6.3, 0.8 Hz, 1H, H2), 3.97 (t, *J* = 9.5 Hz, 1H, H3′), 3.72 (m, 2H, H4′, H5), 3.57 (dd, *J* = 9.5, 3.4 Hz, 1H, H2′), 3.42 (dd, *J* = 9.7, 6.3 Hz, 1H, H3), 3.34 (s, 3H, CH_3_), 1.48 (s, 3H, CH_3_), 1.35 (d, *J* = 6.3 Hz, 3H, CH_3_), 1.28 (s, 3H, CH_3_). ^**13**^**C{**^**1**^**H} NMR** (126 MHz, CDCl_3_) δ 138.7 (C), 138.4 (C), 138.3 (C), 137.0 (C), 136.9 (C), 132.1 (Ar), 129.1 (Ar), 128.6 (Ar), 128.54 (Ar), 128.50 (Ar), 128.3 (Ar), 128.1 (Ar), 127.94 (Ar), 127.90 (Ar), 127.7 (Ar), 127.6 (Ar), 127.1 (Ar), 109.1 (CMe_2_), 98.4 (C1), 97.5 (C1′), 89.5 (C6′), 82.8 (C3), 81.7 (C3′), 80.5 (C2′), 78.7 (CH), 77.0 (CH), 75.7 (CH_2_), 75.6 (CH), 75.5 (CH), 74.8 (CH_2_), 73.5 (CH_2_), 70.5 (CH_2_), 64.4 (CH), 54.8 (OCH_3_), 28.1 (CH_3_), 26.1 (CH_3_), 18.1 (CH_3_). **HRMS (**ESI) *m*/*z*: [M + Na]^+^ calcd for C_50_H_56_O_10_NaS 871.3486; found 871.3477.

#### β-Isomer

Colorless syrup. [α]_D_^20^ = −27.6 (*c* 1.3, CHCl_3_). ^**1**^**H NMR** (500 MHz, CDCl_3_) δ 7.47–7.43 (m, 2H, Ar-H), 7.40–7.36 (m, 2H), 7.35–7.15 (m, 19H, Ar-H), 7.13–7.09 (m, 2H), 5.48 (d, *J* = 2.0 Hz, 1H, H6′), 5.01–4.86 (m, 4H, CH, CH_2_), 4.85–4.65 (m, 6H, CH_2_), 4.48 (d, *J* = 10.9 Hz, 1H, CH_2_), 4.24 (dd, *J* = 7.3, 5.6 Hz, 1H, H3), 4.11 (d, *J* = 5.6 Hz, 1H, H2), 3.82–3.62 (m, 5H, CH), 3.44 (t, *J* = 8.5 Hz, 1H), 3.40 (s, 3H, OCH_3_), 1.59 (s, 3H, CH_3_), 1.42 (d, *J* = 6.2 Hz, 3H, CH_3_), 1.35 (s, 3H, CH_3_). ^**13**^**C{**^**1**^**H} NMR** (126 MHz, CDCl_3_) δ 138.7 (C), 138.6 (C), 138.4 (C), 137.8 (C), 136.7 (C), 130.9 (Ar), 129.1 (Ar), 128.44 (Ar), 128.41 (Ar), 128.3 (Ar), 128.1 (Ar), 127.9 (Ar), 127.8 (Ar), 127.7 (Ar), 127.6 (Ar), 126.8 (Ar), 109.6 (CMe_2_), 102.9 (C1′), 98.1 (C1), 87.8 (CH), 84.8 (CH), 82.4 (CH), 79.3 (CH), 79.2 (CH), 78.5 (CH), 78.4 (CH), 76.0 (CH), 75.8 (CH_2_), 74.9 (CH_2_), 74.8 (CH_2_), 68.9 (CH_2_), 64.4 (CH), 54.9 (CH_3_), 28.2 (CH_3_), 26.5 (CH_3_), 17.8 (CH_3_). **HRMS (**ESI) *m*/*z*: [M + Na]^+^ calcd for C_50_H_56_O_10_NaS 871.3486; found 871.3477.

#### Methyl (6*S*)-6-Phenylthio-2,3,4,6-tetra-*O-*benzyl-α,β-d-glucopyranosyl-(1→4)-2,3,6-tri-*O*-benzyl-α-d-glucopyranoside (**25**)

Compounds **25α** and **25β** were obtained as a mixture of anomers (34.0 mg, 80%, α/β = 1.3:1) from the reaction of donor **7c** (30.5 mg, 38.4 μmol) and acceptor (21.4 mg, 46.1 μmol) by following the general procedure **GP5** for glycosylation (eluting with 10% ethyl acetate in hexanes). Repeated column chromatography gave a pure sample of α-isomer for full characterization.

#### α-Isomer

Colorless syrup. [α]_D_^20^ = +29.5 (*c* 0.6, CHCl_3_). ^**1**^**H NMR** (500 MHz, CDCl_3_) δ 7.46–7.41 (m, 2H, Ar-H), 7.32–7.12 (m, 40H, Ar-H), 7.11–7.07 (m, 2H, Ar-H), 7.04–7.00 (m, 2H, Ar-H), 5.65 (d, *J* = 3.3 Hz, 1H, H1′), 5.34 (d, *J* = 1.7 Hz, 1H, H6′), 4.97 (d, *J* = 11.5 Hz, 1H, CH_2_), 4.86 (d, *J* = 11.5 Hz, 1H, CH_2_), 4.82 (d, *J* = 10.7 Hz, 1H, CH_2_), 4.78 (d, *J* = 11.8 Hz, 1H, CH_2_), 4.74 (d, *J* = 11.0 Hz, 1H, CH_2_), 4.70–4.65 (m, 2H, CH_2_), 4.60–4.57 (m, 2H, CH), 4.59–4.51 (m, 3H, CH), 4.45–4.41 (m, 2H, CH_2_), 4.17 (d, *J* = 11.05 Hz, 1H, CH_2_) 4.13 (dd, *J* = 9.50, 2.0 Hz, 1H, CH), 4.06 (t, *J* = 8.7 Hz, 1H, CH), 3.96–3.82 (m, 5H, CH, CH_2_), 3.63 (t, *J* = 9.4 Hz, 1H, H3′), 3.58 (dd, *J* = 9.4, 3.3 Hz, 1H, H2′), 3.45 (dd, *J* = 9.4, 3.2 Hz, 1H, H2), 3.37 (s, 3H, CH_3_). ^**13**^**C{**^**1**^**H} NMR** (126 MHz, CDCl_3_) δ 139.2 (C), 138.6 (C), 138.5 (C), 138.3 (C), 138.2 (C), 138.0 (C), 136.9 (C), 136.7 (C), 131.2 (Ar), 129.2 (Ar), 128.7 (Ar), 128.5 (Ar), 128.46 (Ar), 128.42 (Ar), 128.40 (Ar), 128.33 (Ar), 128.29 (Ar), 128.24 (Ar), 128.0 (Ar), 127.9 (Ar), 127.7 (Ar), 127.6 (Ar), 127.4 (Ar), 127.09 (Ar), 127.05 (Ar), 126.9 (Ar), 97.7 (C1), 96.9 (C1′), 88.0 (C6′), 81.9 (CH), 81.8 (CH), 79.9 (CH), 79.5 (CH), 78.4 (CH), 76.5 (CH), 75.7 (CH_2_), 74.9 (CH_2_), 74.8 (CH_2_), 74.5 (CH), 73.4 (CH_2_), 73.3 (CH_2_), 73.1 (CH_2_), 70.1 (CH_2_), 69.7 (CH), 55.1 (CH_3_). **HRMS (**ESI) *m*/*z*: [M + Na]^+^ calcd for C_68_H_70_O_11_NaS 1117.4531; found 1117.4506.

The β-isomer was not obtained pure and was characterized in the mixture of anomers by the following diagnostic signals.

#### β-Isomer

^**1**^**H NMR** (500 MHz, CDCl_3_) δ 5.35 (d, *J* = 1.7 Hz, 1H; H6′), 4.46 (d, *J* = 7.3 Hz, 1H; H1′), 3.38 (s, 3H; OCH_3_).

#### (6*R*)-6-Phenylthio-2,3,4,6-tetra-*O-*benzyl-α,β-d-glucopyranosyl-(1→6)-1,2:3,4-*O*-diisopropylidene-α-d-galactopyranose (**26**)

Compounds **26α** and **26β** were obtained as a mixture of anomers (32.0 mg, 73%, α/β = 1:1.3) from the reaction of donor **7d** (38.5 mg, 48.0 μmol) and acceptor (15.2 mg, 58.0 μmol) by following the general procedure **GP5** for glycosylation (eluting with 10% ethyl acetate in hexanes). Repeated column chromatography gave pure samples of α- and β-isomers for full characterization.

#### α-Isomer

Colorless syrup. [α]_D_^20^ = +3.2 (*c* 0.6, CHCl_3_). ^**1**^**H NMR** (500 MHz, CDCl_3_) δ 7.39–7.11 (m, 23H), 7.01 (m, 2H), 5.52 (d, *J* = 5.1 Hz, 1H, H1), 5.36 (d, *J* = 1.5 Hz, 1H, H6′), 5.08 (d, *J* = 3.5 Hz, 1H, H1′), 5.00 (d, *J* = 10.9 Hz, 1H, CH_2_), 4.86 (d, *J* = 11.0 Hz, 1H, CH_2_), 4.76 (m, 2H, CH_2_, CH), 4.68–4.62 (m, 2H, CH), 4.57 (dd, *J* = 8.0, 2.4 Hz, 1H, H5), 4.39 (d, *J* = 11.2 Hz, 1H, CH_2_), 4.34 (dd, *J* = 8.0, 2.0 Hz, 1H, H4), 4.31–4.27 (m, 2H), 4.07 (m, 2H, H3,), 3.90 (dd, *J* = 10.4, 6.4 Hz, 1H), 3.82 (d, *J* = 9.3 Hz, 1H, H3′), 3.78 (dd, *J* = 7.4, 3.0 Hz, 1H, H2), 3.61 (dd, *J* = 9.6, 3.5 Hz, 1H, H2′), 1.54 (s, 3H, CH_3_), 1.42 (s, 3H, CH_3_), 1.32 (s, 3H, CH_3_), 1.29 (s, 3H, CH_3_). ^**13**^**C{**^**1**^**H} NMR** (126 MHz, CDCl_3_) δ 138.8 (C), 138.4 (C), 138.3 (C), 137.4 (C), 135.9 (C), 131.8 (Ar), 129.1 (Ar), 128.5 (Ar), 128.47 (Ar), 128.41 (Ar), 128.39 (Ar), 128.03 (Ar), 128.00 (Ar), 127.83 (Ar), 127.79 (Ar), 127.6 (Ar), 127.1 (Ar), 109.3 (CMe_2_), 108.7 (CMe_2_), 96.8 (C1′), 96.4 (C1), 89.3 (CH), 81.8 (CH), 80.0 (CH), 79.1 (CH), 75.6 (CH_2_), 74.8 (CH), 74.0 (CH_2_), 72.4 (CH_2_), 70.9 (CH), 70.8 (CH), 70.7 (CH), 70.1 (CH), 66.3 (CH), 65.6 (CH_2_), 26.3 (CH_3_), 26.2 (CH_3_), 25.0 (CH_3_), 24.8 (CH_3_). **HRMS (**ESI) *m*/*z*: [M + Na]^+^ calcd for C_52_H_58_O_11_NaS 913.3592; found 913.3573.

#### β-Isomer

Colorless syrup. [α]_D_^20^ = −17.5 (*c* 0.3, CHCl_3_). ^**1**^**H NMR** (500 MHz, CDCl_3_) δ 7.44–7.39 (m, 4H, Ar-H), 7.32–7.15 (m, 19H, Ar-H), 7.02–6.98 (m, 2H, Ar-H), 5.56 (d, *J* = 5.0 Hz, 1H, H1), 5.40 (d, *J* = 1.3 Hz, 1H, H6′), 5.05 (d, *J* = 11.2 Hz, 1H, CH_2_), 4.96 (d, *J* = 11.0 Hz, 1H, CH_2_), 4.84–4.77 (m, 3H, CH_2_), 4.75–4.69 (m, 2H, CH_2_), 4.57 (dd, *J* = 7.9, 2.4 Hz, 1H, H4), 4.51 (d, *J* = 7.7 Hz, 1H, H1′), 4.41 (d, *J* = 10.9 Hz, 1H, CH_2_), 4.30 (dd, *J* = 5.0, 2.4 Hz, 1H, H2), 4.24 (dd, *J* = 7.9, 1.9 Hz, 1H, H5), 4.13 (dd, *J* = 10.7, 3.5 Hz, 1H, H6a), 4.08 (dt, *J* = 9.2, 2.5 Hz, 1H, H3), 3.87–3.79 (m, 2H, H4′, H5′), 3.73 (dd, *J* = 10.7, 7.5 Hz, 1H, H6b), 3.68 (t, *J* = 9.1 Hz, 1H, H3′), 3.49 (dd, *J* = 9.1, 7.7 Hz, 1H, H2′), 1.51 (s, 3H, CH_3_), 1.42 (s, 3H, CH_3_), 1.30 (m, 6H, CH_3_). ^**13**^**C{**^**1**^**H} NMR** (126 MHz, CDCl_3_) δ 138.76 (C), 138.7 (C), 138.1 (C), 137.7 (C), 136.0 (C), 131.5 (Ar), 129.1 (Ar), 128.7 (Ar), 128.44 (Ar), 128.41 (Ar), 128.38 (Ar), 128.3 (Ar), 128.0 (Ar), 127.89 (Ar), 127.8 (Ar), 127.65 (Ar), 127.6 (Ar), 127.0 (Ar), 109.4 (C), 108.7 (C), 104.2 (C1′), 96.5 (C1), 88.7 (C6′), 84.5 (CH), 81.7 (CH), 78.8 (CH), 78.4 (CH), 77.35 (CH), 77.30 (CH), 77.1 (CH), 76.8 (CH), 75.6 (CH_2_), 75.0 (CH_2_), 74.4 (CH), 71.5 (CH), 70.9 (CH), 70.6 (CH_2_), 69.8 (CH_2_), 69.6 (CH_2_), 67.6 (CH), 26.2 (CH_3_), 26.1 (CH_3_), 25.1 (CH_3_), 24.5 (CH_3_). **HRMS (**ESI) *m*/*z*: [M + Na]^+^ calcd for C_52_H_58_O_11_NaS 913.3592; found 913.3573.

#### Adamantyl (6*R*)-6-Phenylthio-2,3,4,6-tetra-*O-*benzyl-α,β-d-glucopyranoside (**27**)

Compounds **27α** and **27β** were obtained as a mixture of anomers (63.0 mg, 88%, α/β = 4.8:1) from the reaction of donor **7d** (72.0 mg, 90.7 μmol) and acceptor (16.6 mg, 109.0 μmol) by following the general procedure **GP5** for glycosylation (eluting with 10% ethyl acetate in hexanes). Repeated column chromatography gave a pure sample of α-isomer for full characterization.

#### α-Isomer

Colorless syrup. [α]_D_^20^ = +46.2 (*c* 1.1, CHCl_3_). ^**1**^**H NMR** (500 MHz, CDCl_3_) δ 7.46–7.14 (m, 24H, Ar-H), 7.08 (m, 2H, Ar-H), 5.33 (m, 2H, CH, H1′, H6′), 5.02 (d, *J* = 10.8 Hz, 1H, CH_2_), 4.90 (d, *J* = 11.0 Hz, 1H, CH_2_), 4.84–4.75 (m, 2H, CH_2_), 4.71–4.67 (m, 2H, CH_2_), 4.55–4.45 (m, 2H, H5′, CH_2_), 4.39 (d, *J* = 10.8 Hz, 1H, CH_2_), 4.10 (t, *J* = 9.5 Hz, 1H, H3′), 3.83 (dd, *J* = 9.5, 8.7 Hz, 1H, H4′), 3.58 (dd, *J* = 9.5, 3.6 Hz, 1H, H2′), 2.08 (t, *J* = 3.2 Hz, 3H, CH), 1.89 (m, 3H, CH_2_), 1.83 (m, 3H, CH_2_), 1.64–1.54 (m, 6H, CH_2_). ^**13**^**C{**^**1**^**H} NMR** (126 MHz, CDCl_3_) δ 138.9 (C), 138.4 (C), 138.3 (C), 137.6 (C), 136.1 (C), 131.8 (Ar), 129.1 (Ar), 128.5 (Ar), 128.4 (Ar), 128.3 (Ar), 128.2 (Ar), 128.0 (Ar), 127.9 (Ar), 127.8 (Ar), 127.7 (Ar), 127.68 (Ar), 127.62 (Ar), 127.5 (Ar), 90.4 (C1′), 89.9 (C6′), 82.0 (C3′), 80.4 (C2′), 79.5 (C4′), 75.5 (CH_2_), 75.0 (CH_2_), 74.9 (CH_2_), 73.3 (CH_2_), 73.0 (C5′), 71.0 (CH_2_), 42.5 (CH_2_), 36.3 (CH_2_), 30.7 (CH). **HRMS (**ESI) *m*/*z*: [M + Na]^+^ calcd for C_50_H_54_O_6_NaS 805.3533; found 805.3519.

The β-isomer was not obtained pure and was characterized in the mixture of anomers by the following diagnostic signals.

#### β-Isomer

^**1**^**H NMR** (500 MHz, CDCl_3_) δ 5.40 (d, *J* = 1.9 Hz, 1H; H6′), 3.71 (m, 1H, H3′), 3.50 (dd, *J* = 9.23, 7.61 Hz, 1H, H2′).

#### Methyl (6*R*)-6-Phenylthio-2,3,4,6-tetra-*O-*benzyl-α,β-d-glucopyranosyl-(1→4)-2,3-*O*-isopropylidene-α-l-rhamnopyranoside (**28**)

Compounds **28α** and **28β** were obtained as a mixture of anomers (27.3 mg, 68%, α/β = 2:1) from the reaction of donor **7d** (37.0 mg, 46.7 μmol) and acceptor (12.2 mg, 56.0 μmol) by following the general procedure **GP5** for glycosylation (eluting with 10% ethyl acetate in hexanes). Repeated column chromatography gave a pure sample of α-isomer for full characterization.

#### α-Isomer

Colorless syrup. [α]_D_^20^ = +7.2 (*c* 0.6, CHCl_3_) ^**1**^**H NMR** (500 MHz, CDCl_3_) δ 7.44–7.38 (m, 2H, Ar-H), 7.37–7.11 (m, 23H, Ar-H), 5.32 (d, *J* = 1.4 Hz, 1H, H6′), 5.12 (d, *J* = 3.4 Hz, 1H, H1′), 4.95 (d, *J* = 10.8 Hz, 1H, CH_2_), 4.85 (d, *J* = 10.8 Hz, 1H, CH_2_), 4.83–4.79 (m, 3H, H1, CH_2_), 4.77 (d, *J* = 11.6 Hz, 1H, CH_2_), 4.69 (d, *J* = 11.6 Hz, 1H, CH_2_), 4.64 (d, *J* = 12.1 Hz, 1H, CH_2_), 4.58 (d, *J* = 10.8 Hz, 1H, CH_2_), 4.51 (dd, *J* = 9.9, 1.4 Hz, 1H, H5′), 4.21 (dd, *J* = 7.0, 5.7 Hz, 1H, H3), 4.06 (t, *J* = 9.5 Hz, 1H, H3′), 3.99 (d, *J* = 5.7 Hz, 1H, H2), 3.89 (dd, *J* = 9.9, 9.5 Hz, 1H, H4′), 3.75–3.68 (m, 1H, H5), 3.57 (dd, *J* = 9.5, 3.4 Hz, 1H, H2′), 3.38 (dd, *J* = 9.9, 7.0 Hz, 1H, H4), 3.34 (s, 3H, OCH_3_), 1.41 (s, 3H, CH_3_), 1.33 (d, *J* = 6.3 Hz, 3H, CH_3_), 1.16 (s, 3H, CH_3_). ^**13**^**C{**^**1**^**H} NMR** (126 MHz, CDCl_3_) δ 138.7 (C), 138.3 (C), 138.1 (C), 137.8 (C), 136.8 (C), 131.5 (Ar), 129.0 (Ar), 128.53 (Ar), 128.50 (Ar), 128.3 (Ar), 128.0 (Ar), 127.95 (Ar), 127.93 (Ar), 127.8 (Ar), 127.7 (Ar), 127.6 (Ar), 127.56 (Ar), 126.9 (Ar), 109.2 (C), 98.1 (C1′), 98.0 (C1), 89.8 (C6′), 82.3 (C4), 81.8 (C3′), 80.6 (C2′), 78.9 (C4′), 77.2 (C3), 75.80 (CH_2_), 75.6 (CH_2_), 75.0 (CH_2_), 74.9 (CH), 73.9 (CH_2_), 70.6 (CH_2_), 64.6 (CH5), 54.7 (CH_3_), 28.0 (CH_3_), 26.2 (CH_3_), 17.8 (CH_3_). **HRMS (**ESI) *m*/*z*: [M + Na]^+^ calcd for C_50_H_56_O_10_NaS 871.3486; found 871.3455.

The β-isomer was not obtained pure and was characterized in the mixture of anomers by the following diagnostic signals.

#### β-Isomer

^**1**^**H NMR** (500 MHz, CDCl_3_) δ 5.35 (s, 1H, H6′), 4.99 (d, *J* = 8.3 Hz, 1H, H1′), 3.38 (s, 3H, CH_3_).

### General Procedure for Desulfurization

A suspension of glycoside (1.0 equiv) in ethanol (0.1 M) was treated with Raney nickel (1 g/mmol) that had been previously washed with ethanol. The reaction mixture was stirred overnight under 1 atm of hydrogen at room temperature. The reaction mixture was diluted with ethanol and filtered through celite. The filter cake was washed with additional ethanol, and the combined filtrate was dried over Na_2_SO_4_, filtered through cotton, and concentrated under vacuum to afford a crude residue, which was purified by silica gel chromatography (0→10% CH_3_OH in CH_2_Cl_2_) to give the desired deprotected glycoside.

#### 6-*O*-(β-d-Galactopyranosyl)-1,2:3,4-*O*-diisopropylidene-α-d-galactopyranose (**29**)

Obtained from the deprotection of compound **9β** (15 mg) using the general procedure for desulfurization eluting from silica gel with 10% MeOH/ in CH_2_Cl_2_ to give 5.3 mg for a 75% yield, and spectral data consistent with the literature.^50,51^

Colorless syrup. [α]_D_^23^ = −36.5 (*c* = 0.3, MeOH) ^**1**^**H NMR** (500 MHz, CD_3_OD) δ 5.49 (d, *J* = 5.0 Hz, 1H), 4.60 (dd, *J* = 7.9, 2.4 Hz, 1H), 4.35 (dd, *J* = 5.0, 2.4 Hz, 1H), 4.28 (dd, *J* = 7.9, 1.6 Hz, 1H), 4.21 (d, *J* = 7.6 Hz, 1H), 4.07–3.98 (m, 2H), 3.80 (dd, *J* = 3.3, 1.1 Hz, 1H), 3.75–3.59 (m, 3H), 3.54–3.47 (m, 2H), 3.44 (dd, *J* = 9.7, 3.4 Hz, 1H), 1.49 (s, 3H), 1.37 (s, 3H), 1.32 (s, 3H), 1.30 (s, 3H). ^**13**^**C {**^**1**^**H} NMR** (126 MHz, CD_3_OD) δ 109.1 (C), 108.7 (C), 104.0 (CH), 96.4 (CH), 75.4 (CH), 73.4 (CH), 71.1 (CH), 70.65 (CH), 70.61 (CH), 68.9 (CH), 68.4 (CH), 67.6 (CH), 61.1 (CH_2_), 24.9 (CH_2_), 23.7 (CH_3_), 23.1 (CH_3_). **HRMS** (ESI) *m*/*z*: [M + Na]^+^ calcd for C_18_H_30_O_11_Na 445.1680; found 445.1674.

#### Adamantyl β-d-Galactopyranoside (**30**)

Obtained from the deprotection of compound **10β** (9.0 mg) using general procedure for desulfurization eluting from silica gel with 10% MeOH in CH_2_Cl_2_ to give 2.7 mg for a 76% yield.

Colorless syrup. [α]_D_^23^ = +1.4 (*c* = 0.3, MeOH). ^**1**^**H NMR** (500 MHz, MeOD-*d*_4_) δ 4.52–4.43 (m, 1H, H1), 3.82–3.77 (m, 1H, H4), 3.72–3.61 (m, 2H, H6), 3.49–3.39 (m, 3H, H2, H3, H5), 2.10 (m, 3H, Ada), 1.93–1.87 (m, 3H, Ada), 1.83–1.74 (m, 3H, Ada), 1.70–1.59 (m, 6H, Ada). ^**13**^**C {**^**1**^**H} NMR** (126 MHz, MeOD-*d*_4_) δ 96.41 (C1), 74.91 (CH), 74.55 (CH), 73.76 (CH), 71.24 (CH), 68.84 (C4), 61.02 (C6), 42.31 (Ada), 36.07 (Ada), 30.87 (Ada). **HRMS** (ESI) *m*/*z*: [M + Na]^+^ calcd for C_16_H_26_O_6_Na 337.1621; found 337.1613.

#### Methyl 4-*O*-(β-d-Galactopyranosyl)-2,3-*O*-isopropylidene-α-l-rhamnopyranoside (**31**)

Obtained from the deprotection of compound **12β** (12 mg) using the general procedure for desulfurization eluting from silica gel with 10% MeOH in CH_2_Cl_2_ to give 5.0 mg for a 93% yield.

Colorless syrup. [α]_D_^23^ = −27.6 (*c* = 0.7, MeOH). ^**1**^**H NMR** (500 MHz, MeOD-*d*_4_) δ 4.77 (s, 1H, H1), 4.73–4.65 (m, 1H, H1′), 4.23 (dd, *J* = 7.0, 5.7 Hz, 1H, CH), 4.07 (dd, *J* = 5.6, 0.8 Hz, 1H, CH), 3.84–3.80 (m, 1H, H4′), 3.75–3.57 (m, 4H, CH_2_, CH), 3.45–3.39 (m, 3H, H2′), 3.33 (s, 3H, OCH_3_), 1.47 (s, 3H, CH_3_), 1.30 (s, 3H, CH_3_), 1.24 (d, *J* = 6.0 Hz, 3H, CH_3_). ^**13**^**C {**^**1**^**H} NMR** (126 MHz, MeOD-*d*_4_) δ 109.0 (CMe2), 101.6 (C1′), 98.0 (C1), 78.1 (CH), 77.9 (CH), 75.9 (CH), 75.3 (CH), 73.7 (CH), 71.5 (CH), 68.8 (CH), 64.0 (CH), 60.8 (C6′), 53.8 (OCH_3_), 27.0 (CH_3_), 26.9 (CH_3_), 25.25 (CH_3_), 16.7 (CH_3_). **HRMS** (ESI) *m*/*z*: [M + Na]^+^ calcd for C_16_H_28_O_10_Na 403.1574; found 403.1567.

#### 3-*O*-(β-d-Galactopyranosyl)-1,2:5,6-di-*O*-isopropylidene-α-d-glucofuranose (**32**)

Obtained from the deprotection of compound **11β** (15 mg) using the general procedure for desulfurization eluting from silica gel with 10% MeOH in CH_2_Cl_2_ to give 6.5 mg for a 91% yield.

Colorless syrup. [α]_D_^23^ = −1.8 (*c* = 0.8, MeOH). ^**1**^**H NMR** (500 MHz, MeOD-*d*_4_) δ 5.91 (d, *J* = 3.7 Hz, 1H, H1), 4.68 (d, *J* = 3.7 Hz, 1H, H2), 4.46 (td, *J* = 6.3, 4.7 Hz, 1H, CH), 4.36 (d, *J* = 3.2 Hz, 1H, CH), 4.34–4.29 (m, 2H, H1′), 4.09 (dd, *J* = 8.5, 6.6 Hz, 1H, CH_2_), 4.02 (dd, *J* = 8.6, 6.1 Hz, 1H, CH_2_), 3.84–3.75 (m, 2H, H4′, H6b), 3.70 (dd, *J* = 11.4, 4.9 Hz, 1H, H6a), 3.56–3.49 (m, 1H, CH), 3.49–3.44 (m, 2H, CH), 1.46 (s, 3H, CH_3_), 1.40 (s, 3H, CH_3_), 1.33 (s, 3H, CH_3_), 1.31 (s, 3H, CH_3_); ^**13**^**C {**^**1**^**H} NMR** (126 MHz, CD_3_OD) δ 111.68 (CMe_2_), 108.31 (CMe_2_), 105.24 (C1), 102.32 (C1′), 83.13 (CH), 80.36 (CH), 80.12 (CH), 75.66 (CH), 73.71 (CH), 73.59 (CH), 70.67 (CH), 68.93 (CH), 65.45 (CH_2_), 61.25 (CH_2_), 25.77 (CH_3_), 25.41 (CH_3_), 25.11 (CH_3_), 24.08 (CH_3_). **HRMS** (ESI) *m*/*z*: [M + Na]^+^ calcd for C_18_H_30_O_11_Na 445.1680; found 445.1673.

#### Methyl α-d-Galactopyranosyl-(l-4)-α-d-glucopyranoside (**33**)

Obtained from the deprotection of compound **13α** (15 mg) using the general procedure for desulfurization. The crude residue obtained was triturated with CHCl_3_ (3 × 2 mL), filtered, and concentrated to afford pure product; yield (4.6 mg, 94%), and spectral data consistent with the reported literature.^52^

Colorless syrup. [α]_D_^23^ = +30.7 (*c* = 0.4, MeOH). ^**1**^**H NMR** (600 MHz, D_2_O) δ 5.33 (s, 1H, H1), 4.74 (d, *J* = 3.9 Hz, 1H, H1′), 3.95–3.73 (m, 7H, CH, CH_2_), 3.70-3.66 (m, 3H, CH, CH_2_), 3.59–3.49 (m, 2H, CH), 3.35 (s, 3H, OCH_3_). ^**13**^**C{**^**1**^**H} NMR** (151 MHz, D_2_O) δ 99.9 (C1), 99.0 (C1′), 77.0 (CH), 73.5 (CH), 71.7 (CH), 71.0 (CH), 70.1 (CH), 69.3 (CH), 69.1 (CH), 68.6 (CH), 61.1 (CH_2_), 60.5 (CH_2_), 55.0 (OCH_3_). **HRMS** (ESI) *m*/*z*: [M + Na]^+^ calcd for C_13_H_24_O_11_Na 379.1210; found 379.1206.

#### Methyl β-d-Galactopyranosyl-(l-4)-α-d-glucopyranoside (**34**)

Obtained from the deprotection of compound **13β** (16 mg) using the general procedure for desulfurization. The crude residue obtained was triturated with CHCl_3_ (3 × 2 mL), filtered, and concentrated to afford pure product; yield (4.6 mg, 89%).^53,54^

Colorless syrup. [α]_D_^23^ = +31.6 (*c* = 0.3, MeOH). ^1^H NMR (500 MHz, D_2_O) δ 4.84 (d, *J* = 3.8 Hz, 1H, H1), 4.47 (d, *J* = 7.8 Hz, 1H, H1′), 3.99–3.93 (m, 2H, CH), 3.90–3.73 (m, 6H, CH), 3.69 (dd, *J* = 9.8, 3.5 Hz, 2H, CH, CH_2_), 3.66–3.62 (m, 1H, CH), 3.57 (dd, *J* = 10.0, 7.8 Hz, 1H, CH_2_), 3.45 (s, 3H, OCH_3_). ^**13**^**C{**^**1**^**H} NMR** (126 MHz, D_2_O) δ 102.9 (C1′), 99.1 (C1), 78.46 (CH), 75.44 (CH), 72.63 (CH), 71.82 (CH), 71.05 (CH), 70.99 (CH), 70.34 (CH), 68.65 (CH), 61.12 (CH_2_), 59.99 (CH_2_), 55.18 (OMe). **HRMS** (ESI) *m*/*z*: [M + Na]^+^ calcd for C_13_H_24_O_11_Na 379.1210; found 379.1201.

#### Methyl 4-*O*-(α-d-Galactopyranosyl)-2,3-*O*-isopropylidene-α-l-rhamnopyranoside (**35**)

Obtained from the deprotection of compound **18α** (14 mg) using the general procedure for desulfurization eluting from silica gel with 10% MeOH in CH_2_Cl_2_ to give 5.9 mg for a 94% yield.

Colorless syrup. [α]_D_^23^ = +46.6 (*c* = 0.6, MeOH). ^**1**^**H NMR** (500 MHz, CD_3_OD) δ 4.91 (d, *J* = 3.8 Hz, 1H, H1′), 4.77 (s, 1H, H1), 4.12–4.06 (m, 2H, CH), 4.06–4.01 (m, 1H, CH), 3.96 (dd, *J* = 3.3, 1.3 Hz, 1H, H4′), 3.77 (dd, *J* = 10.2, 3.8 Hz, 1H, H2′), 3.72 (s, 4H, H5′, CH_2_), 3.69–3.64 (m, 1H, CH), 3.34 (s, 3H, OCH_3_), 1.48 (s, 3H, CH_3_), 1.36–1.25 (m, 6H, 2xCH_3_). ^**13**^**C{**^**1**^**H} NMR** (126 MHz, CD_3_OD) δ 109.00 (CMe2), 100.46 (C1′), 97.95 (C1), 81.24 (CH), 77.11 (CH), 75.97 (CH), 70.21 (CH), 69.99 (CH), 69.76 (C4′), 69.06 (CH), 64.80 (CH), 61.03 (CH_2_), 53.81 (OMe), 27.02 (CH_3_), 25.15 (CH_3_), 16.67 (CH_3_). **HRMS** (ESI) *m*/*z*: [M + Na]^+^ calcd for C_16_H_28_O_10_Na 403.1574; found 403.1567.

#### 6-*O*-β-d-Glucopyranosyl-1,2:3,4-*O*-diisopropylidene-α-d-galactopyranose (**36**)

Obtained from the deprotection of compound **21β** (20 mg) using the general procedure for desulfurization eluting from silica gel with 10% MeOH in CH_2_Cl_2_ to give 6.4 mg for a 67% yield.

Obtained from the deprotection of compound **26β** (12 mg) using the general procedure for desulfurization eluting from silica gel with 10% MeOH in CH_2_Cl_2_ to give 5.1 mg for an 83% yield.

#### 602 mg Scale Desulfurization with Raney Nickel

To a suspension of 603 mg of **21β** in 2 mL of ethanol while stirring was added 1 g of Raney nickel that had been previously washed with ethanol. The reaction mixture was stirred for 36 h under 1 atm of hydrogen at room temperature. The reaction mixture was diluted with 50 mL of ethanol and filtered through a pad of celite; the filter cake was washed with additional 50 mL of ethanol. The combined filtrate was dried over Na_2_SO_4_, filtered through cotton, and concentrated under reduced pressure to afford a crude residue. Two hundred and fifty-two milligrams were obtained after silica gel column chromatography eluting with 10% MeOH in CH_2_Cl_2_ for an 88% yield. The spectral data is consistent with the reported literature.^55,56^

Colorless syrup. [α]_D_^20^ = −41.8 (*c* 0.3, CH_3_CN) ^**1**^**H NMR** (500 MHz, CD_3_CN) δ 5.44 (d, *J* = 5.1 Hz, 1H, H1), 4.58 (dd, *J* = 8.0, 2.5 Hz, 1H, H3), 4.31 (dd, *J* = 5.1, 2.4 Hz, 1H, H2), 4.28–4.22 (m, 2H, H5, H1′), 3.97–3.88 (m, 2H, CH_2_6a, CH_2_6′a), 3.71 (d, *J* = 9.9 Hz 1H, CH_2_6b), 3.63–3.51 (m, 3H, H4′, H5′, CH_2_6′), 3.43 (br, 1H, CH_2_6′b), 3.35 (s, 1H, OH), 3.30 (s, 1H, OH), 3.27–3.23 (m, 1H, H3′), 3.20 (s, 1H, OH), 3.07 (app t, *J* = 8.4 Hz, 1H, H2′), 2.80 (s, 1H, OH), 1.47 (s, 3H, CH_3_), 1.35 (s, 3H, CH_3_), 1.28 (s, 6H, CH_3_). ^**13**^**C{**^**1**^**H} NMR** (126 MHz, CD_3_CN) δ 108.9 (CMe_2_), 108.4 (CMe_2_), 103.4 (CH), 96.3 (CH), 76.7 (CH), 76.3 (CH), 73.7 (CH), 71.0 (CH), 70.6 (CH), 70.5 (CH), 68.4 (CH_2_), 67.2 (CH), 61.9 (CH_2_), 25.4 (CH_3_), 25.3 (CH_3_), 24.2 (CH_3_), 23.7 (CH_3_). **HRMS (**ESI) *m*/*z*: [M + Na]^+^ calcd for C_18_H_30_O_11_Na 445.1680; found 445.1680.

#### Adamantyl β-d-Glucopyranoside (**37**)

Obtained from the deprotection of compound **22β** (25 mg) using the general procedure for desulfurization eluting from silica gel with 10% MeOH in CH_2_Cl_2_ to give 4.7 mg for a 46% yield. The spectral data is consistent with the literature.^57^

Colorless syrup. [α]_D_^20^ = −12.4 (*c* 0.3, CH_3_OH) ^**1**^**H NMR** (500 MHz, CD_3_OD) δ 4.53 (d, *J* = 7.8 Hz, 1H, H1), 3.79 (dd, *J* = 11.9, 2.3 Hz, 1H, H6a), 3.61 (dd, *J* = 11.9, 5.3 Hz, 1H, H6b), 3.33 (t, *J* = 8.7 Hz, 1H, H3), 3.24 (d, *J* = 8.2 Hz, 1H, H4), 3.26–3.19 (m, 1H), 3.09 (dd, *J* = 9.2, 7.8 Hz, 1H, H2), 2.11 (q, *J* = 3.3 Hz, 3H, Ada), 1.93–1.87 (m, 3H, Ada), 1.81–1.76 (m, 3H, Ada), 1.69–1.60 (m, 6H, Ada). ^**13**^**C{**^**1**^**H} NMR** (126 MHz, CD_3_OD) δ 95.8 (C1), 76.8 (CH), 76.2 (CH), 74.7 (CH), 73.7 (CH), 70.4 (CH), 61.5 (CH_2_), 42.2 (Ada), 36.0 (Ada), 30.8 (Ada). **HRMS (**ESI) *m*/*z*: [M + Na]^+^ calcd for C_16_H_26_O_6_Na 337.1622; found 337.1612.

#### Methyl 4-*O*-α*-*d-Glucopyranosyl-2,3-*O*-isopropylidene-α-l-rhamnopyranoside (**38**)

Obtained from the deprotection of compound **24α** (23.5 mg) using the general procedure for desulfurization eluting from silica gel with 10% MeOH in CH_2_Cl_2_ to give 8.6 mg for a 76% yield.

Obtained from the deprotection of compound **28α** (9.7 mg) using the general procedure for desulfurization eluting from silica gel with 10% MeOH in CH_2_Cl_2_ to give 3.8 mg for an 87% yield. The spectral data is consistent with the reported literature.^58^

White amorphous solid. [α]_D_^20^ = +41.7 (*c* 0.6, CH_3_OH) ^**1**^**H NMR** (500 MHz, CD_3_OD) δ 4.92 (d, *J* = 3.8 Hz, 1H, H1), 4.80 (s, 1H, H1′), 4.15–4.06 (m, 2H, H2, H3′), 3.84 (dt, *J* = 10.0, 3.1 Hz, 1H, H6′), 3.77 (m, 2H, CH), 3.70 (dq, *J* = 9.9, 6.3 Hz, 1H, H5), 3.65–3.60 (m, 1H, H4), 3.47–3.35 (m, 2H, H3, H4′), 3.36 (s, 3H, OCH_3_), 3.35–3.32 (m, 1H, CH) 1.51 (s, 3H, CH_3_), 1.33 (d, *J* = 6.4 Hz, 3H, CH_3_), 1.31 (s, 3H, CH_3_). ^**13**^**C{**^**1**^**H} NMR** (126 MHz, CD_3_OD) δ 110.3 (CMe_2_), 101.5 (C1′), 99.3 (C1), 82.54 (CH), 78.5 (CH), 77.3 (CH), 74.9 (CH), 73.7 (CH), 73.3 (CH), 71.3 (CH), 66.2 (CH), 62.1 (CH_3_), 55.1 (CH_2_), 28.4 (CH_3_), 26.5 (CH_3_), 17.9 (CH_3_). **HRMS (**ESI) *m*/*z*: [M + Na]^+^ calcd for C_16_H_28_O_10_Na 403.1575; found 403.1572.

#### Adamantyl α-d-Glucopyranoside (**39**)

Obtained from the deprotection of compound **27α** (16.7 mg) using the general procedure for desulfurization eluting from silica gel with 10% MeOH in CH_2_Cl_2_ to give 4.3 mg for a 64% yield.

White amorphous solid. [α]_D_^20^ = +19.1 (*c* 0.5, CH_3_CN) ^**1**^**H NMR** (500 MHz, CD_3_OD) δ 5.17 (d, *J* = 3.9 Hz, 1H, H1), 3.75–3.70 (m, 2H, H4, CH_2_6), 3.65 (dd, *J* = 12.3, 5.6 Hz, 1H, CH_2_6), 3.62–3.58 (m, 1H, H5), 3.30 (d, *J* = 3.9 Hz, 1H, H2), 3.26 (br, 1H, H3), 2.15–2.08 (m, 3H, CHada), 1.94–1.84 (m, 3H, Ada), 1.85–1.78 (m, 3H, Ada), 1.65 (dd, *J* = 3.8 Hz, 6H, Ada). ^**13**^**C{**^**1**^**H} NMR** (126 MHz, CD_3_OD) δ 91.6 (C1), 74.0 (CH), 73.9 (CH), 72.1 (CH), 71.8 (CH), 70.7 (CH), 61.4 (CH_2_), 42.2 (Ada), 36.1 (Ada), 30.8 (Ada). **HRMS (**ESI) *m*/*z*: [M + Na]^+^ calcd for C_16_H_26_O_6_Na 337.1622; found 337.1608.

## Data Availability

The data underlying this study are available in the published article and its online supplementary material.

## Supporting Information Available

Copies of ^1^H and ^13^C NMR spectra for all compounds (PDF)
