# Supplementary material for: Can Side-Chain Conformation and Glycosylation Selectivity of Hexopyranosyl Donors Be Controlled with a Dummy Ligand?
Source: J Org Chem. 2023 Mar 6;88(6):3678–96. doi: 10.1021/acs.joc.2c02889 (PMC10028612; doi:10.1021/acs.joc.2c02889)

Supporting information

For

Can Side Chain Conformation and Glycosylation Selectivity of Hexopyranosyl Donors be Controlled with a Dummy Ligand?

Kapil Upadhyaya,[a, §] Nicolas Osorio-Morales,[a,b, §] and David Crich[a,b,c*]

a) Department of Pharmaceutical and Biomedical Sciences, 250 West Green Street, Athens, GA 30602, USA
b) Department of Chemistry, University of Georgia, 302 E campus Rd, Athens, GA 30602, USA
c) Complex Carbohydrate Research Center, University of Georgia, 315 Riverbend Road, Athens, GA 30602, USA

§) These authors contributed equally to this work

e-mail: david.crich@uga.edu

Table of contents

	Spectra
Allyl 2,3,4-tri-*O*-benzyl-*β*-D-*galacto*pyranoside. **(1)**	S8-S9
Allyl 2,3,4-tri-*O*-benzyl-*β*-D-*gluco*pyranoside. **(2)**	S10-S11
Allyl 2,3,4,6,6-penta-*O*-benzyl-*β*-D-galactopyranoside **(3)**	S12-S13
Allyl 2,3,4,6,6-penta-*O*-benzyl-*β*-D-glucopyranoside **(4)**	S14-S15
Allyl (6*R*)-6-phenylthio-2,3,4,6-tetra-*O*-benzyl-*β*-D-galactopyranoside **(5a)**	S16-S121
Allyl (6*S*)-6-phenylthio-2,3,4,6-tetra-*O*-benzyl-*β*-D-galactopyranoside **(5b)**	S22-S27
Allyl (6*S*)-6-phenylthio-2,3,4,6-tetra-*O*-benzyl-*β*-D-glucopyranoside **(5c)**	S28-S33
Allyl (6*R*)-6-phenylthio-2,3,4,6-tetra-*O*-benzyl-*β*-D-glucopyranoside **(5d)**	S34-S39
(6*R*)-6-Phenylthio-2,3,4,6-tetra-*O*-benzyl-*α*,*β*-D-galactopyranose **(6a)**	S40-S41
(6*S*)-6-Phenylthio-2,3,4,6-tetra-*O*-benzyl-*α*,*β*-D-galactopyranose **(6b)**	S42-S43
(6*S*)-6-Phenylthio-2,3,4,6-tetra-*O*-benzyl-*α*,*β*-D-glucopyranose **(6c)**	S44-S45
(6*R*)-6-Phenylthio-2,3,4,6-tetra-*O*-benzyl-*α*,*β*-D-glucopyranose **(6d)**	S46-S47
Methyl (6*R*)-6-phenylthio-2,3,4,6-tetra-*O*-benzyl-*β*-D-galactopyranosyl-(1→6)-2,3,4-tri-*O*-benzyl-α-D-glucopyranoside **(8β)**	S48-S49

(6*R*)-6-Phenylthio-2,3,4,6-tetra-*O*-benzyl-*β*-D-galactopyranosyl-(1→6)-1,2:3,4-*O*-diisopropylidene-α-D-galactopyranose **(9β)**	S50-S51
Adamantyl (6*R*)-6-phenylthio-2,3,4,6-tetra-*O*-benzyl-*β*-D-galactopyranoside **(10β)**	S52-S53
(6*R*)-6-Phenylthio-2,3,4,6-tetra-*O*-benzyl-*α*-D-galactopyranosyl-(1→3)-1,2:5,6-di-*O*-isopropylidene-α-D-glucofuranose **(11*α*)**	S54-S55
(6*R*)-6-Phenylthio-2,3,4,6-tetra-*O*-benzyl-*β*-D-galactopyranosyl-(1→3)-1,2:5,6-di-*O*-isopropylidene-α-D-glucofuranose **(11β)**	S56-S57
Methyl (6*R*)-6-phenylthio-2,3,4,6-tetra-*O*-benzyl-*β*-D-galactopyranosyl-(1→4)-2,3-*O*-isopropylidene-α-L-rhamnopyranoside **(12β)**	S58-S59
Methyl (6*R*)-6-phenylthio-2,3,4,6-tetra-*O*-benzyl-*α*-D-galactopyranosyl-(1→4)-2,3,6-tri-*O*-benzyl-α-D-glucopyranoside **(13α)**	S60-S61
Methyl (6*R*)-6-phenylthio-2,3,4,6-tetra-*O*-benzyl-*β*-D-galactopyranosyl-(1→4)-2,3,6-tri-*O*-benzyl-α-D-glucopyranoside **(13β)**	S62-S63
Methyl (6*S*)-6-phenylthio-2,3,4,6-tetra-*O*-benzyl-*α*-D-galactopyranosyl-(1→6)-2,3,4-tri-*O*-benzyl-α-D-glucopyranoside **(14α)**	S64-S65
Methyl (6*S*)-6-phenylthio-2,3,4,6-tetra-*O*-benzyl-*β*-D-galactopyranosyl-(1→6)-2,3,4-tri-*O*-benzyl-α-D-glucopyranoside **(14β)**	S66-S67

(6*S*)-6-Phenylthio-2,3,4,6-tetra-*O*-benzyl-*α*-D-galactopyranosyl-(1→6)-1,2:3,4-*O*-diisopropylidene-α-D-galactopyranose **(15α)**	S68
(6*S*)-6-Phenylthio-2,3,4,6-tetra-*O*-benzyl-*β*-D-galactopyranosyl-(1→6)-1,2:3,4-*O*-diisopropylidene-α-D-galactopyranose **(15β)**	S69-S70
Adamantyl (6*S*)-6-phenylthio-2,3,4,6-tetra-*O*-benzyl-*α*-D-galactopyranose **(16α)**	S71-S72
Adamantyl (6*S*)-6-phenylthio-2,3,4,6-tetra-*O*-benzyl-*β*-D-galactopyranose **(16β)**	S73-S74
(6*S*)-6-Phenylthio-2,3,4,6-tetra-*O*-benzyl-*α*-D-galactopyranosyl-(1→3)-1,2:5,6-di-*O*-isopropylidene-α-D-glucofuranose **(17α)**	S75-S76
(6*S*)-6-Phenylthio-2,3,4,6-tetra-*O*-benzyl-*β*-D-galactopyranosyl-(1→3)-1,2:5,6-di-*O*-isopropylidene-α-D-glucofuranose **(17β)**	S77-S78
Methyl (6*S*)-6-phenylthio-2,3,4,6-tetra-*O*-benzyl-*α*-D-galactopyranosyl-(1→4)-2,3-*O*-isopropylidene-α-L-rhamnopyranoside **(18α)**	S79-S80
Methyl (6*S*)-6-phenylthio-2,3,4,6-tetra-*O*-benzyl-*β*-D-galactopyranosyl-(1→4)-2,3-*O*-isopropylidene-α-L-rhamnopyranoside **(18β)**	S81-S82
Methyl (6*S*)-6-phenylthio-2,3,4,6-tetra-*O*-benzyl-*α*-D-galactopyranosyl-(1→4)-2,3,6-tri-*O*-benzyl-α-D-glucopyranoside **(19α)**	S83-S84

Methyl (6*S*)-6-phenylthio-2,3,4,6-tetra-*O*-benzyl-*β*-D-galactopyranosyl-(1→4)-2,3,6-tri-*O*-benzyl-α-D-glucopyranoside **(19β)**	S85-S86
Methyl (6*S*)-6-phenylthio-2,3,4,6-tetra-*O*-benzyl-*β*-D-glucopyranosyl-(1→6)-2,3,4-tri-*O*-benzyl α-D-glucopyranoside **(20β)**	S87-S88
Methyl (6*S*)-6-phenylthio-2,3,4,6-tetra-*O*-benzyl-*α*-D-glucopyranosyl-(1→6)-2,3,4-tri-*O*-benzyl-α-D-glucopyranoside **(20α)**	S89
(6*S*)-6-Phenylthio-2,3,4,6-tetra-*O*-benzyl-*β*-D-glucopyranosyl-(1→6)-1,2:3,4-*O*-diisopropylidene-α-D-galactopyranose **(21 α)**	S90-S91
(6*S*)-6-Phenylthio-2,3,4,6-tetra-*O*-benzyl-*α*-D-glucopyranosyl-(1→6)-1,2:3,4-*O*-diisopropylidene-α-D-galactopyranose **(21 β)**	S92-S93
Adamantyl (6*S*)-6-phenylthio-2,3,4,6-tetra-*O*-benzyl-*α*-D-glucopyranoside **(22α)**	S94-S95
Adamantyl (6*S*)-6-phenylthio-2,3,4,6-tetra-*O*-benzyl-*β*-D-glucopyranoside **(22β)**	S96-97
(6*S*)-6-Phenylthio-2,3,4,6-tetra-*O*-benzyl-*α*-D-glucopyranosyl-(1→3)-1,2:5,6-di-*O*-isopropylidene-α-D-glucofuranose **(23α)**	S98-S99
(6*S*)-6-Phenylthio-2,3,4,6-tetra-*O*-benzyl-*β*-D-glucopyranosyl-(1→3)-1,2:5,6-di-*O*-isopropylidene-α-D-glucofuranose **(23β)**	S100-S101

Methyl (6*S*)-6-phenylthio-2,3,4,6-tetra-*O*-benzyl-*α*-D-glucopyranosyl-(1→4)-2,3-*O*-isopropylidene-α-L-rhamnopyranoside. **(24α)**	S102-S103
Methyl (6*S*)-6-phenylthio-2,3,4,6-tetra-*O*-benzyl-*β*-D-glucopyranosyl-(1→4)-2,3-*O*-isopropylidene-α-L-rhamnopyranoside. **(24β)**	S104-S105
Methyl (6*S*)-6-phenylthio-2,3,4,6-tetra-*O*-benzyl-*α*-D-glucopyranosyl-(1→4)-2,3,6-tri-*O*-benzyl-α-D-glucopyranoside **(25α)**	S106-S107
Methyl (6*S*)-6-phenylthio-2,3,4,6-tetra-*O*-benzyl-*β*-D-glucopyranosyl-(1→4)-2,3,6-tri-*O*-benzyl-α-D-glucopyranoside **(25β)**	S108
(6*R*)-6-Phenylthio-2,3,4,6-tetra-*O*-benzyl-*α*-D-glucopyranosyl-(1→6)-1,2:3,4-*O*-diisopropylidene-α-D-galactopyranose **(26α)**	S109-S110
(6*R*)-6-Phenylthio-2,3,4,6-tetra-*O*-benzyl-*β*-D-glucopyranosyl-(1→6)-1,2:3,4-*O*-diisopropylidene-α-D-galactopyranose **(26β)**	S111-S112
Adamantyl (6*R*)-6-phenylthio-2,3,4,6-tetra-*O*-benzyl-*α*-D-glucopyranoside. **(27α)**	S113-S114
Adamantyl (6*R*)-6-phenylthio-2,3,4,6-tetra-*O*-benzyl-*β*-D-glucopyranoside. **(27β)**	S115
Methyl (6*R*)-6-phenylthio-2,3,4,6-tetra-*O*-benzyl-*α*-D-glucopyranosyl-(1→4)-2,3-*O*-isopropylidene-α-L-rhamnopyranoside. **(28α)**	S116-S117
Methyl (6*R*)-6-phenylthio-2,3,4,6-tetra-*O*-benzyl-*β*-D-glucopyranosyl-(1→4)-2,3-*O*-isopropylidene-α-L-rhamnopyranoside. **(28β)**	S118

6-*O*-(β-D-Galactopyranosyl)-1,2:3,4-*O*-diisopropylidene-α-D-galactopyranose **(29)**	S119-S120
Adamantyl β-D-galactopyranose **(30)**	S121-S122
Methyl 4-*O*-(β-D-galactopyranosyl)-2,3-*O*-isopropylidene-α-L-rhamnopyranose **(31)**	S123-S124
3-*O*-(β-D-Galactopyranosyl)-1,2:5,6-di-*O*-isopropylidene-α-D-glucofuranose **(32)**	S125-S126
Methyl α-D-galactopyranosyl-(l-4)-α-D-glucopyranose **(33)**	S127-S128
Methyl β-D-galactopyranosyl-(l-4)-α-D-glucopyranose **(34)**	S129-S130
Methyl 4-*O*-(α-D-galactopyranosyl)-2,3-*O*-isopropylidene-α-L-rhamnopyranose **(35)**	S131-S132
6-*O*-β-D-Glucopyranosyl-1,2:3,4-*O*-diisopropylidene-α-D-galactopyranose. **(36)**	S133-S134
Adamantyl *β*-D-glucopyranose. **(37)**	S135-S136
Methyl 4-*O*-*α*-D-glucopyranosyl-2,3-*O*-isopropylidene-α-L-rhamnopyranose **(38)**	S137-S138
Adamantyl *α*-D-glucopyranose. **(39)**	S139-S140

¹H NMR (500 MHz, $CDCl_3$) spectrum of Allyl 2,3,4-tri-*O*-benzyl-*β*-D-galactopyranoside (1)

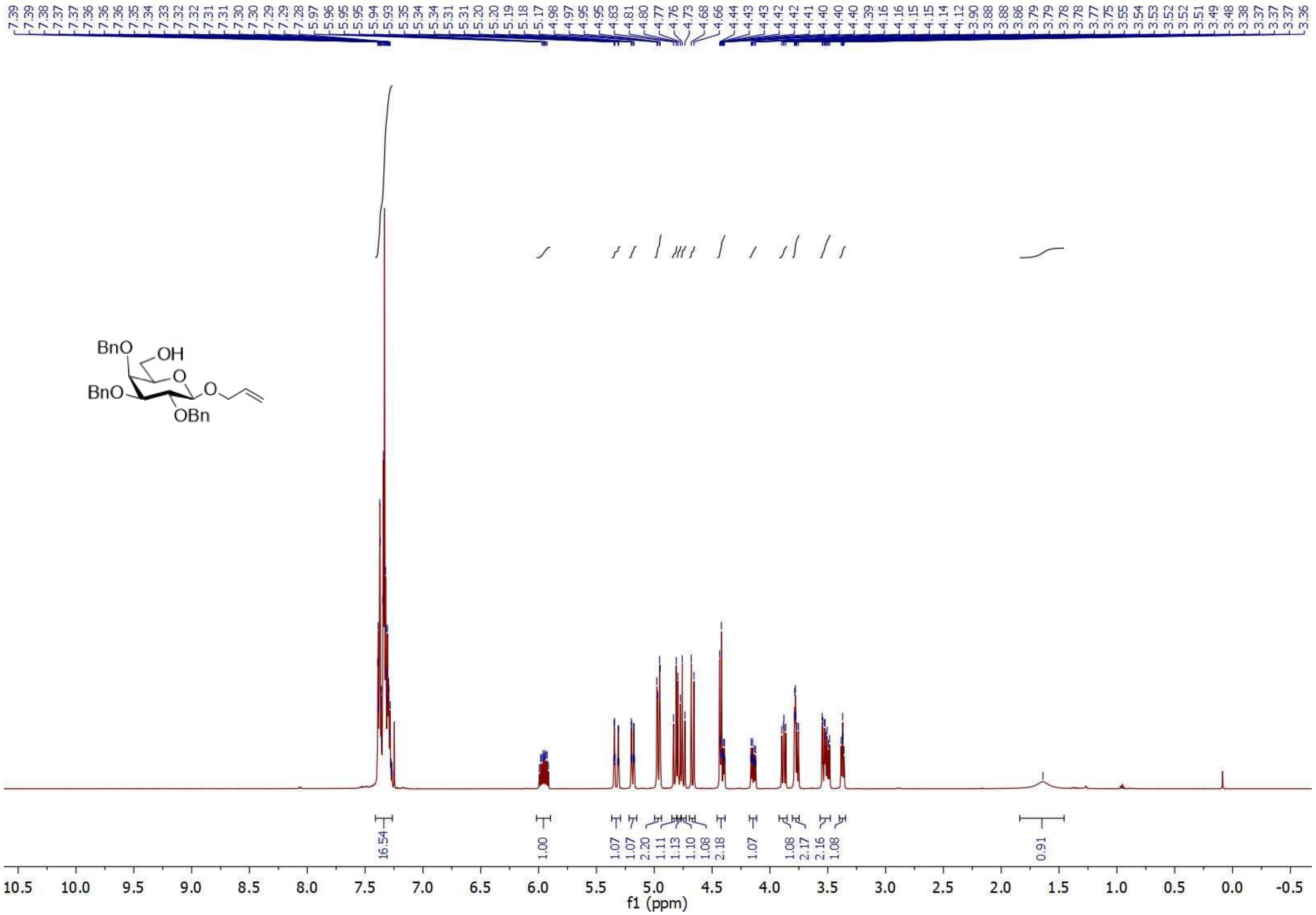

^{13}C {^{1}H} NMR (126 MHz, $CDCl_3$) spectrum of Allyl 2,3,4-tri-*O*-benzyl-*β*-D-galactopyranoside (1)

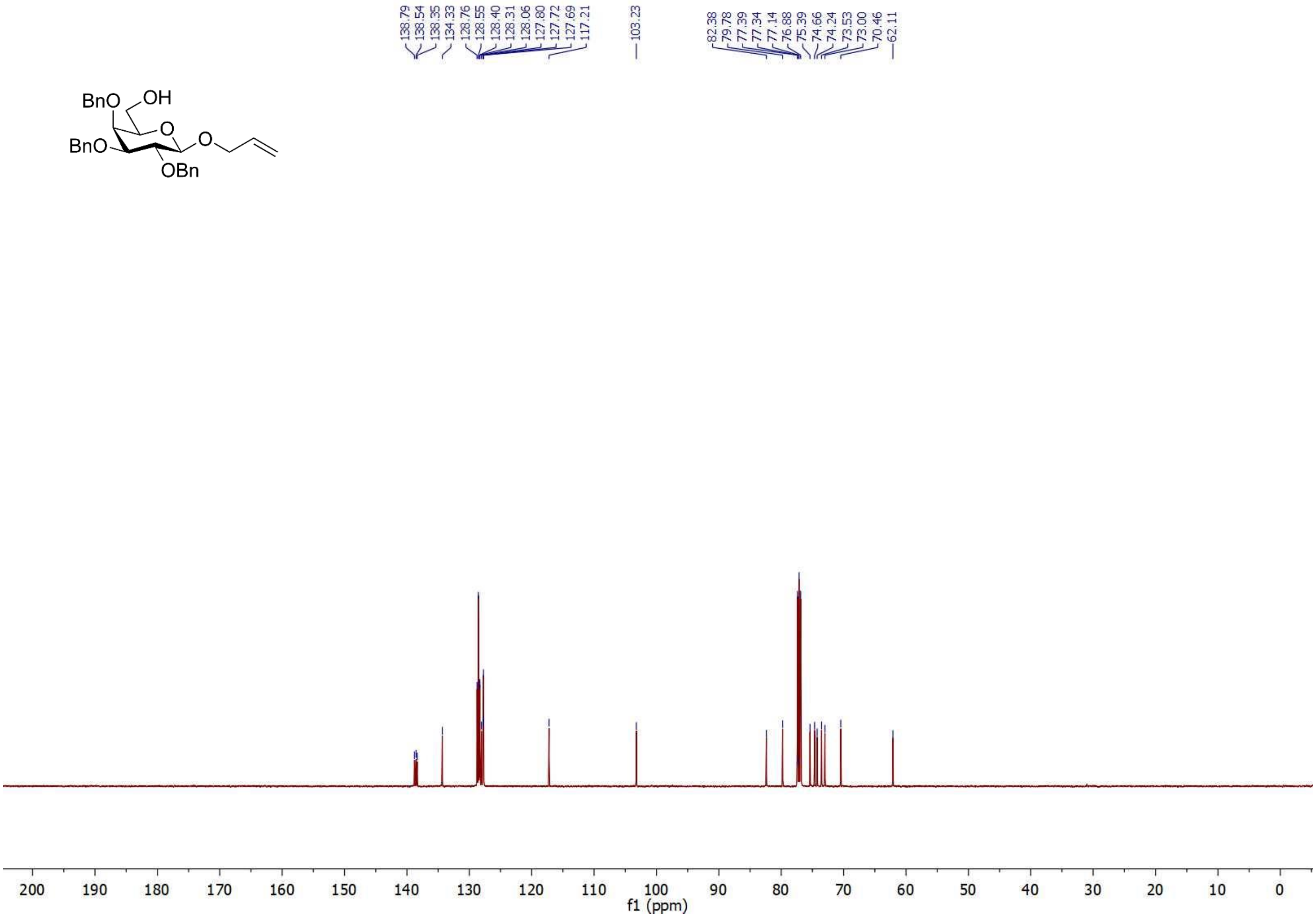

¹H NMR (500 MHz, C_6D_6) spectrum of Allyl 2,3,4-tri-*O*-benzyl-*β*-D-*gluco*pyranoside. **2**

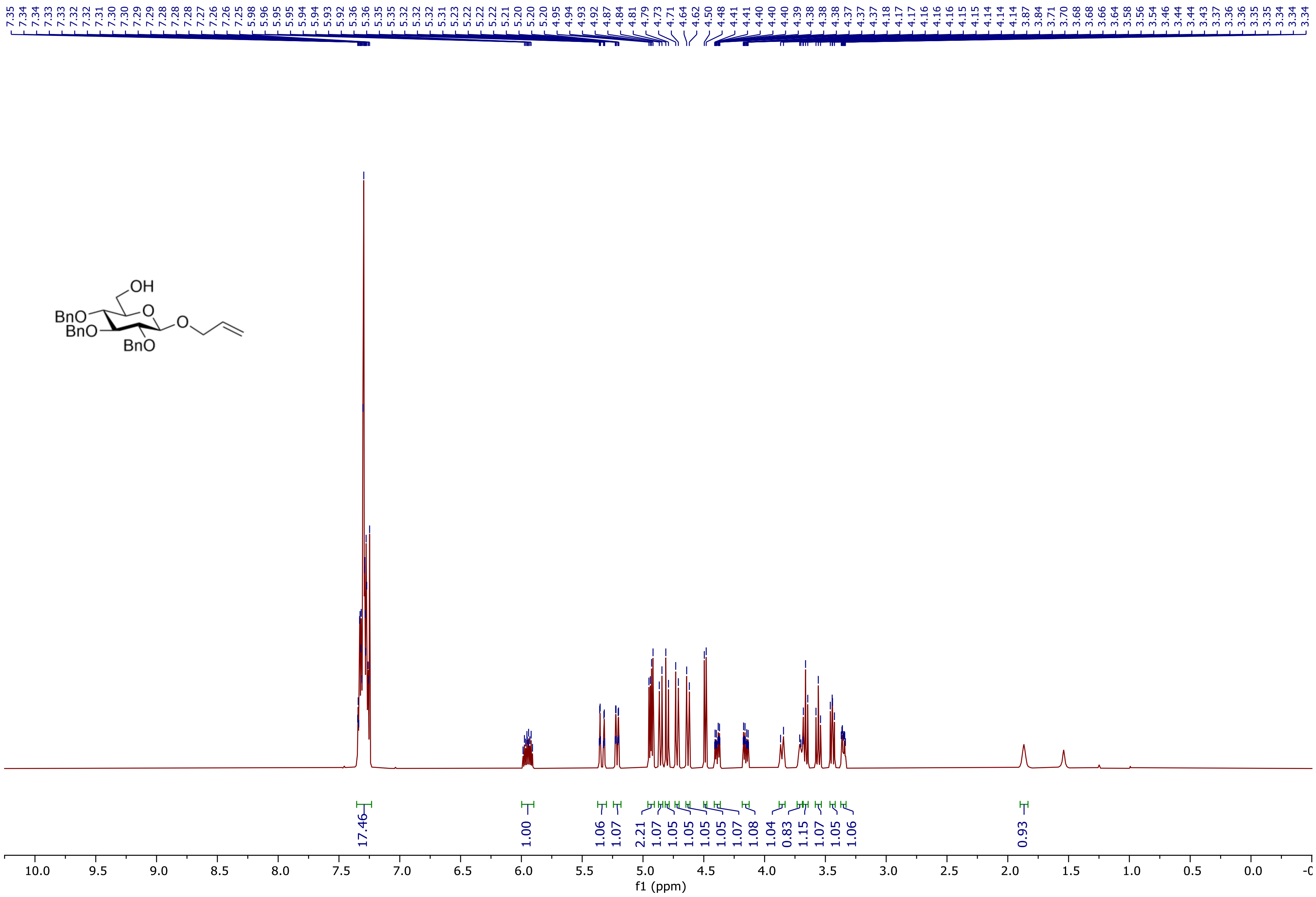

^{13}C{^{1}H} NMR (126 MHz, C_6D_6) spectrum of Allyl 2,3,4-tri-*O*-benzyl-*β*-D-*gluco*pyranoside. **2**

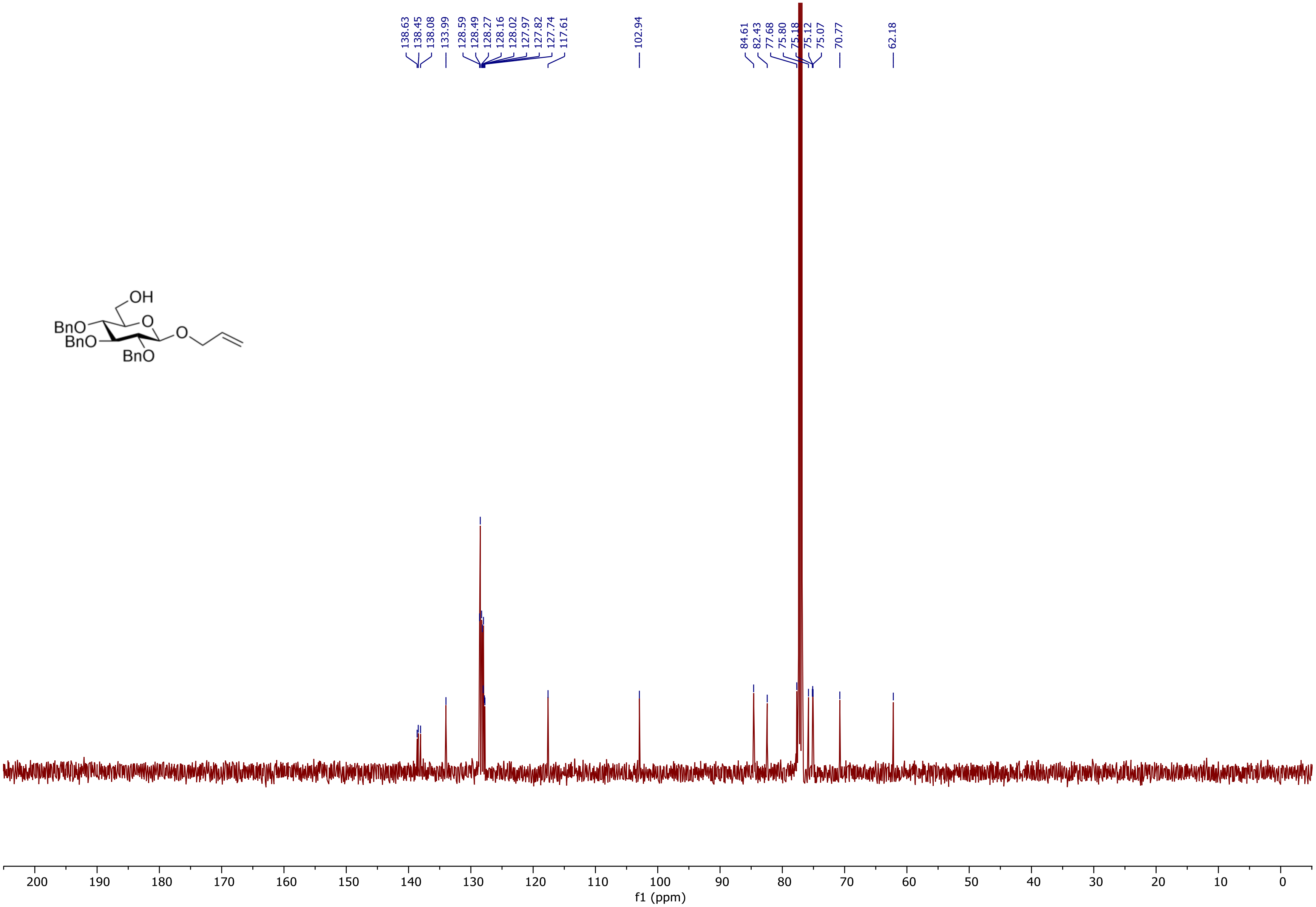

^{1}H NMR (500 MHz, $CDCl_3$) Spectrum of allyl 2,3,4,6,6-penta-*O*-benzyl-*β*-D-*galacto*pyranoside. **3**

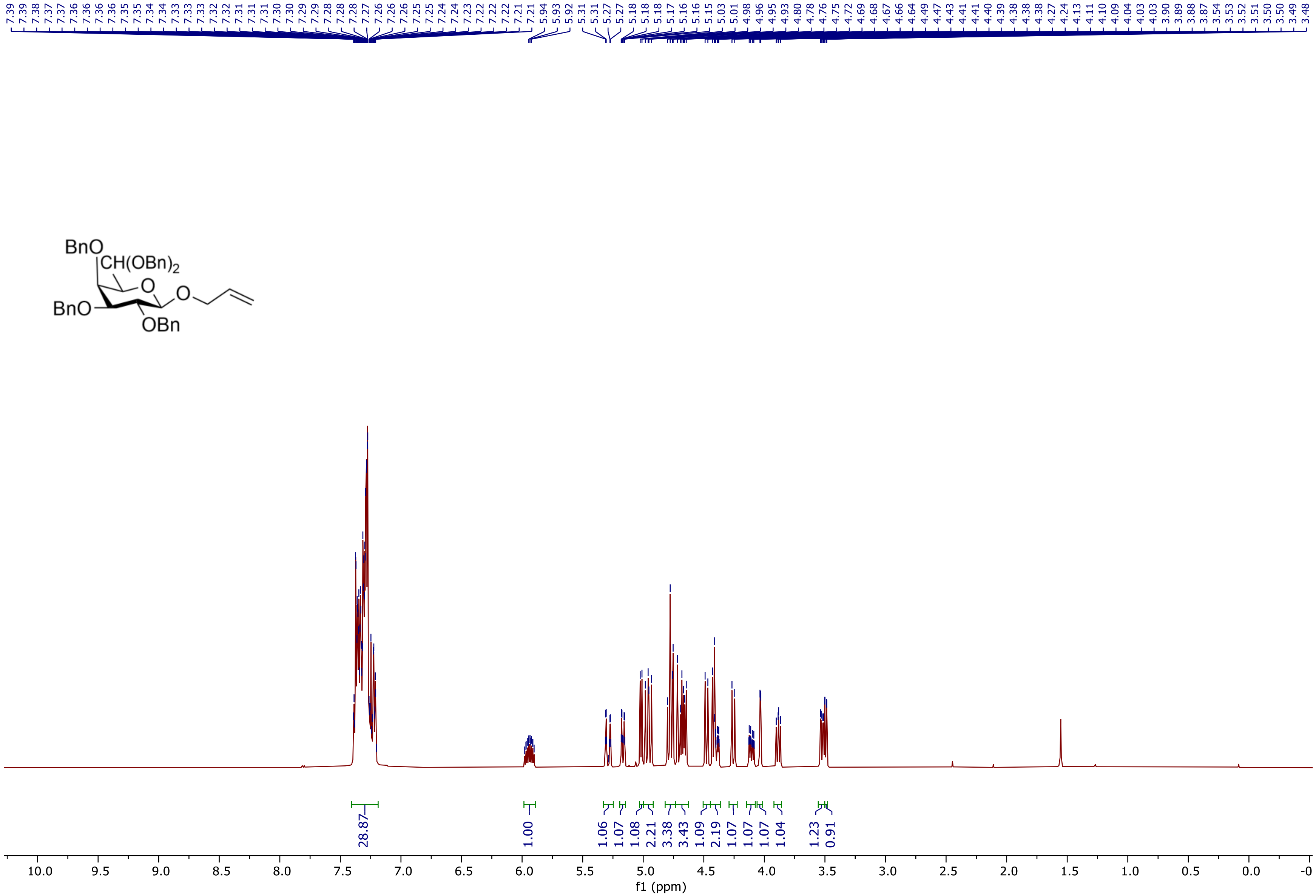

$^{13}C\{^{1}H\}$ NMR (126 MHz, $CDCl_3$) Spectrum of allyl 2,3,4,6,6-penta-*O*-benzyl-*β*-D-*galacto*pyranoside. **3**

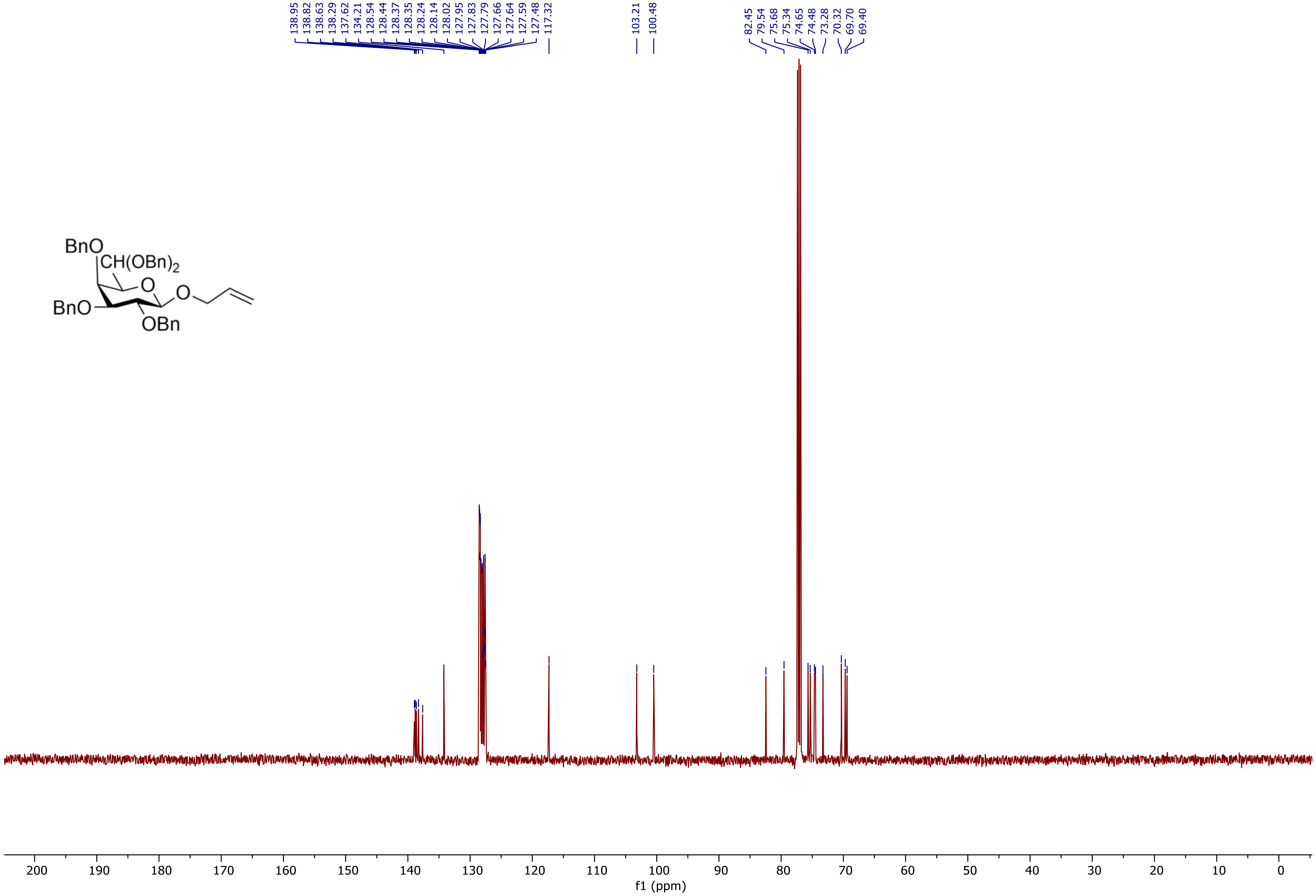

^{1}**H NMR** (500 MHz, C_6D_6) Spectrum of allyl 2,3,4,6,6-penta-*O*-benzyl-*β*-D-*gluco*pyranoside. **4**

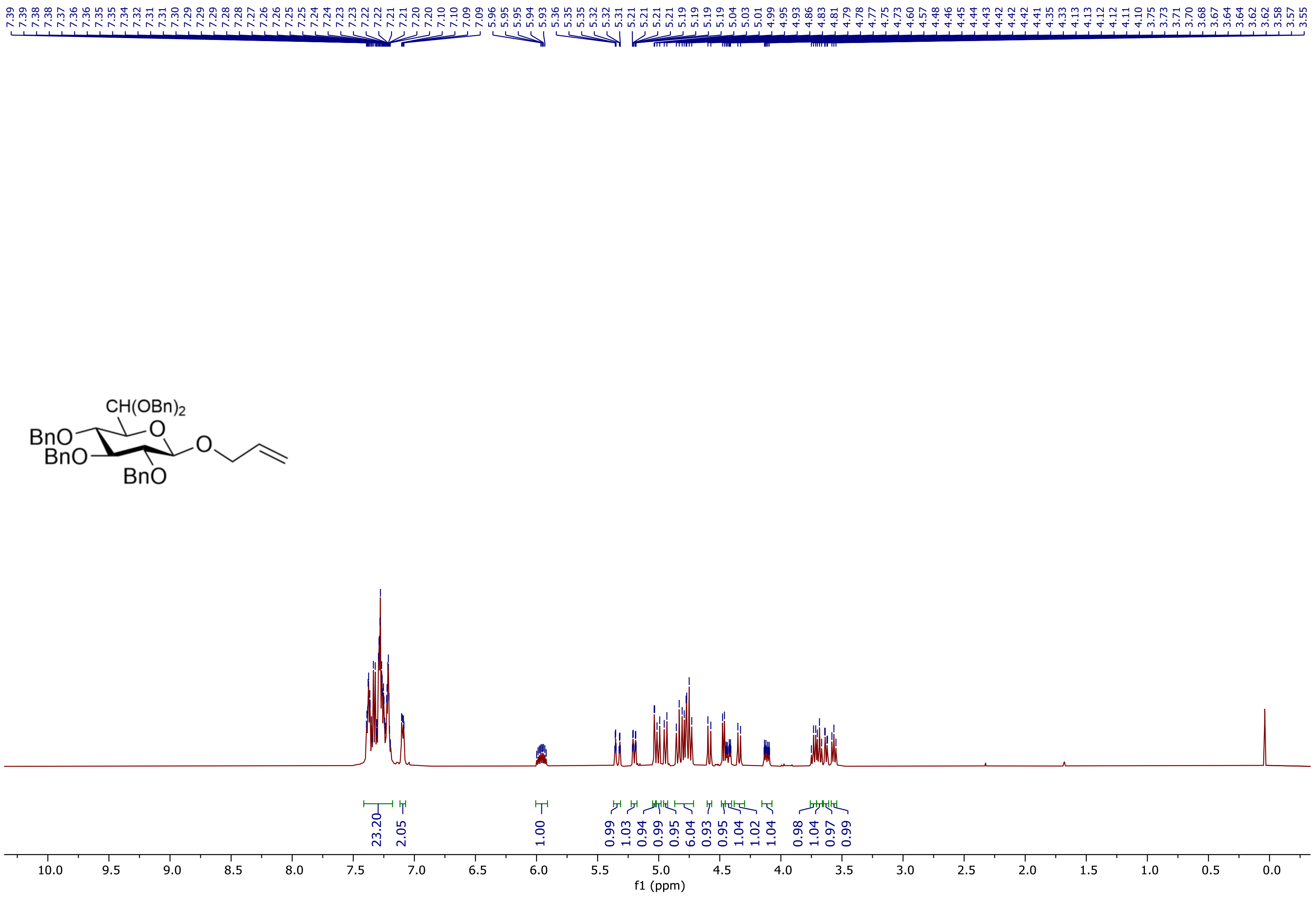

$^{13}C\{^{1}H\}$ NMR (126 MHz, C_6D_6) Spectrum of allyl 2,3,4,6,6-penta-*O*-benzyl-*β*-D-*gluco*pyranoside. **4**

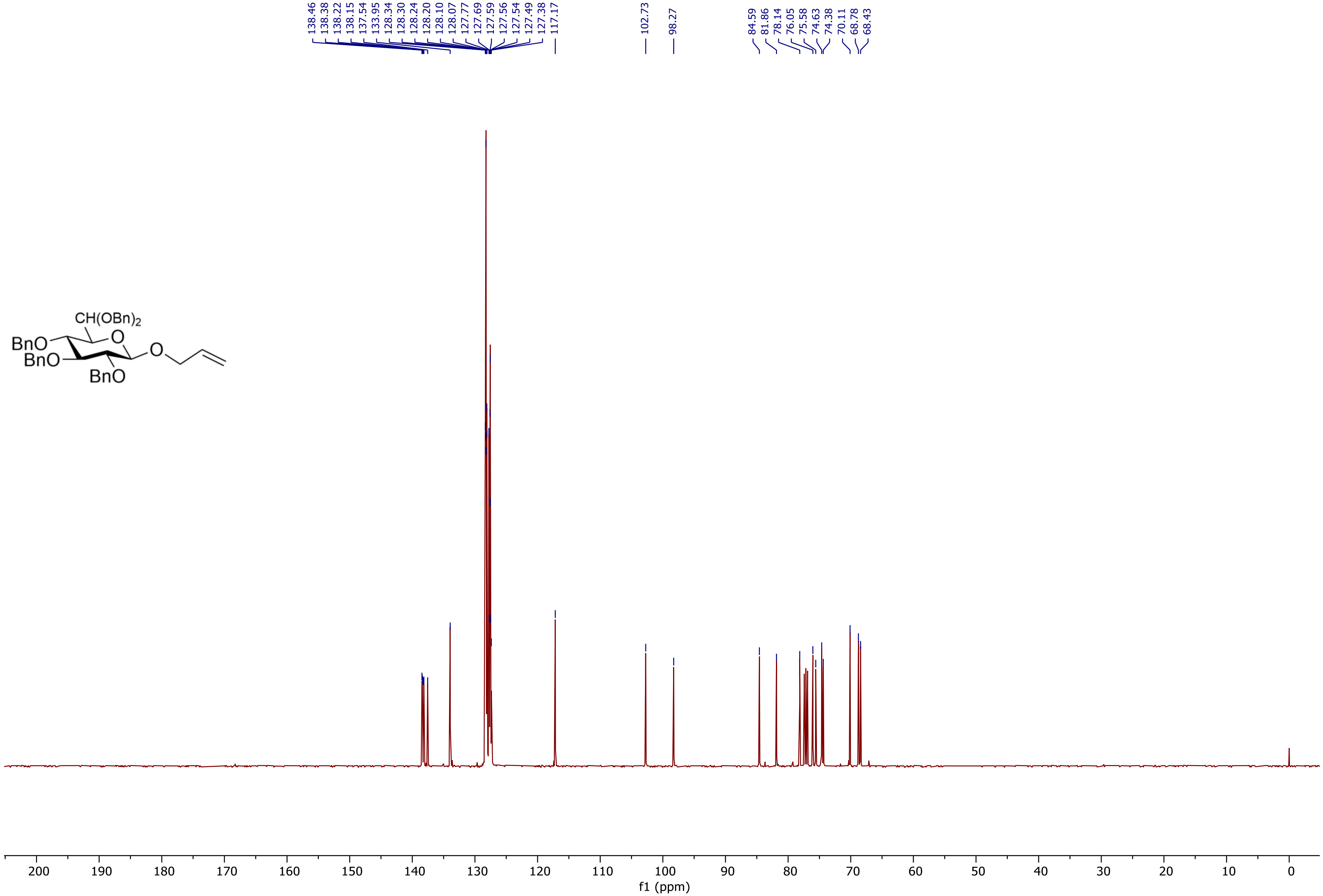

^{1}H NMR (500 MHz, CD_2Cl_2) Spectrum of Allyl (6*R*)-6-phenylthio-2,3,4,6-tetra-*O*-benzyl-*β*-D-galactopyranoside **5a**

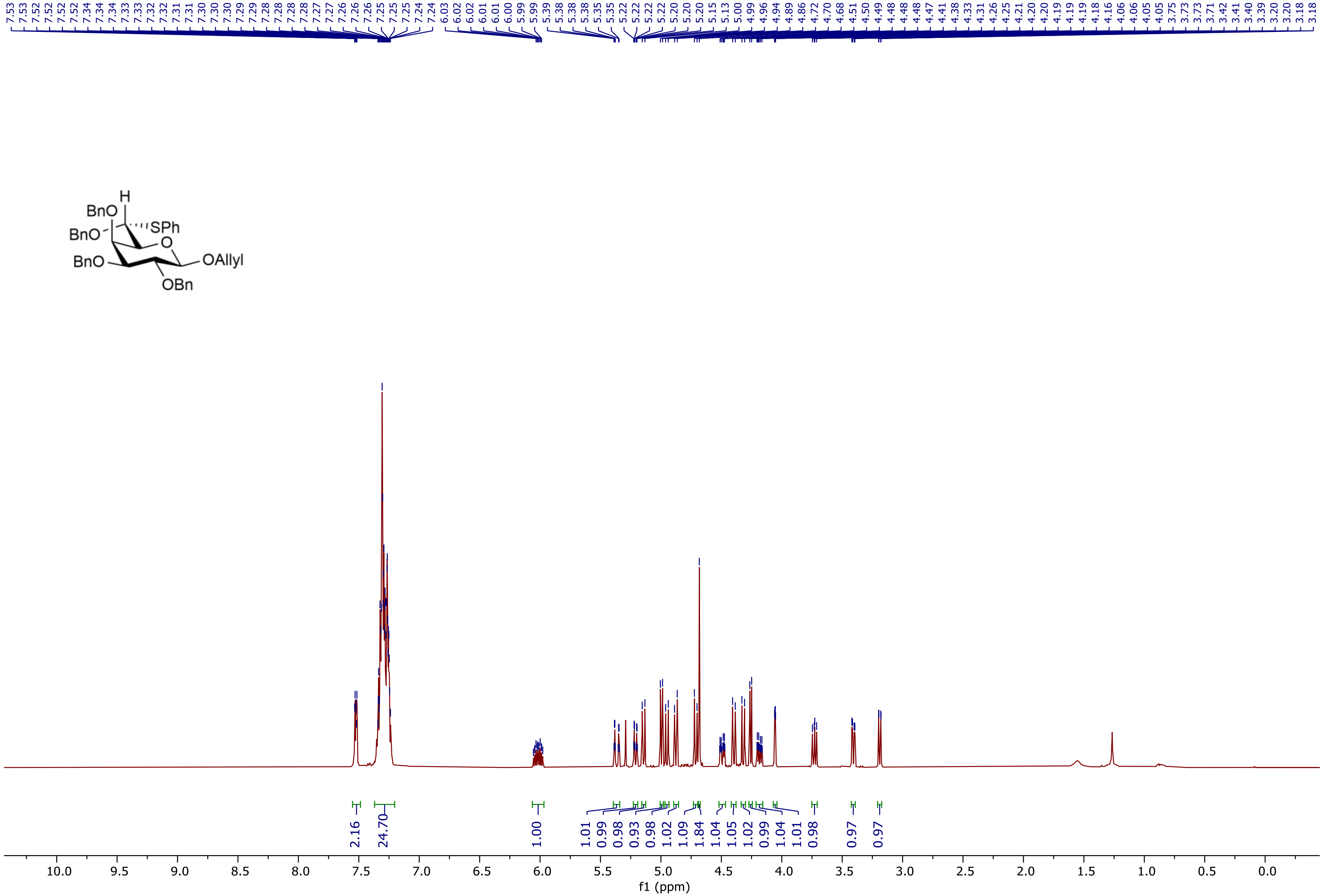

^{13}C {^{1}H} NMR (126 MHz, CD_2Cl_2) Spectrum of Allyl (6*R*)-6-phenylthio-2,3,4,6-tetra-*O*-benzyl-*β*-D-galactopyranoside **5a**

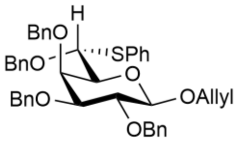

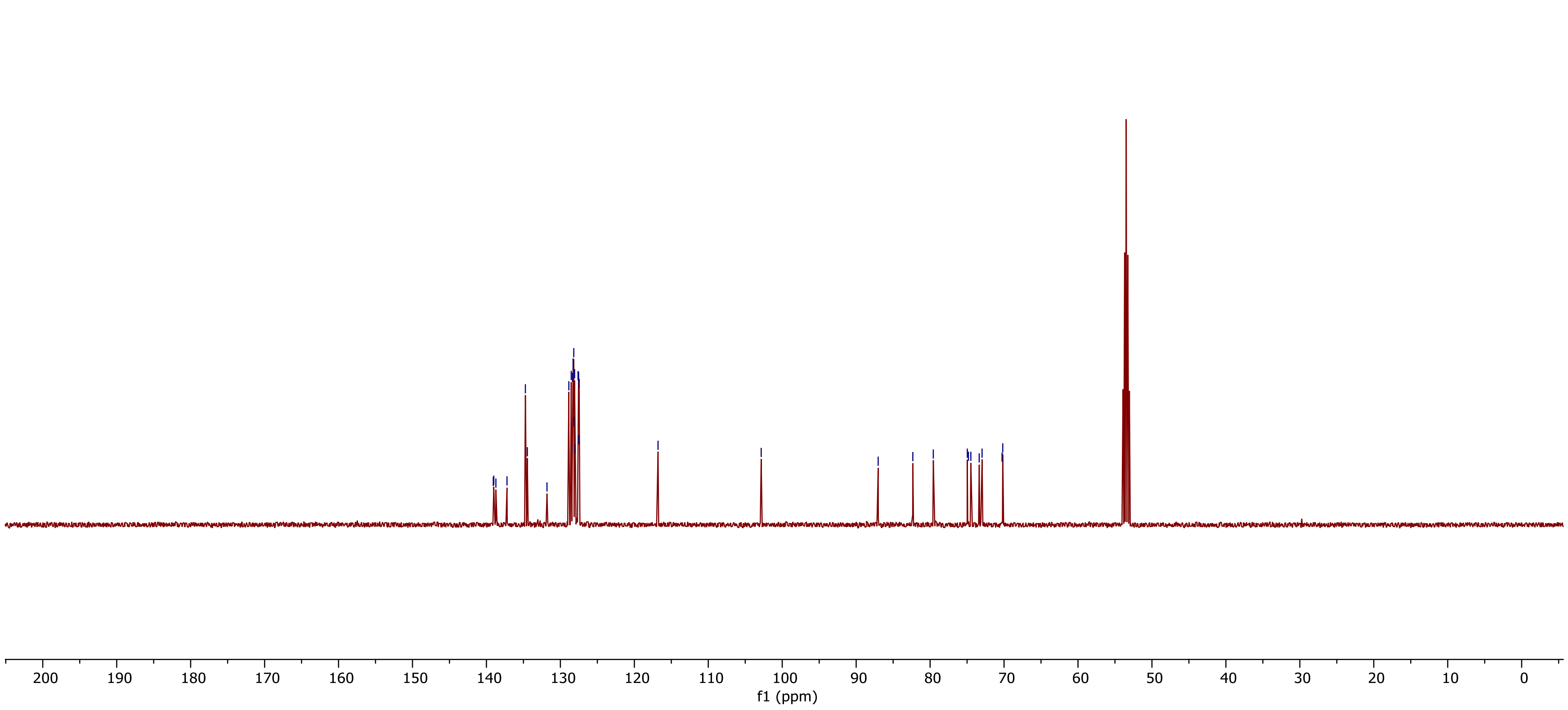

COSY Spectrum of Allyl (6*R*)-6-phenylthio-2,3,4,6-tetra-*O*-benzyl-*β*-D-galactopyranoside **5a**

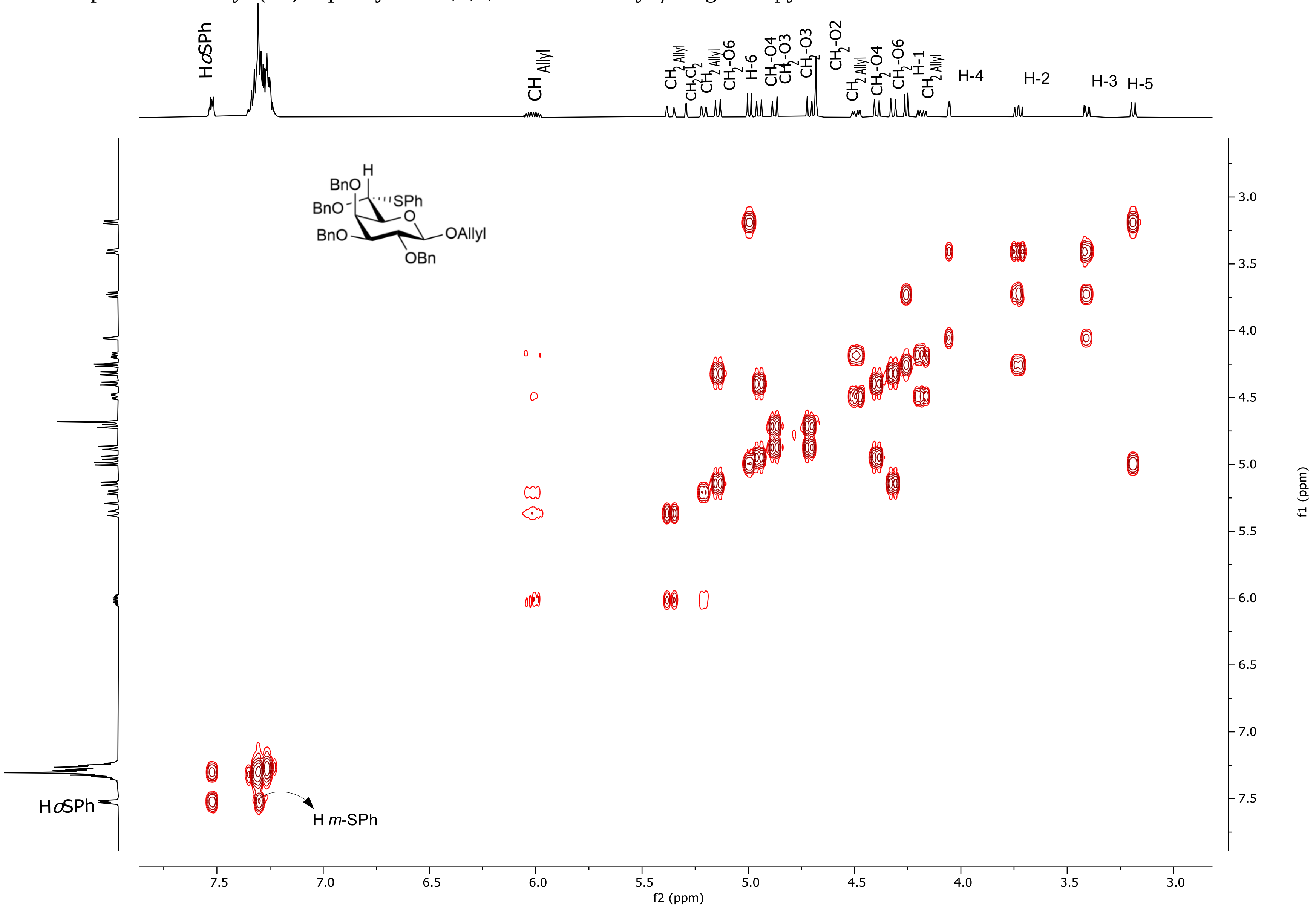

HSQC Spectrum of Allyl (6*R*)-6-phenylthio-2,3,4,6-tetra-*O*-benzyl-*β*-D-galactopyranoside **5a**

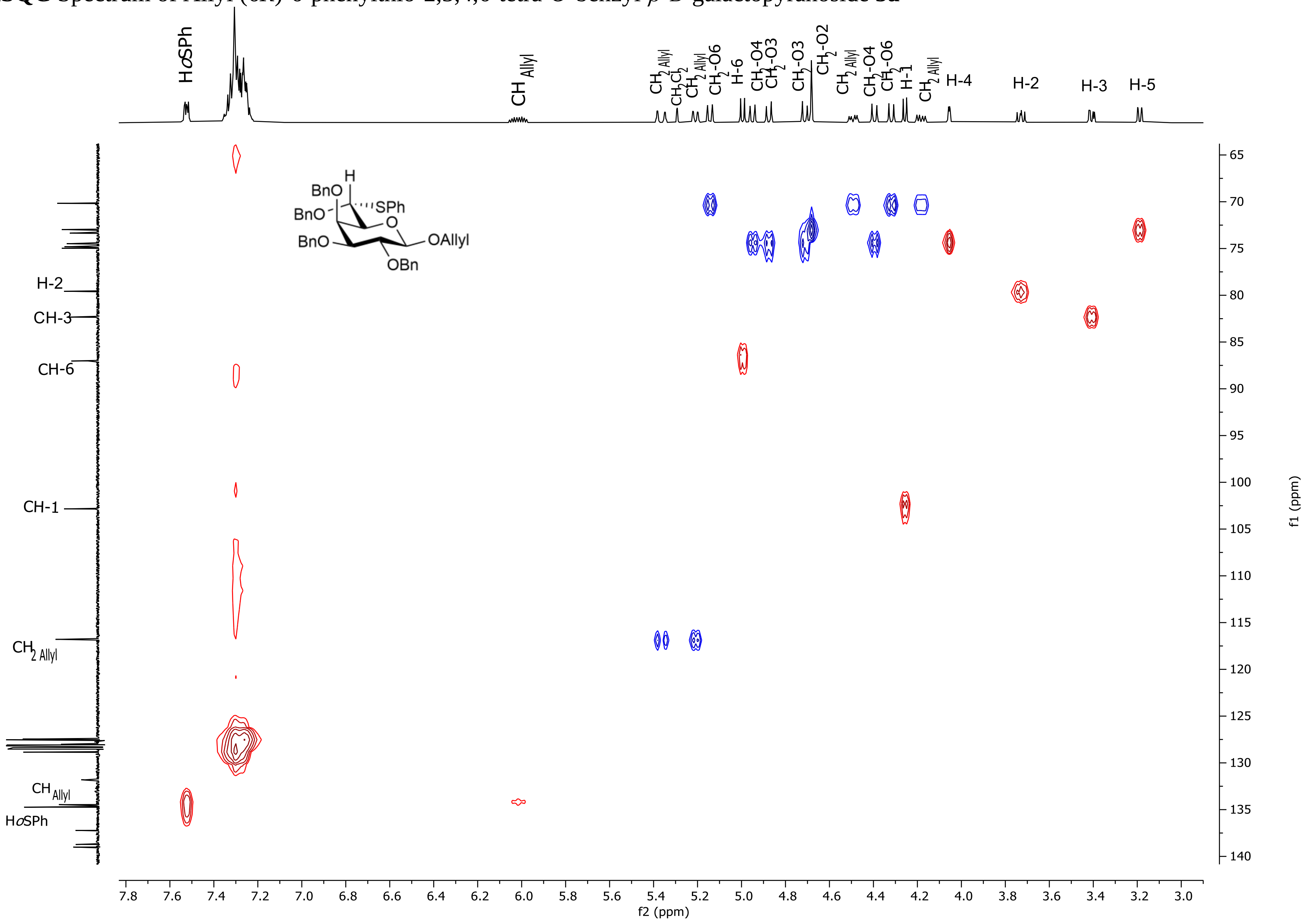

HMBC Spectrum of Allyl (6*R*)-6-phenylthio-2,3,4,6-tetra-*O*-benzyl-*β*-D-galactopyranoside **5a**

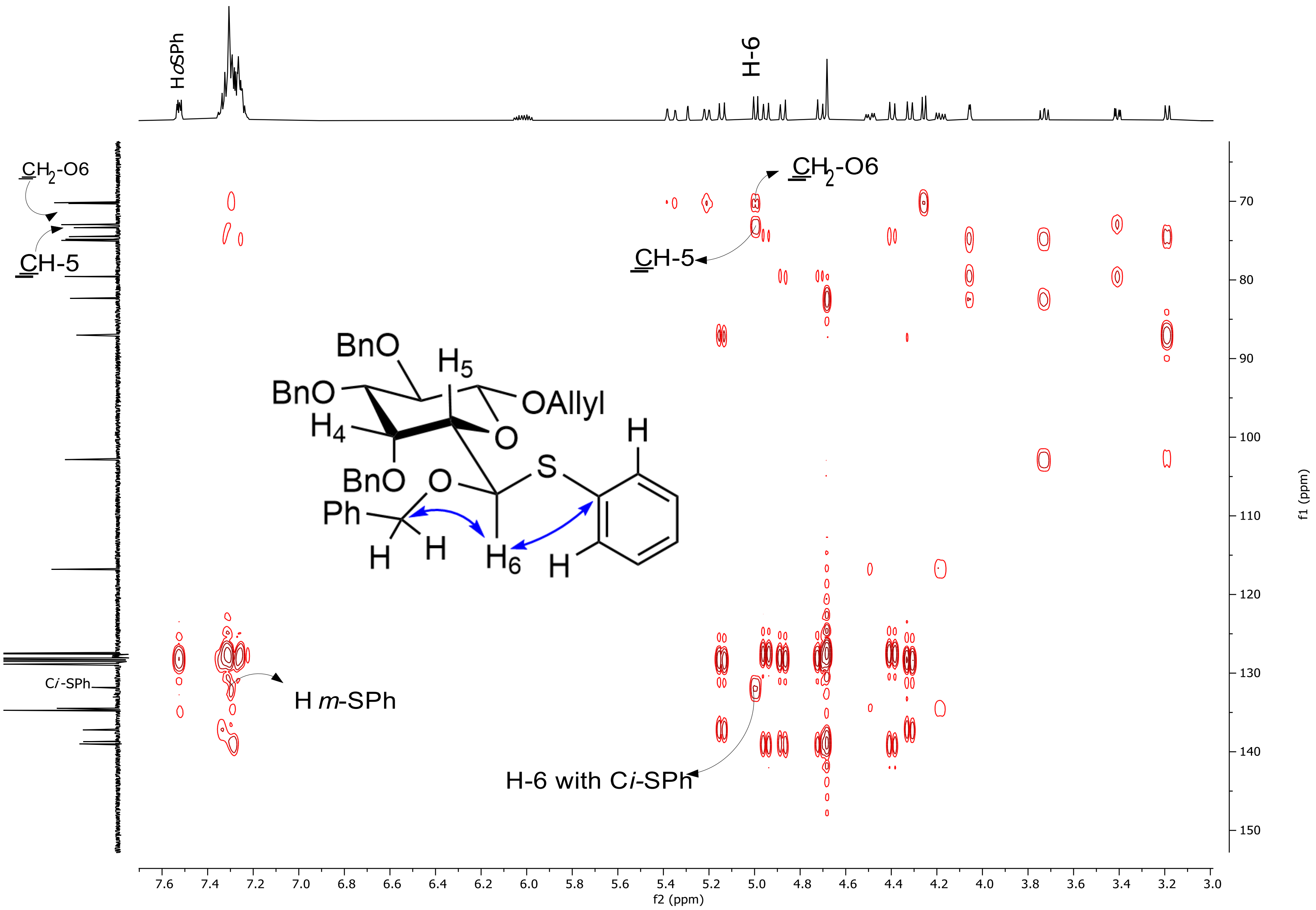

NOESY Spectrum of Allyl (6*R*)-6-phenylthio-2,3,4,6-tetra-*O*-benzyl-*β*-D-galactopyranoside **5a**

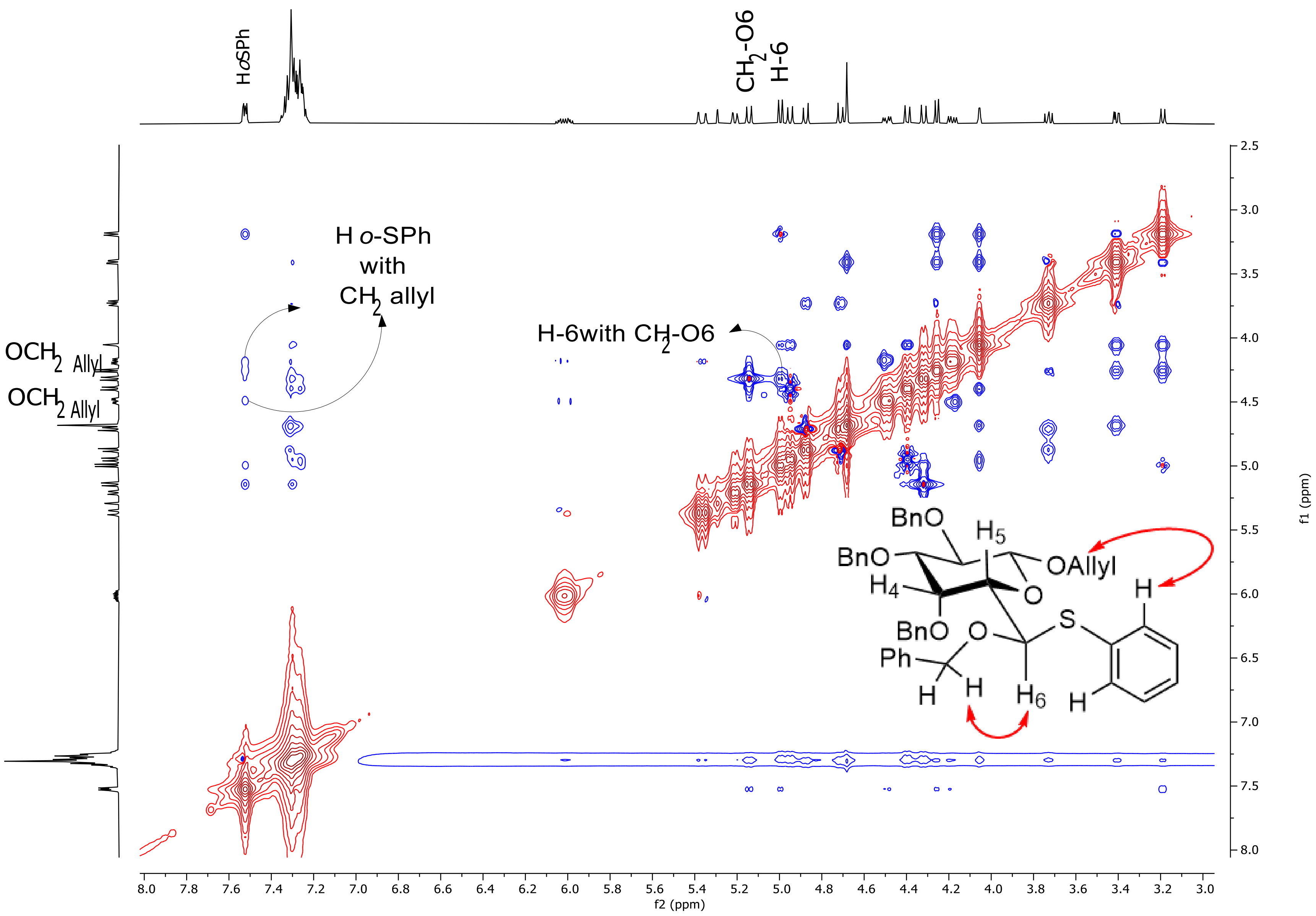

^{1}H NMR (500 MHz, C_6D_6) Spectrum of Allyl (6*S*)-6-phenylthio-2,3,4,6-tetra-*O*-benzyl-*β*-D-galactopyranoside **(5b)**

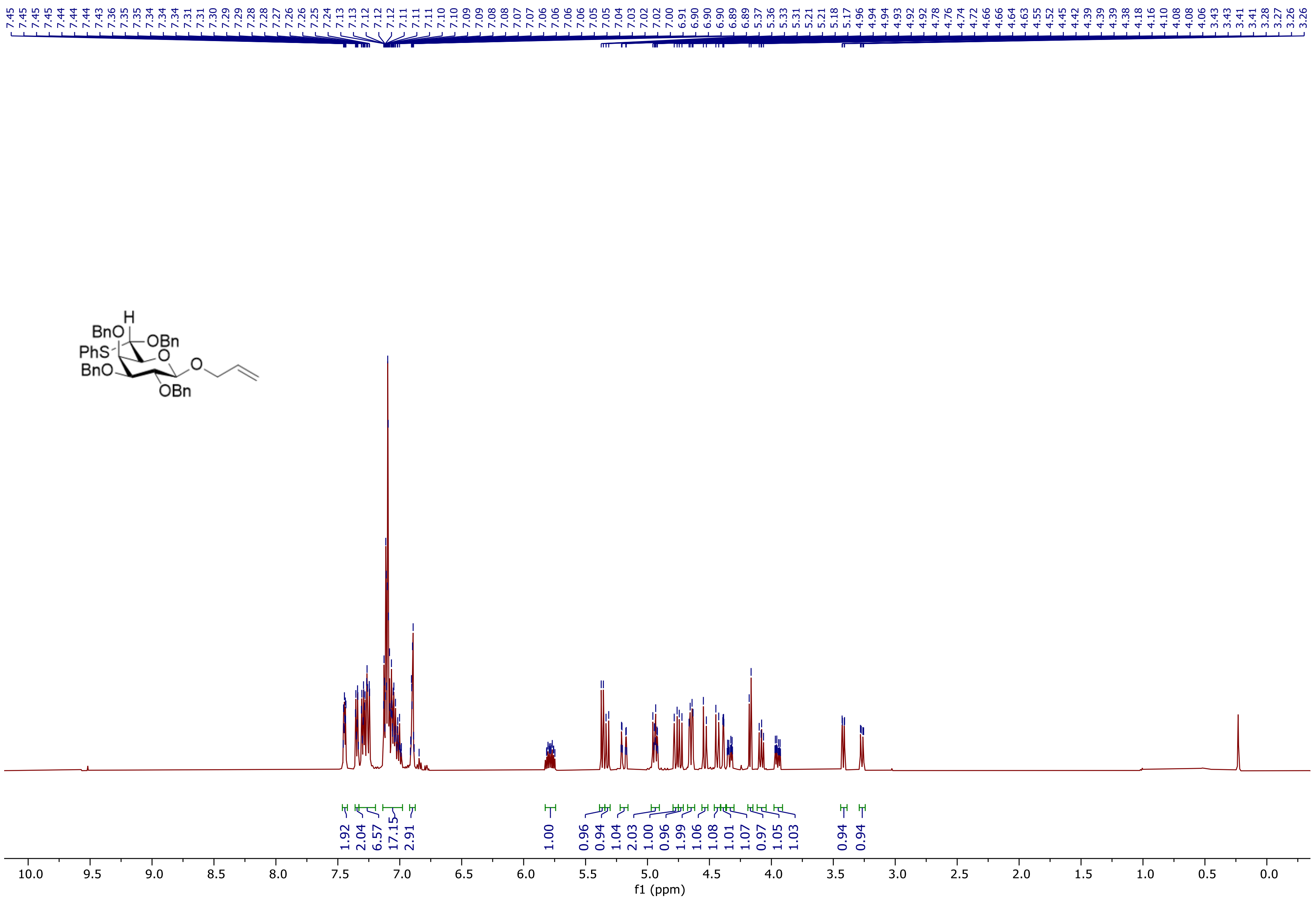

^{13}C{^{1}H} NMR (126 MHz, C_6D_6) Spectrum of Allyl (6*S*)-6-phenylthio-2,3,4,6-tetra-*O*-benzyl-*β*-D-galactopyranoside **(5b)**

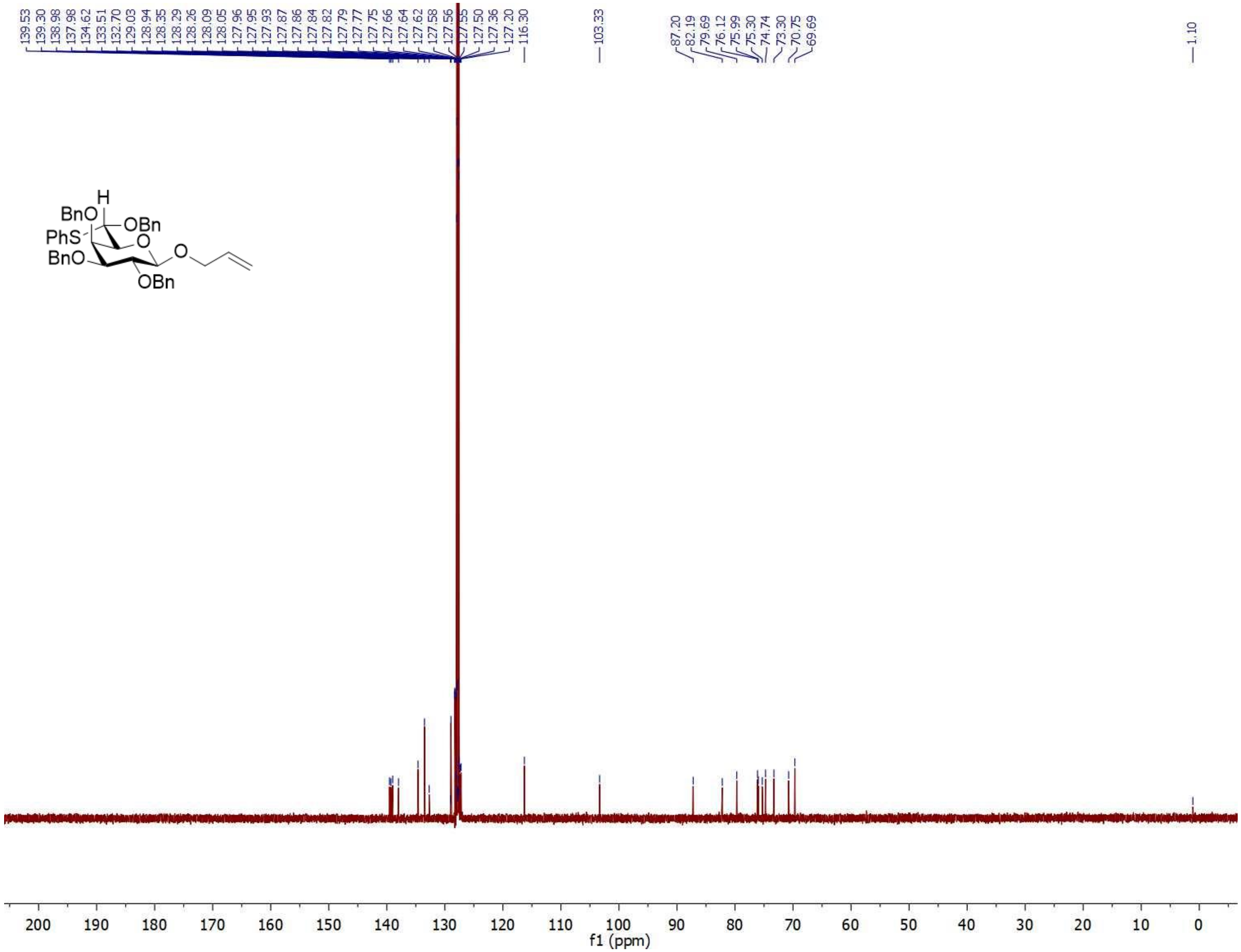

COSY (500 MHz, C_6D_6) Spectrum of Allyl (6*S*)-6-phenylthio-2,3,4,6-tetra-*O*-benzyl-*β*-D-galactopyranoside **(5b)**

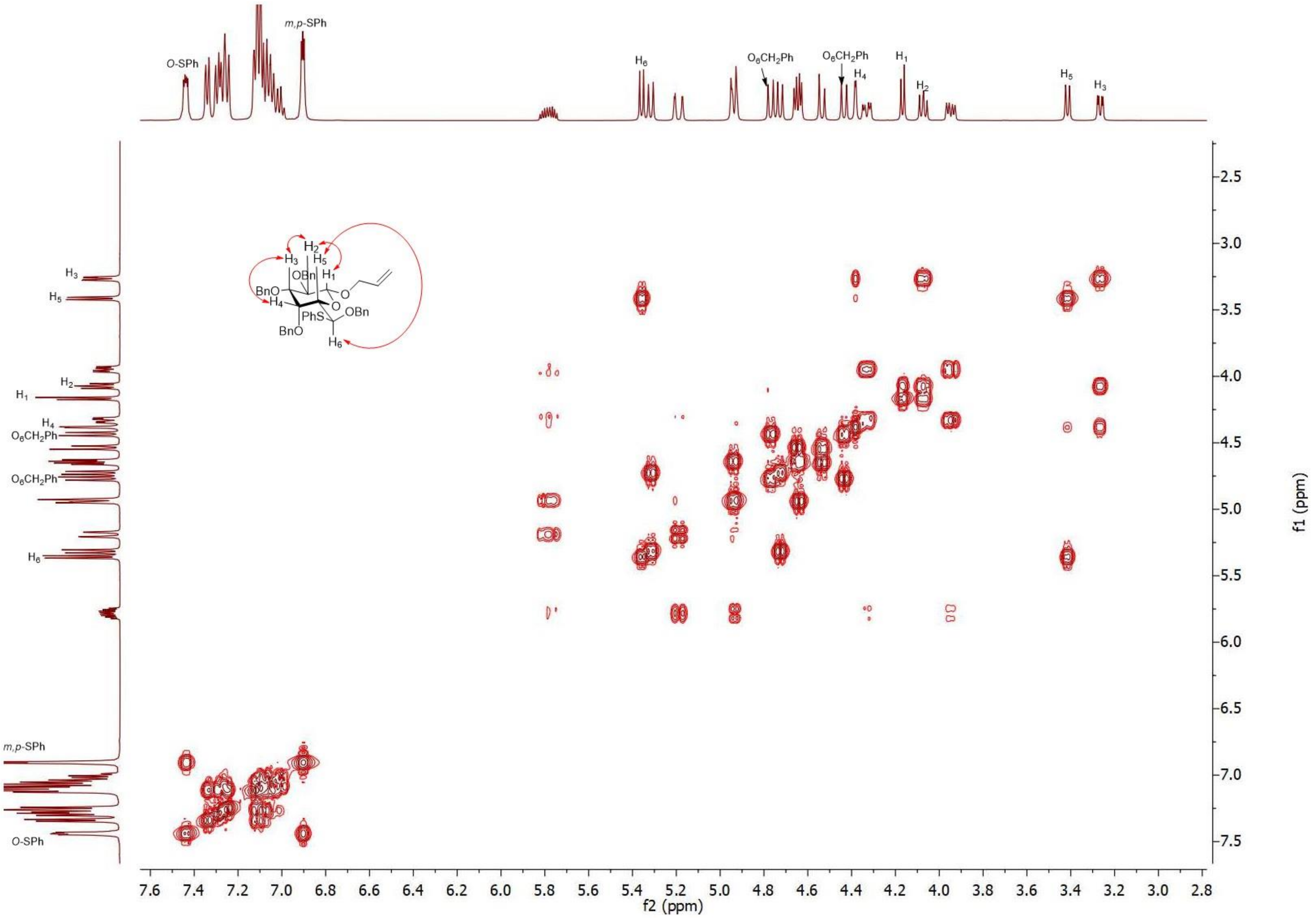

HSQC (500 MHz, C_6D_6) Spectrum of Allyl (6*S*)-6-phenylthio-2,3,4,6-tetra-*O*-benzyl-*β*-D-galactopyranoside **(5b)**

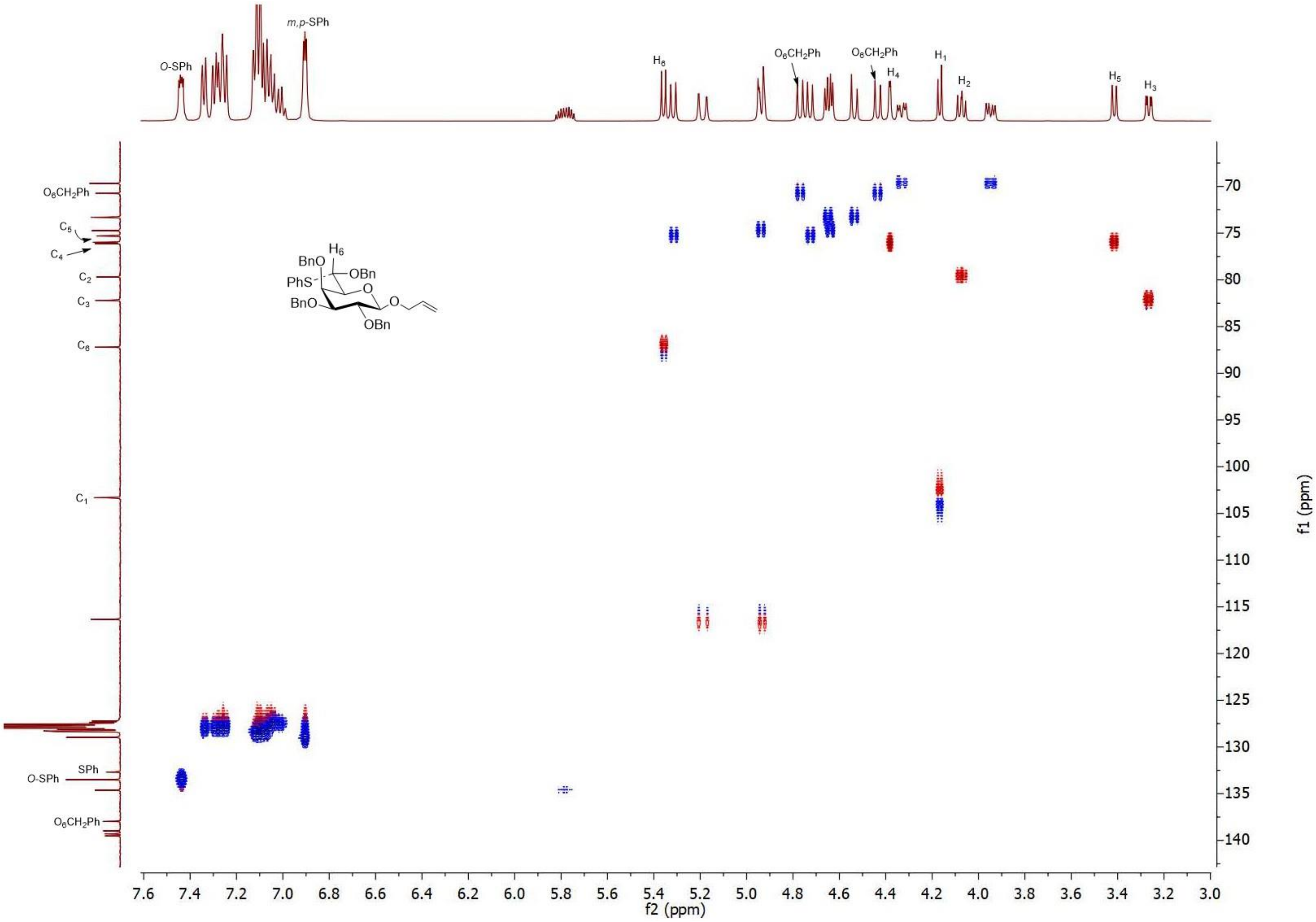

HMBC (500 MHz, C_6D_6) Spectrum of Allyl (6*S*)-6-phenylthio-2,3,4,6-tetra-*O*-benzyl-*β*-D-galactopyranoside **(5b)**

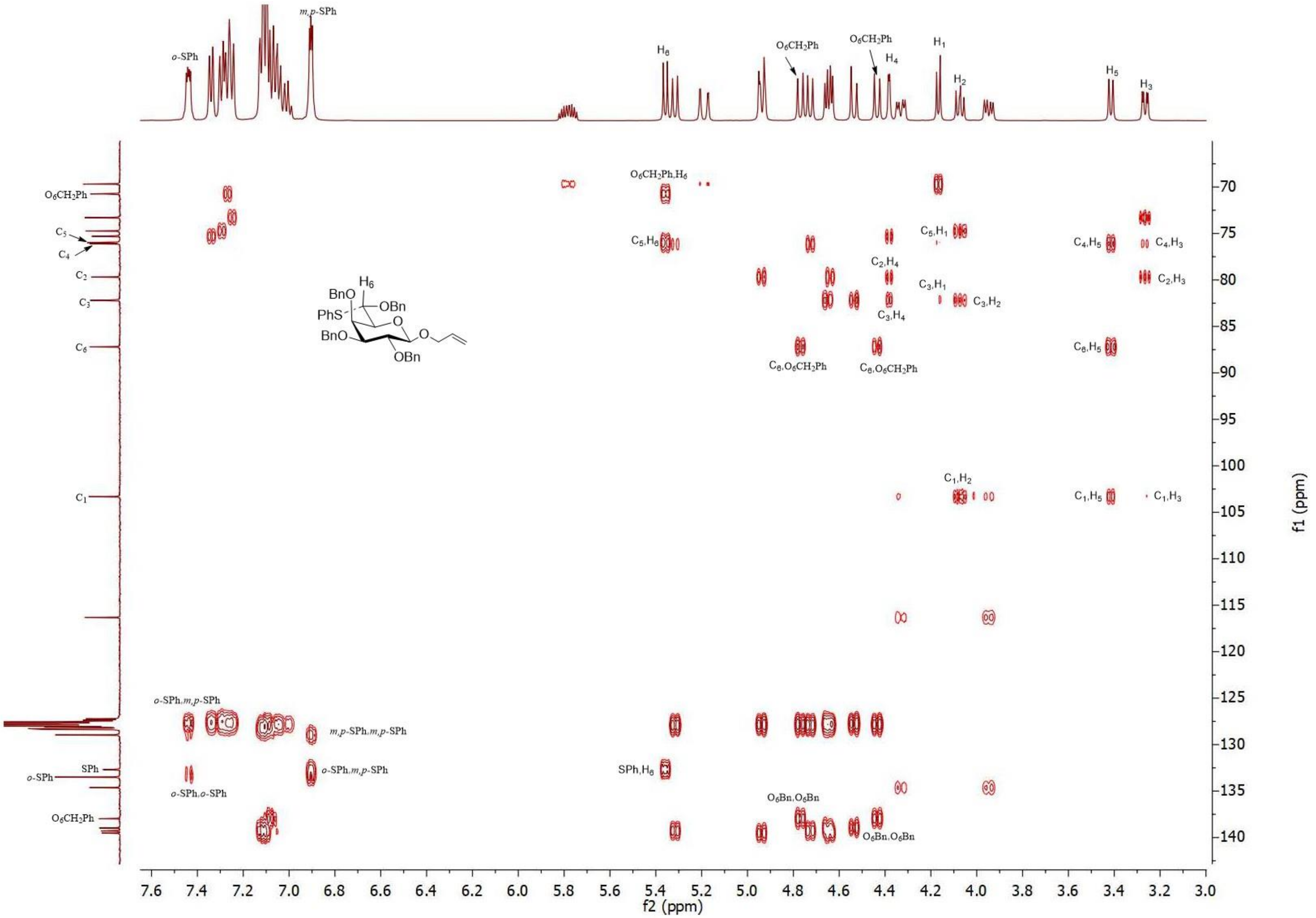

NOESY (500 MHz**,** C_6D_6) Spectrum of Allyl (6*S*)-6-phenylthio-2,3,4,6-tetra-*O*-benzyl-*β*-D-galactopyranoside (5b)

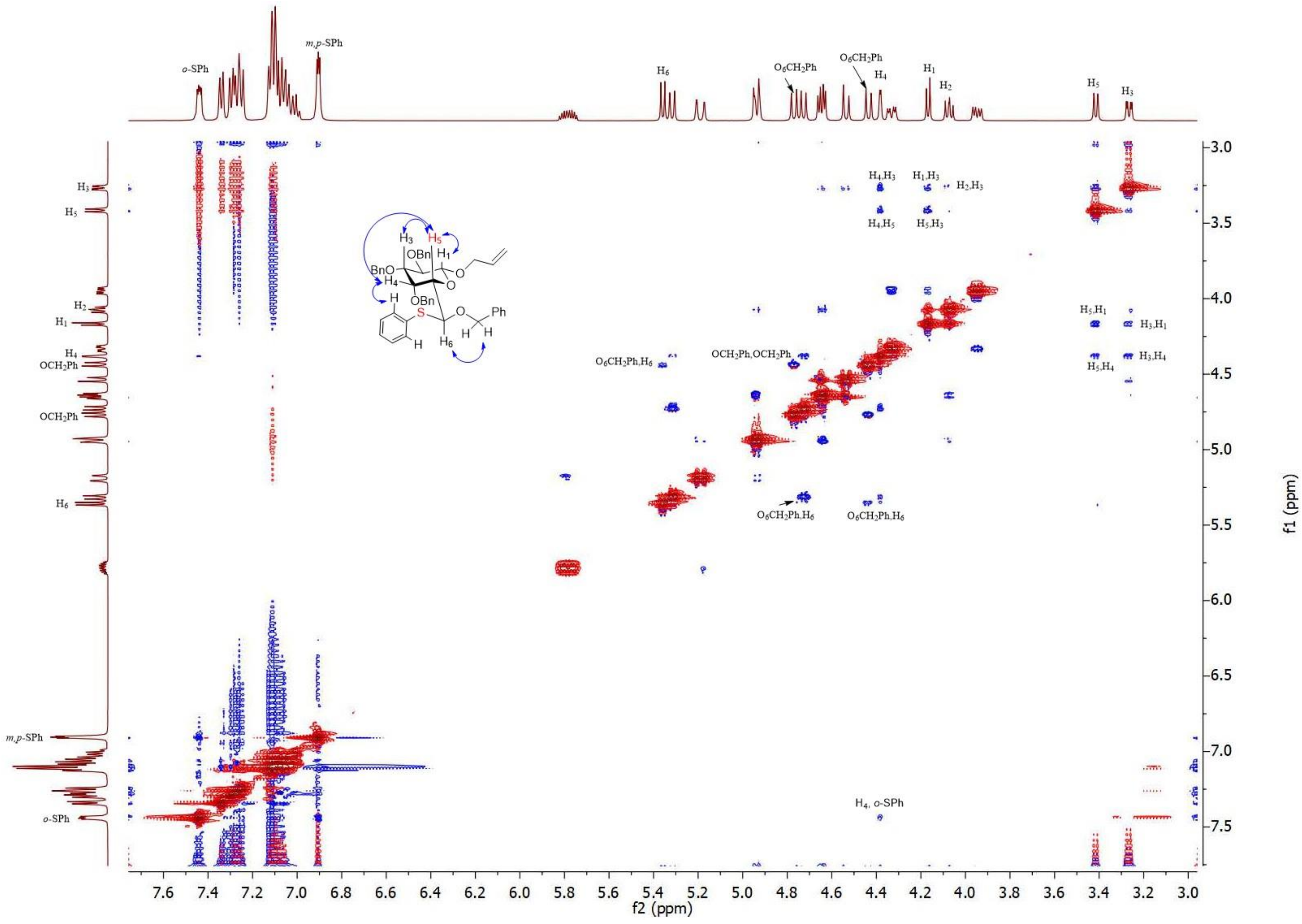

^{1}H NMR (500 MHz, C_6D_6) Spectrum of Allyl (6*S*)-6-phenylthio-2,3,4,6-tetra-*O*-benzyl-*β*-D-glucopyranoside. **5c**

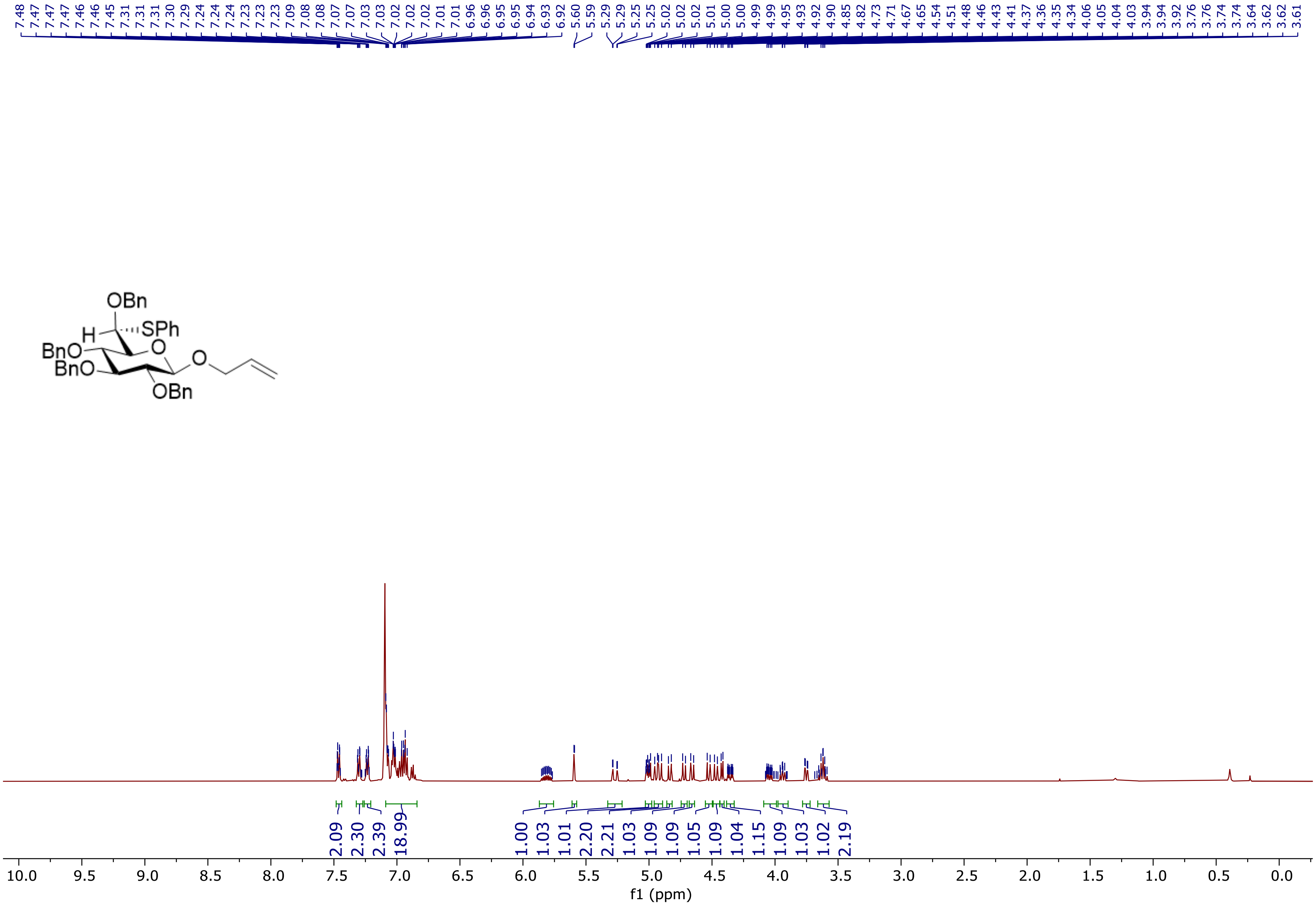

^{13}C{^{1}H} NMR (126 MHz, C_6D_6) Spectrum of Allyl (6*S*)-6-phenylthio-2,3,4,6-tetra-*O*-benzyl-*β*-D-glucopyranoside. **5c**

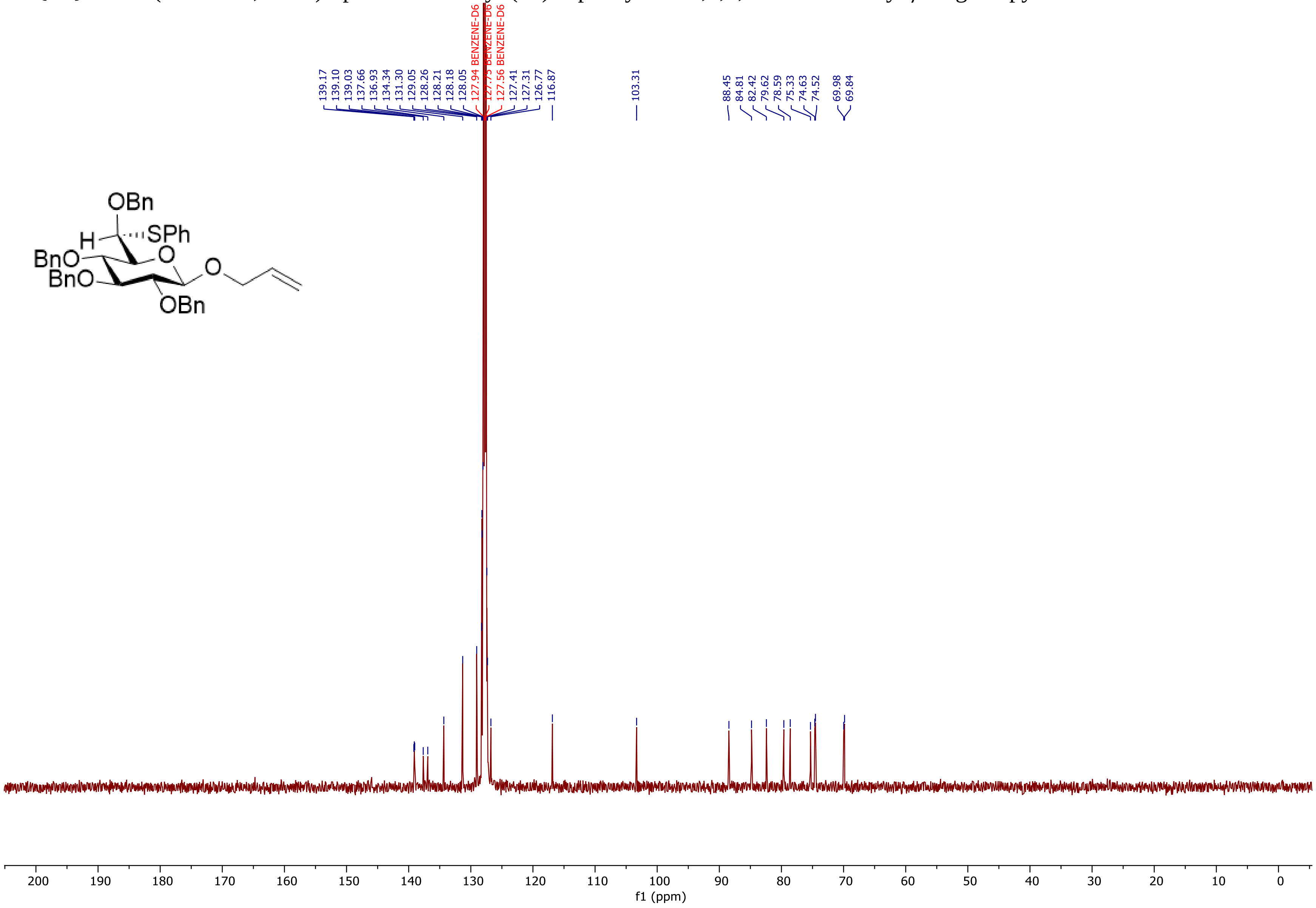

HSQC Spectrum of Allyl (6*S*)-6-phenylthio-2,3,4,6-tetra-*O*-benzyl-*β*-D-glucopyranoside. **5c**

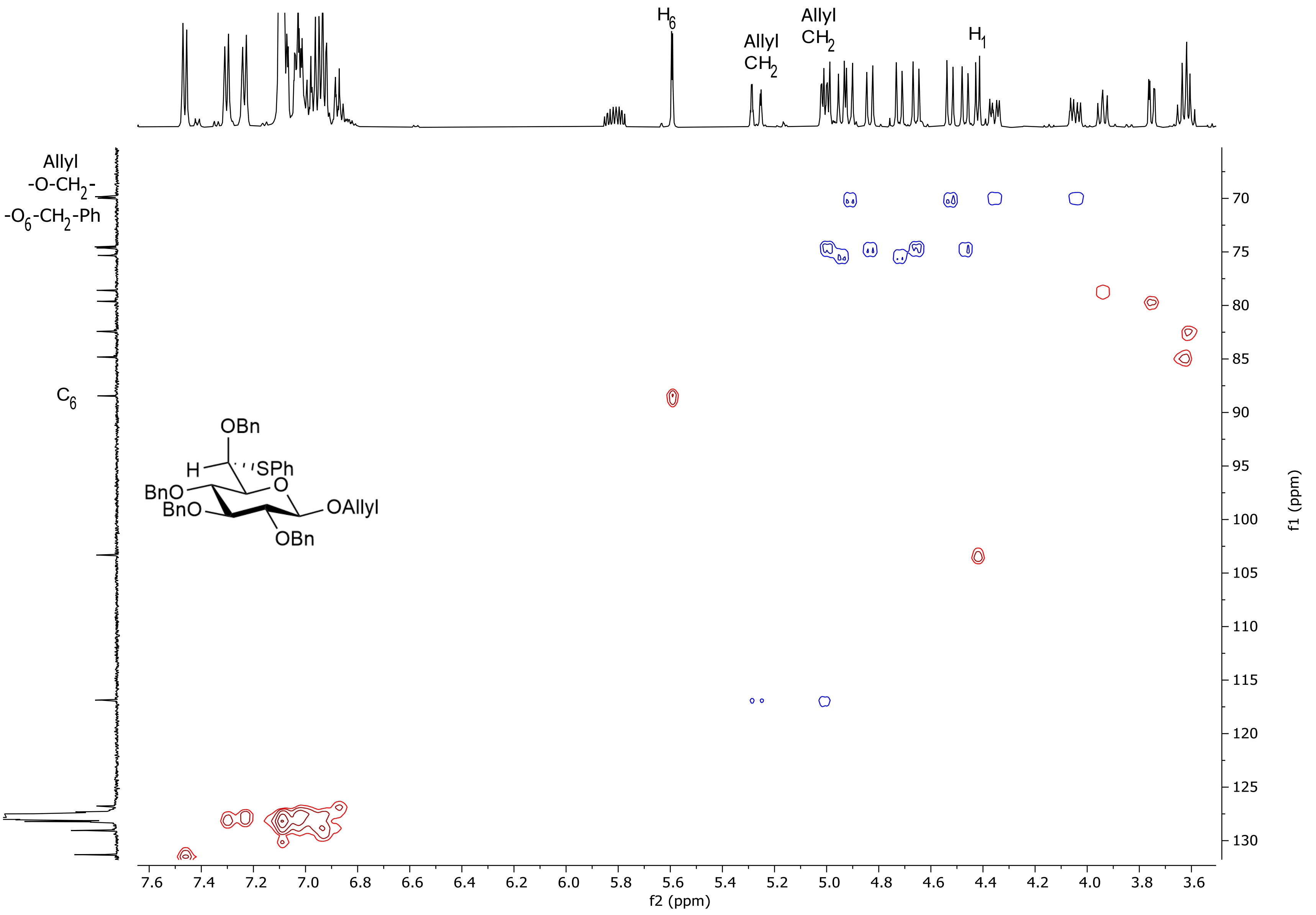

COSY Spectrum of Allyl (6*S*)-6-phenylthio-2,3,4,6-tetra-*O*-benzyl-*β*-D-glucopyranoside. **5c**

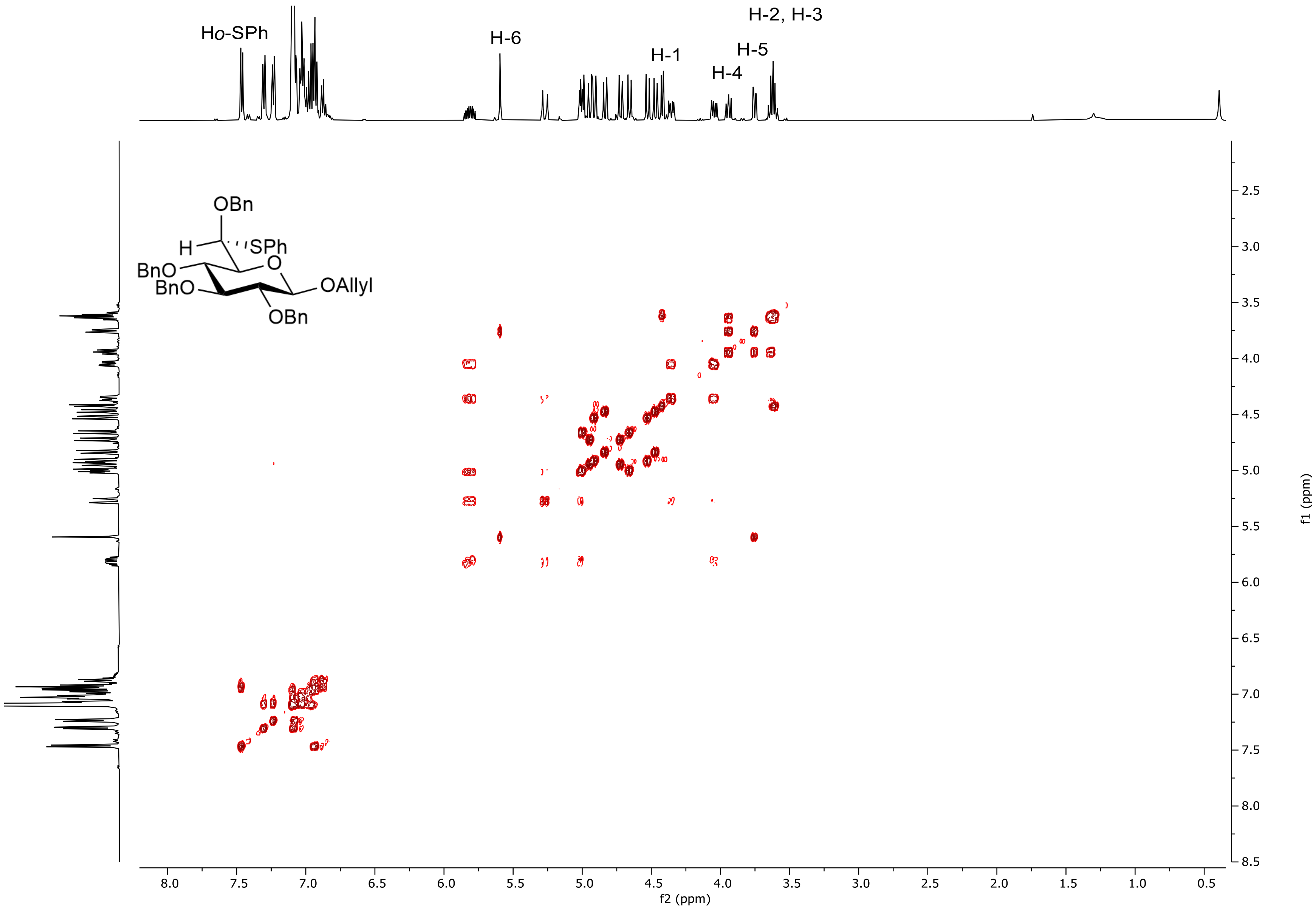

HMBC Spectrum of Allyl (6*S*)-6-phenylthio-2,3,4,6-tetra-*O*-benzyl-*β*-D-glucopyranoside. **5c**

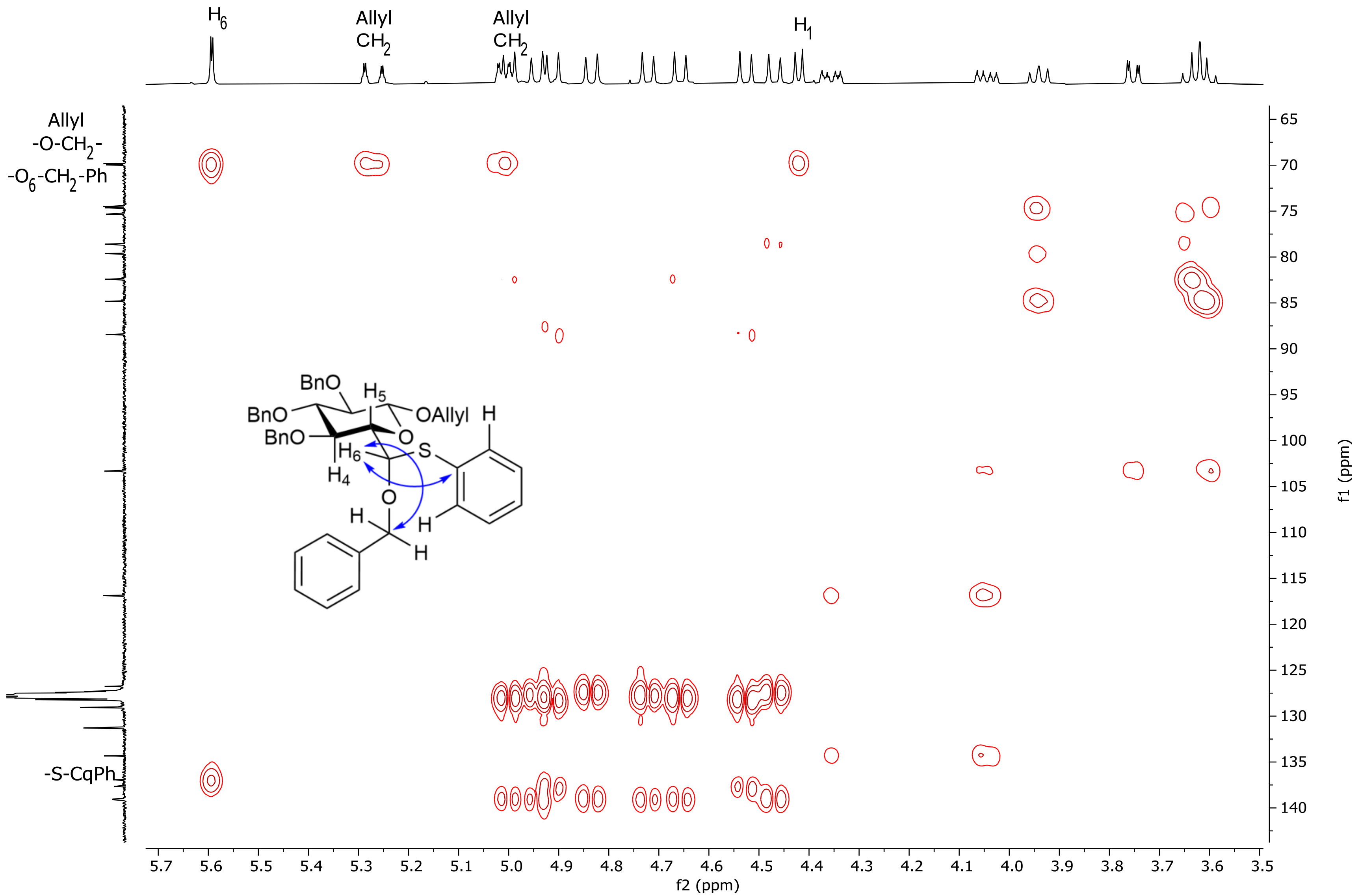

NOESY Spectrum of Allyl (6*S*)-6-phenylthio-2,3,4,6-tetra-*O*-benzyl-*β*-D-glucopyranoside. **5c**

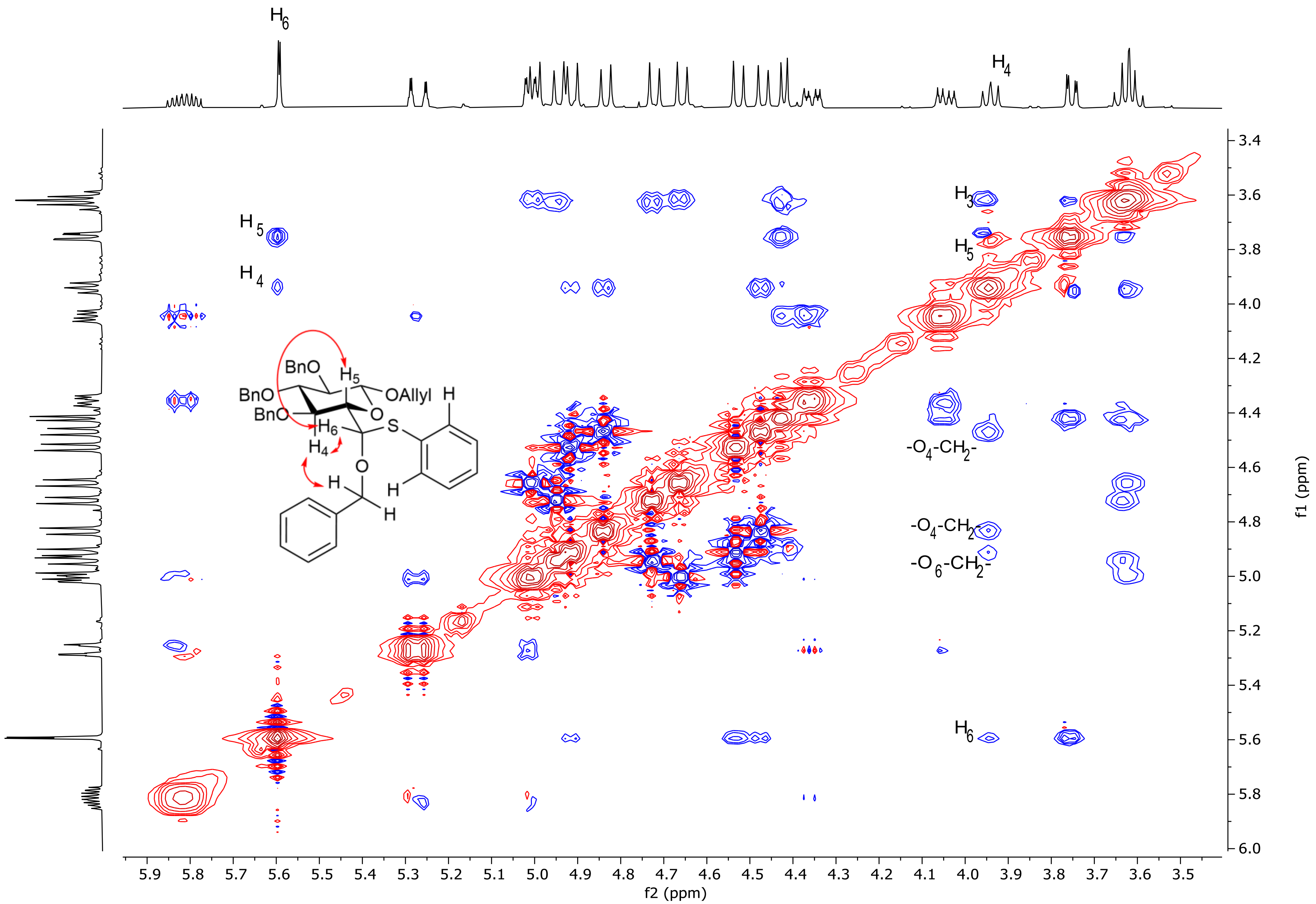

^{1}H NMR (500 MHz, C_6D_6) Spectrum of Allyl (6*R*)-6-phenylthio-2,3,4,6-tetra-*O*-benzyl-*β*-D-glucopyranoside. **5d**

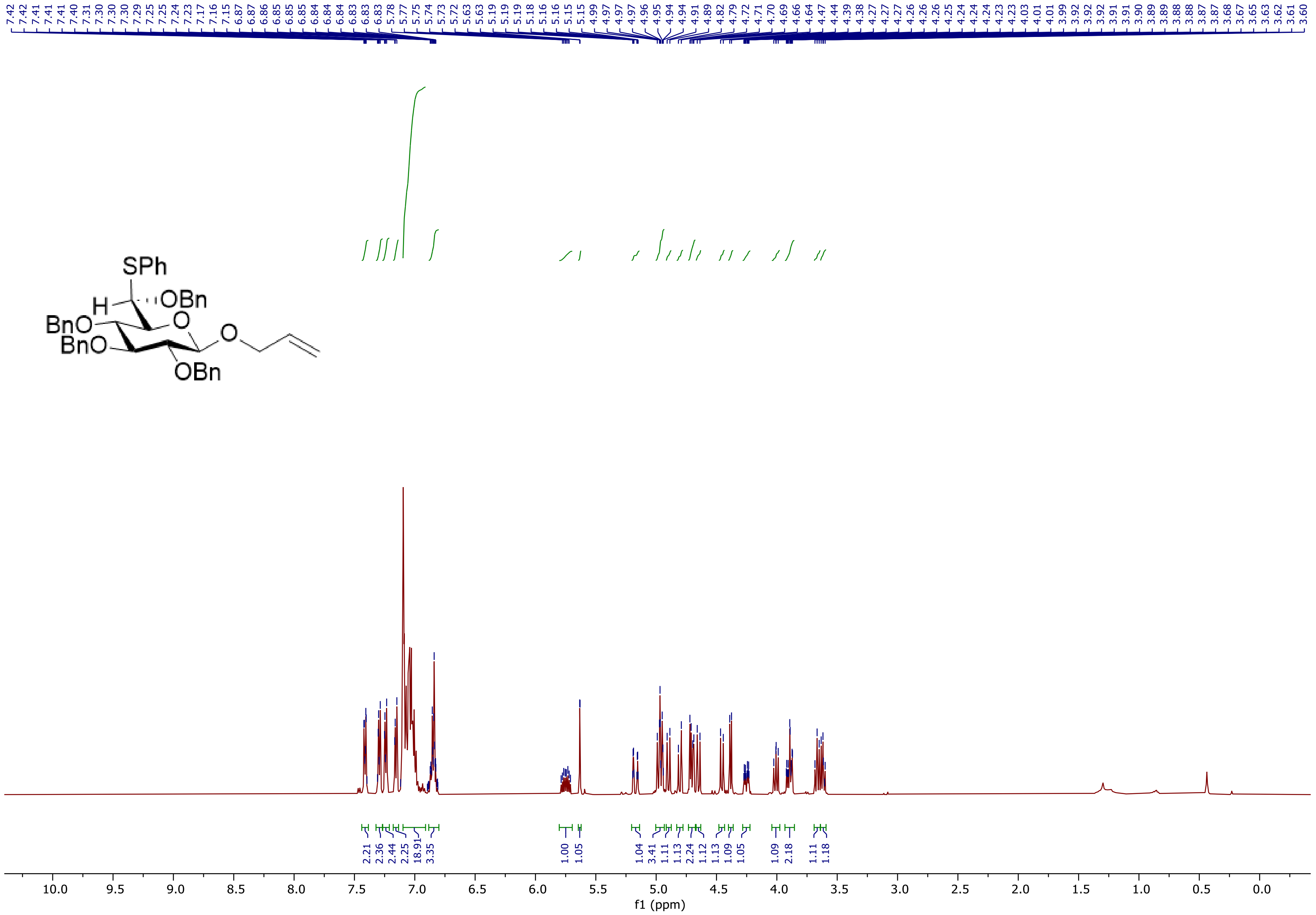

$^{13}C\{^{1}H\}$ NMR (126 MHz, C_6D_6) Spectrum of Allyl (6*R*)-6-phenylthio-2,3,4,6-tetra-*O*-benzyl-*β*-D-glucopyranoside. **5d**

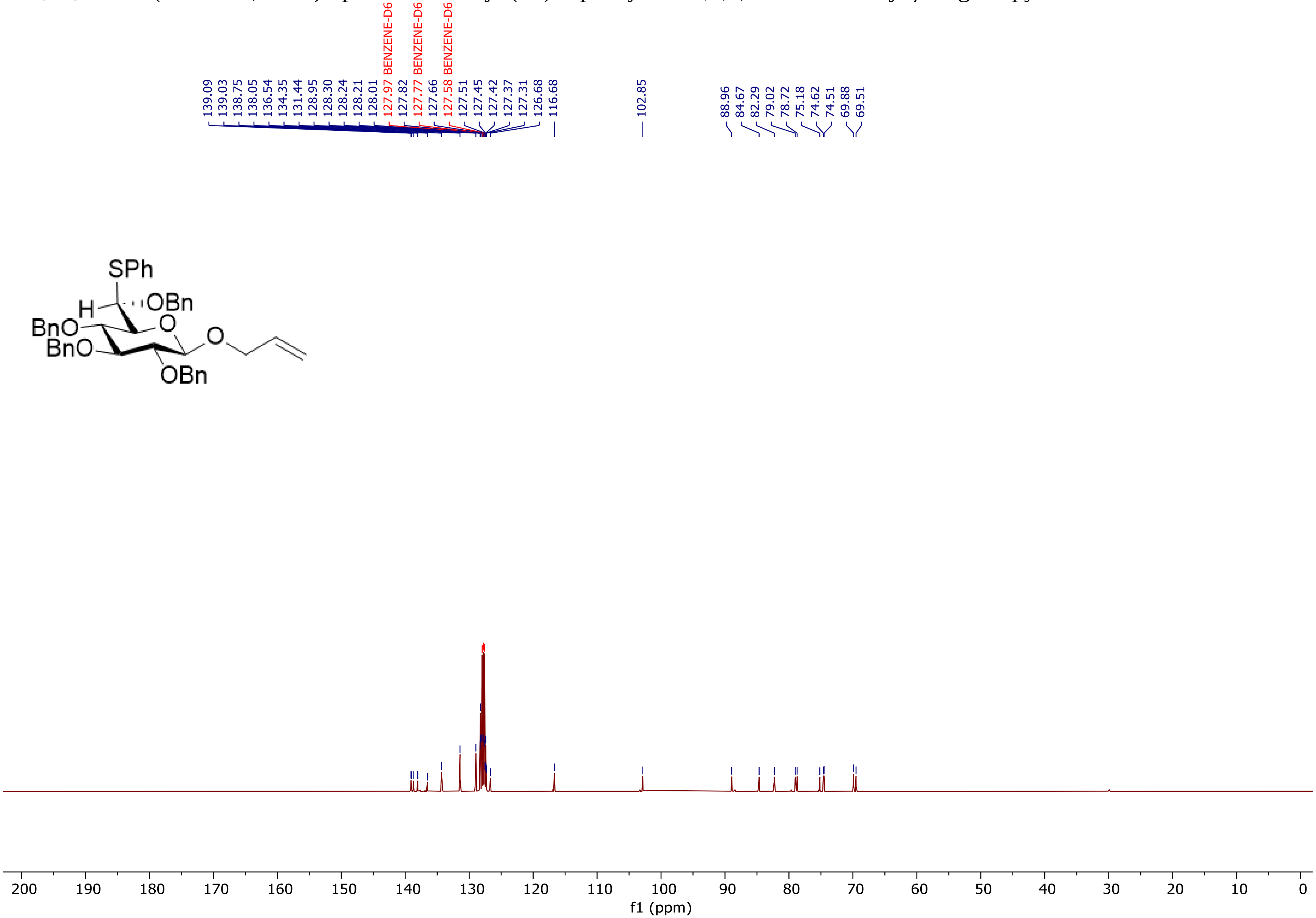

COSY Spectrum of Allyl (6*R*)-6-phenylthio-2,3,4,6-tetra-*O*-benzyl-*β*-D-glucopyranoside. **5d**

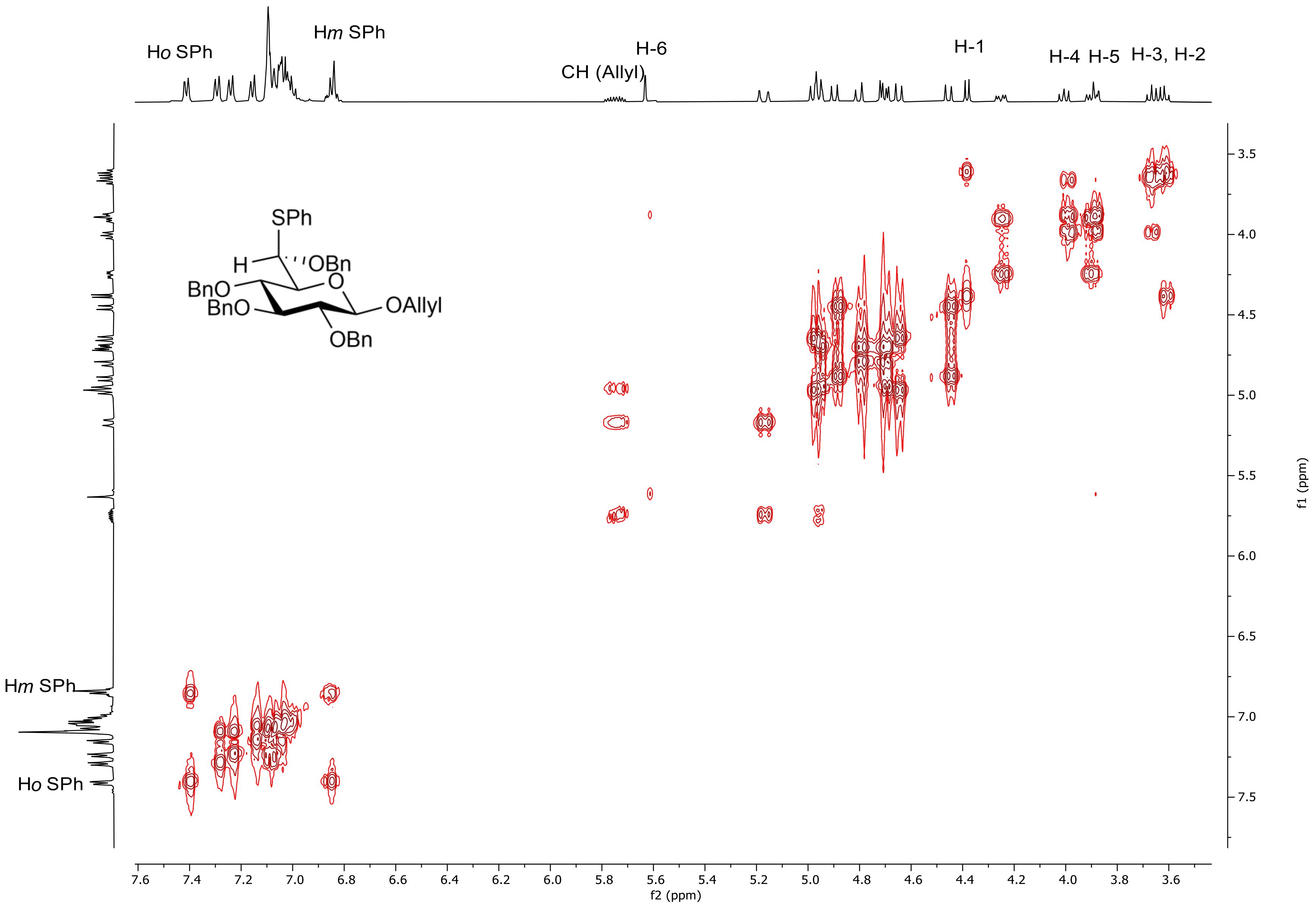

HSQC Spectrum of Allyl (6*R*)-6-phenylthio-2,3,4,6-tetra-*O*-benzyl-*β*-D-glucopyranoside. **5d**

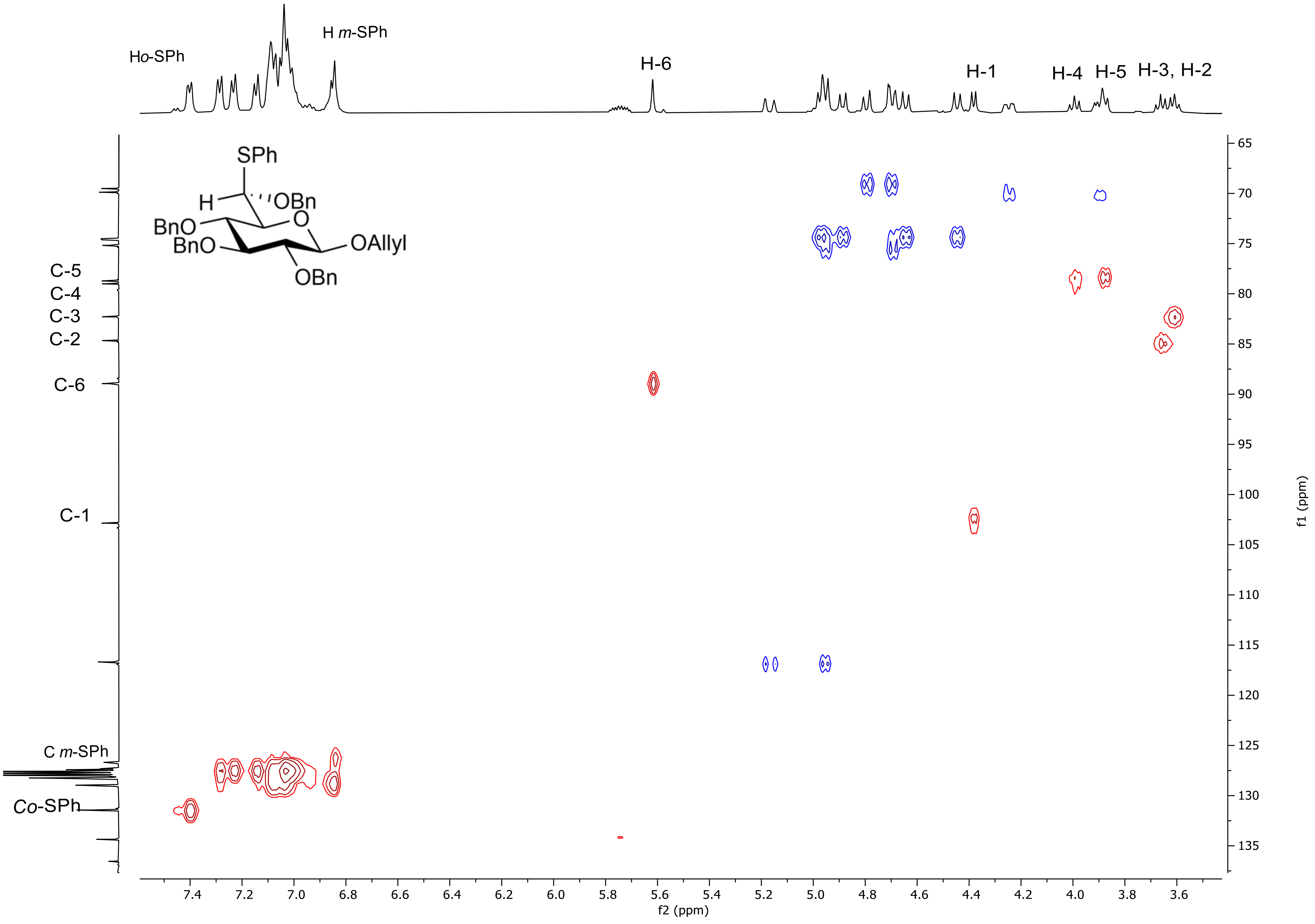

HMBC Spectrum of Allyl (6*R*)-6-phenylthio-2,3,4,6-tetra-*O*-benzyl-*β*-D-glucopyranoside. **5d**

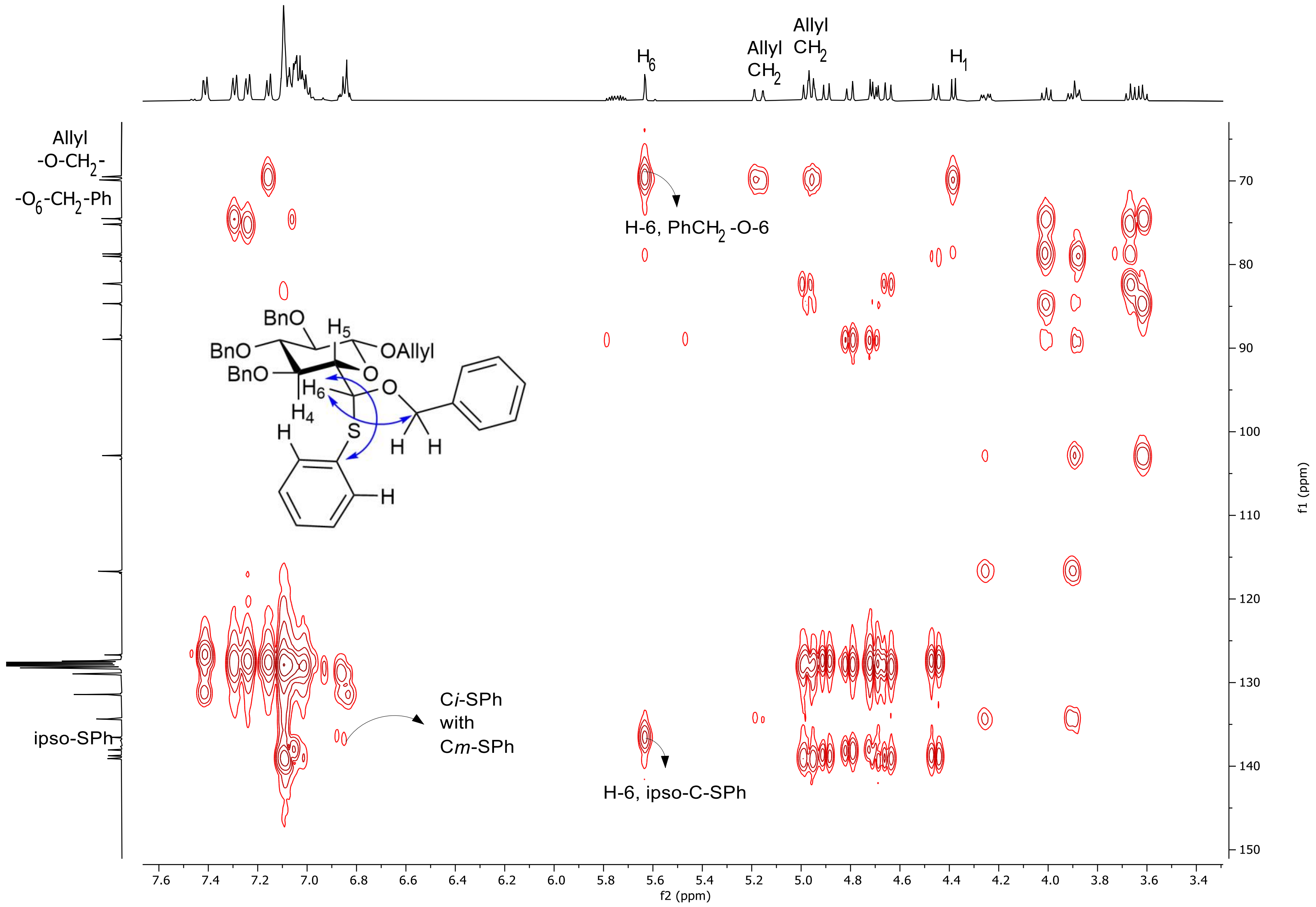

NOESY Spectrum of Allyl (6*R*)-6-phenylthio-2,3,4,6-tetra-*O*-benzyl-*β*-D-glucopyranoside. **5d**

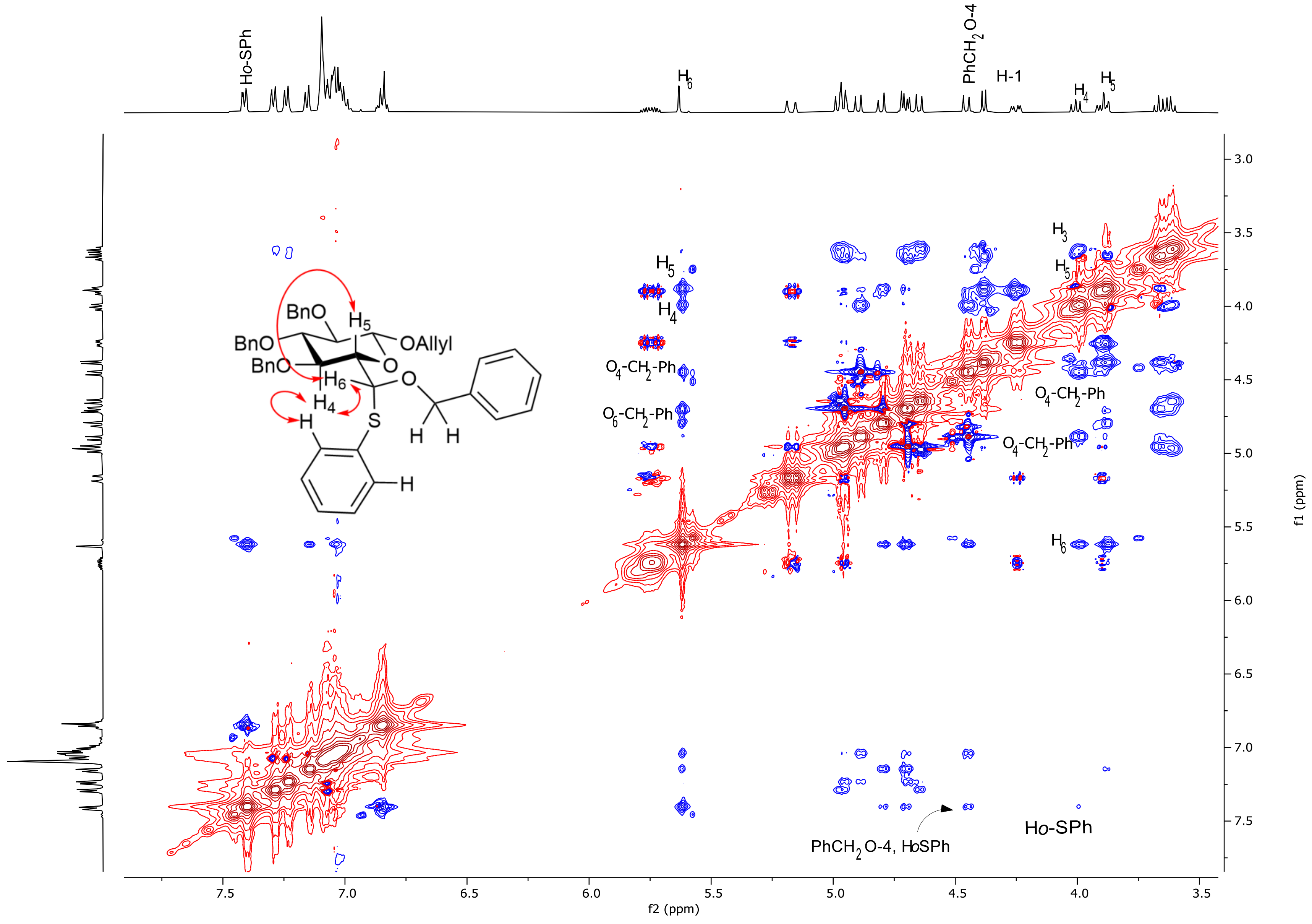

^{1}H NMR (500 MHz, $CDCl_3$) Spectrum of (6*R*)-6-phenylthio-2,3,4,6-tetra-*O*-benzyl-α/*β*-D-galactopyranose **(6a)**

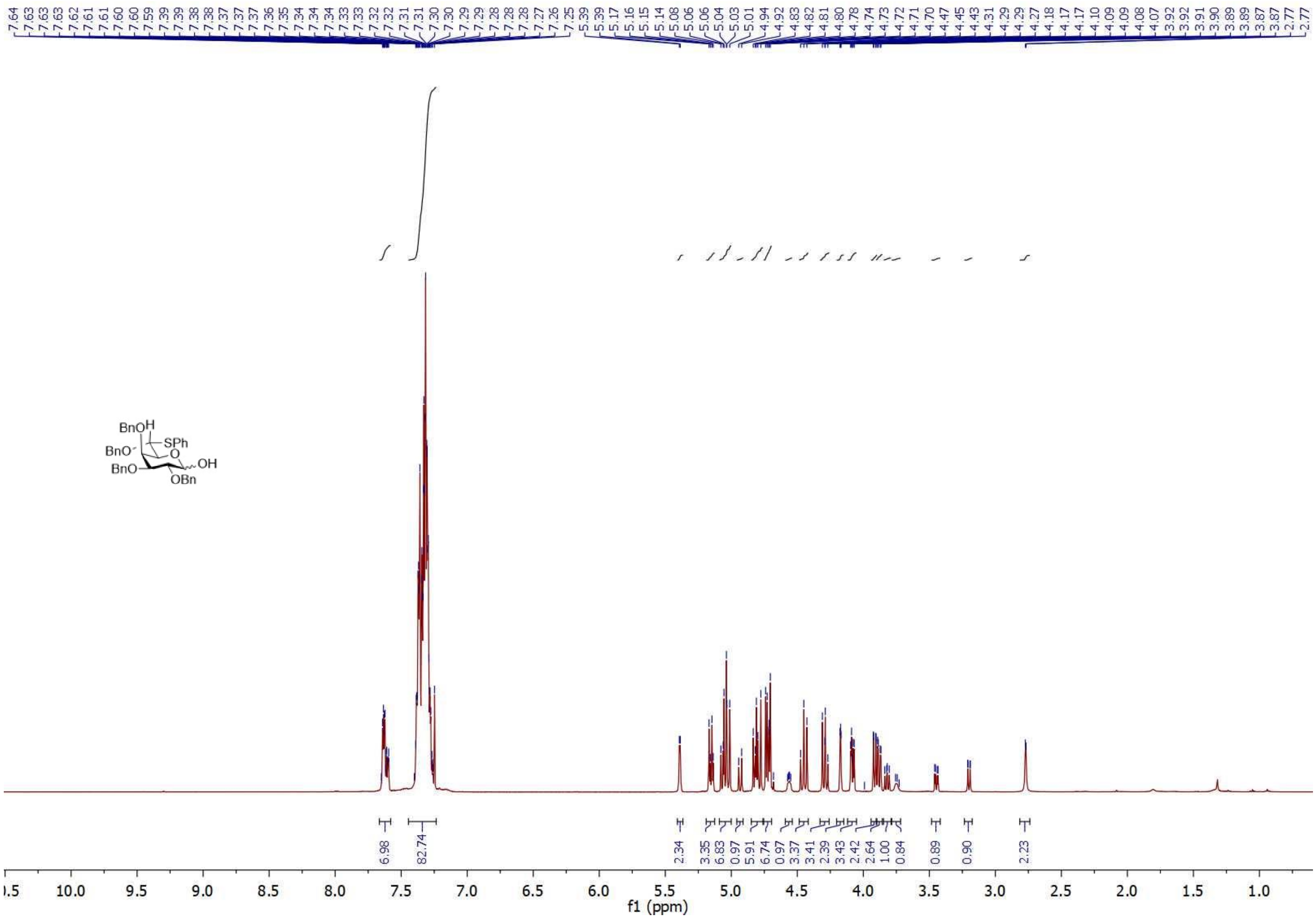

^{13}C{^{1}H} NMR (126 MHz, $CDCl_3$) Spectrum of (6*R*)-6-phenylthio-2,3,4,6-tetra-*O*-benzyl-α/*β*-D-galactopyranose **(6a)**

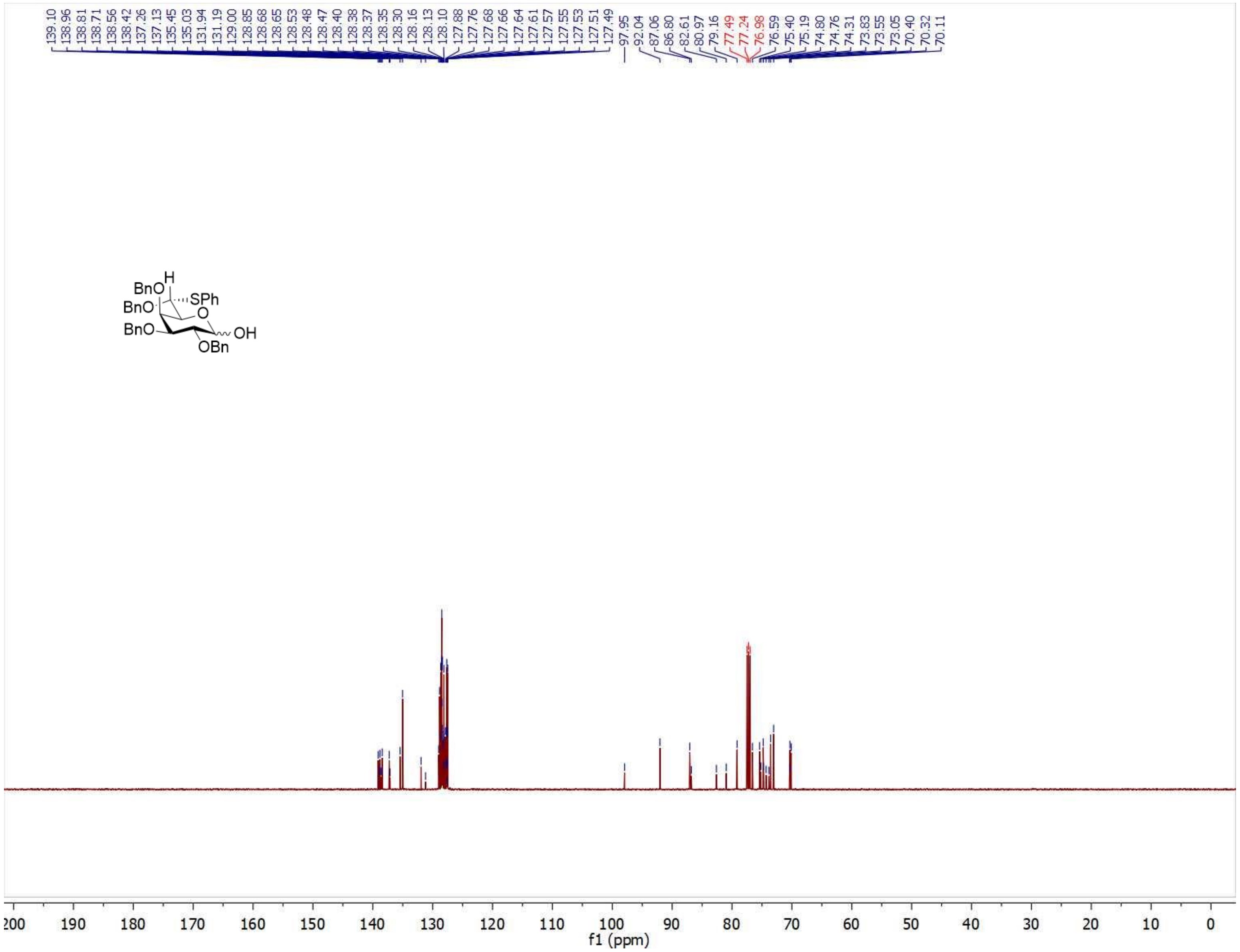

^{1}H NMR (500 MHz, $CDCl_3$) Spectrum of (6*S*)-6-phenylthio-2,3,4,6-tetra-*O*-benzyl-α/*β*-D-galactopyranose **(6b)**

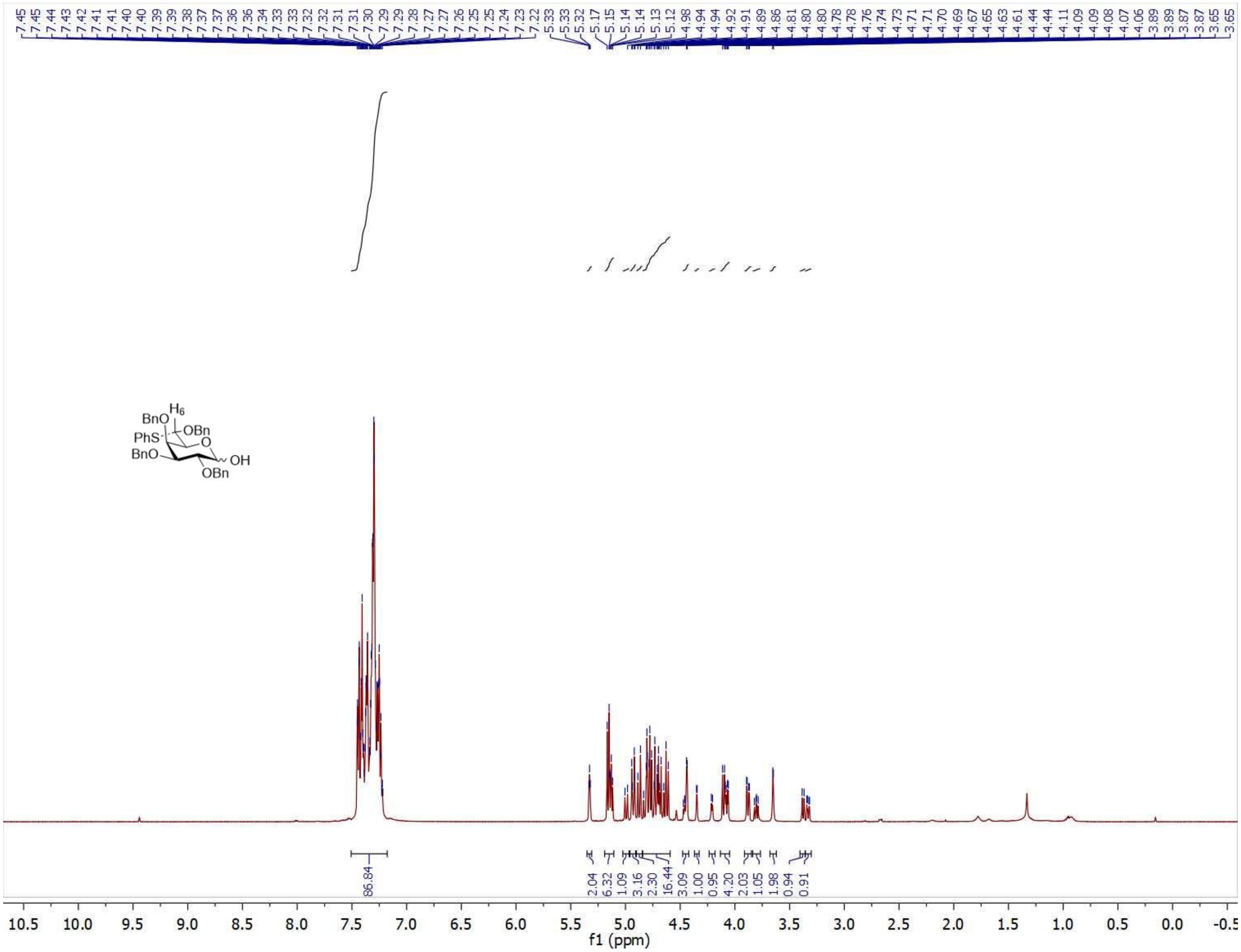

^{13}C NMR (126 MHz, $CDCl_3$) Spectrum of (6*S*)-6-phenylthio-2,3,4,6-tetra-*O*-benzyl-α/*β*-D-galactopyranose **(6b)**

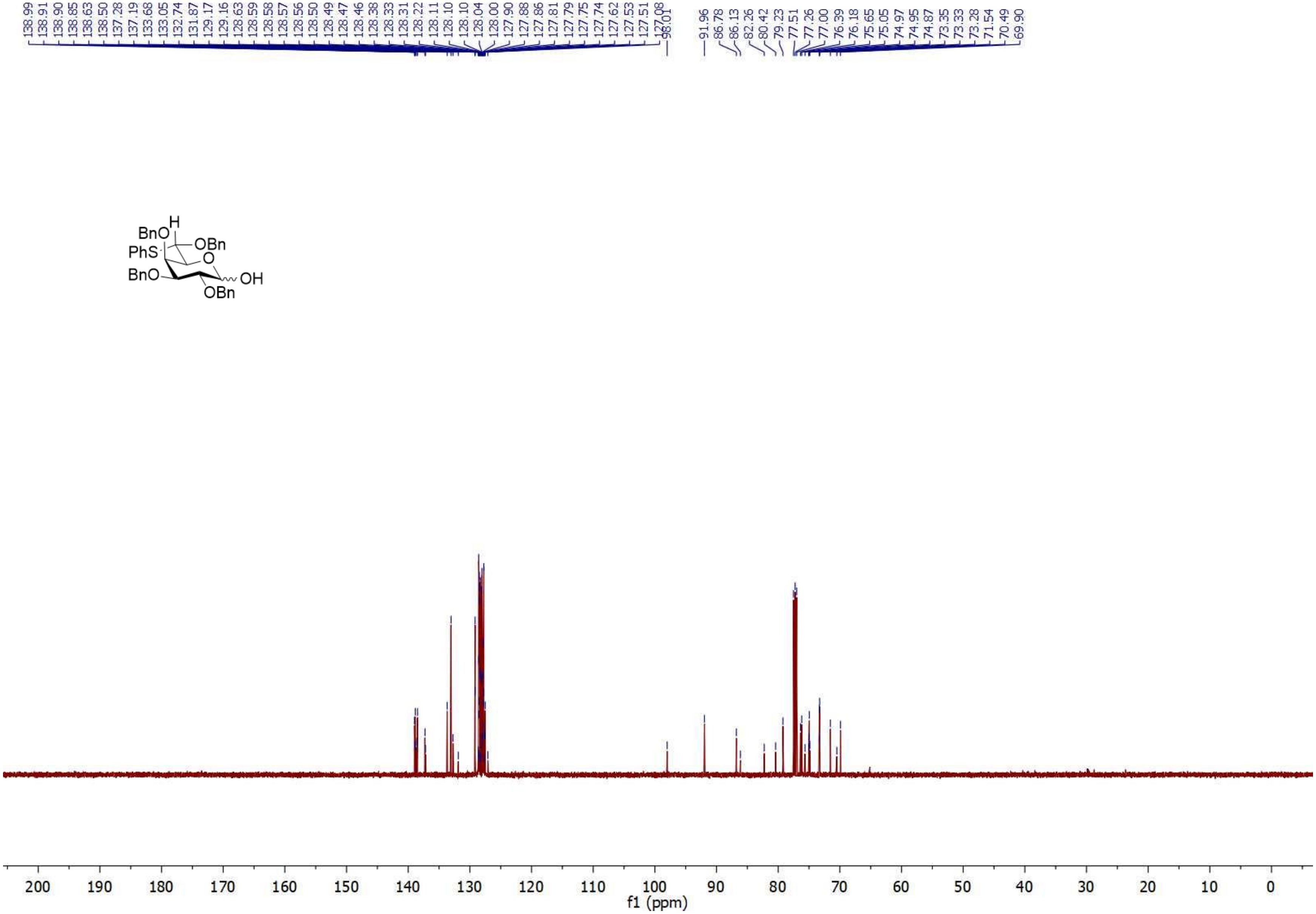

^{1}H NMR (500 MHz, CDCl$_3$) Spectrum of (6*S*)-6-phenylthio-2,3,4,6-tetra-*O*-benzyl-*α*,*β*-D-glucopyranose **(6c)**

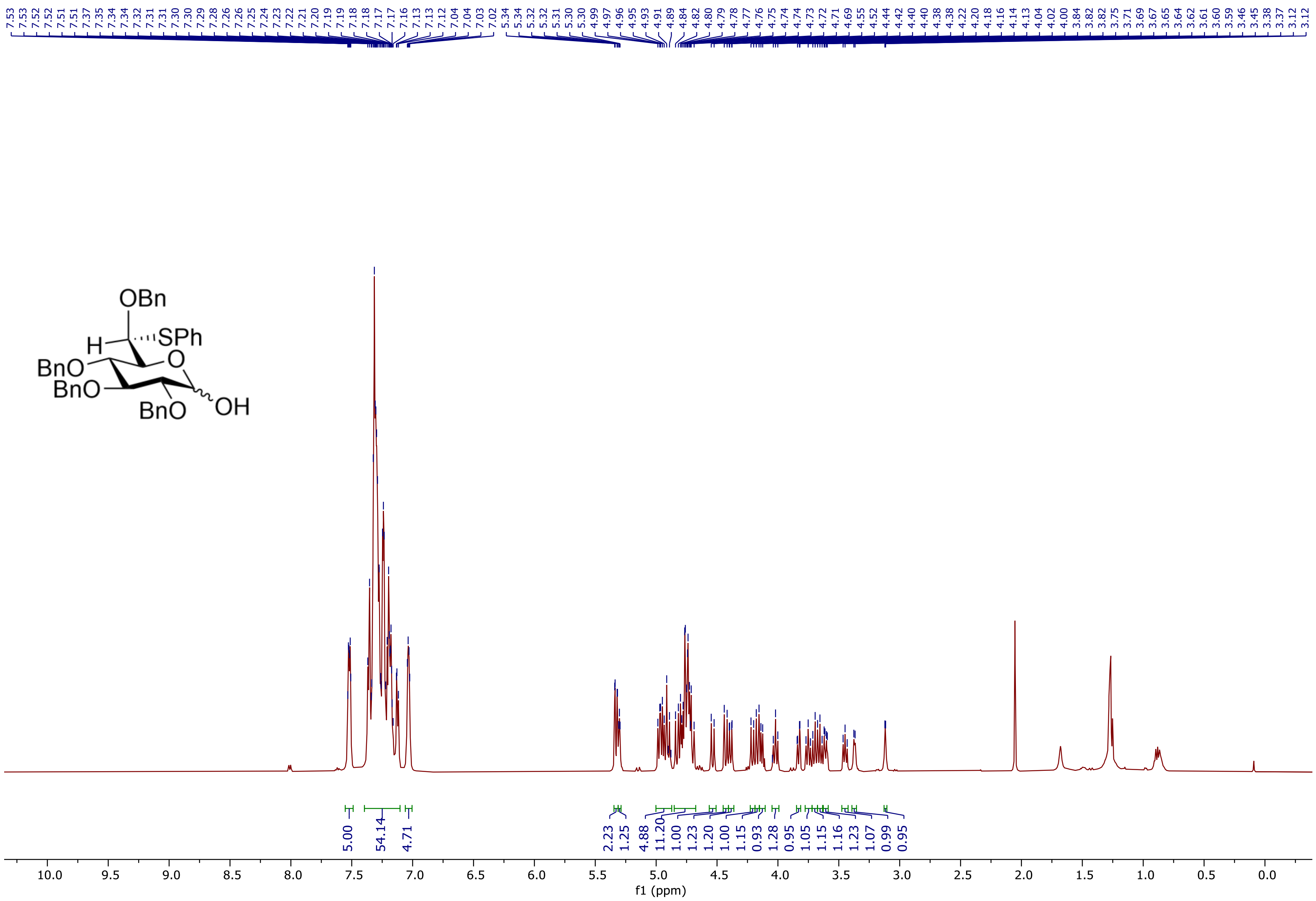

^{13}C{^{1}H} NMR (126 MHz, $CDCl_3$) Spectrum of (6*S*)-6-phenylthio-2,3,4,6-tetra-*O*-benzyl-*α*,*β*-D-glucopyranose **(6c)**

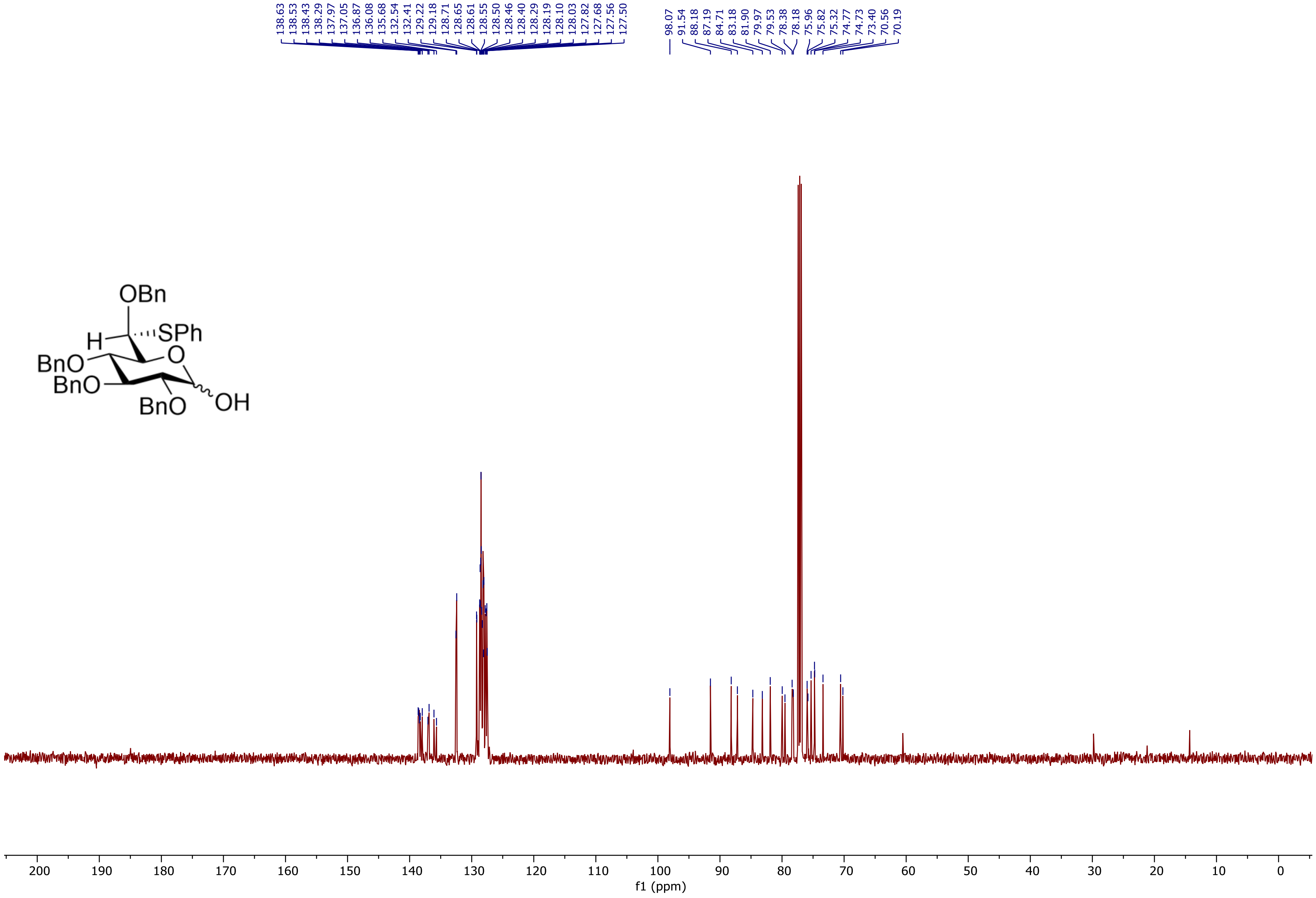

^{1}H NMR (500 MHz, $CDCl_3$) Spectrum of (6*R*)-6-phenylthio-2,3,4,6-tetra-*O*-benzyl-*α*,*β*-D-glucopyranose **(6d)**

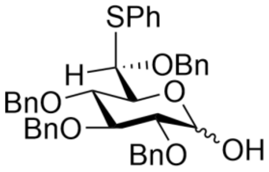

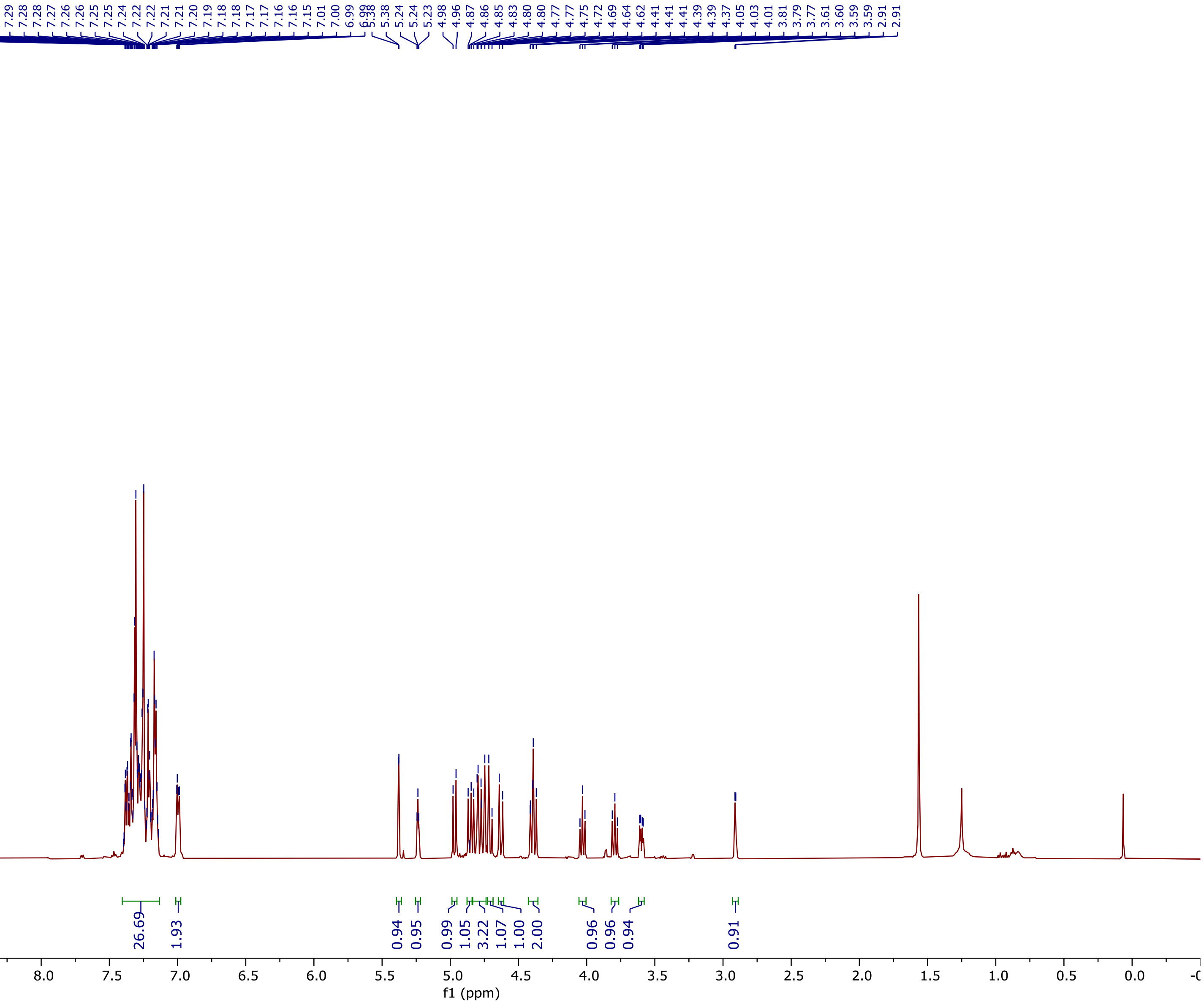

^{13}C{^{1}H} NMR (126 MHz, $CDCl_3$) Spectrum of (6*R*)-6-phenylthio-2,3,4,6-tetra-*O*-benzyl-*α*,*β*-D-glucopyranose **(6d)**

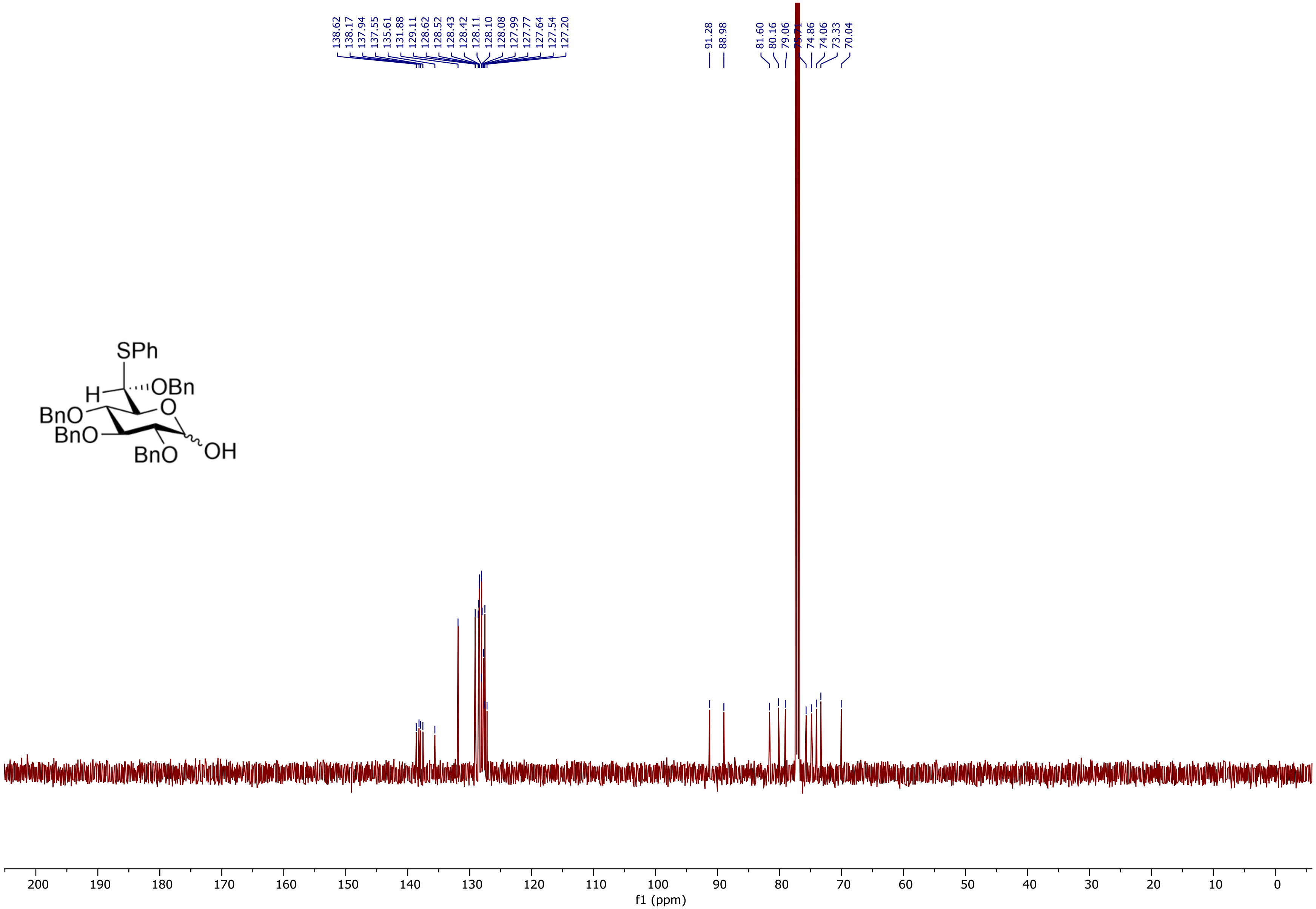

^{1}H NMR (500 MHz, $CDCl_3$) Spectrum of Methyl (6*R*)-6-phenylthio-2,3,4,6-tetra-*O*-benzyl-*β*-D-galactopyranosyl-(1→6)-2,3,4-tri-*O*-benzyl-*α*-D-glucopyranoside **(8*β*)**

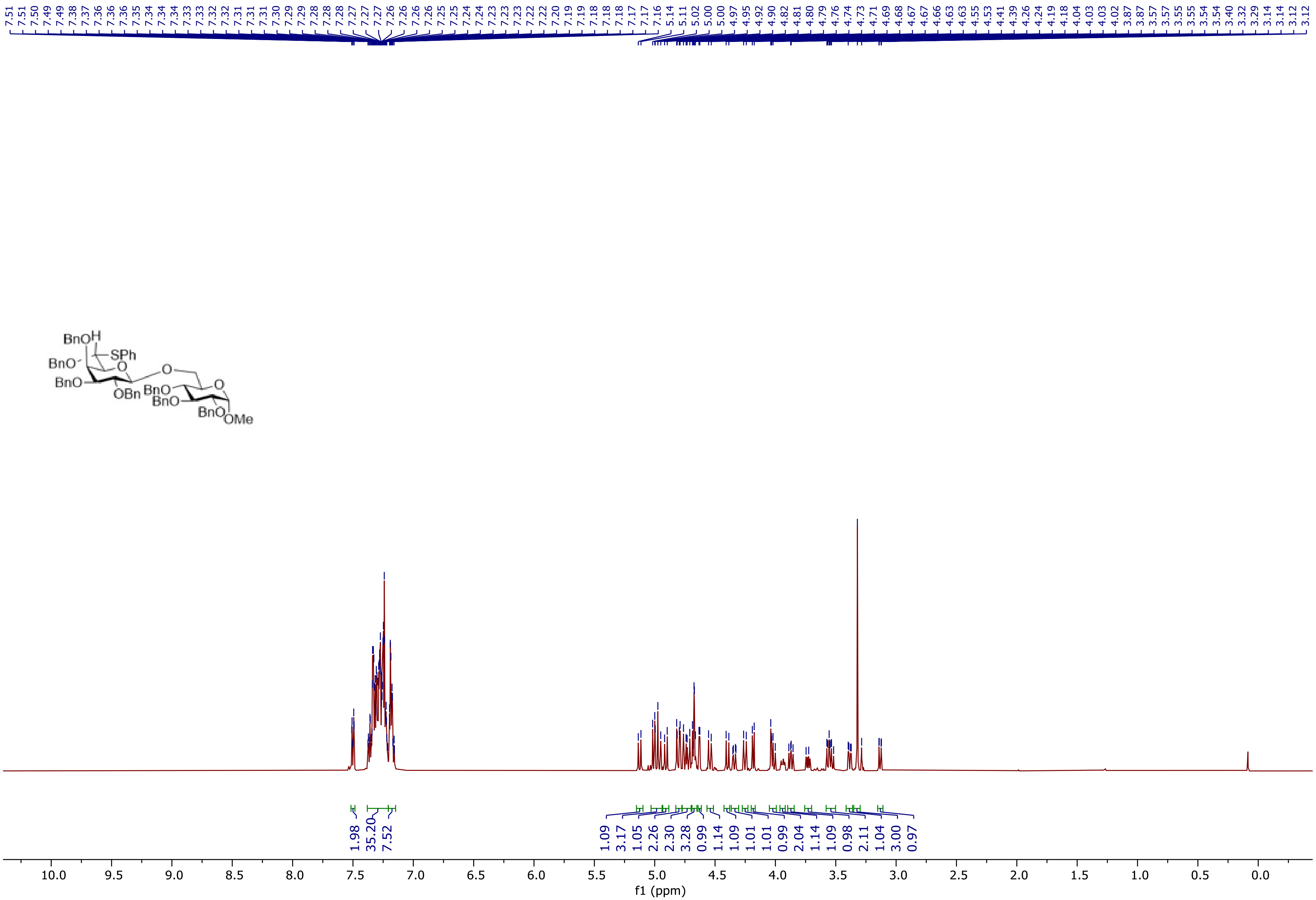

^{13}C {^{1}H} NMR (126 MHz, $CDCl_3$) Spectrum of methyl (6*R*)-6-phenylthio-2,3,4,6-tetra-*O*-benzyl-*β*-D-galactopyranosyl-(1→6)-2,3,4-tri-*O*-benzyl-*α*-D-glucopyranoside **(8*β*)**

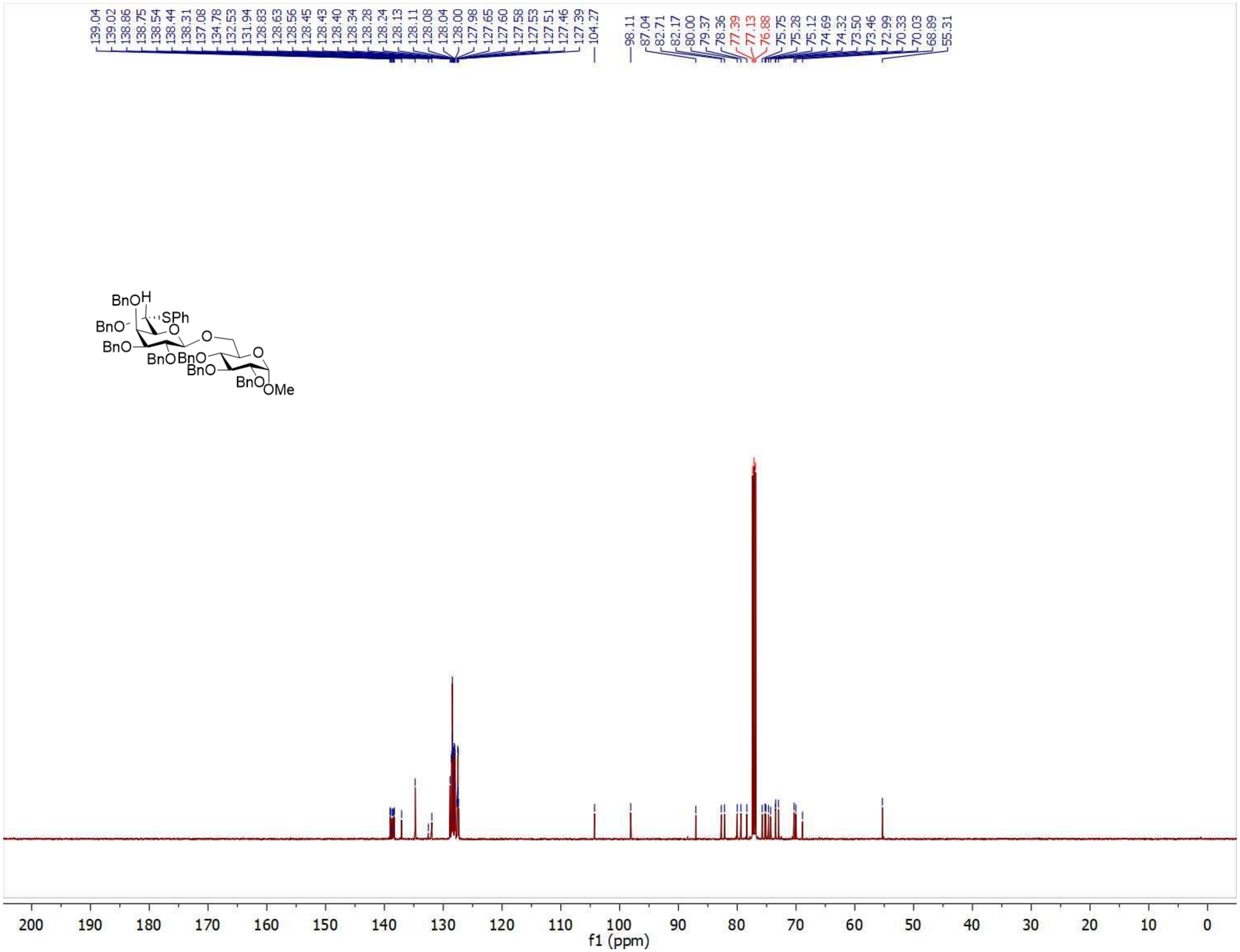

^{1}H NMR (500 MHz, $CDCl_3$) Spectrum of (6*R*)-6-phenylthio-2,3,4,6-tetra-*O*-benzyl-*β*-D-galactopyranosyl-(1→6)-1,2:3,4-*O*-diisopropylidene-*α*-D-galactopyranose **(9*β*)**

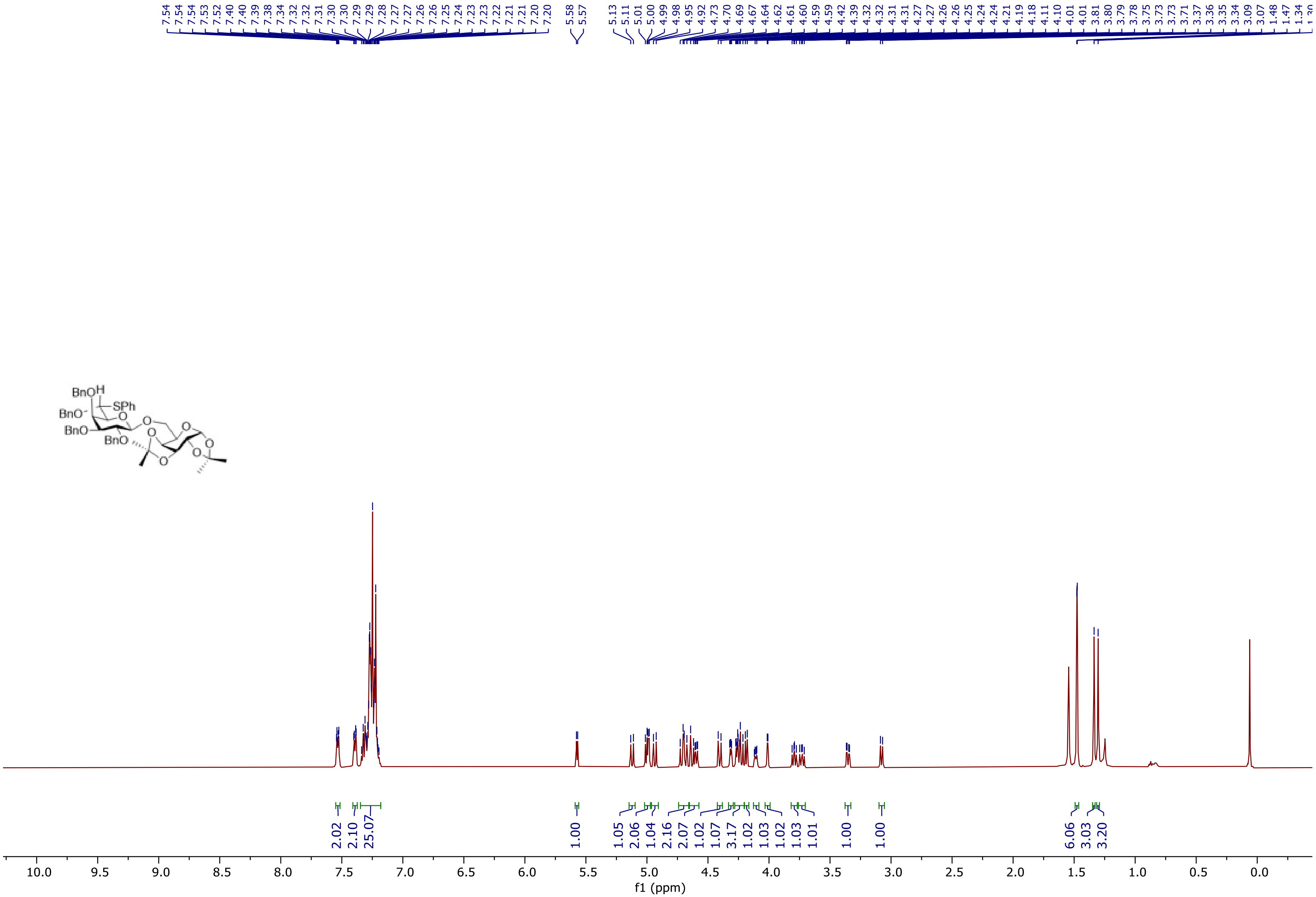

^{13}C {^{1}H} NMR (126 MHz, $CDCl_3$) Spectrum of (6*R*)-6-phenylthio-2,3,4,6-tetra-*O*-benzyl-*β*-D-galactopyranosyl-(1→6)-1,2:3,4-*O*-diisopropylidene-*α*-D-galactopyranose **(9*β*)**

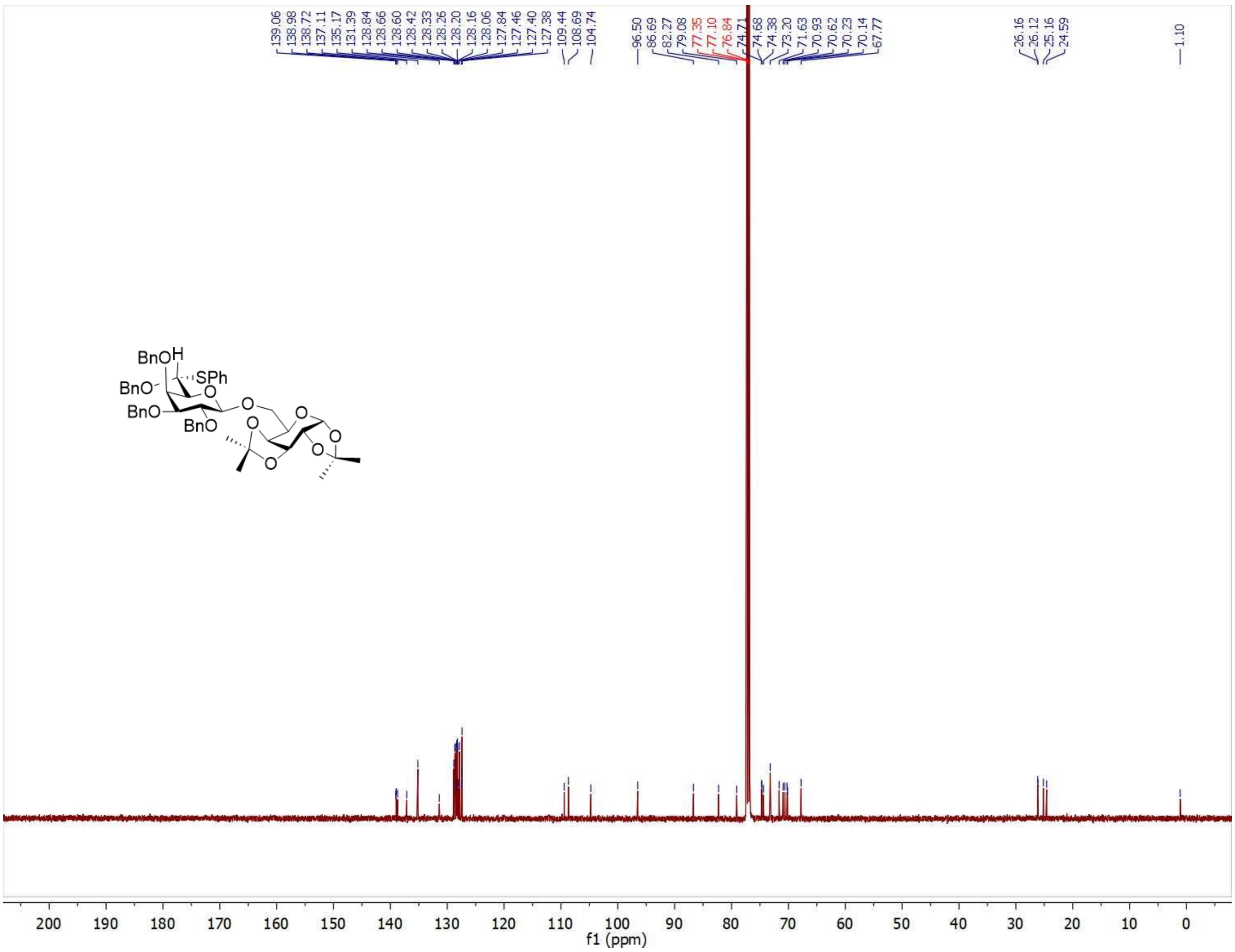

^{1}H NMR (500 MHz, $CDCl_3$) Spectrum of Adamantyl (6*R*)-6-phenylthio-2,3,4,6-tetra-*O*-benzyl-*β*-D-galactopyranoside **(10*β*)**

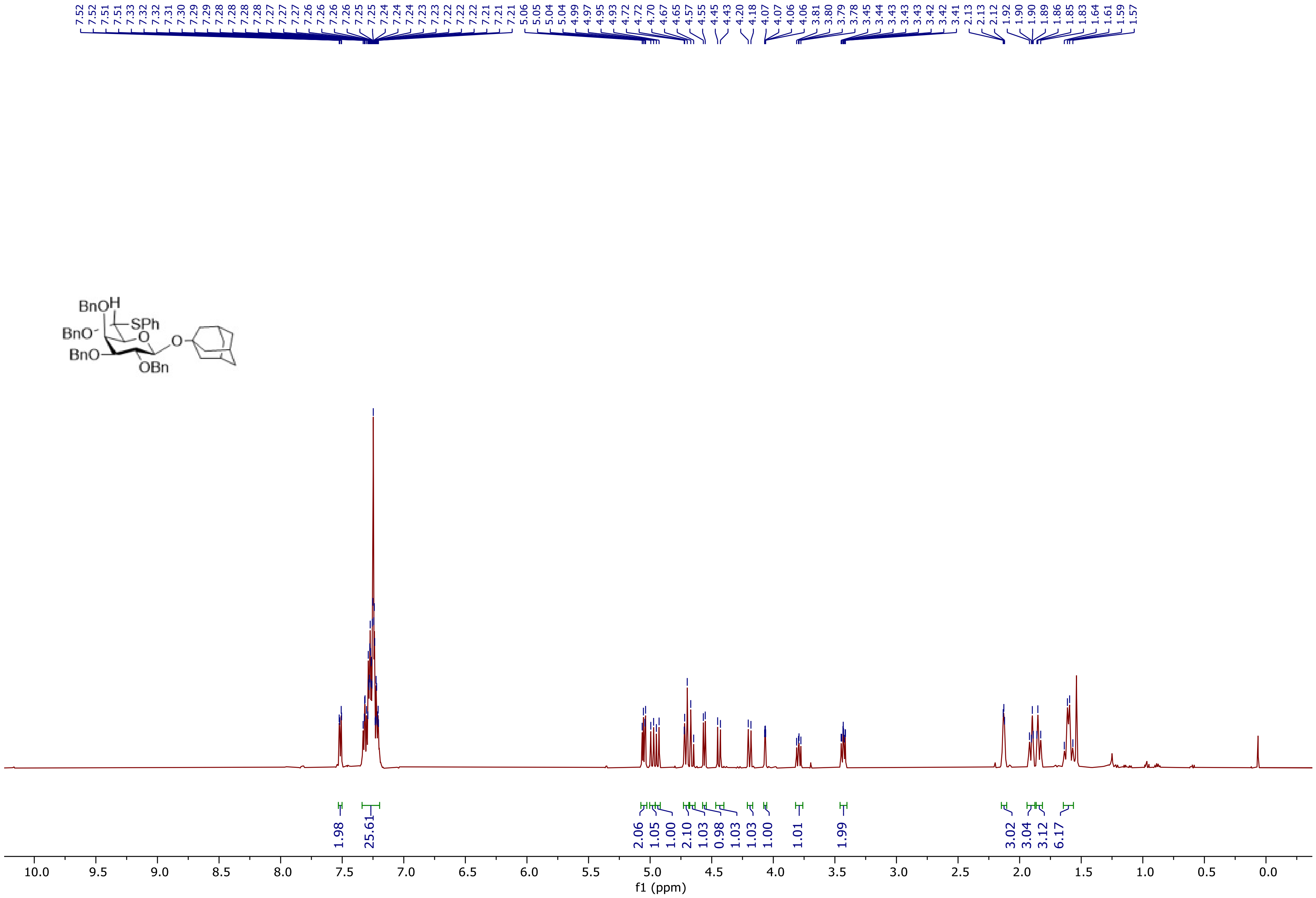

^{13}C {^{1}H} NMR (126 MHz, $CDCl_3$) Spectrum of Adamantyl (6*R*)-6-phenylthio-2,3,4,6-tetra-*O*-benzyl-*β*-D-galactopyranoside **(10*β*)**

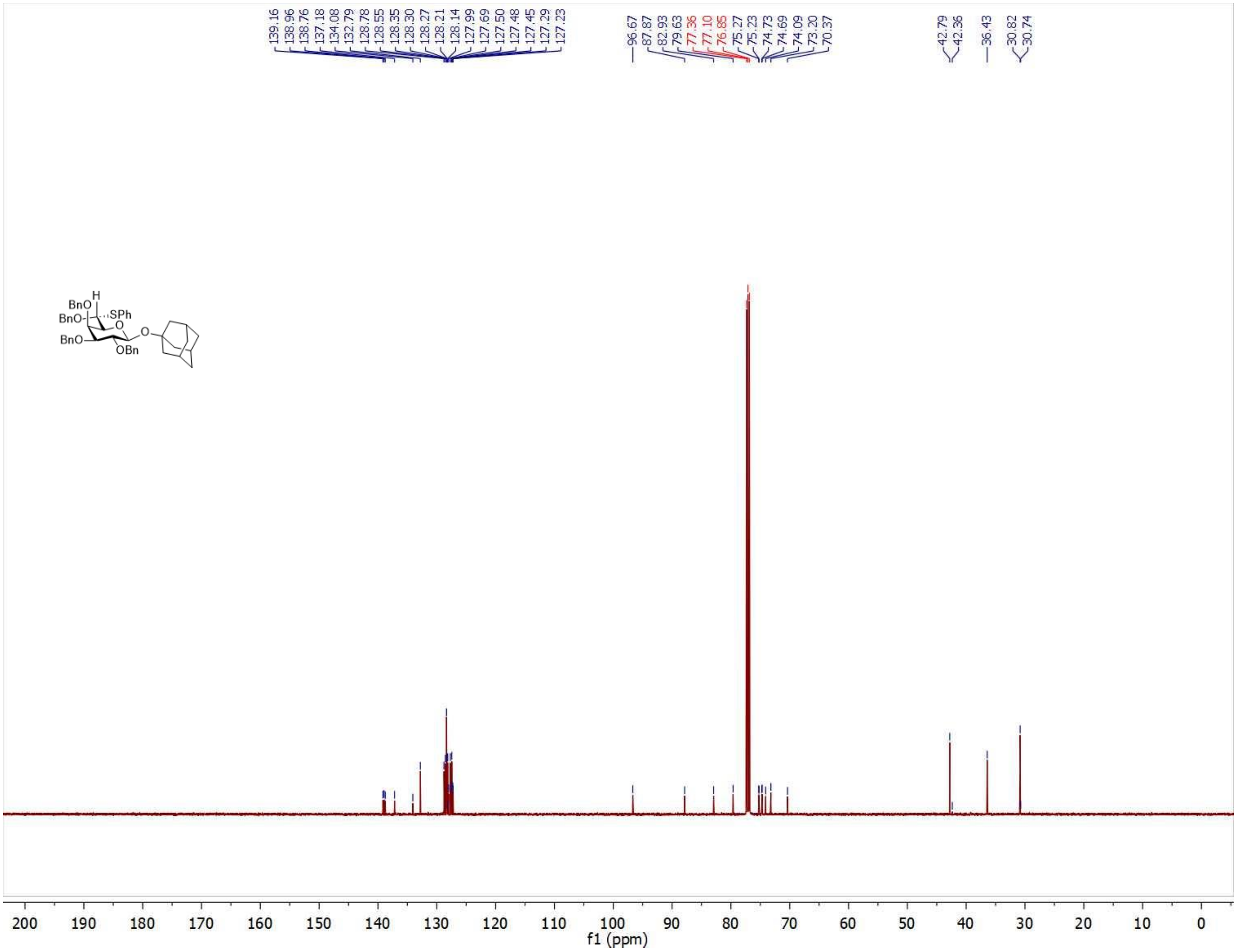

^{1}H NMR (500 MHz, $CDCl_3$) Spectrum of (6*R*)-6-phenylthio-2,3,4,6-tetra-*O*-benzyl-*α*-D-galactopyranosyl-(1→3)-1,2:5,6-di-*O*-isopropylidene-*α*-D-glucofuranose **(11*α*)**

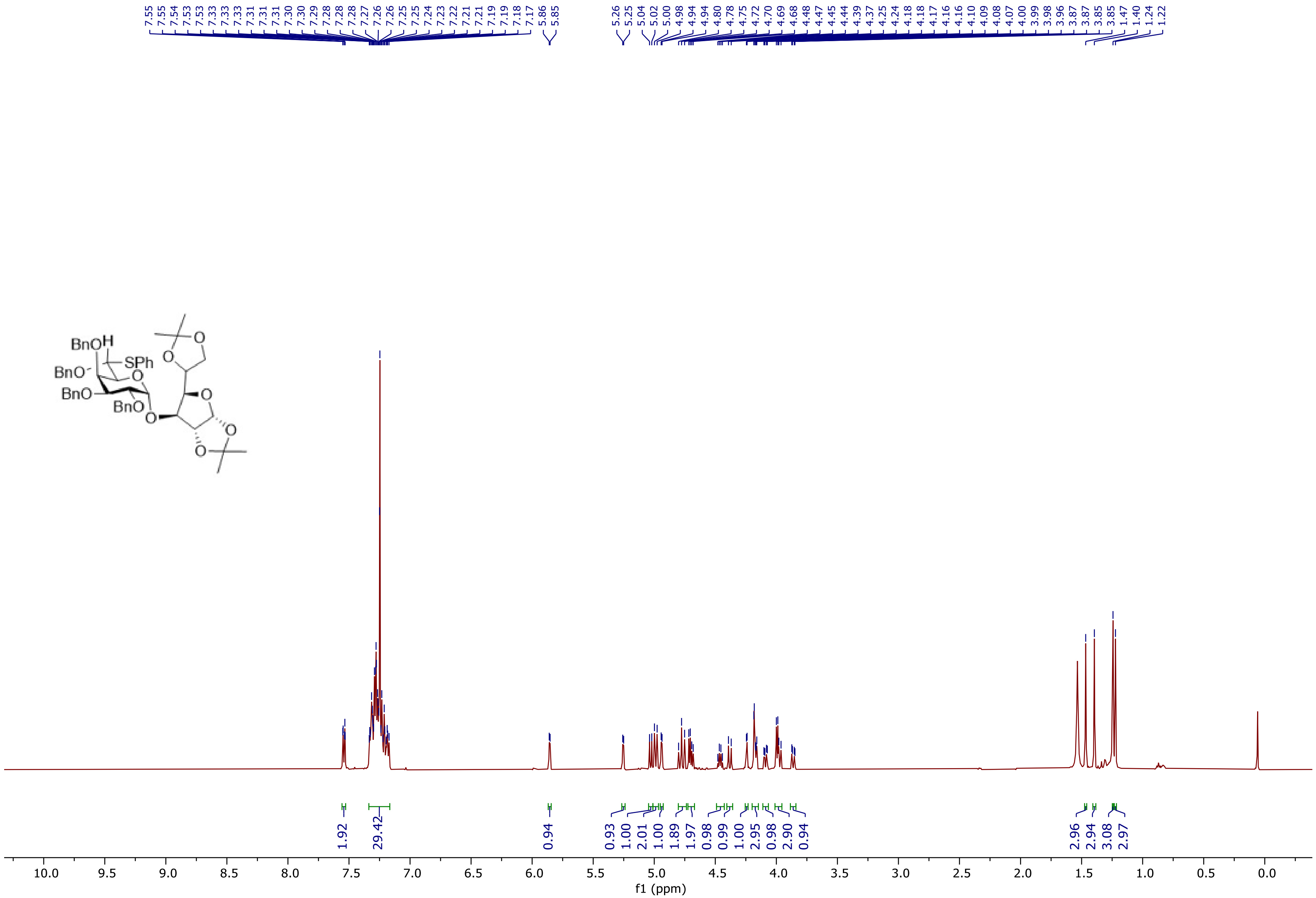

^{13}C {^{1}H} NMR (126 MHz, $CDCl_3$) Spectrum of (6*R*)-6-phenylthio-2,3,4,6-tetra-*O*-benzyl-*α*-D-galactopyranosyl-(1→3)-1,2:5,6-di-*O*-isopropylidene-*α*-D-glucofuranose **(11*α*)**

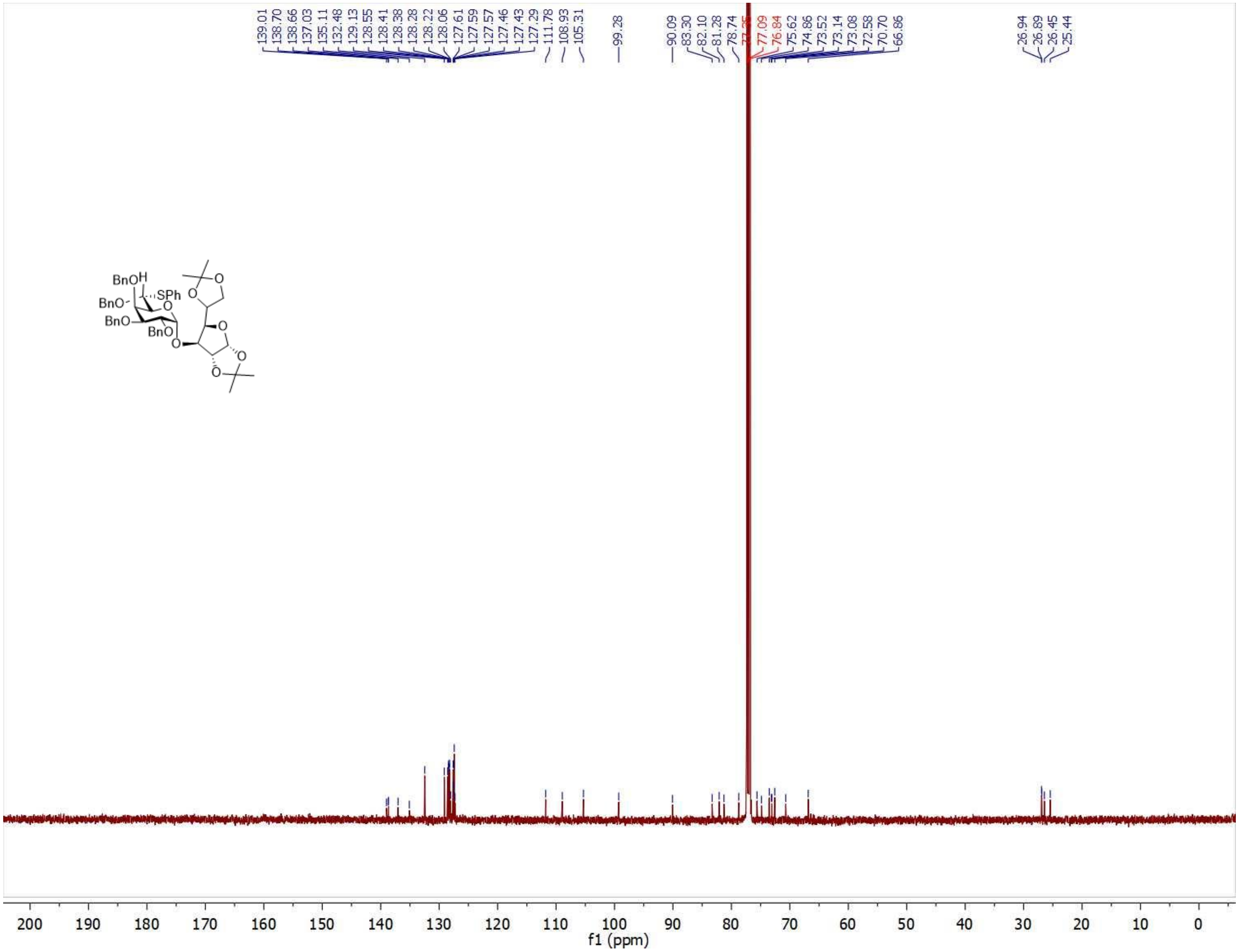

^{1}H NMR (500 MHz, $CDCl_3$) Spectrum of (6*R*)-6-phenylthio-2,3,4,6-tetra-*O*-benzyl-*β*-D-galactopyranosyl-(1→3)-1,2:5,6-di-*O*-isopropylidene-*α*-D-glucofuranose **(11*β*)**

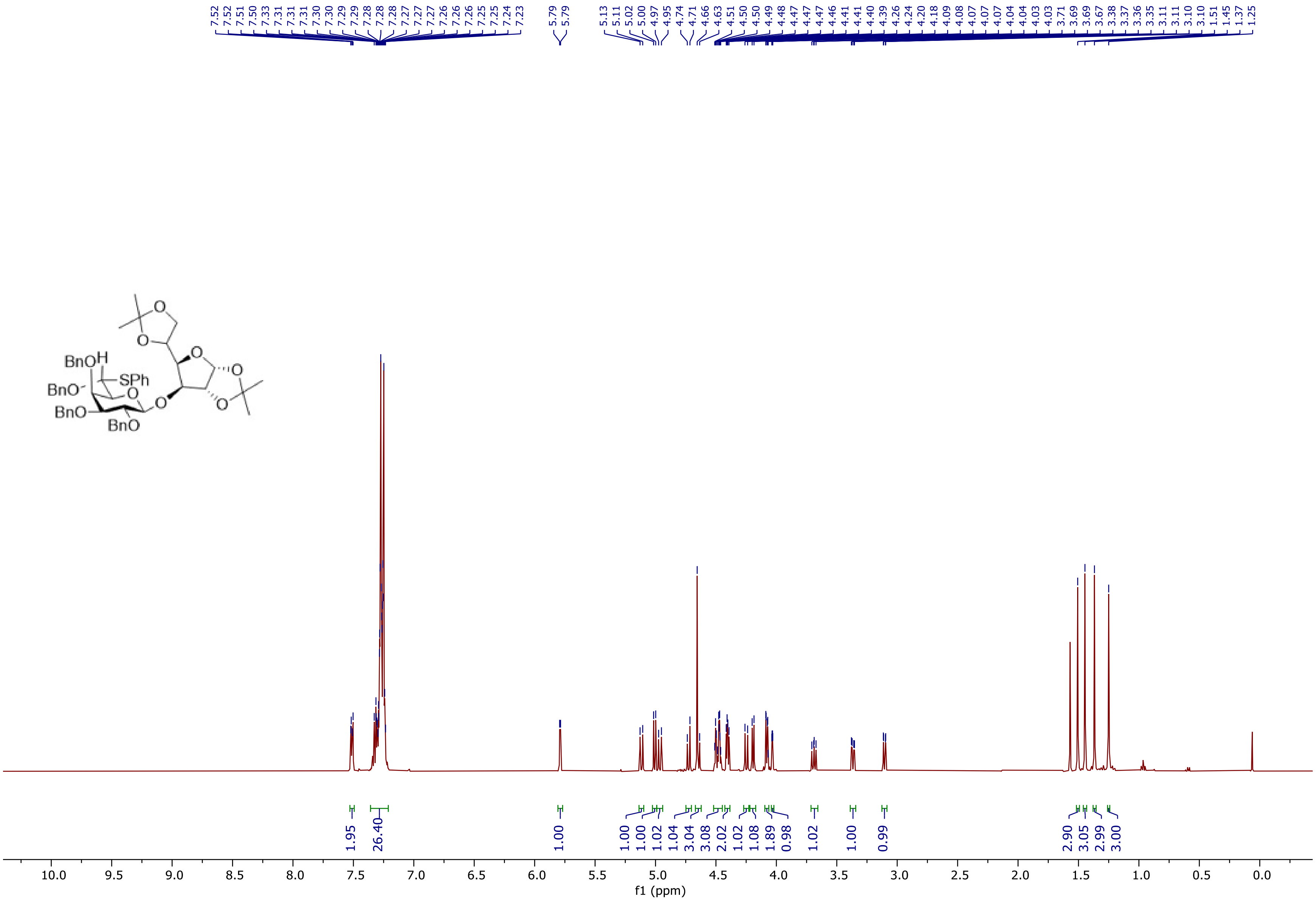

^{13}C {^{1}H} NMR (126 MHz, $CDCl_3$) Spectrum of (6*R*)-6-phenylthio-2,3,4,6-tetra-*O*-benzyl-*β*-D-galactopyranosyl-(1→3)-1,2:5,6-di-*O*-isopropylidene-*α*-D-glucofuranose **(11*β*)**

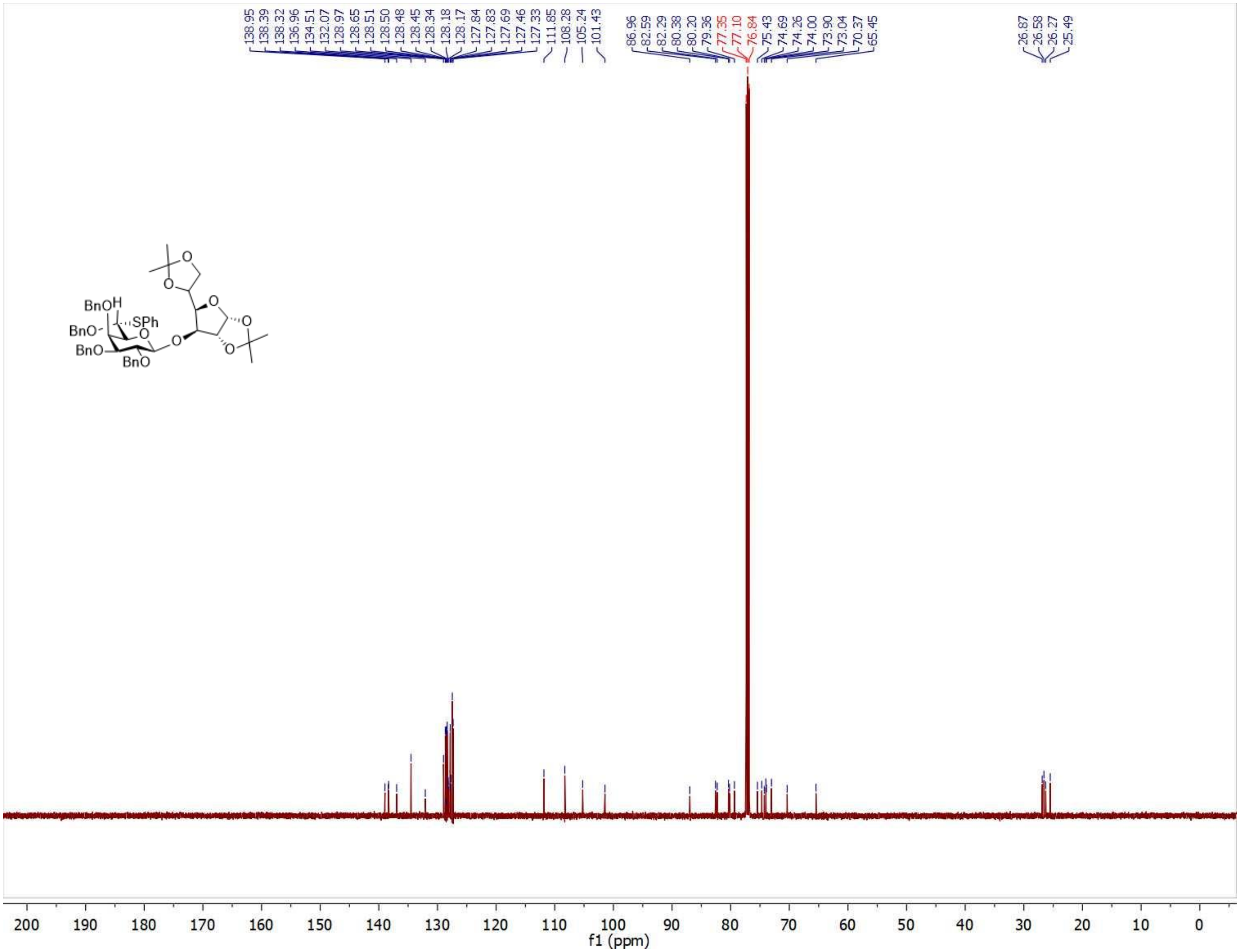

^{1}H NMR (500 MHz, $CDCl_3$) Spectrum of Methyl (6*R*)-6-phenylthio-2,3,4,6-tetra-*O*-benzyl-*β*-D-galactopyranosyl-(1→4)-2,3-*O*-isopropylidene-*α*-L-rhamnopyranoside **(12*β*)**

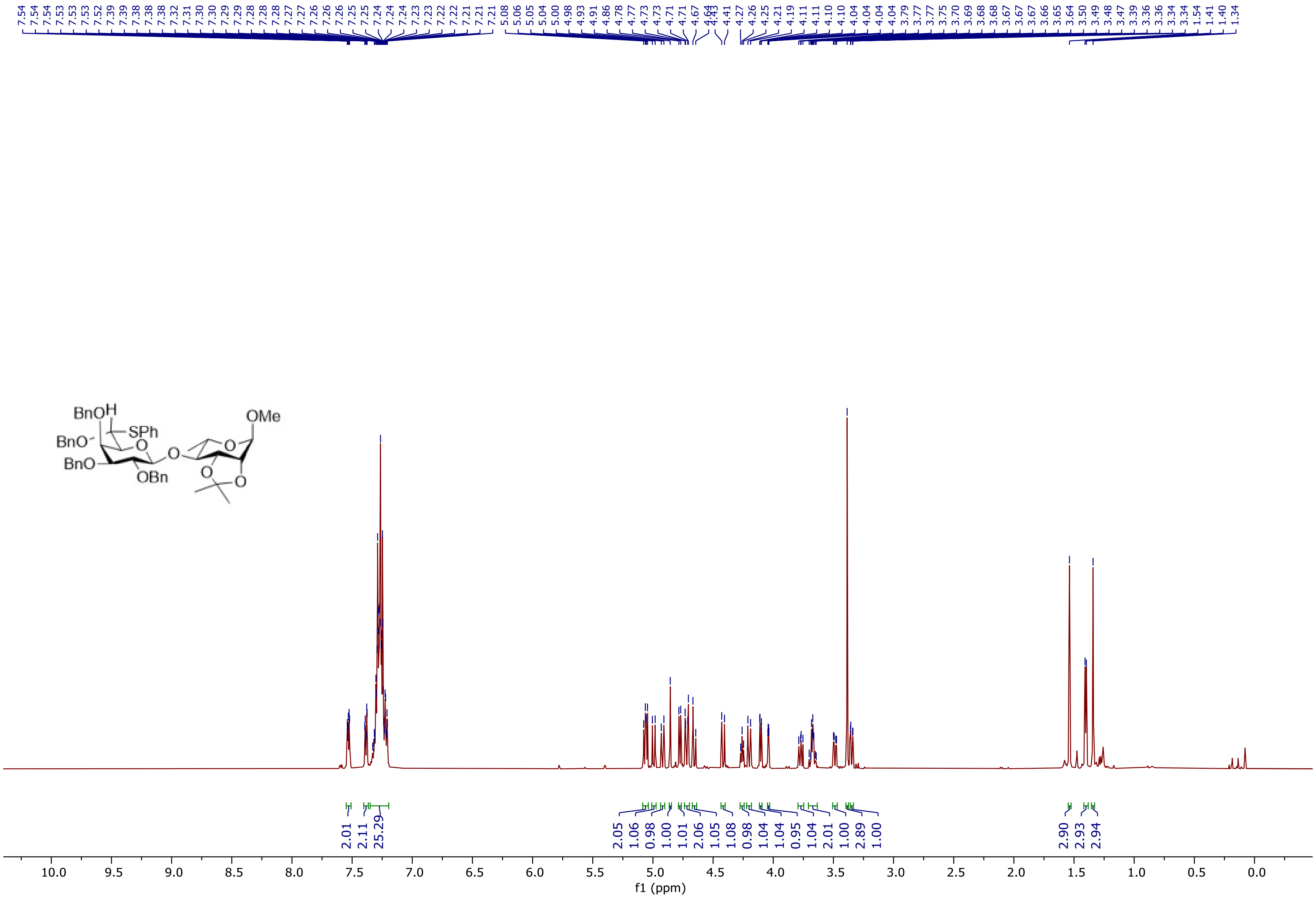

^{13}C {^{1}H} NMR (126 MHz, $CDCl_3$) Spectrum of Methyl (6*R*)-6-phenylthio-2,3,4,6-tetra-*O*-benzyl-*β*-D-galactopyranosyl-(1→4)-2,3-*O*-isopropylidene-*α*-L-rhamnopyranoside **(12*β*)**

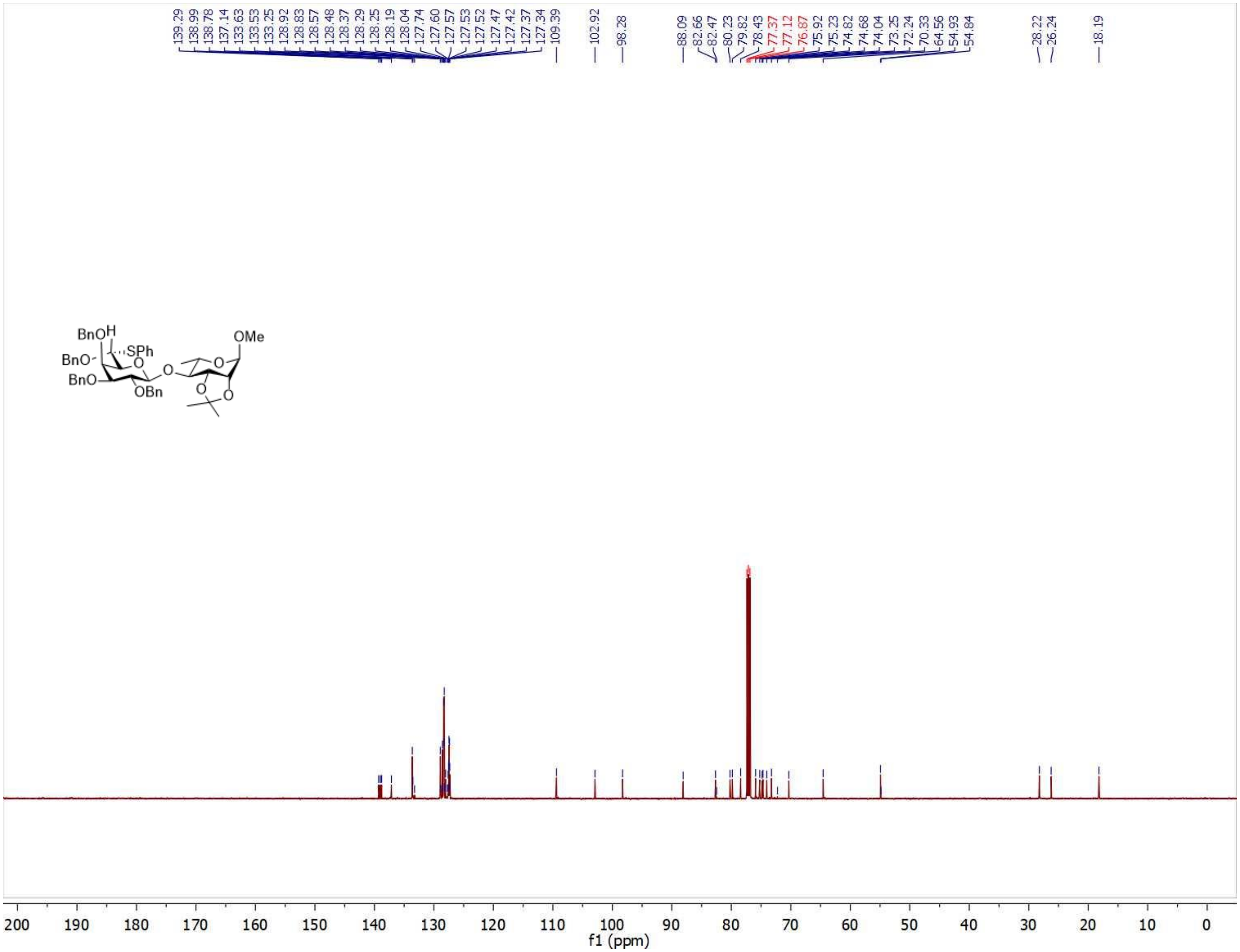

^{1}H NMR (500 MHz, $CDCl_3$) Spectrum of Methyl (6*R*)-6-phenylthio-2,3,4,6-tetra-*O*-benzyl-*α*-D-galactopyranosyl-(1→4)-2,3,4-tri-*O*-benzyl-*α*-D-glucopyranoside **(13*α*)**

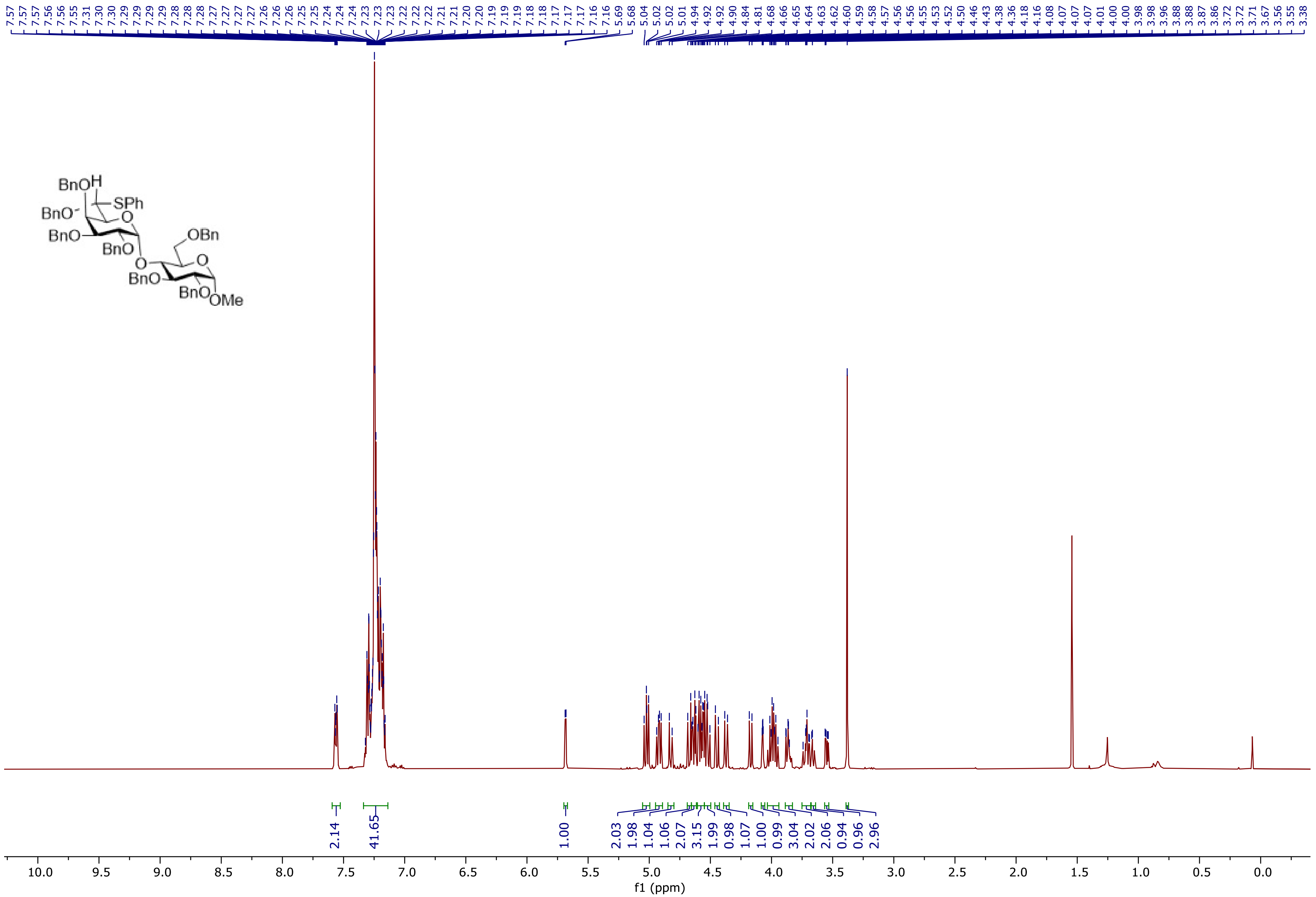

^{13}C {^{1}H} NMR (126 MHz, $CDCl_3$) Spectrum of Methyl (6*R*)-6-phenylthio-2,3,4,6-tetra-*O*-benzyl-*α*-D-galactopyranosyl-(1→4)-2,3,4-tri-*O*-benzyl-*α*-D-glucopyranoside **(13*α*)**

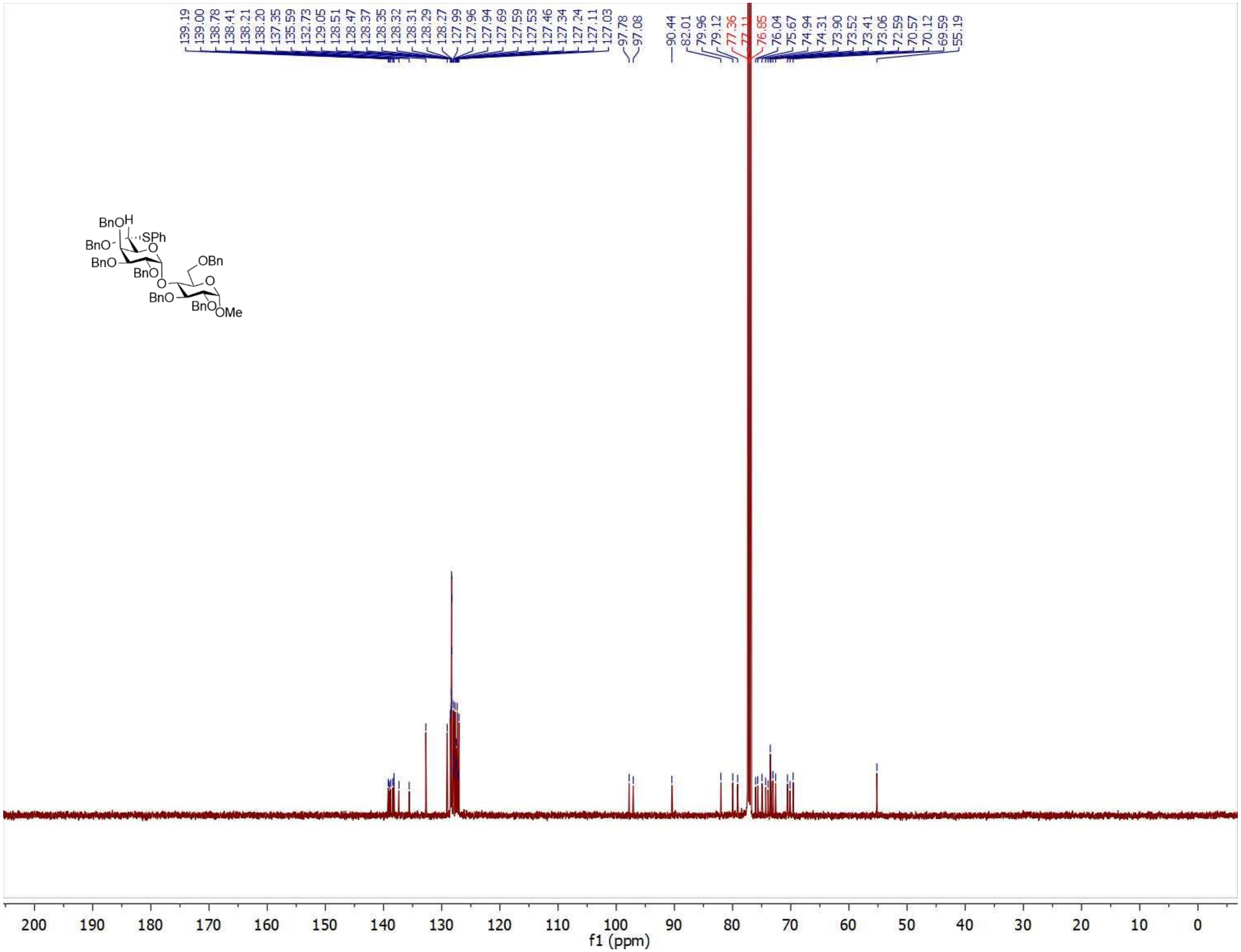

^{1}H NMR (500 MHz, $CDCl_3$) Spectrum of Methyl (6*R*)-6-phenylthio-2,3,4,6-tetra-*O*-benzyl-*β*-D-galactopyranosyl-(1→4)-2,3,4-tri-*O*-benzyl-*α*-D-glucopyranoside **(13*β*)**

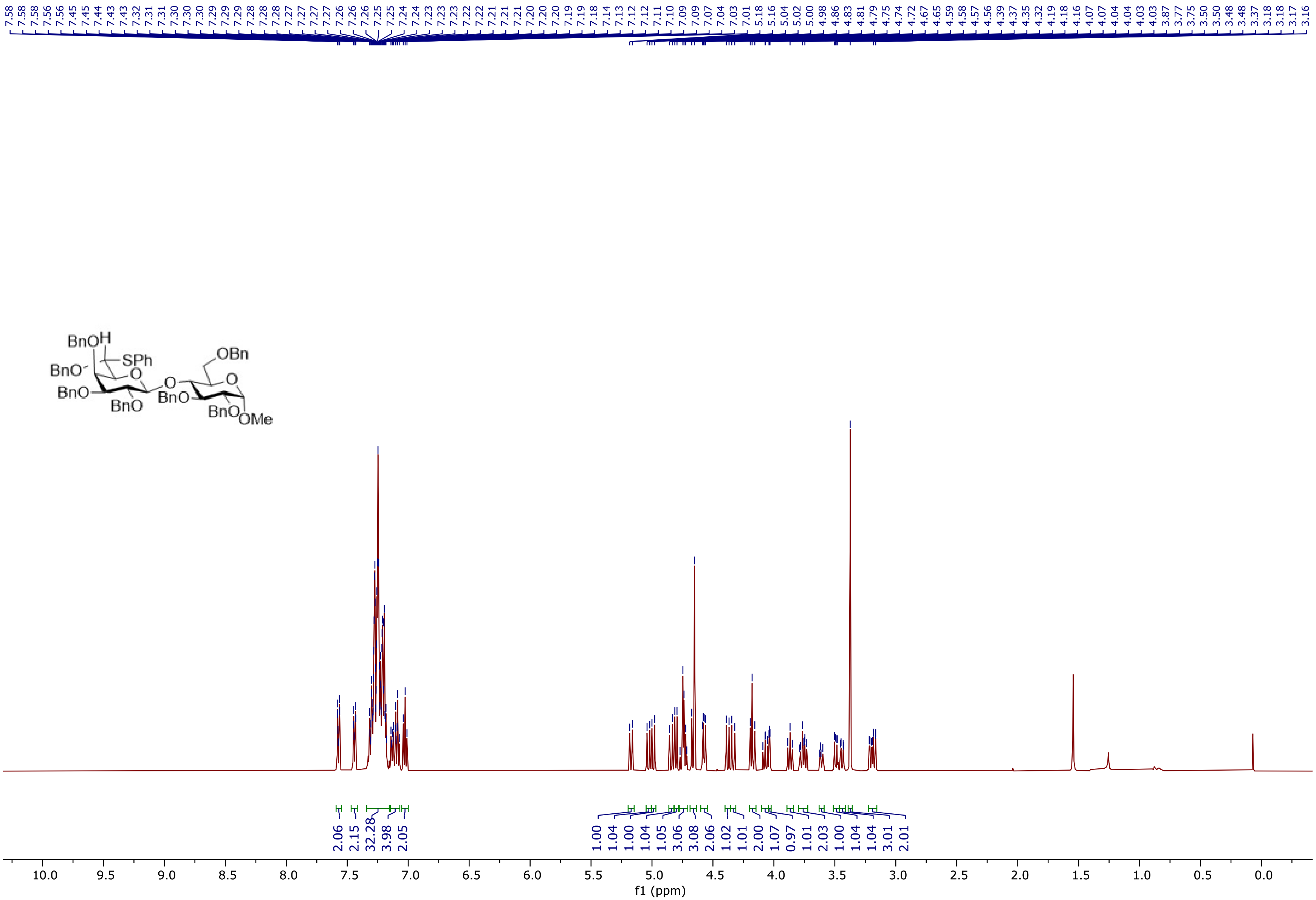

^{13}C {^{1}H} NMR (126 MHz, $CDCl_3$) Spectrum of Methyl (6*R*)-6-phenylthio-2,3,4,6-tetra-*O*-benzyl-*β*-D-galactopyranosyl-(1→4)-2,3,4-tri-*O*-benzyl-*α*-D-glucopyranoside **(13*β*)**

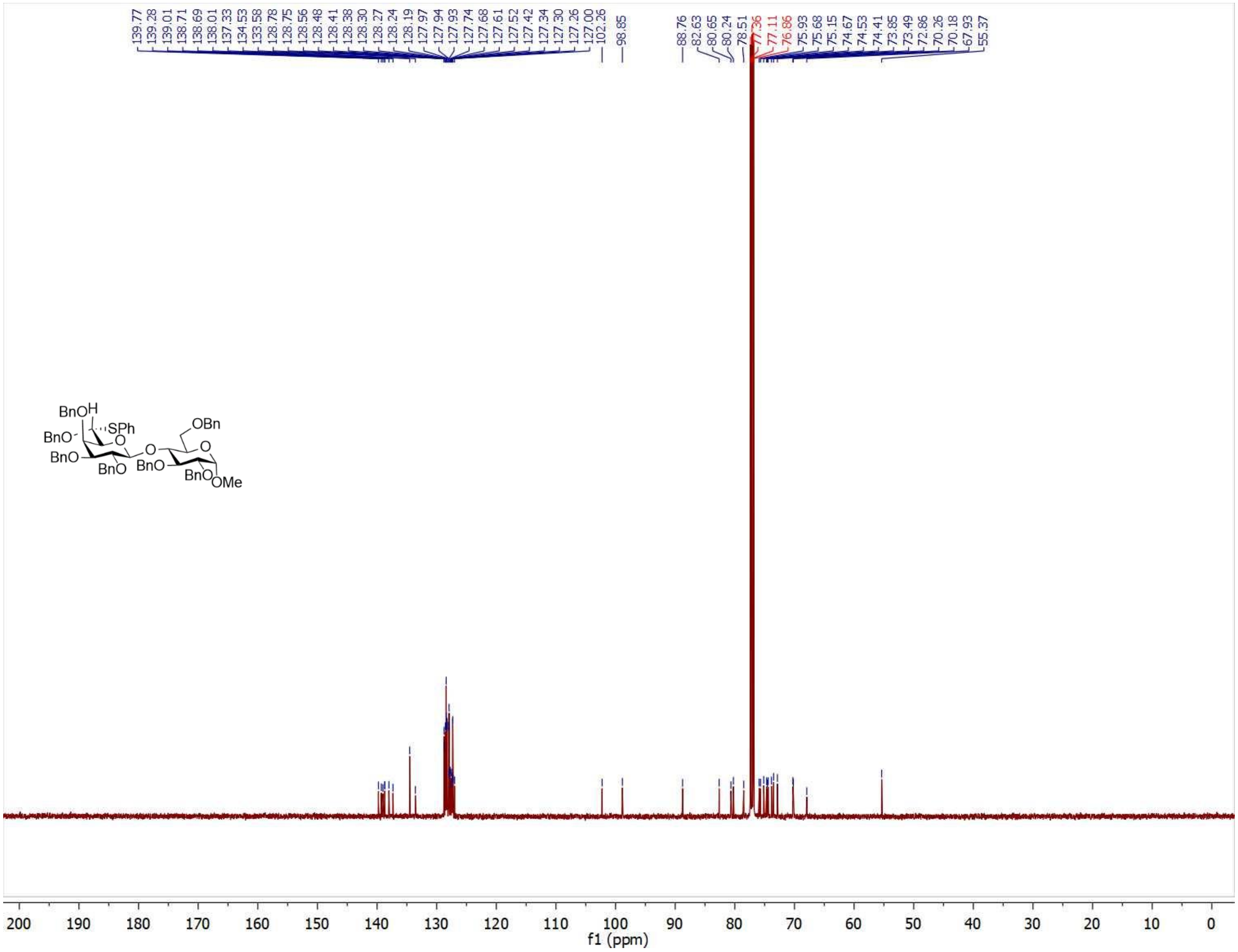

^{1}H NMR (900 MHz, $CDCl_3$) Spectrum of Methyl (6*S*)-6-phenylthio-2,3,4,6-tetra-*O*-benzyl-*α*-D-galactopyranosyl-(1→6)-2,3,4-tri-*O*-benzyl-*α*-D-glucopyranoside **(14*α*)**

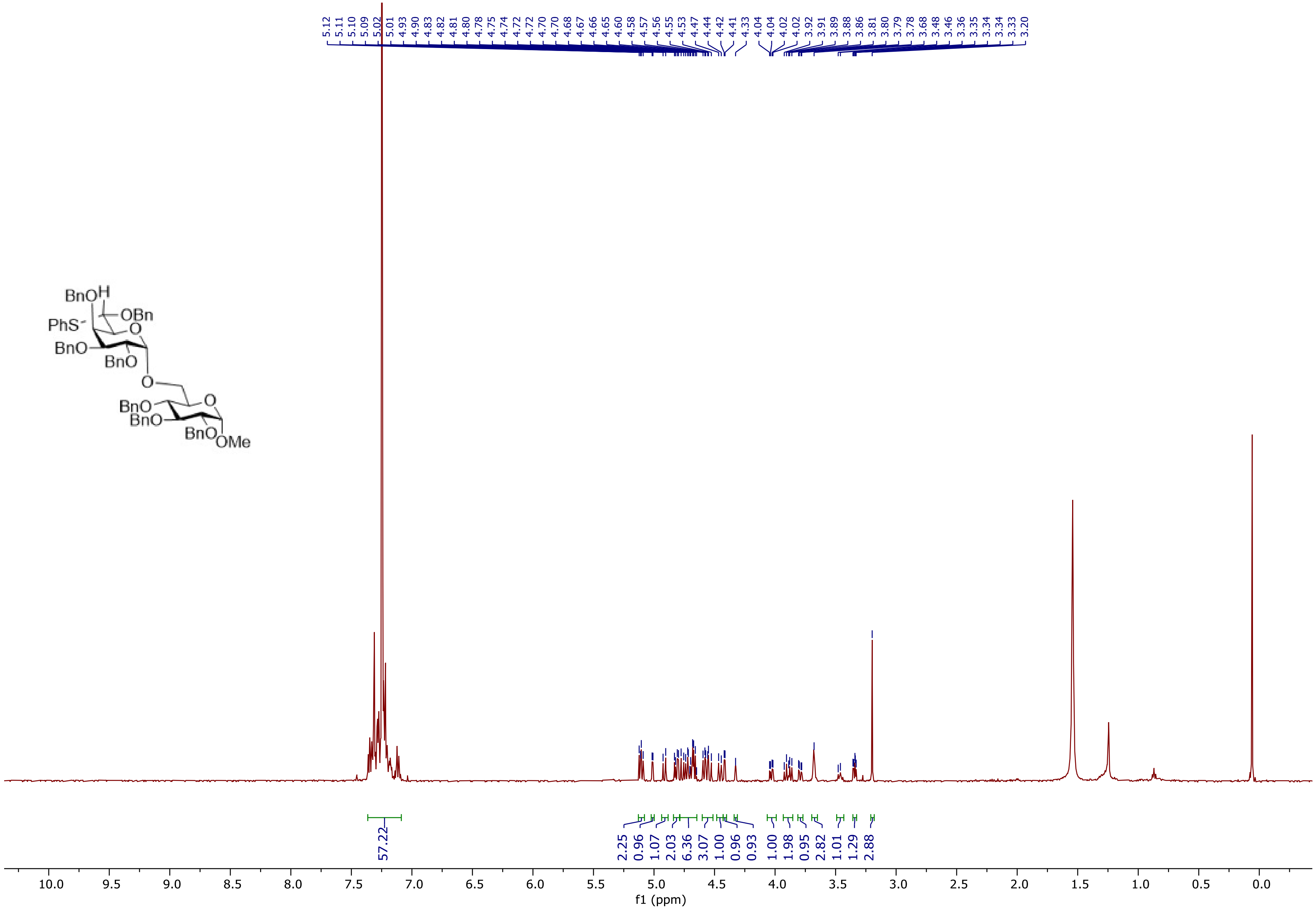

^{13}C {^{1}H} NMR (225 MHz, $CDCl_3$) Spectrum of Methyl (6*S*)-6-phenylthio-2,3,4,6-tetra-*O*-benzyl-*α*-D-galactopyranosyl-(1→6)-2,3,4-tri-*O*-benzyl-*α*-D-glucopyranoside **(14*α*)**

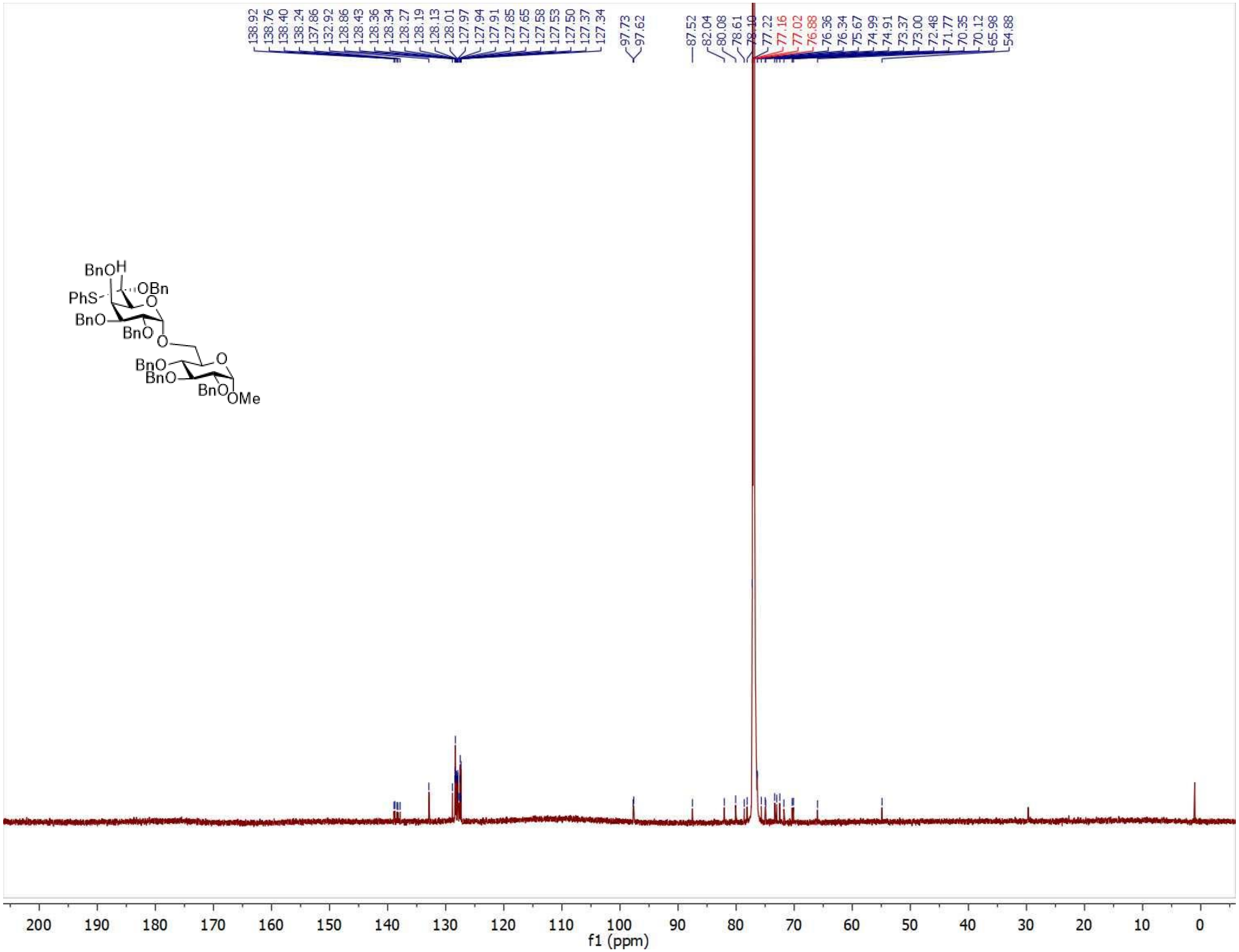

¹H NMR (500 MHz, $CDCl_3$) Spectrum of Methyl (6*S*)-6-phenylthio-2,3,4,6-tetra-*O*-benzyl-*β*-D-galactopyranosyl-(1→6)-2,3,4-tri-*O*-benzyl-*α*-D-glucopyranoside **(14*β*)**

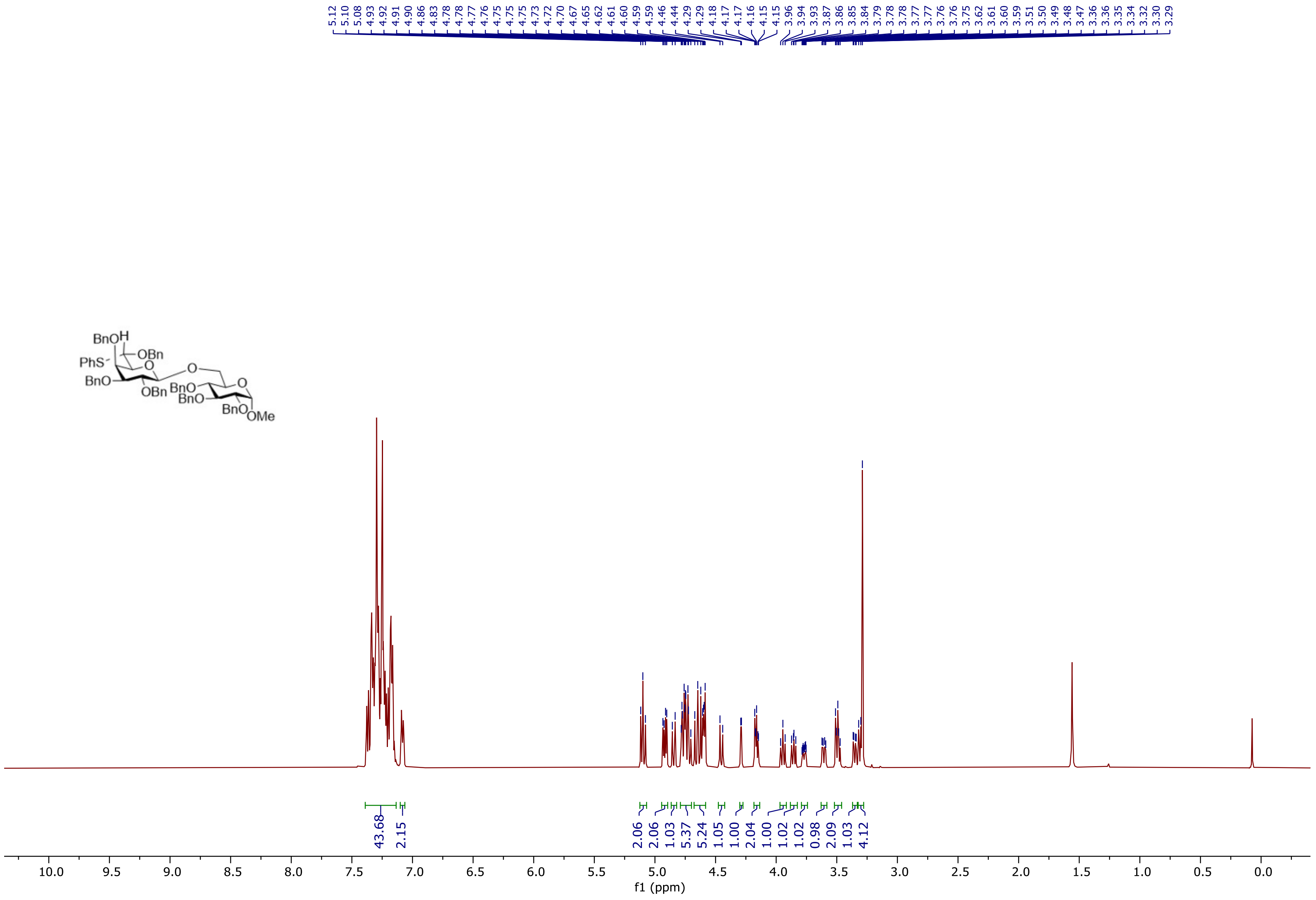

^{13}C {^{1}H} NMR (126 MHz, $CDCl_3$) Spectrum of Methyl (6*S*)-6-phenylthio-2,3,4,6-tetra-*O*-benzyl-*β*-D-galactopyranosyl-(1→6)-2,3,4-tri-*O*-benzyl-*α*-D-glucopyranoside **(14*β*)**

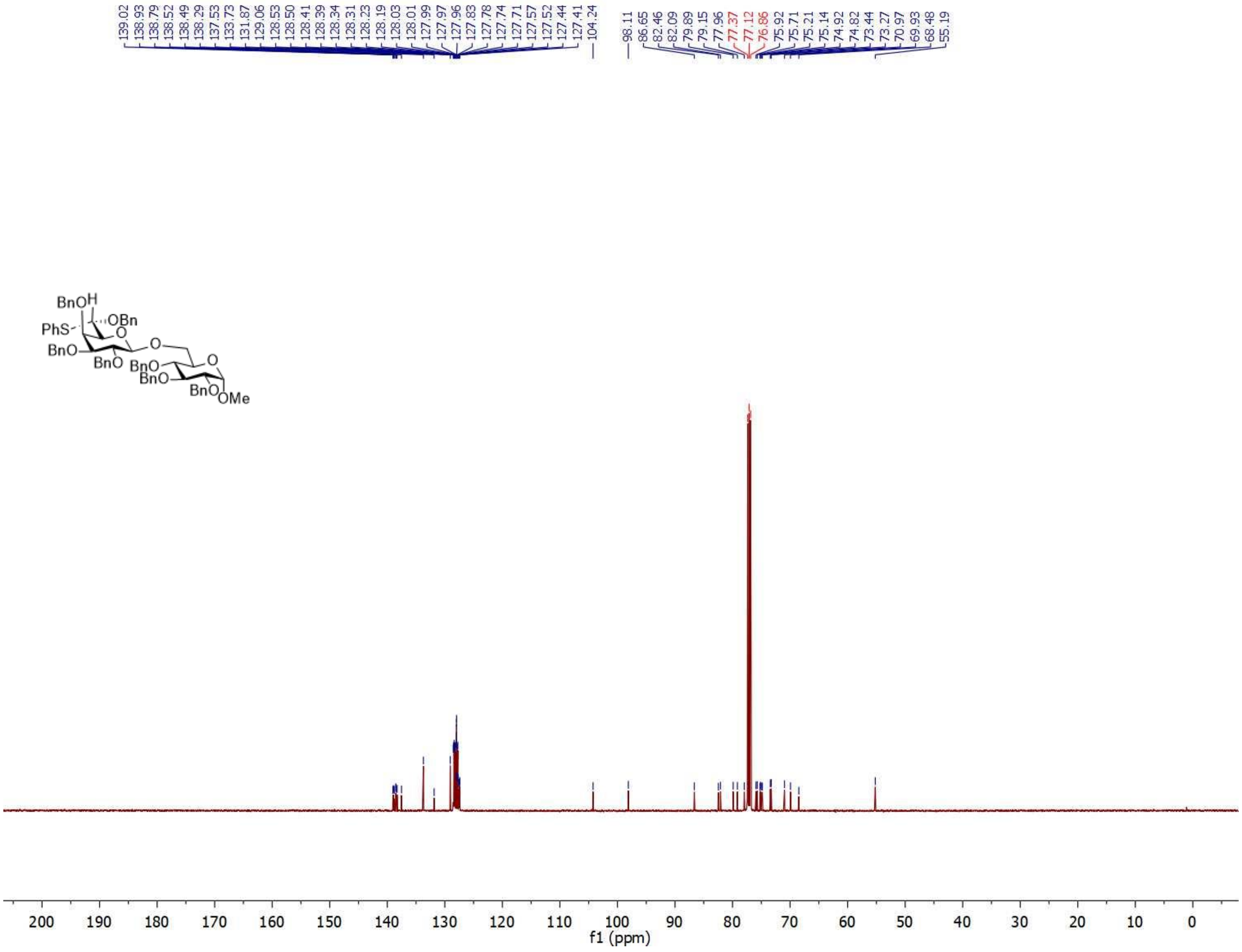

¹H NMR (500 MHz, $CDCl_3$) Spectrum of (6*S*)-6-phenylthio-2,3,4,6-tetra-*O*-benzyl-*α*-D-galactopyranosyl-(1→6)-1,2:3,4-*O*-diisopropylidene-*α*-D-galactopyranose **(15*α*)**

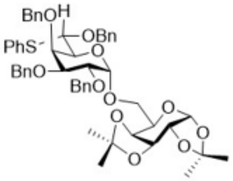

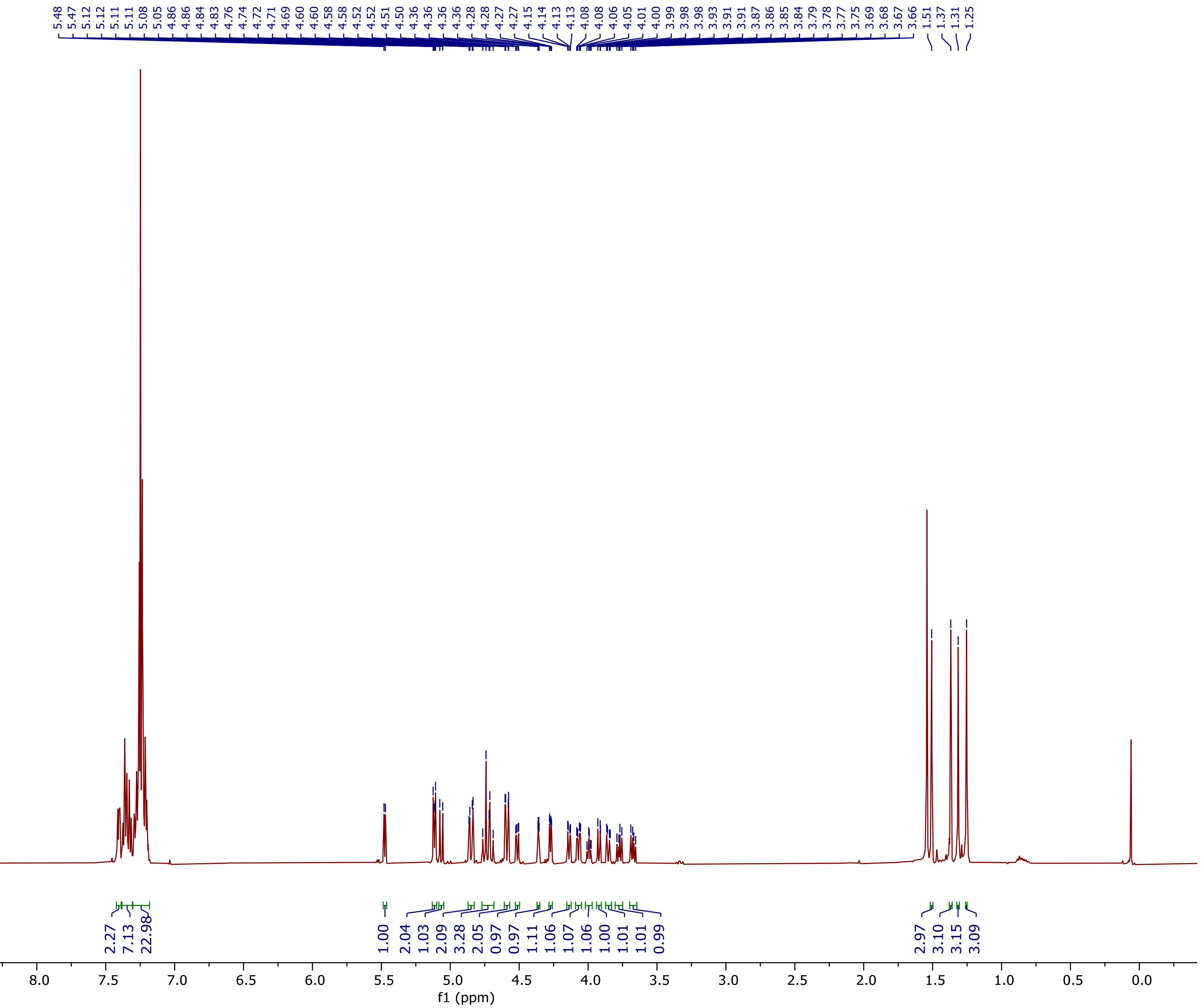

^{1}H NMR (500 MHz, $CDCl_3$) Spectrum of (6*S*)-6-phenylthio-2,3,4,6-tetra-*O*-benzyl-*β*-D-galactopyranosyl-(1→6)-1,2:3,4-*O*-diisopropylidene-*α*-D-galactopyranose **(15*β*)**

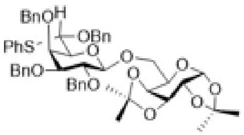

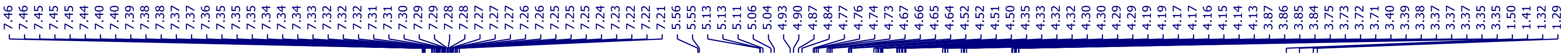

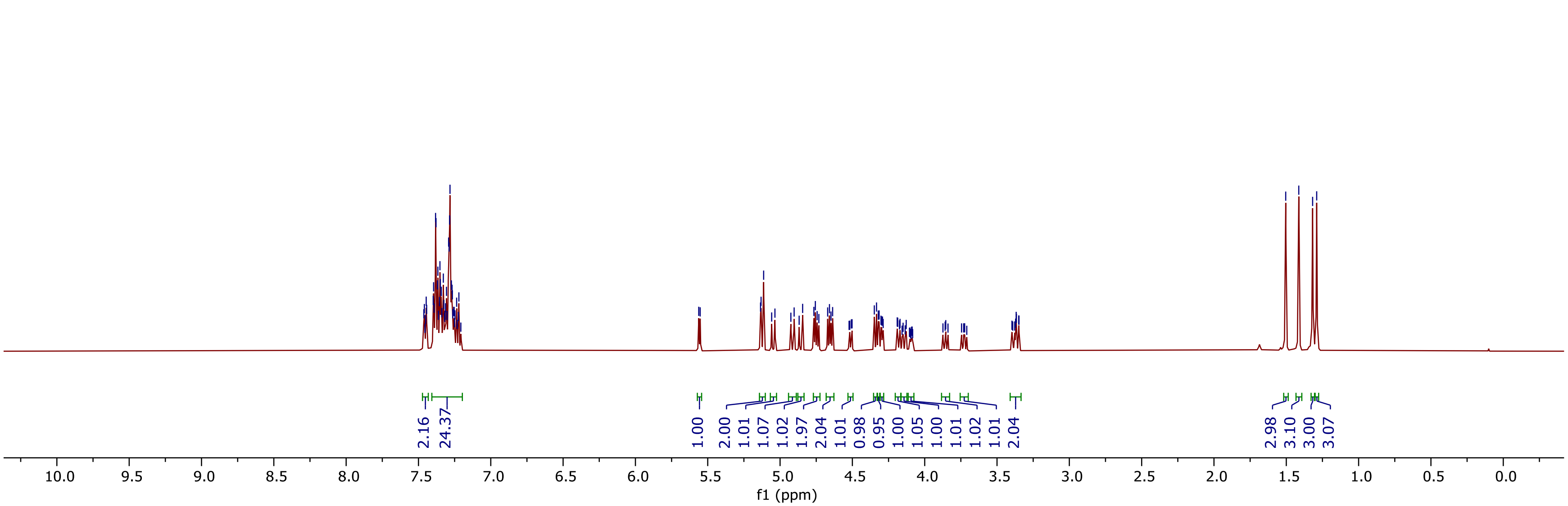

^{13}**C {^{1}H} NMR** (126 MHz, $CDCl_3$) Spectrum of (6*S*)-6-phenylthio-2,3,4,6-tetra-*O*-benzyl-*β*-D-galactopyranosyl-(1→6)-1,2:3,4-*O*-diisopropylidene-*α*-D-galactopyranose **(15*β*)**

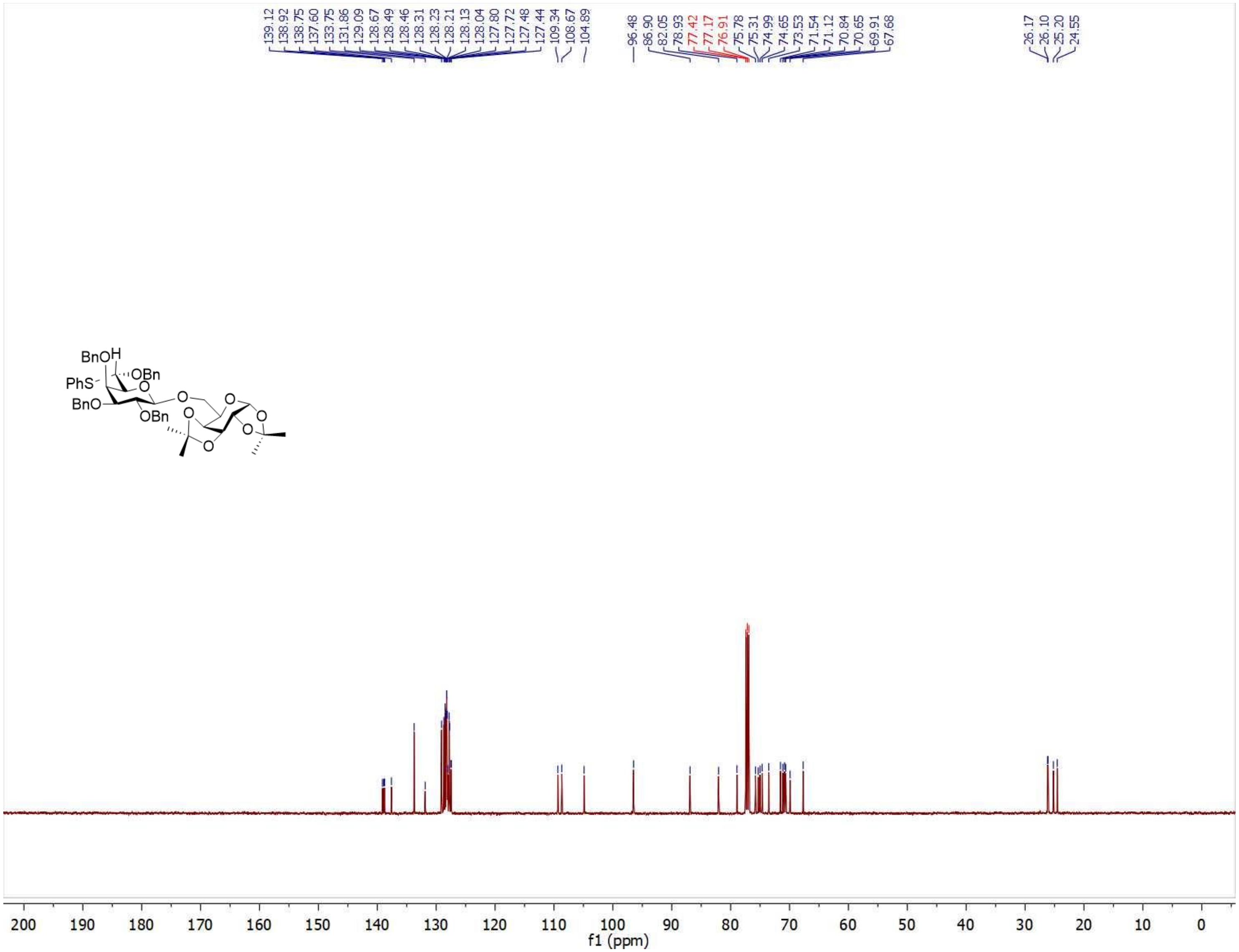

[1]**H NMR** (500 MHz, $CDCl_3$) Spectrum of Adamantyl (6*S*)-6-phenylthio-2,3,4,6-tetra-*O*-benzyl-*α*-D-galactopyranoside **(16*α*)**

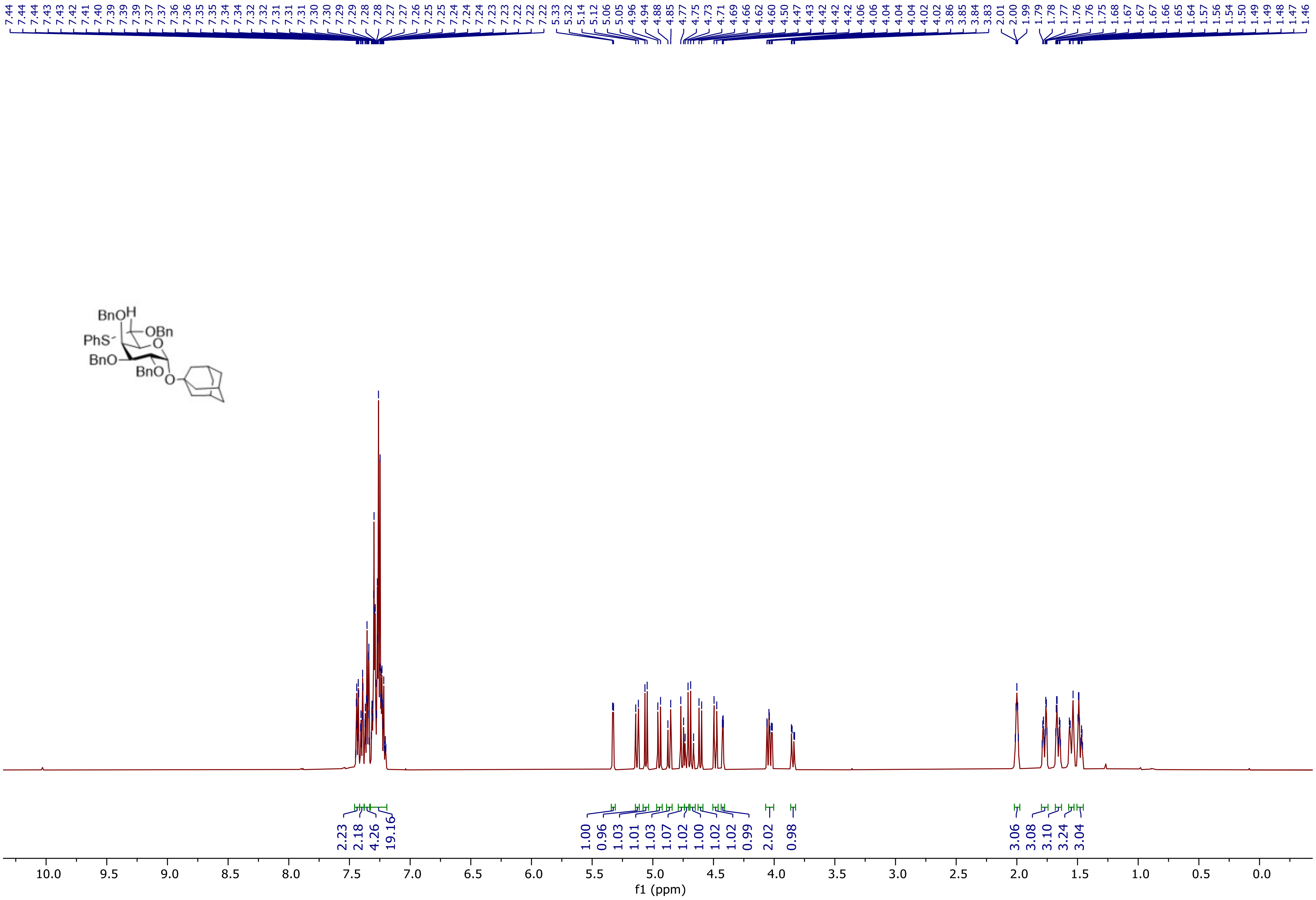

^{13}C {^{1}H} NMR (126 MHz, $CDCl_3$) Spectrum of Adamantyl (6*S*)-6-phenylthio-2,3,4,6-tetra-*O*-benzyl-*α*-D-galactopyranoside **(16*α*)**

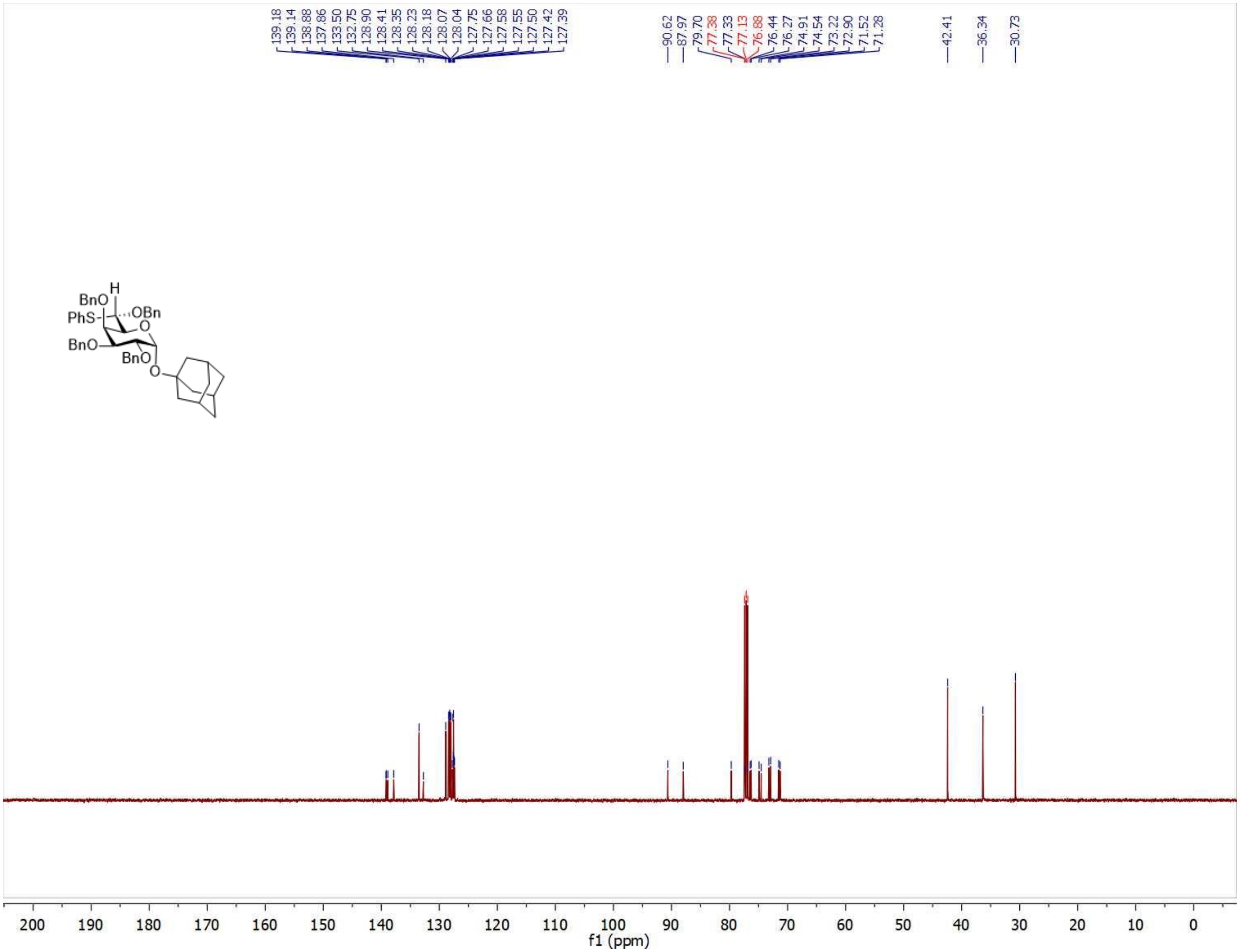

^{1}H NMR (500 MHz, CDCl$_3$) Spectrum of Adamantyl (6*S*)-6-phenylthio-2,3,4,6-tetra-*O*-benzyl-*β*-D-galactopyranoside **(16*β*)**

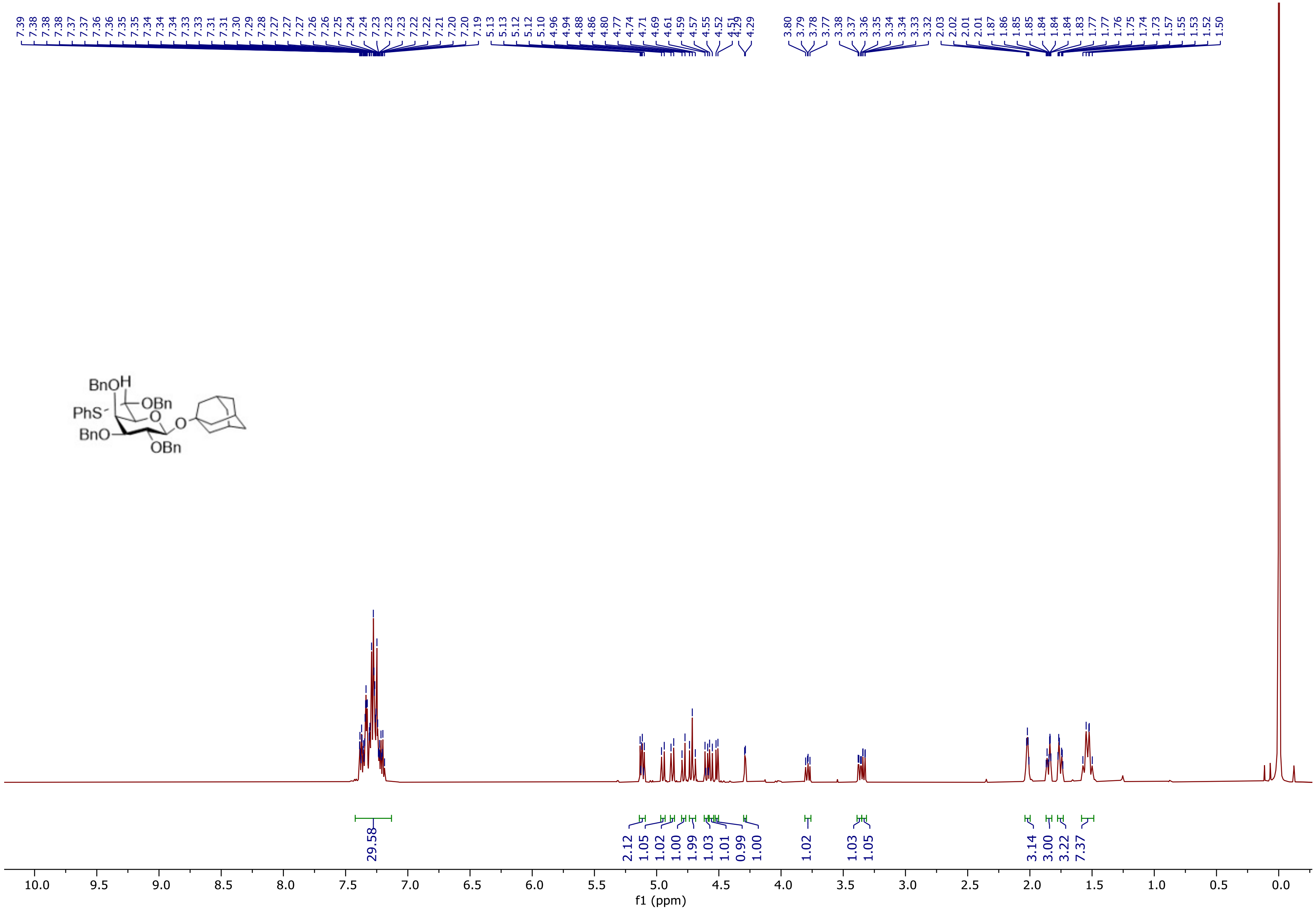

^{13}C {^{1}H} NMR (126 MHz, $CDCl_3$) Spectrum of Adamantyl (6*S*)-6-phenylthio-2,3,4,6-tetra-*O*-benzyl-*β*-D-galactopyranoside **(16*β*)**

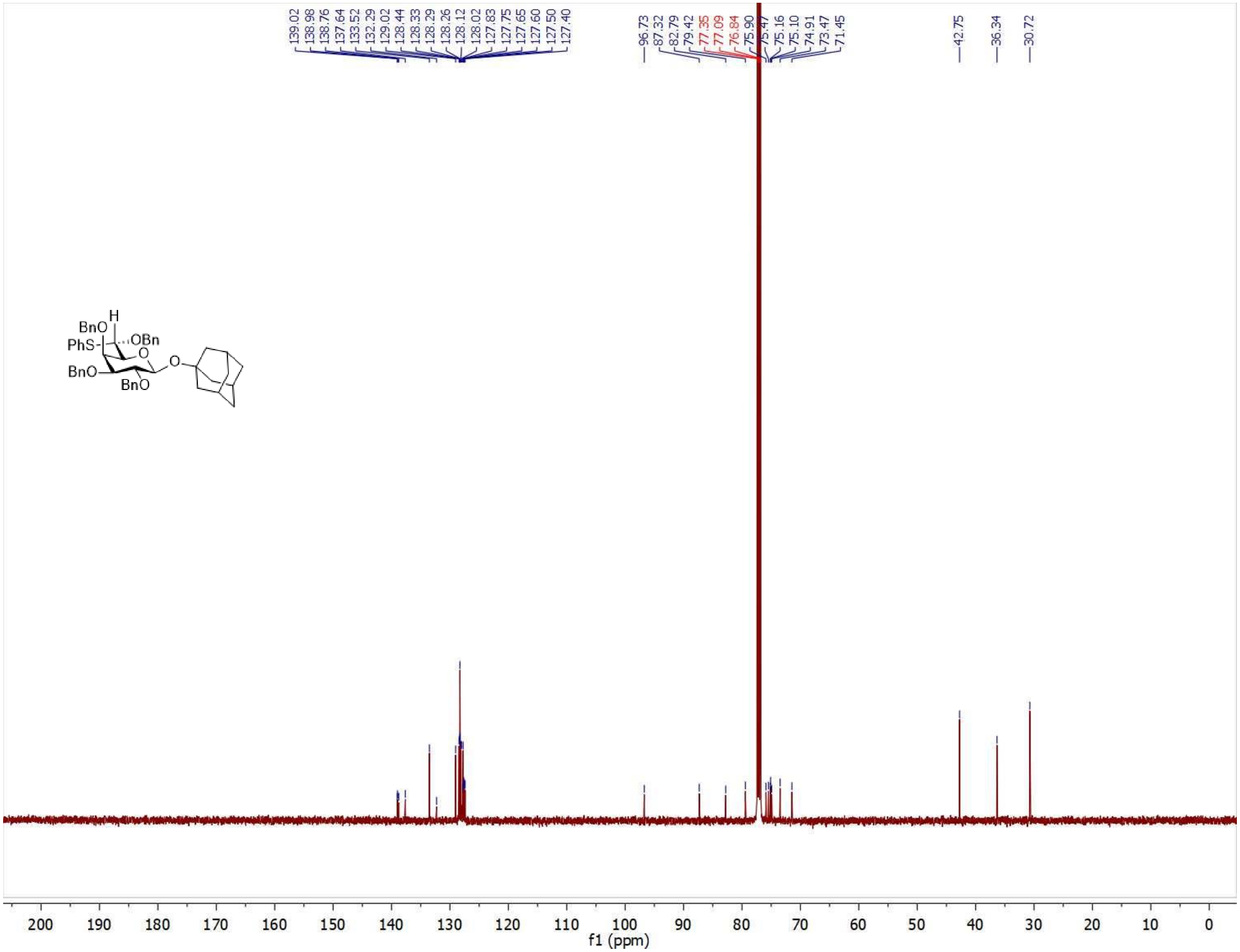

¹H NMR (500 MHz, $CDCl_3$) Spectrum of (6*S*)-6-phenylthio-2,3,4,6-tetra-*O*-benzyl-*α*-D-galactopyranosyl-(1→3)-1,2:5,6-di-*O*-isopropylidene-*α*-D-glucofuranose **(17*α*)**

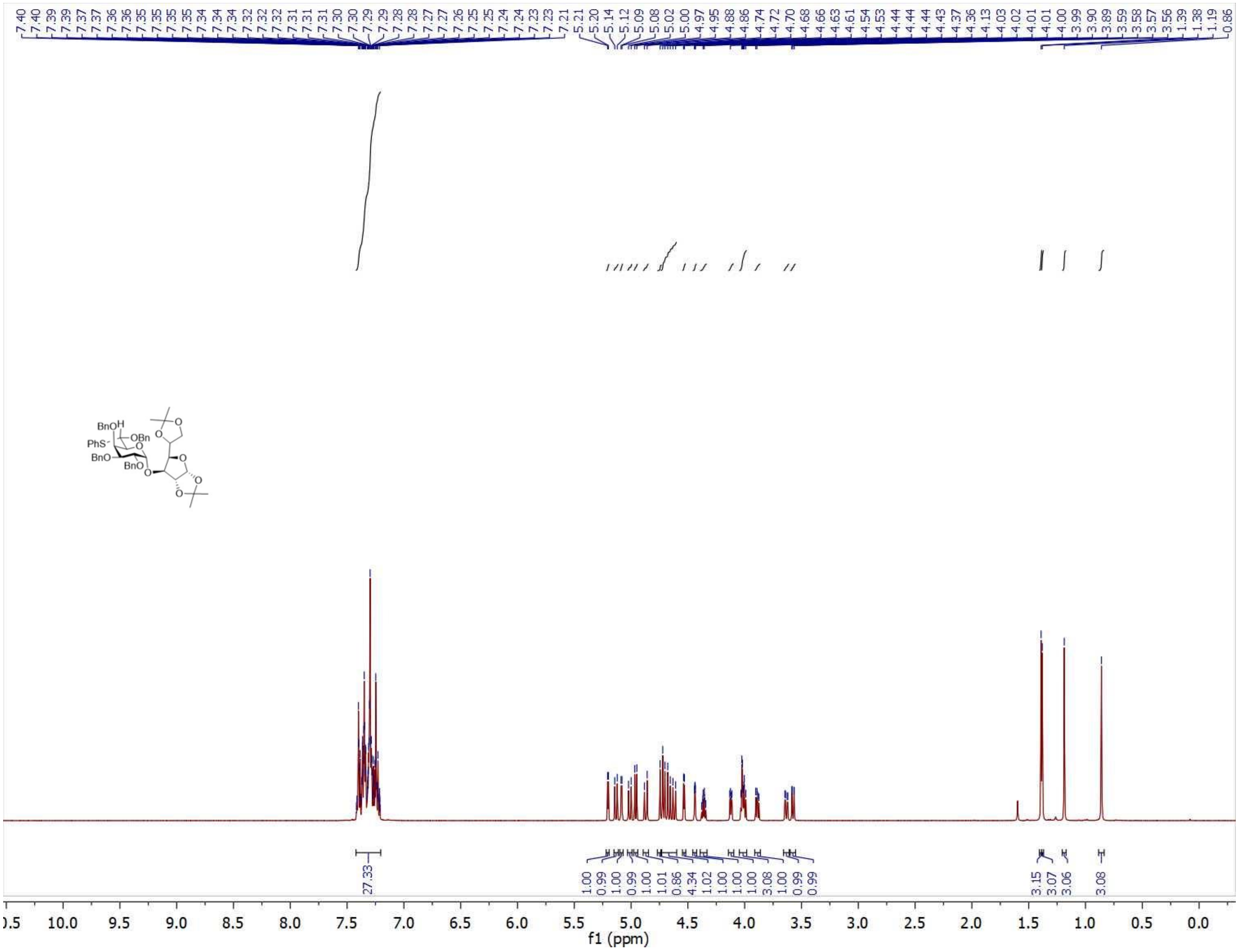

^{13}C {^{1}H} NMR (126 MHz, $CDCl_3$) Spectrum of (6*S*)-6-phenylthio-2,3,4,6-tetra-*O*-benzyl-*α*-D-galactopyranosyl-(1→3)-1,2:5,6-di-*O*-isopropylidene-*α*-D-glucofuranose **(17*α*)**

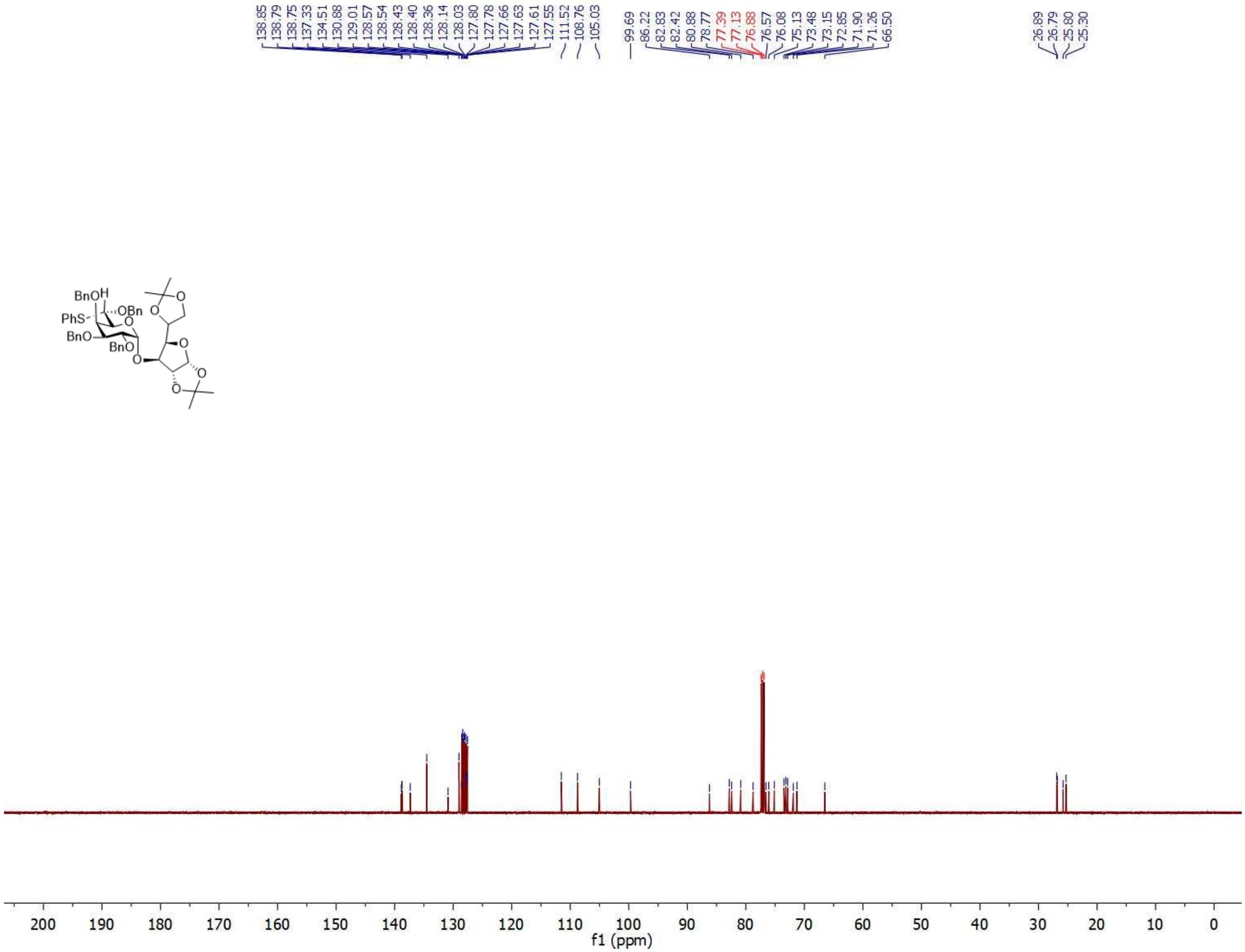

^{1}H NMR (500 MHz, CDCl$_3$) Spectrum of (6*S*)-6-phenylthio-2,3,4,6-tetra-*O*-benzyl-*β*-D-galactopyranosyl-(1→3)-1,2:5,6-di-*O*-isopropylidene-*α*-D-glucofuranose **(17*β*)**

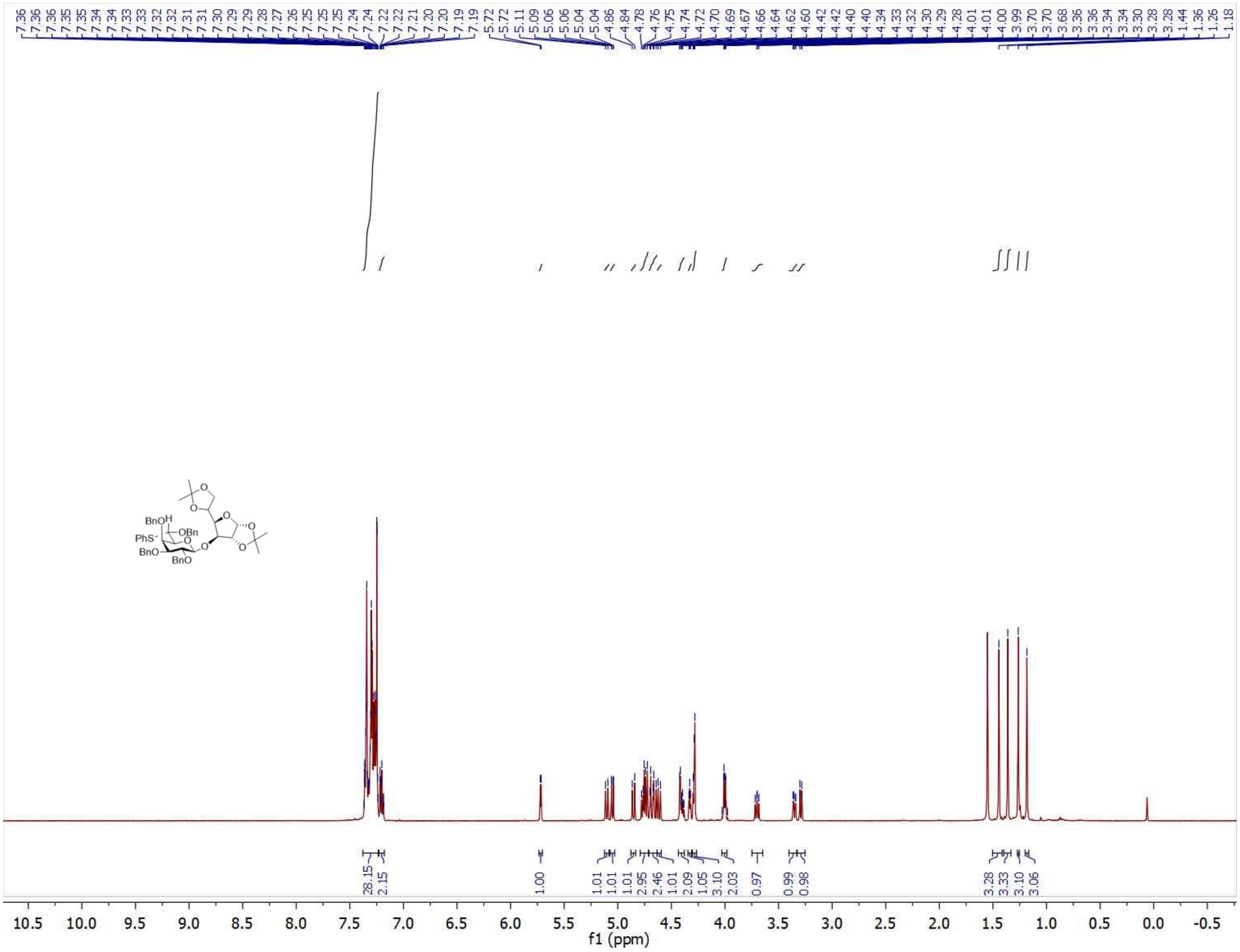

^{13}C {^{1}H} NMR (126 MHz, $CDCl_3$) Spectrum of (6*S*)-6-phenylthio-2,3,4,6-tetra-*O*-benzyl-*β*-D-galactopyranosyl-(1→3)-1,2:5,6-di-*O*-isopropylidene-*α*-D-glucofuranose **(17*β*)**

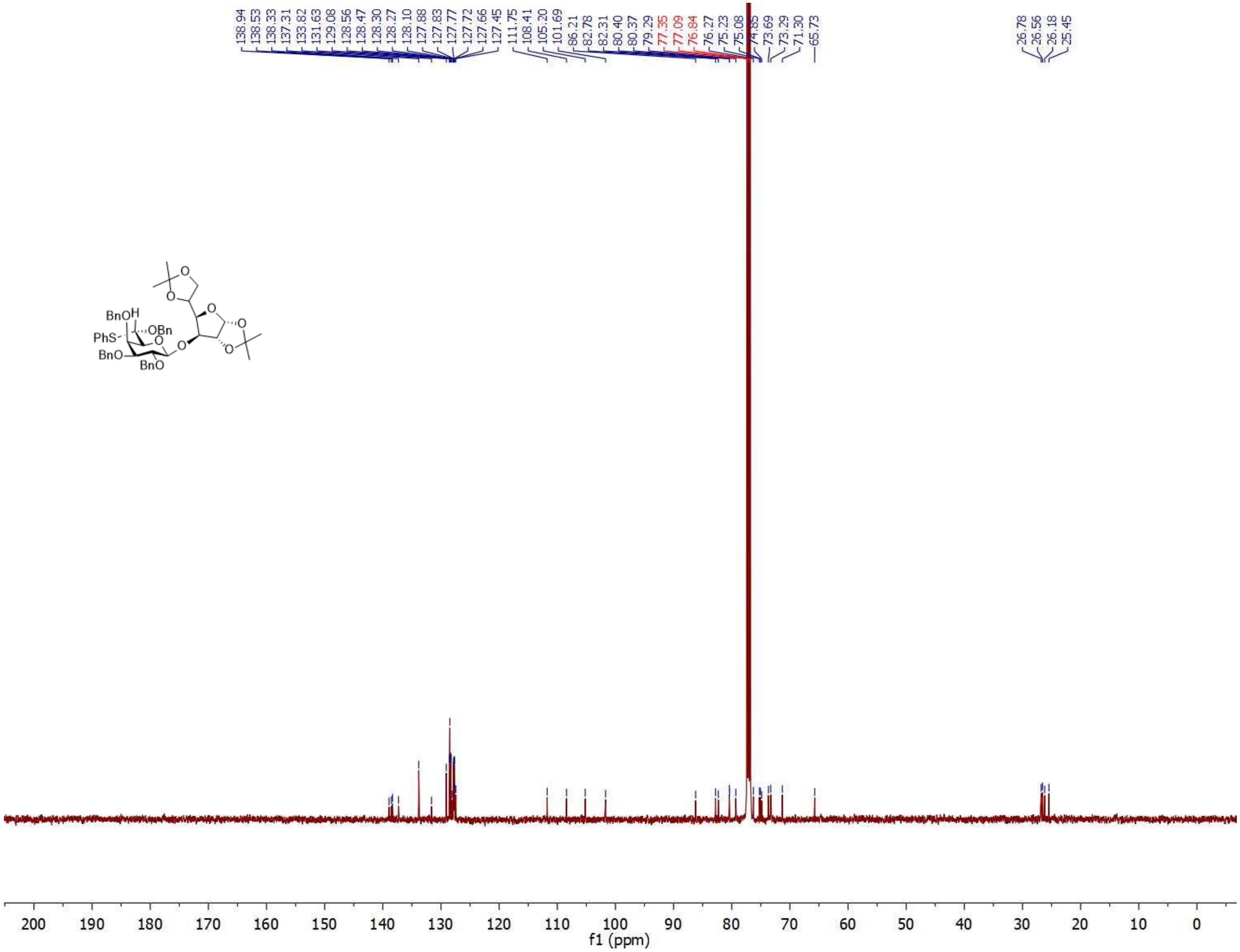

^{1}H NMR (500 MHz, $CDCl_3$) Spectrum of Methyl (6*S*)-6-phenylthio-2,3,4,6-tetra-*O*-benzyl-*α*-D-galactopyranosyl-(1→4)-2,3-*O*-isopropylidene-*α*-L-rhamnopyranoside **(18*α*)**

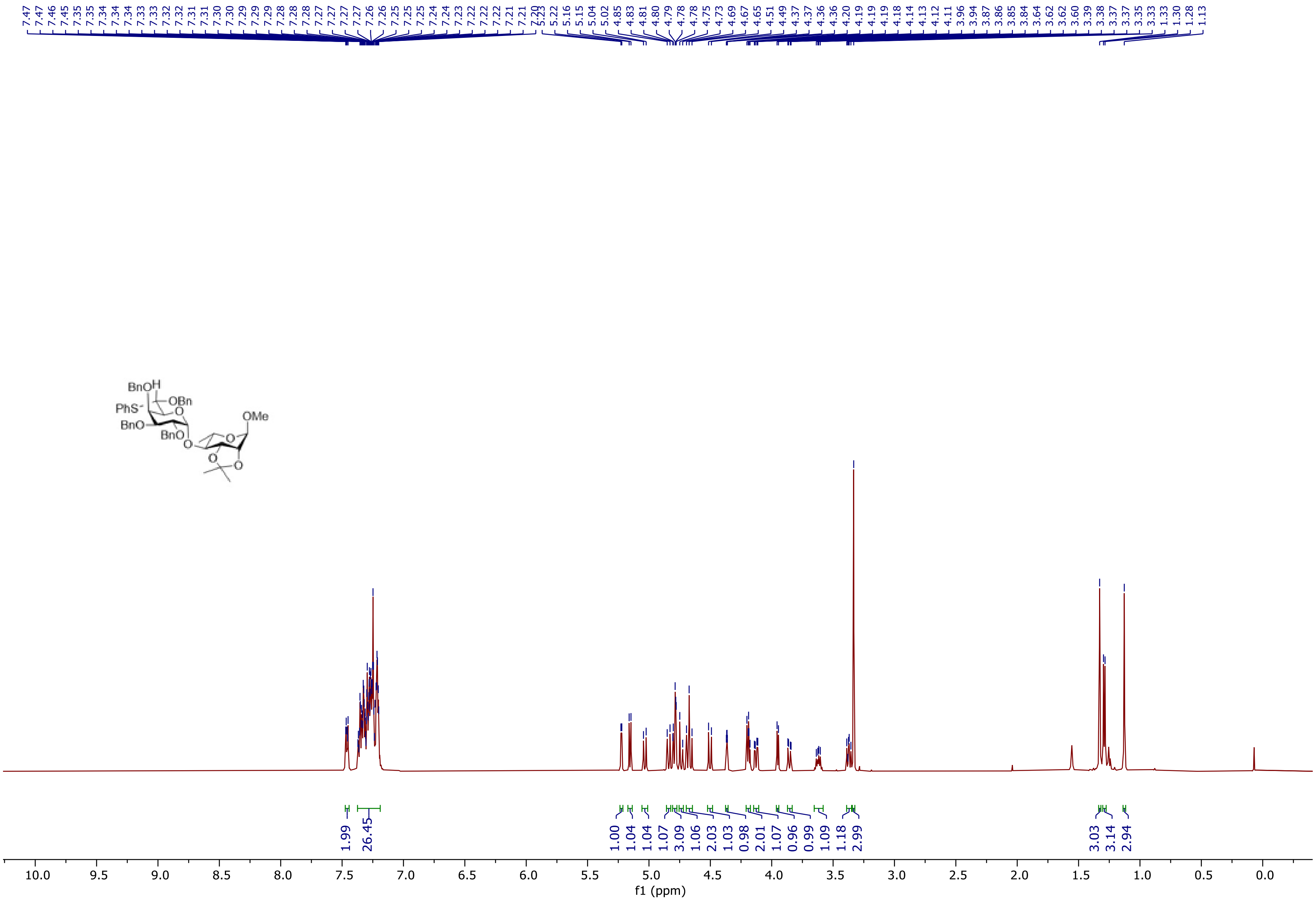

^{13}C {^{1}H} NMR (126 MHz, $CDCl_3$) Spectrum of Methyl (6*S*)-6-phenylthio-2,3,4,6-tetra-*O*-benzyl-*α*-D-galactopyranosyl-(1→4)-2,3-*O*-isopropylidene-*α*-L-rhamnopyranoside **(18*α*)**

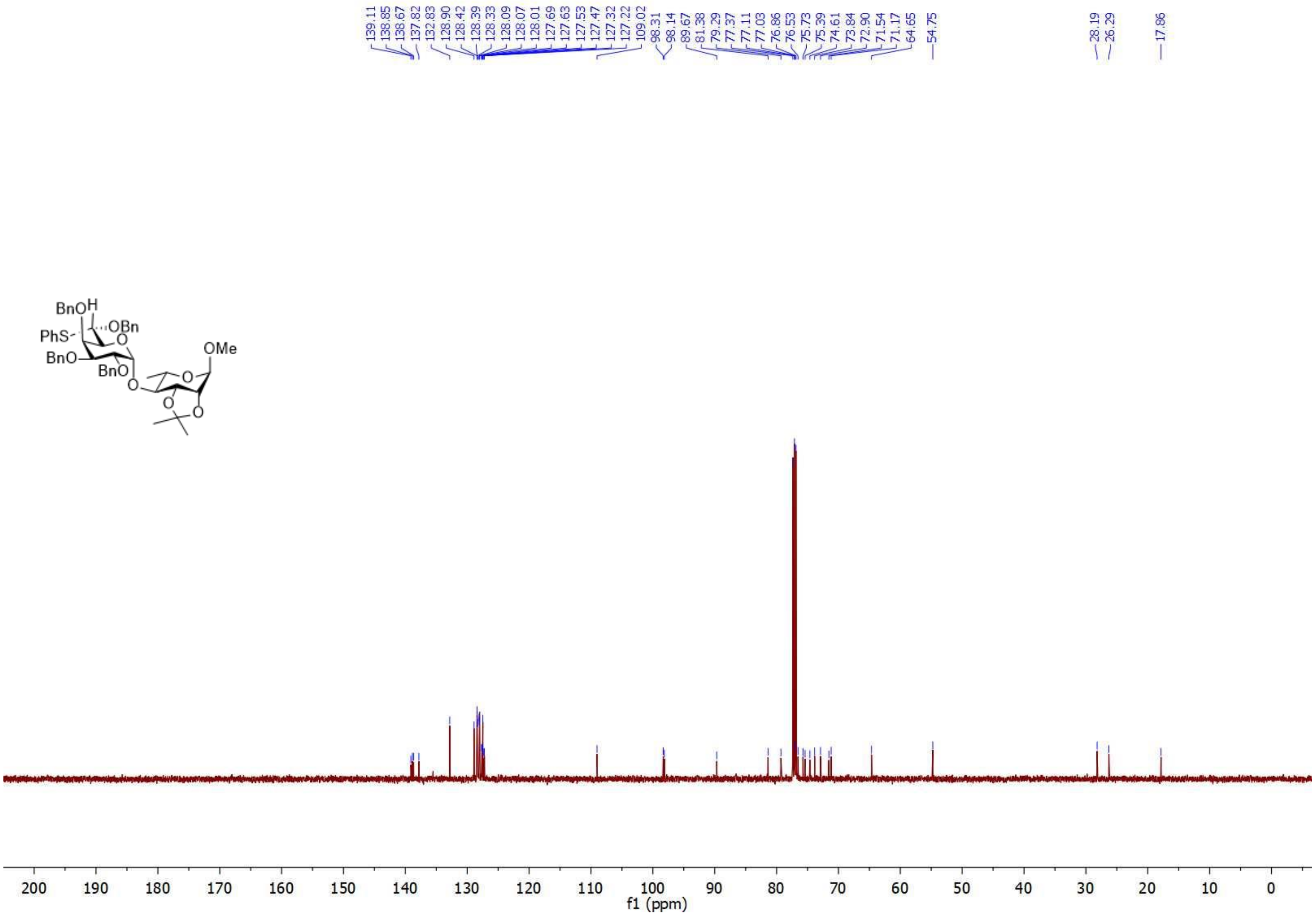

^{1}H NMR (500 MHz, CDCl$_3$) Spectrum of Methyl (6*S*)-6-phenylthio-2,3,4,6-tetra-*O*-benzyl-*β*-D-galactopyranosyl-(1→4)-2,3-*O*-isopropylidene-*α*-L-rhamnopyranoside **(18*β*)**

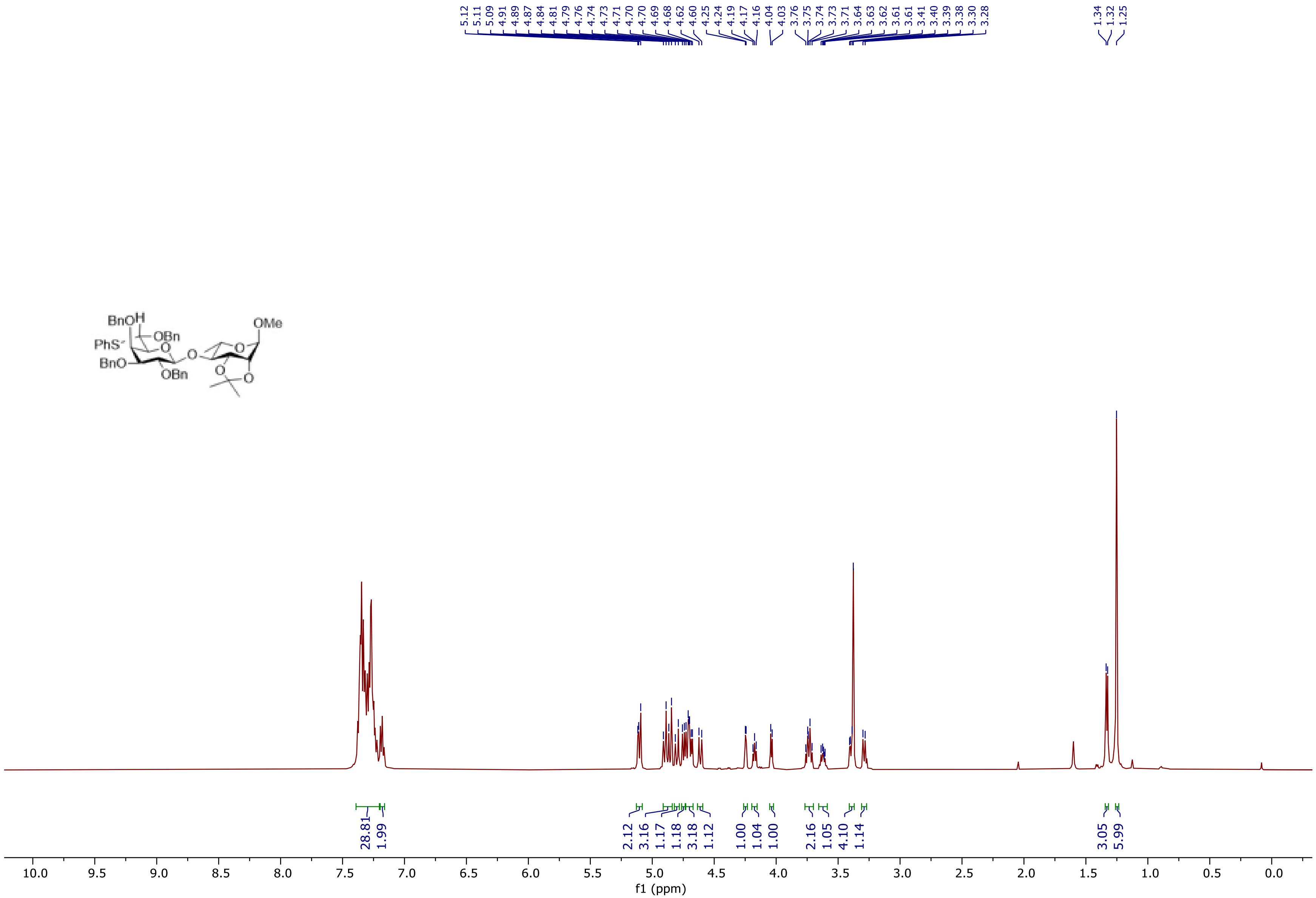

^{13}C {^{1}H} NMR (126 MHz, $CDCl_3$) Spectrum of Methyl (6*S*)-6-phenylthio-2,3,4,6-tetra-*O*-benzyl-*β*-D-galactopyranosyl-(1→4)-2,3-*O*-isopropylidene-*α*-L-rhamnopyranoside **(18*β*)**

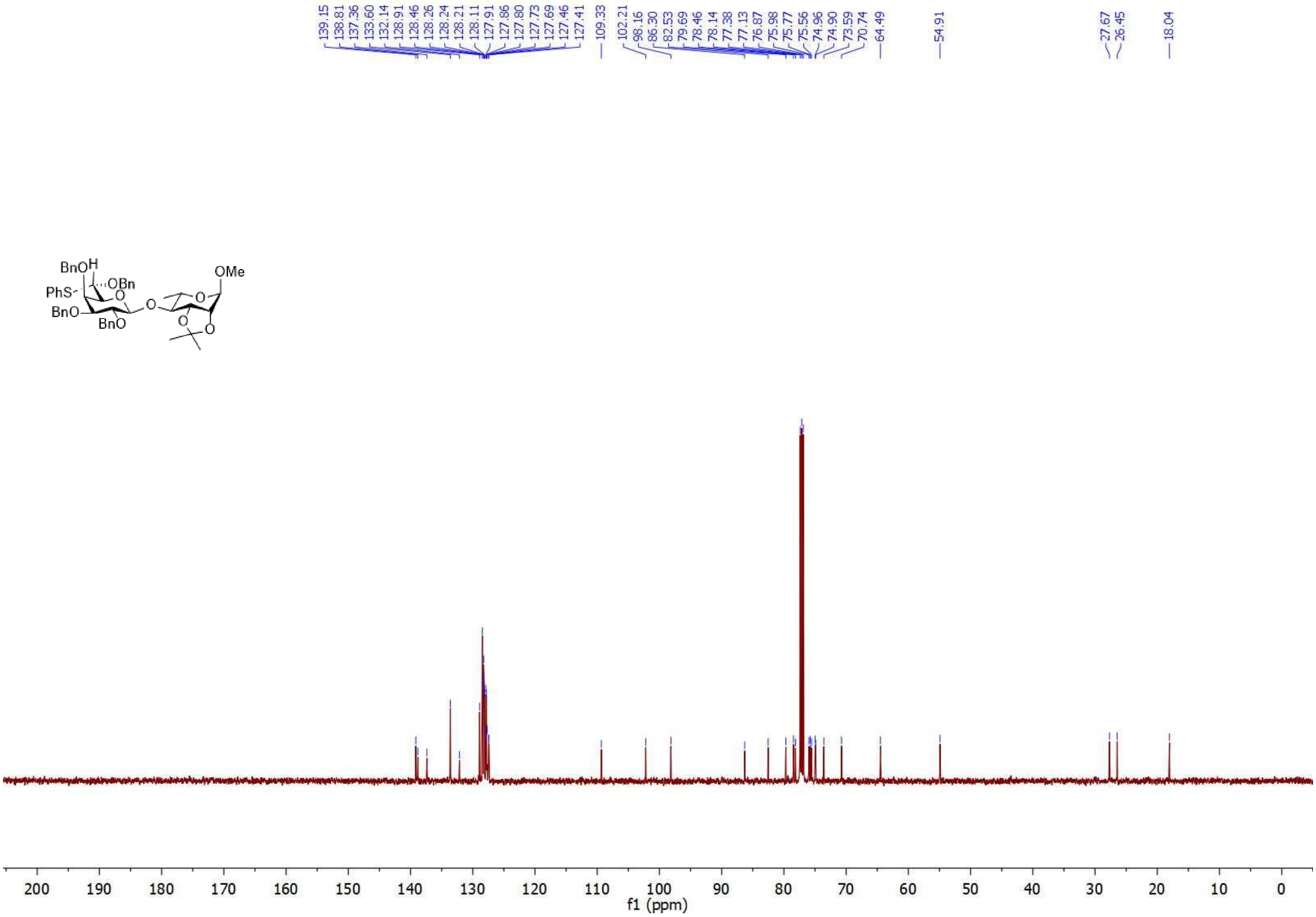

^{1}H NMR (500 MHz, $CDCl_3$) Spectrum of Methyl (6*S*)-6-phenylthio-2,3,4,6-tetra-*O*-benzyl-*α*-D-galactopyranosyl-(1→4)-2,3,4-tri-*O*-benzyl-*α*-D-glucopyranoside **(19*α*)**

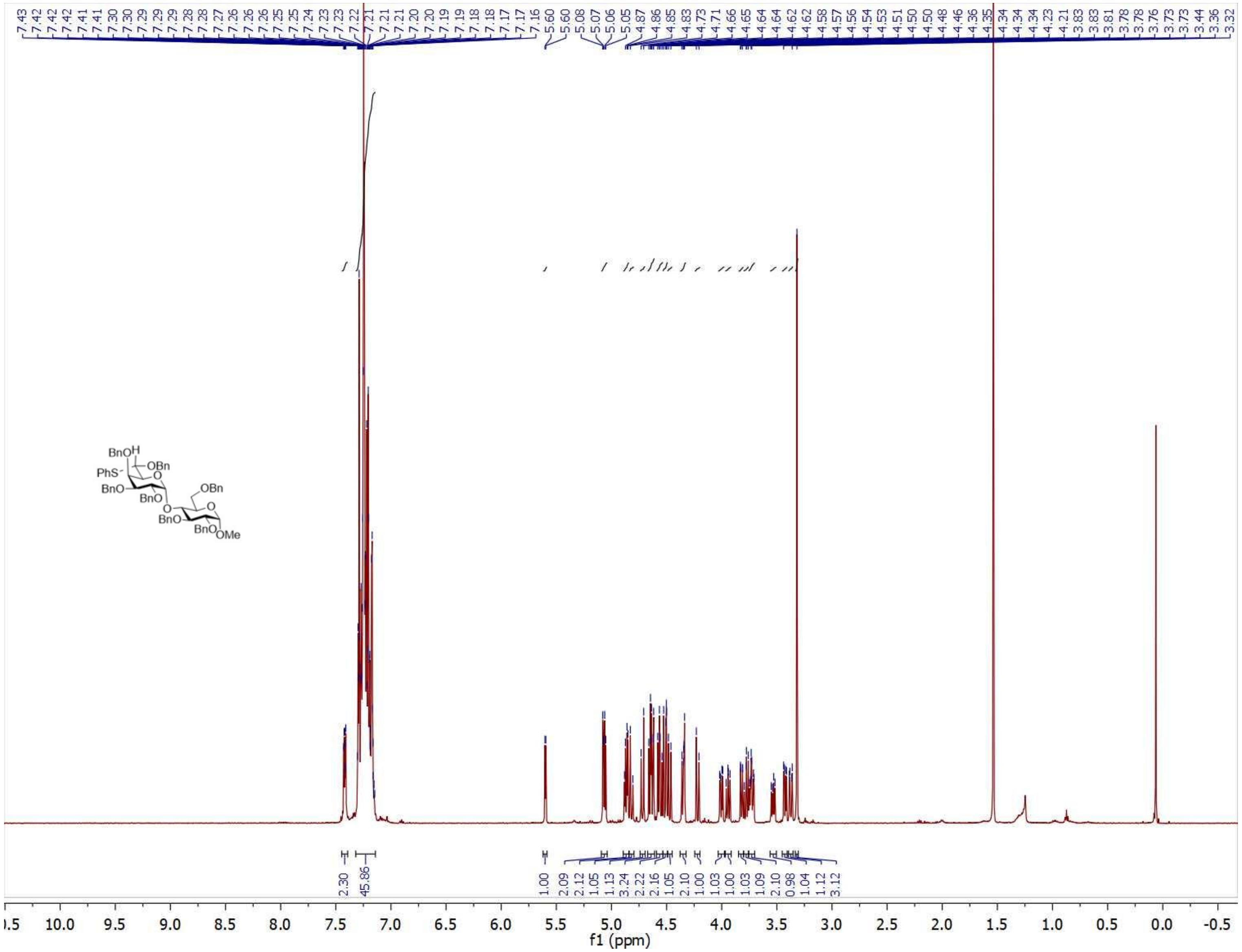

^{13}C {^{1}H} NMR (126 MHz, $CDCl_3$) Spectrum of Methyl (6*S*)-6-phenylthio-2,3,4,6-tetra-*O*-benzyl-*α*-D-galactopyranosyl-(1→4)-2,3,4-tri-*O*-benzyl-*α*-D-glucopyranoside **(19*α*)**

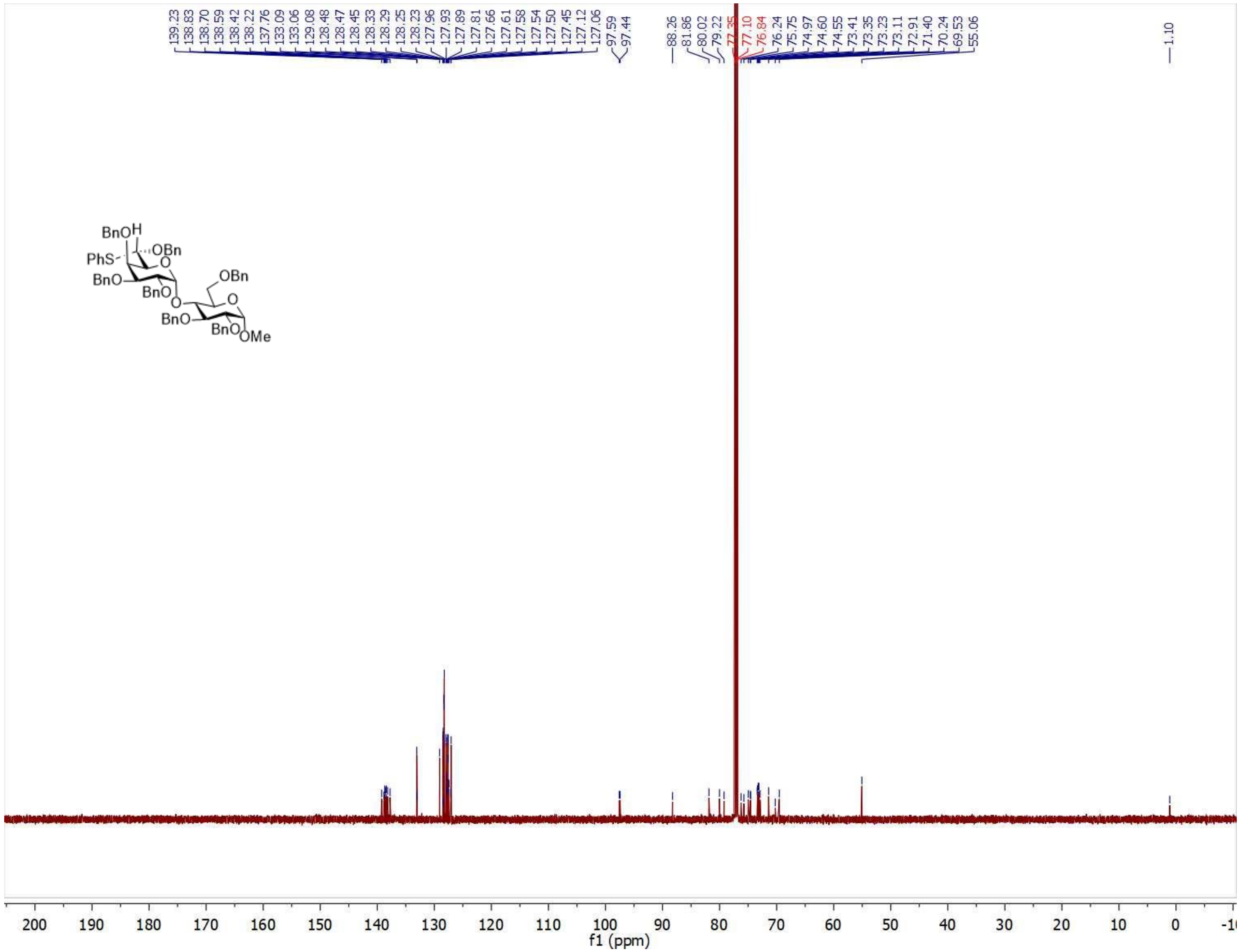

^{1}H NMR (500 MHz, CDCl$_3$) Spectrum of mixture of anomers enriched in **19β** Methyl (6*S*)-6-phenylthio-2,3,4,6-tetra-*O*-benzyl-*β*-D-galactopyranosyl-(1→4)-2,3,4-tri-*O*-benzyl-*α*-D-glucopyranoside

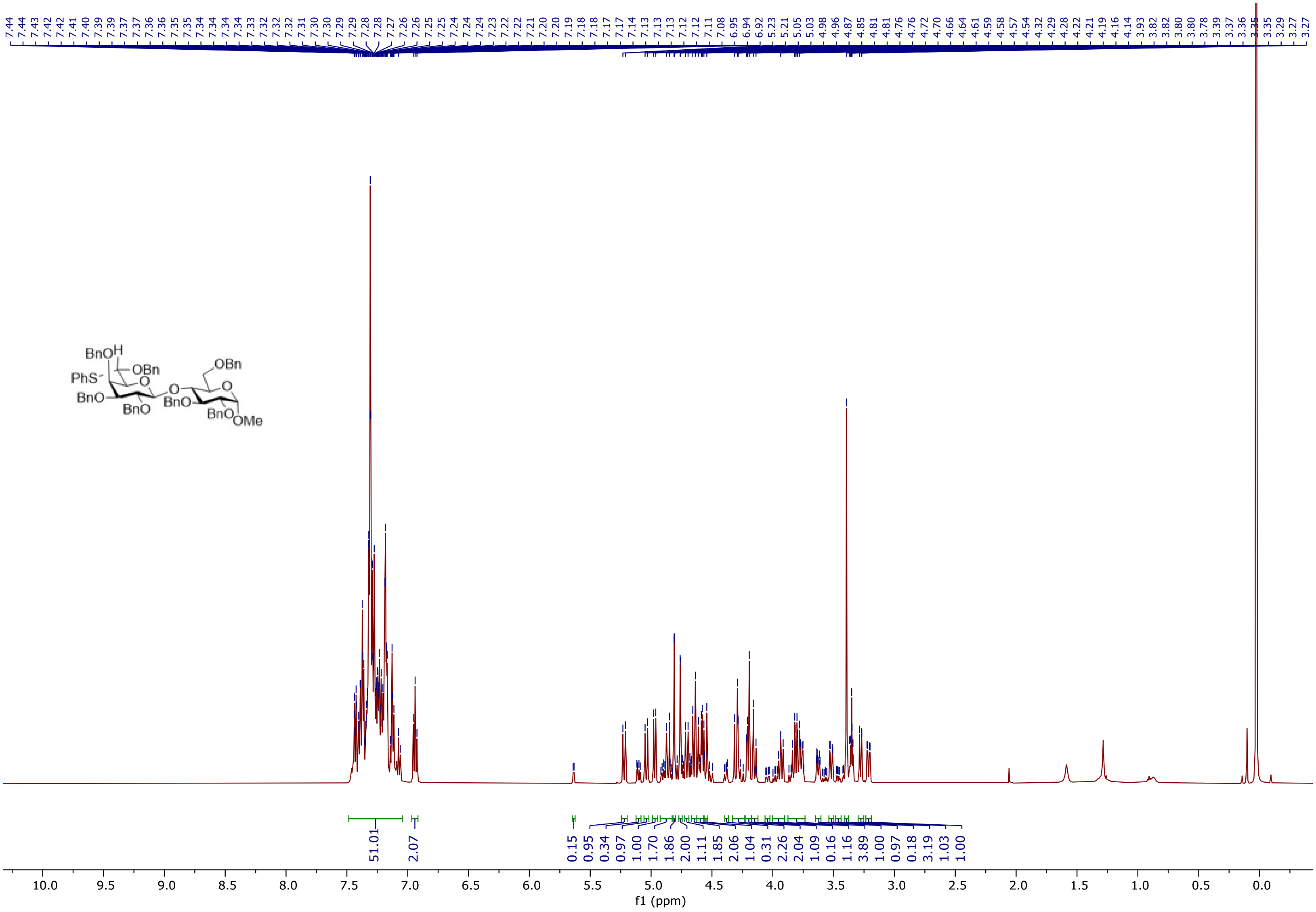

^{13}C {^{1}H} NMR (126 MHz, $CDCl_3$) Spectrum of **19α** and **19β** enriched in **19β** Methyl (6*S*)-6-phenylthio-2,3,4,6-tetra-*O*-benzyl-*β*-D-galactopyranosyl-(1→4)-2,3,4-tri-*O*-benzyl-*α*-D-glucopyranoside

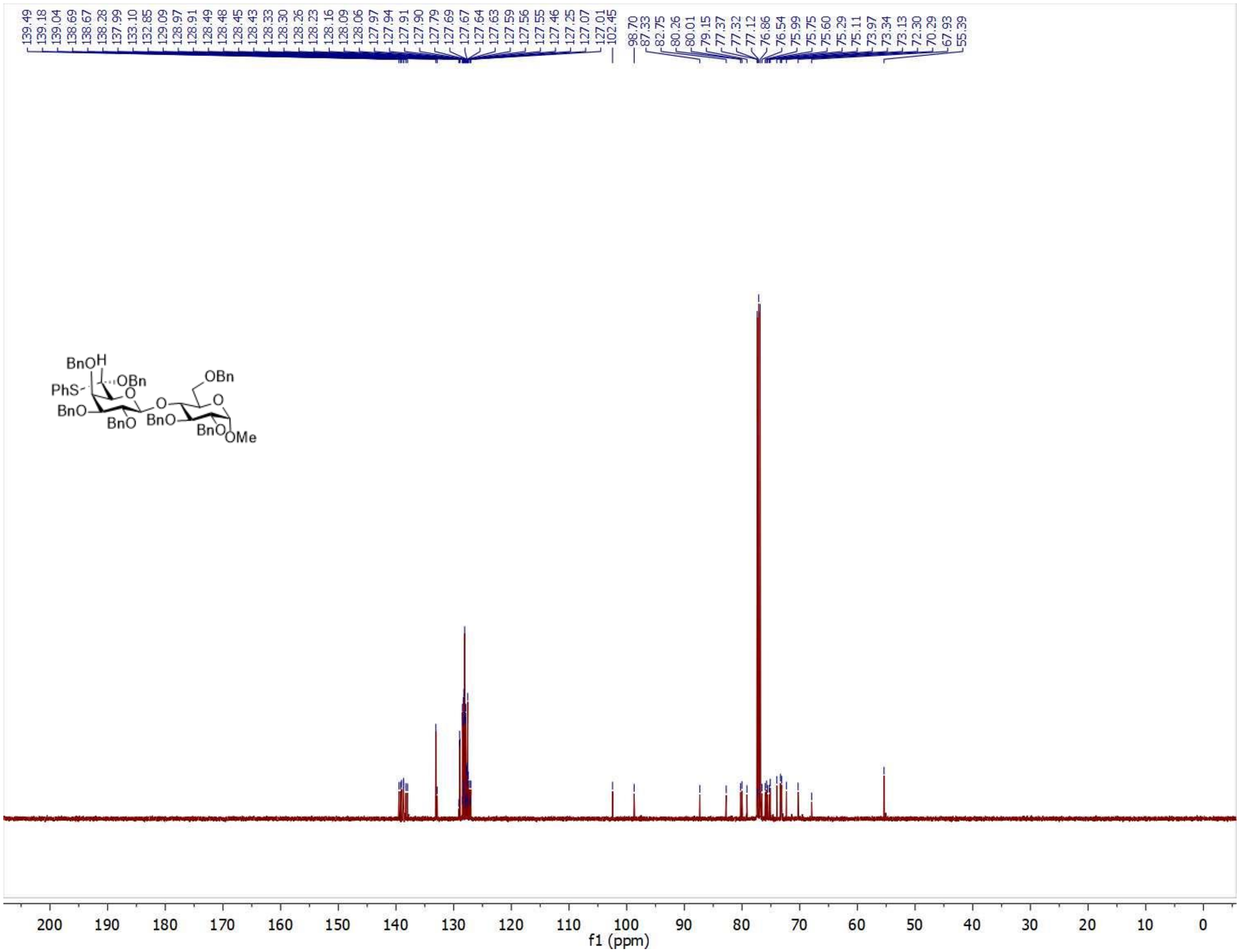

^{1}H NMR (500 MHz, $CDCl_3$) Spectrum of Methyl (6*S*)-6-phenylthio-2,3,4,6-tetra-*O*-benzyl-*β*-D-glucopyranosyl-(1→6)-2,3,4-tri-*O*-benzyl α-D-glucopyranoside **(20β)**

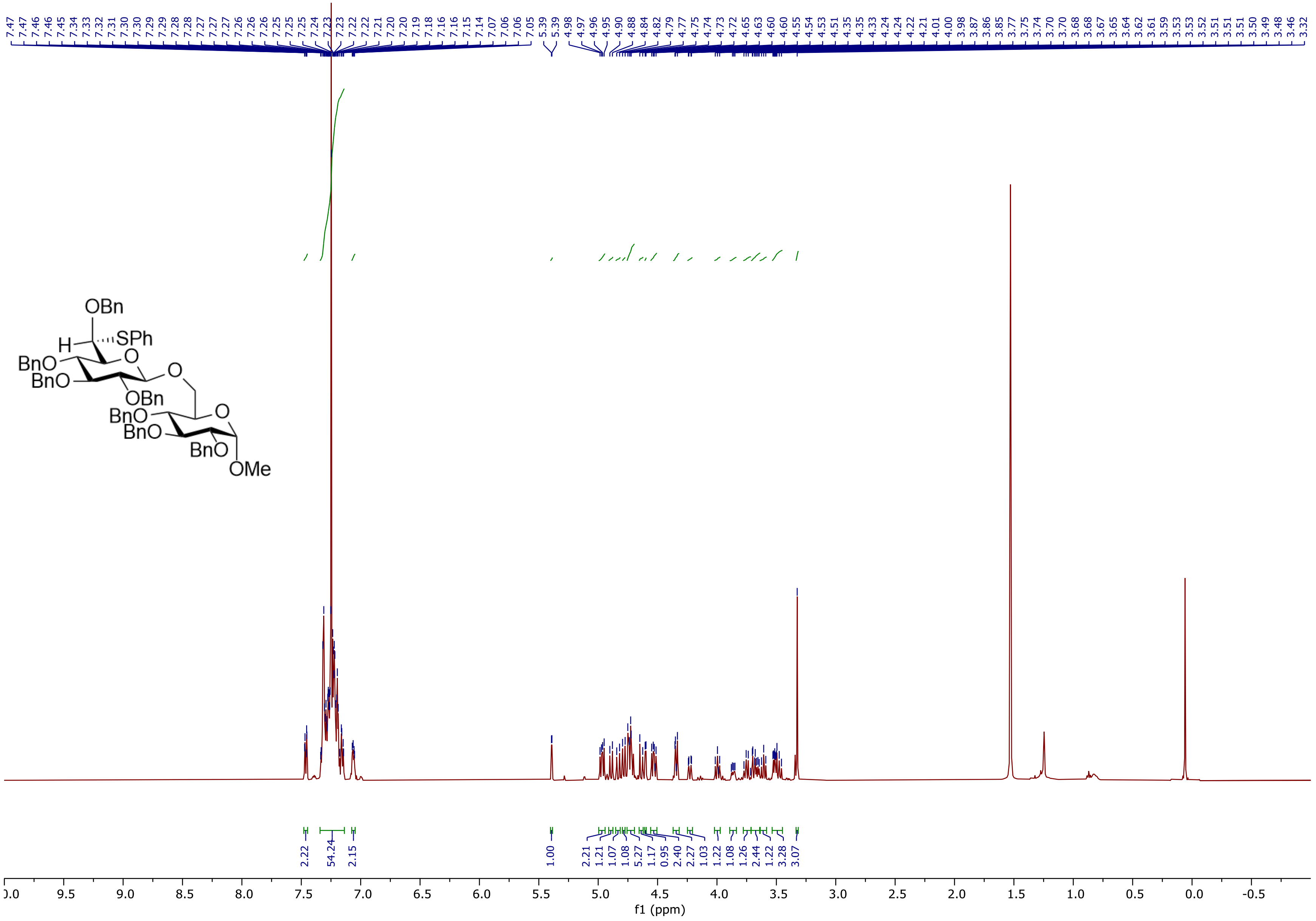

$^{13}C\{^{1}H\}$ NMR (126 MHz, $CDCl_3$) Spectrum of Methyl (6*S*)-6-phenylthio-2,3,4,6-tetra-*O*-benzyl-*β*-D-glucopyranosyl-(1→6)-2,3,4-tri-*O*-benzyl α-D-glucopyranoside **(20β).**

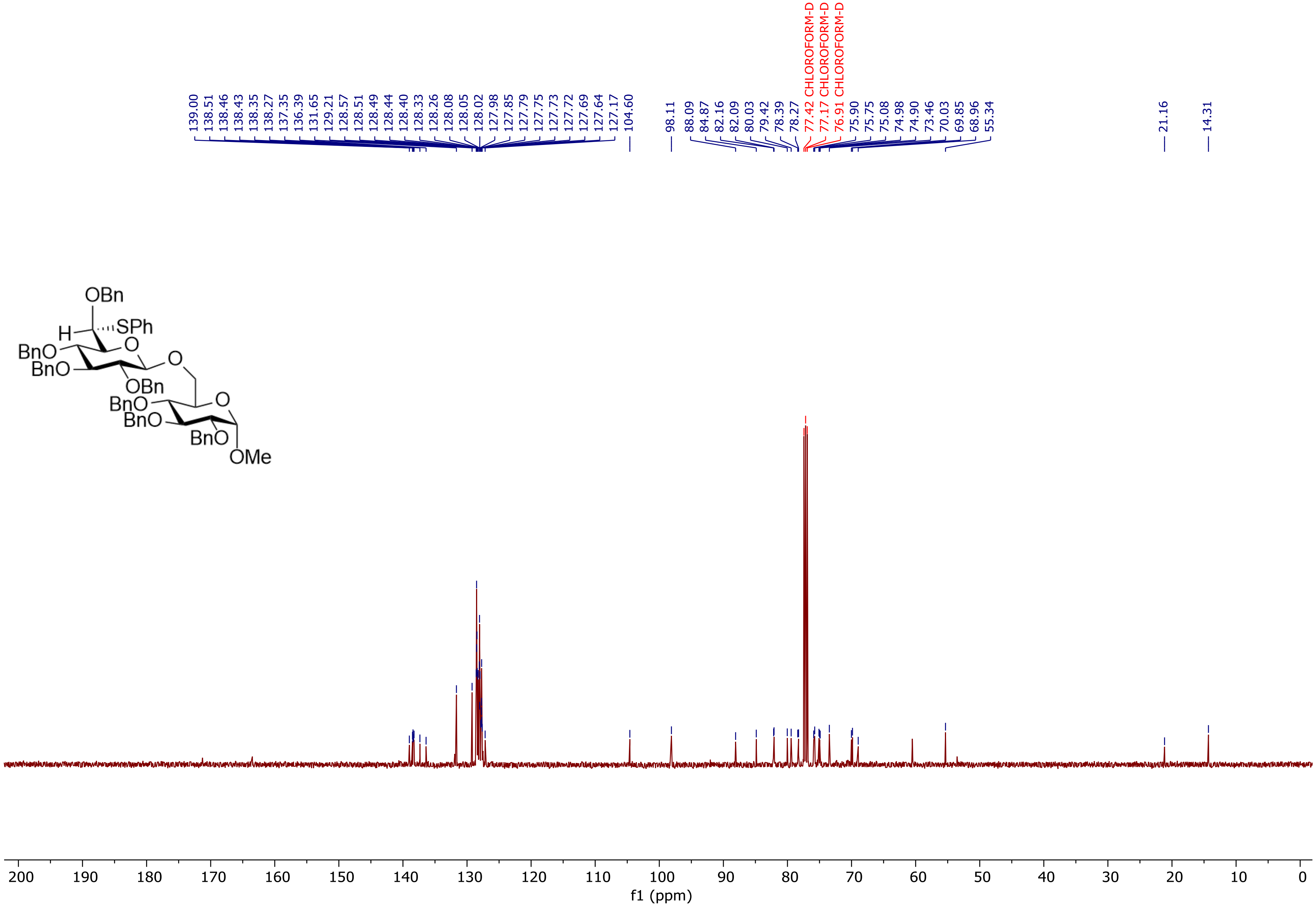

^{1}H NMR (500 MHz, $CDCl_3$) Spectrum of **20α** and **20β** Methyl (6*S*)-6-phenylthio-2,3,4,6-tetra-*O*-benzyl-*β*-D-galactopyranosyl-(1→4)-2,3,6-tri-*O*-benzyl-α-D-glucopyranoside.

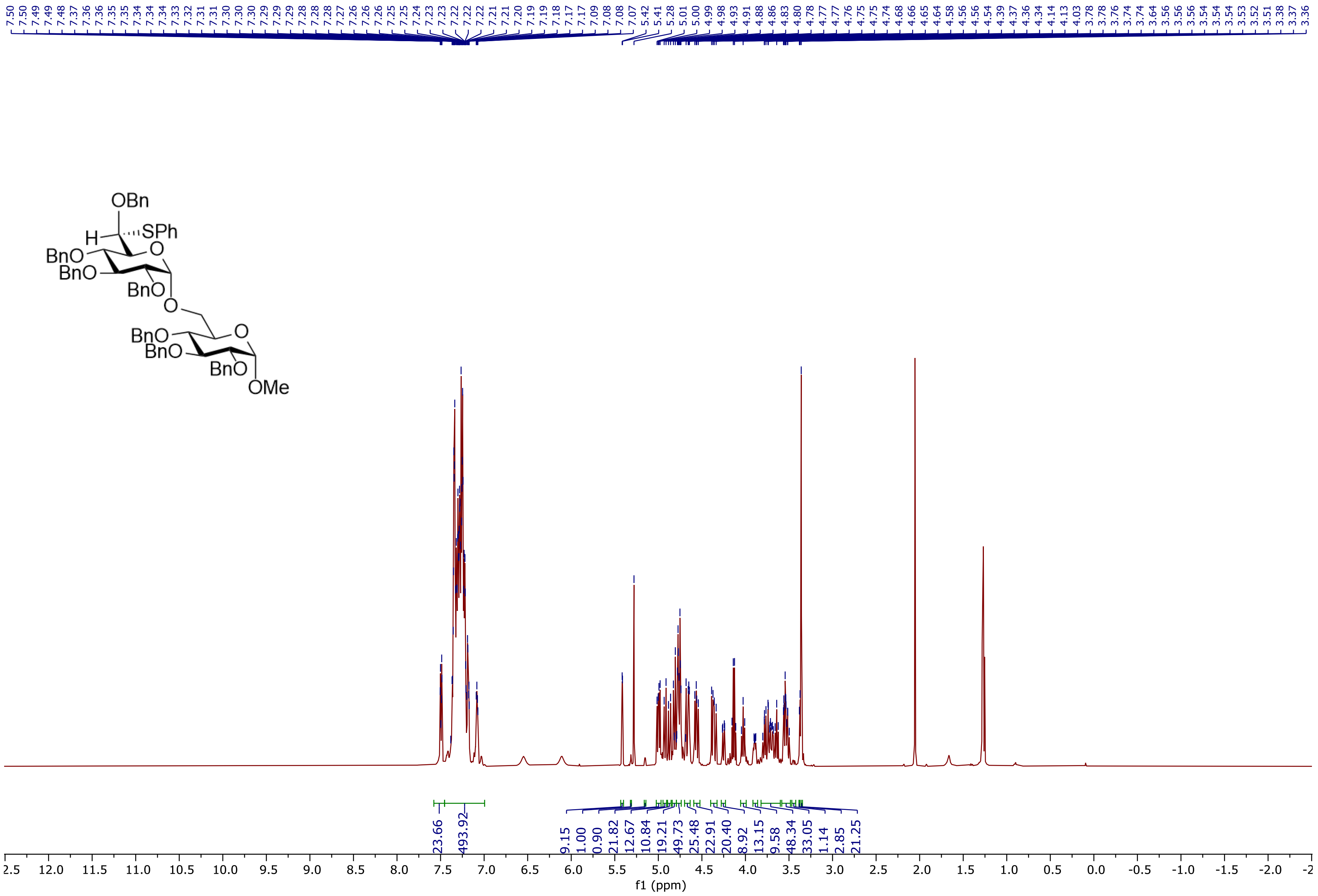

¹H NMR (500 MHz, $CDCl_3$) Spectrum of (6*S*)-6-phenylthio-2,3,4,6-tetra-*O*-benzyl-*α*-D-glucopyranosyl-(1→6)-1,2:3,4-*O*-diisopropylidene-α-D-galactopyranose **(21α)**

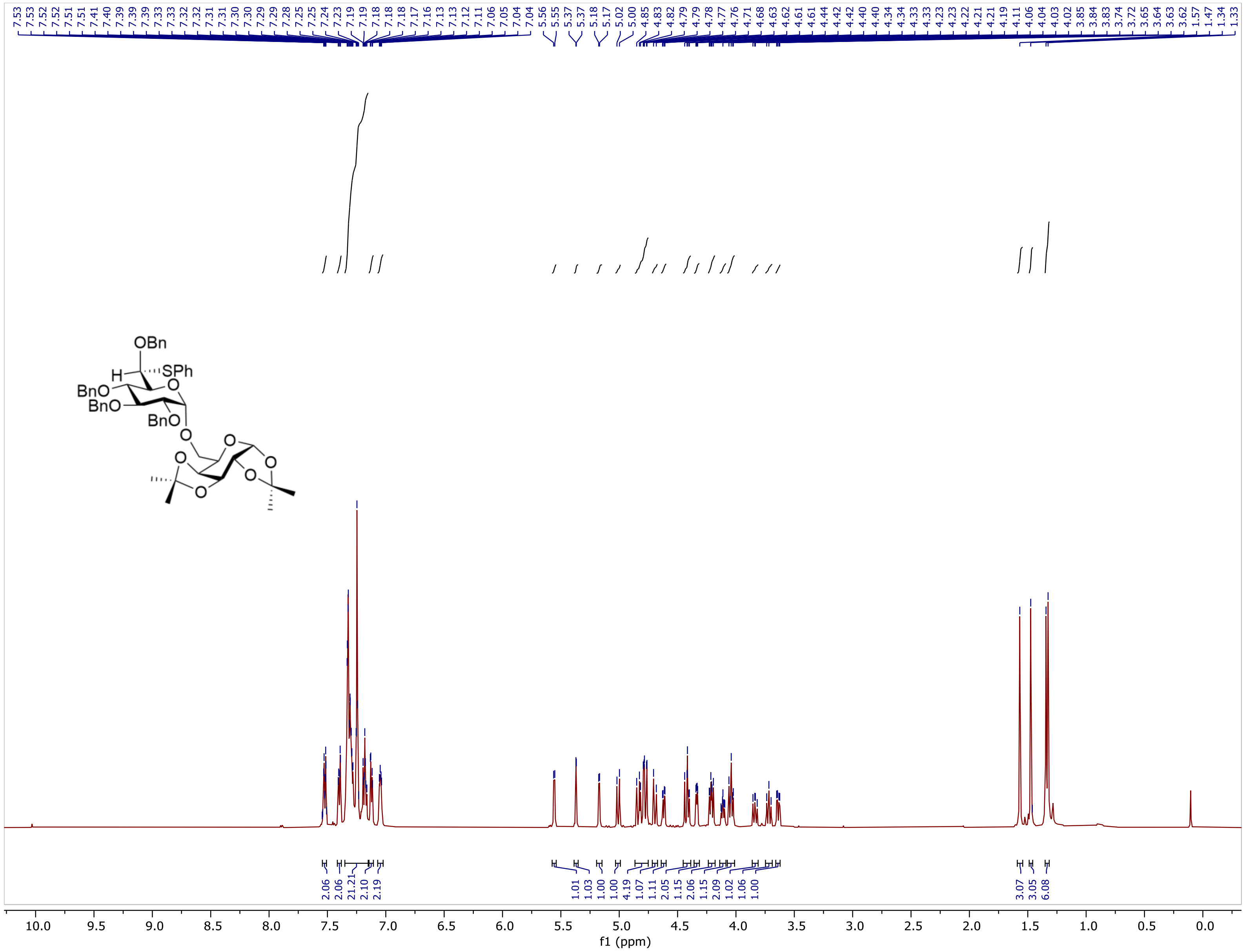

$^{13}C\{^{1}H\}$ NMR (126 MHz, $CDCl_3$) Spectrum of (6*S*)-6-phenylthio-2,3,4,6-tetra-*O*-benzyl-*α*-D-glucopyranosyl-(1→6)-1,2:3,4-*O*-diisopropylidene-α-D-galactopyranose (**21α**)

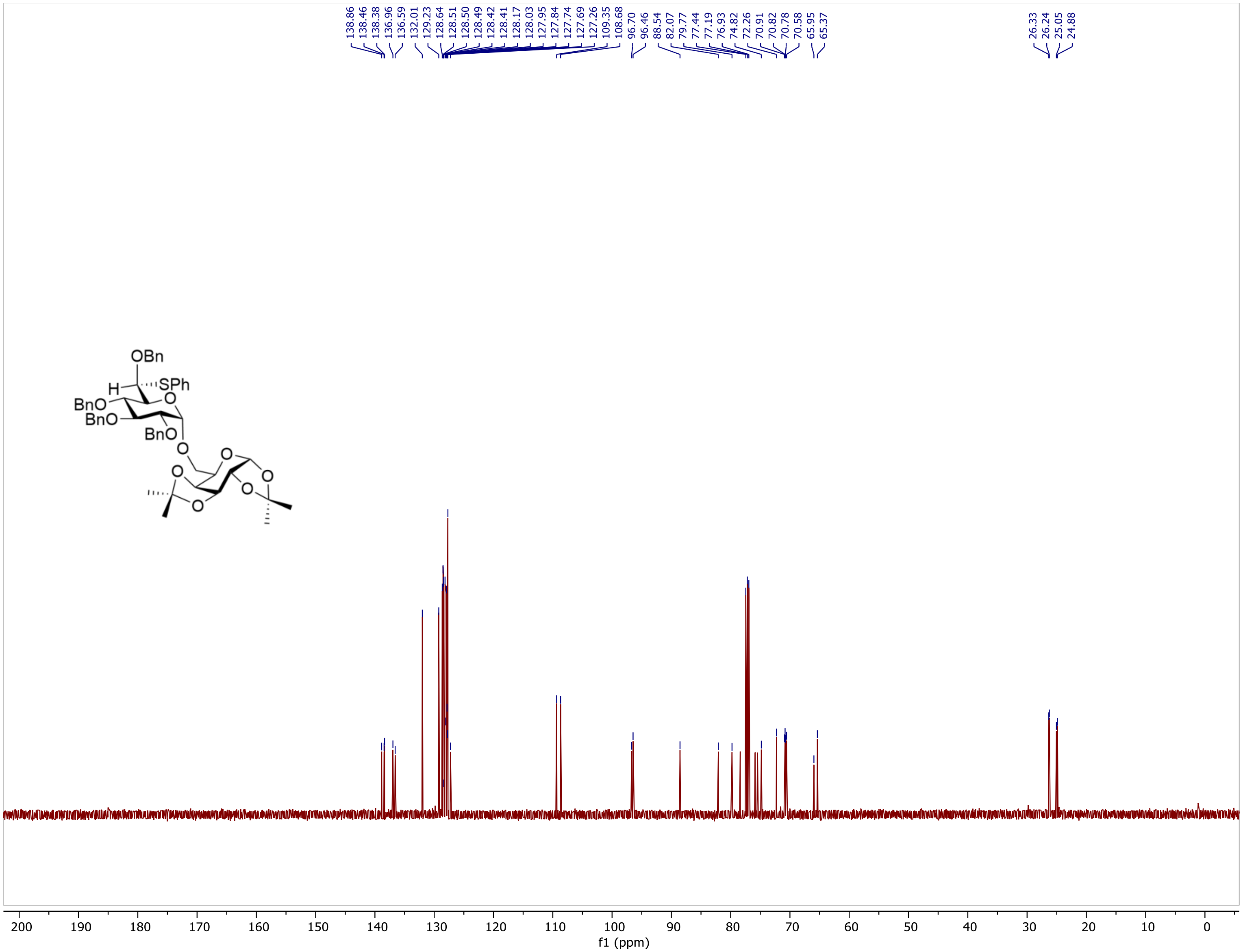

^{1}H NMR (500 MHz, $CDCl_3$) Spectrum of (6*S*)-6-phenylthio-2,3,4,6-tetra-*O*-benzyl-*β*-D-glucopyranosyl-(1→6)-1,2:3,4-*O*-diisopropylidene-α-D-galactopyranose (**21β**)

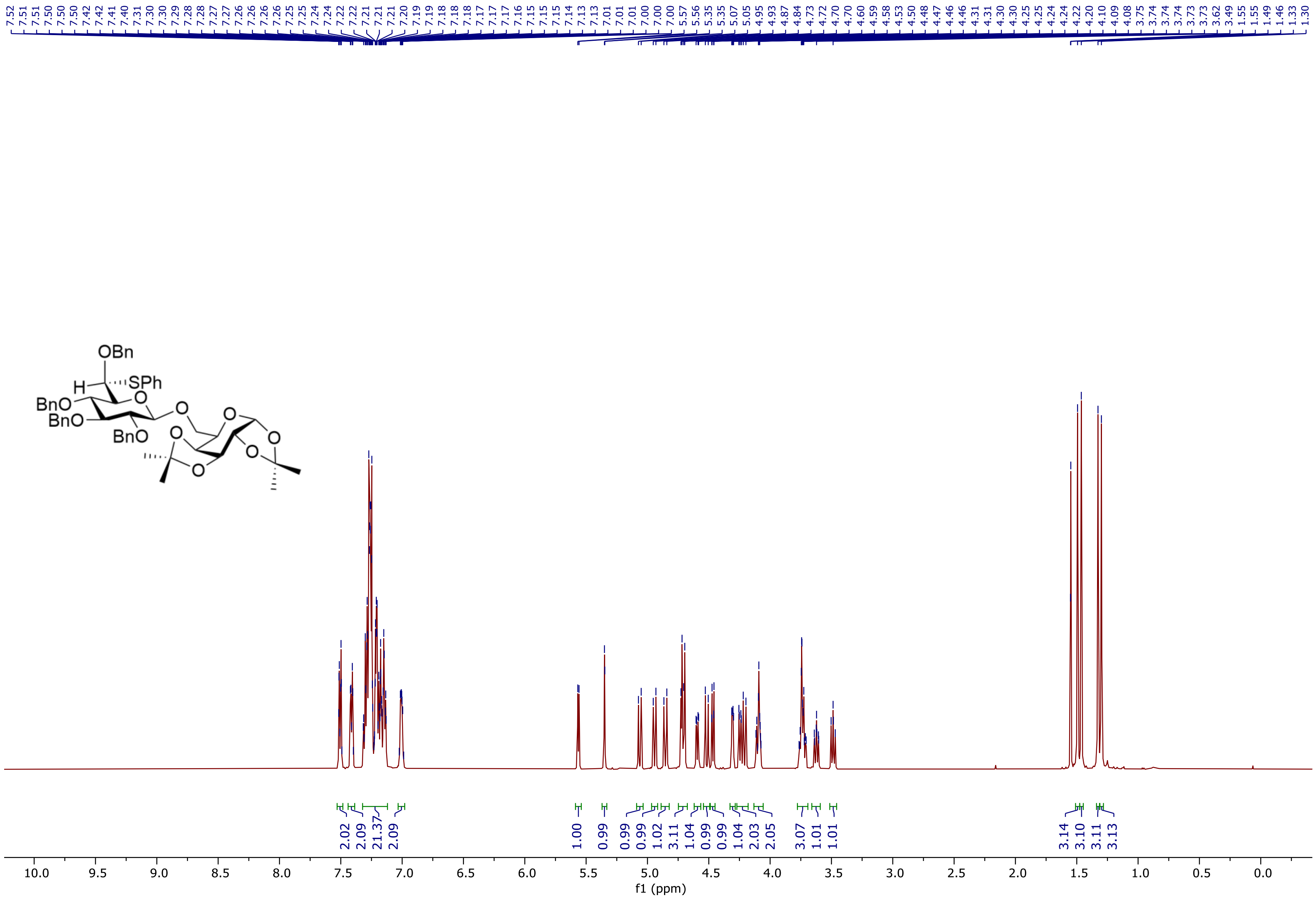

^{13}C{^{1}H} NMR (126 MHz, $CDCl_3$) Spectrum of (6*S*)-6-phenylthio-2,3,4,6-tetra-*O*-benzyl-*β*-D-glucopyranosyl-(1→6)-1,2:3,4-*O*-diisopropylidene-α-D-galactopyranose (**21β**)

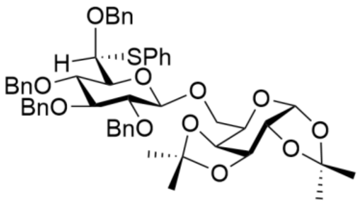

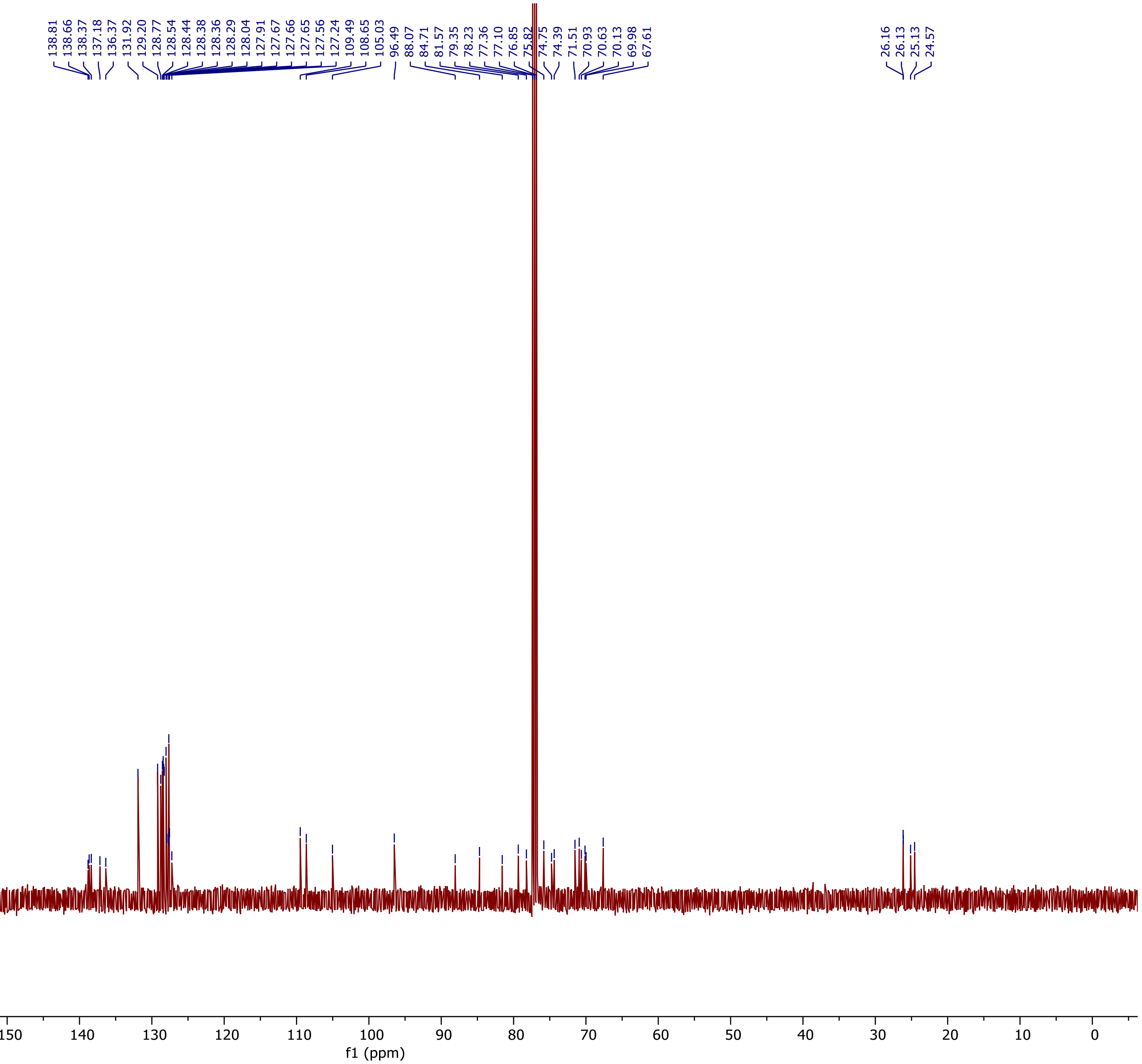

^{1}**H NMR** (600 MHz, $CDCl_3$) Spectrum of Adamantyl (6*S*)-6-phenylthio-2,3,4,6-tetra-*O*-benzyl-*α*-D-glucopyranoside **(22α)**

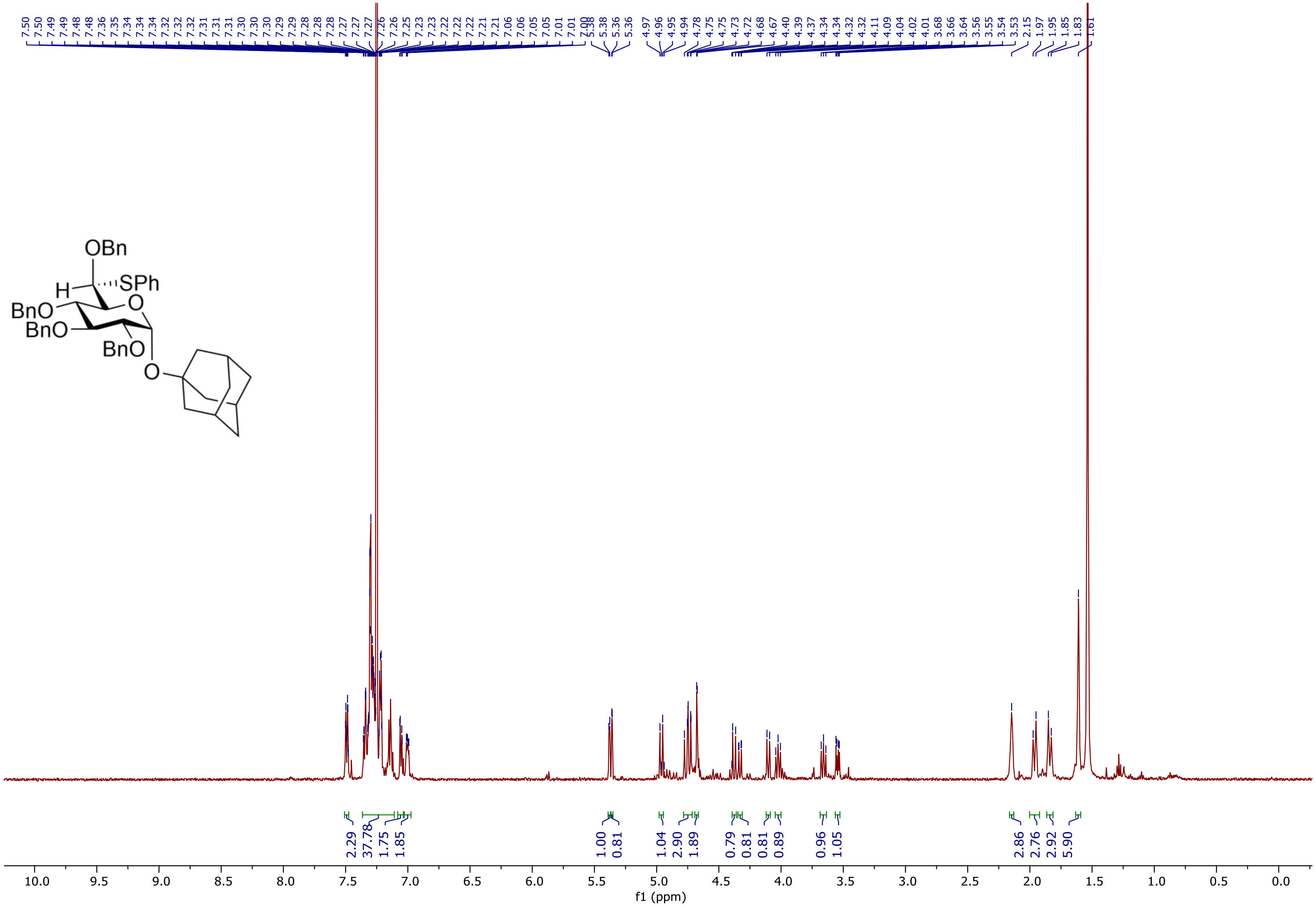

$^{13}C\{^{1}H\}$ **NMR** (156 MHz, $CDCl_3$) Spectrum of Adamantyl (6*S*)-6-phenylthio-2,3,4,6-tetra-*O*-benzyl-*α*-D-glucopyranoside **(22α)**

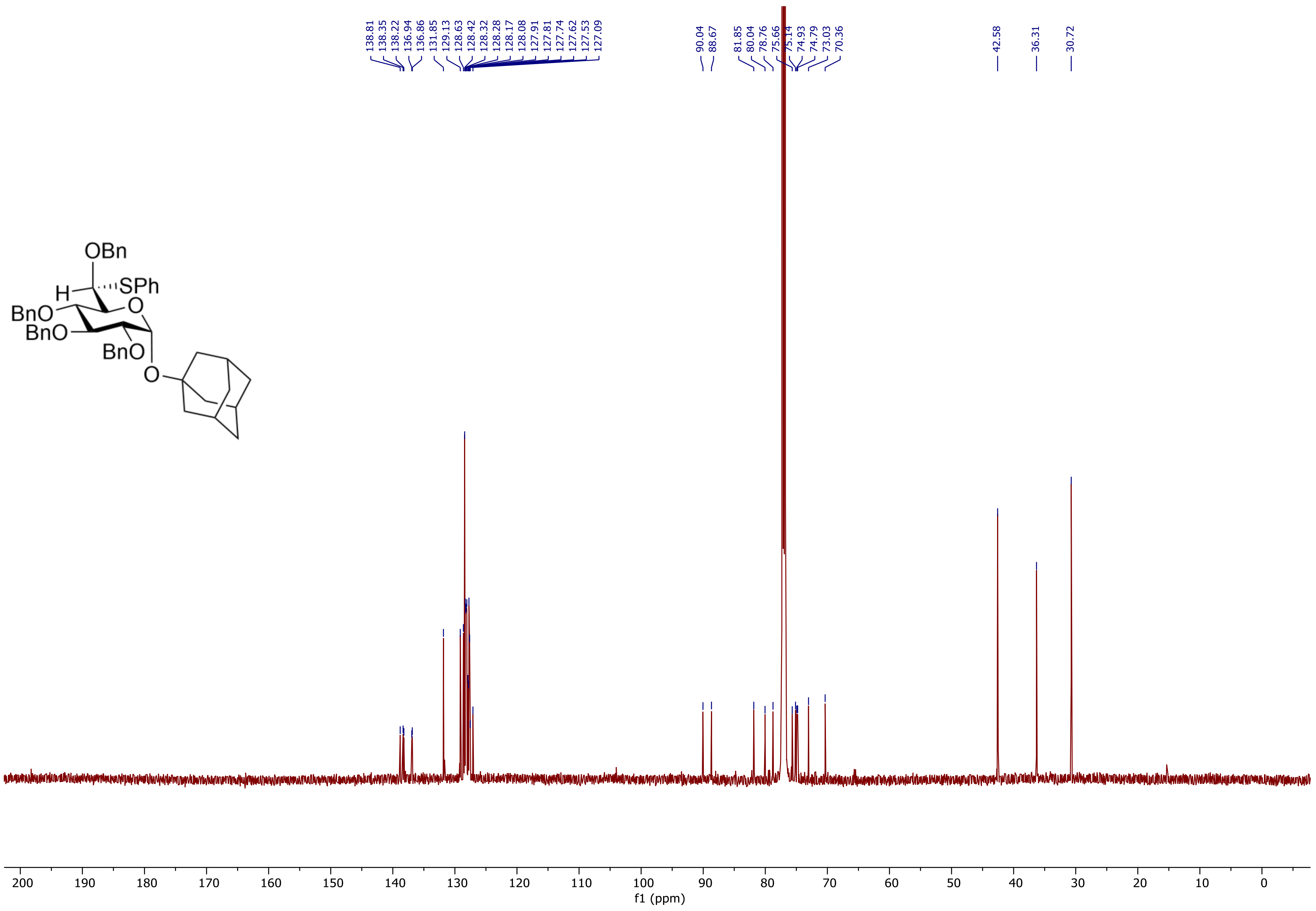

^{1}H NMR (500 MHz, $CDCl_3$) Spectrum of Adamantyl (6*S*)-6-phenylthio-2,3,4,6-tetra-*O*-benzyl-*β*-D-glucopyranoside **(22β)**

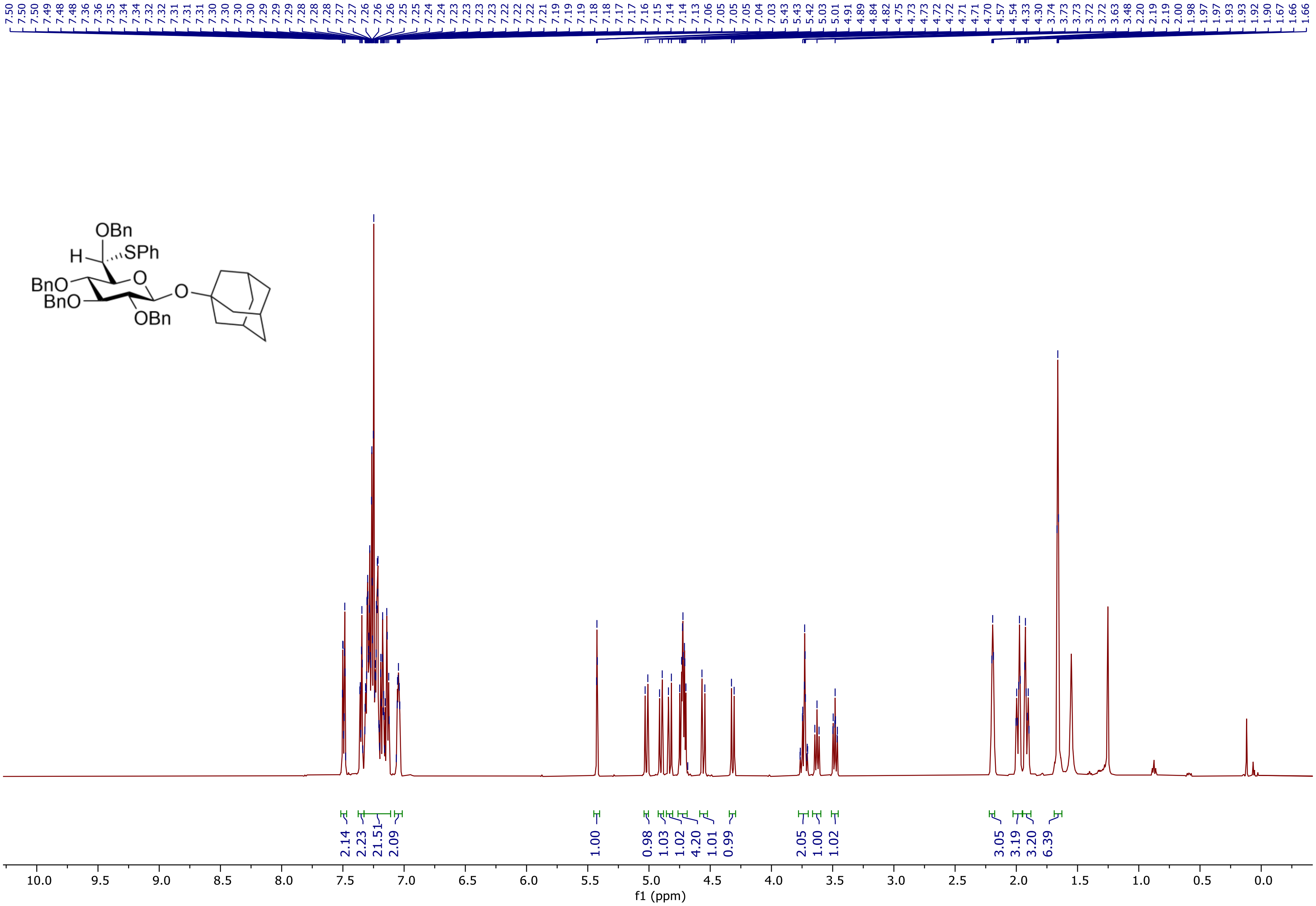

$^{13}C\{^{1}H\}$ NMR (126 MHz, $CDCl_3$) Spectrum of Adamantyl (6*S*)-6-phenylthio-2,3,4,6-tetra-*O*-benzyl-*β*-D-glucopyranoside **(22β)**

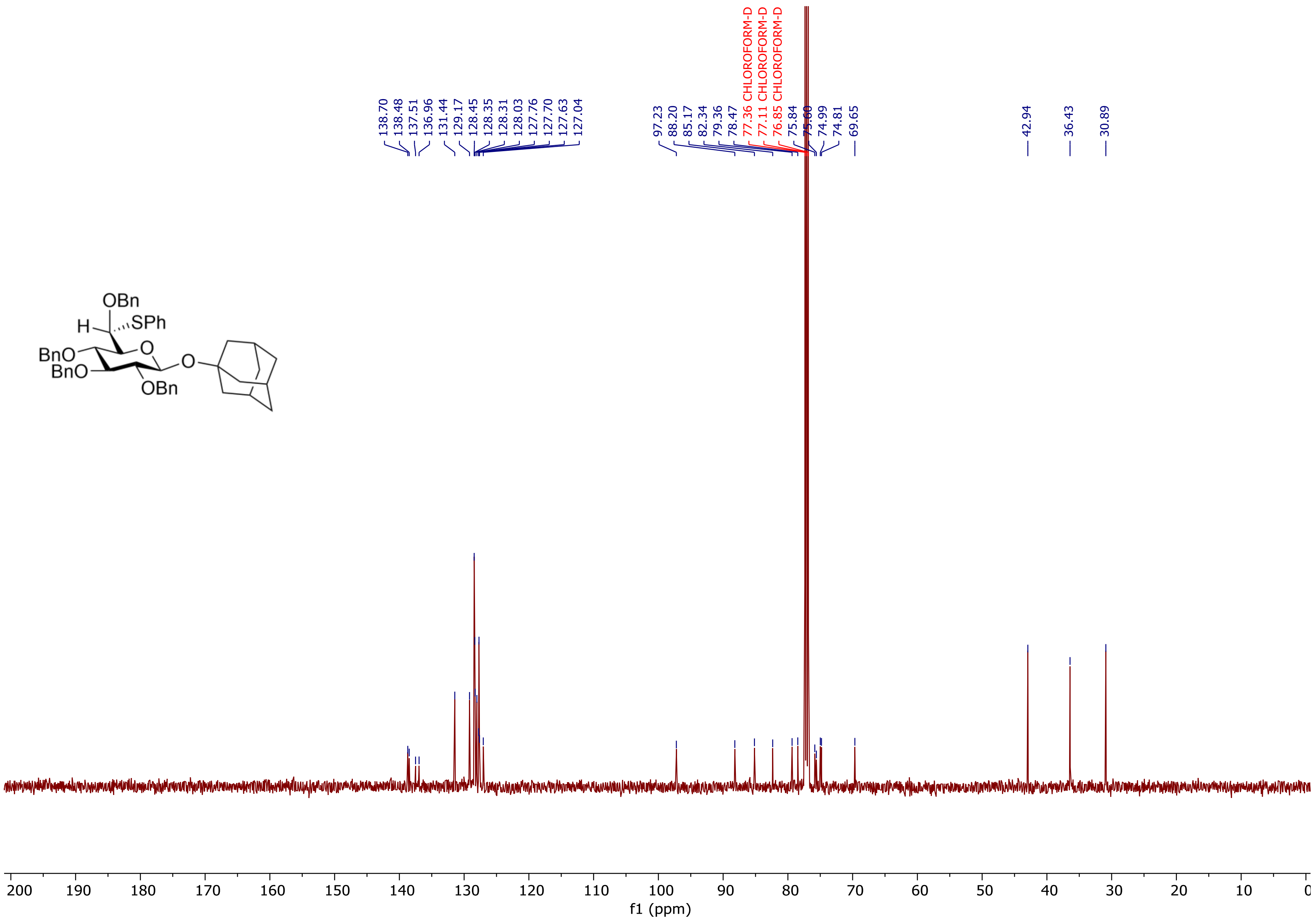

^{1}H NMR (500 MHz, $CDCl_3$) Spectrum of (6*S*)-6-phenylthio-2,3,4,6-tetra-*O*-benzyl-*α*-D-glucopyranosyl-(1→3)-1,2:5,6-di-*O*-isopropylidene-α-D-glucofuranose **(23α)**

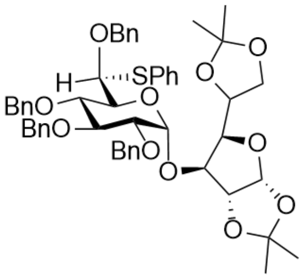

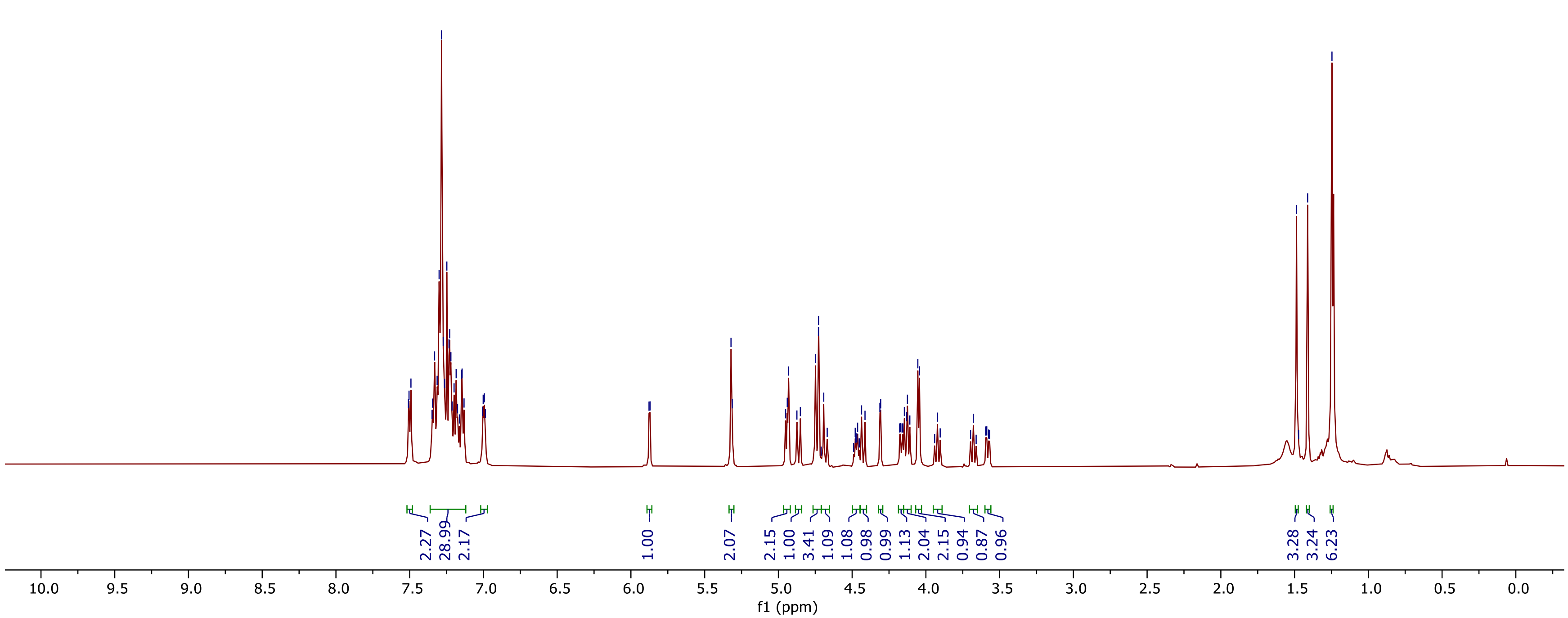

^{13}C{^{1}H} NMR (126 MHz, $CDCl_3$) Spectrum of (6*S*)-6-phenylthio-2,3,4,6-tetra-*O*-benzyl-*α*-D-glucopyranosyl-(1→3)-1,2:5,6-di-*O*-isopropylidene-α-D-glucofuranose **(23α)**

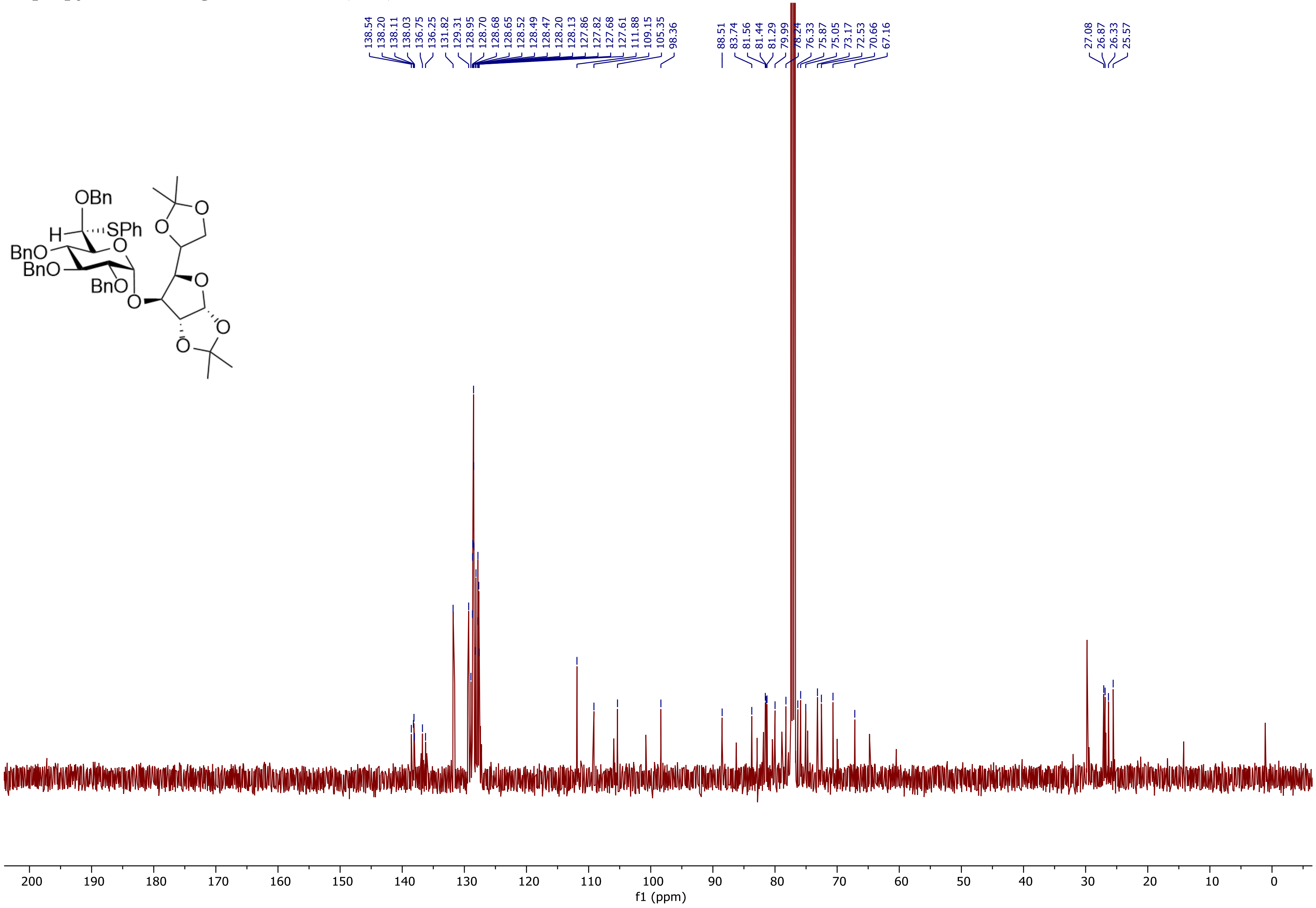

^{1}H NMR (500 MHz, $CDCl_3$) Spectrum of (6*S*)-6-phenylthio-2,3,4,6-tetra-*O*-benzyl-*β*-D-glucopyranosyl-(1→3)-1,2:5,6-di-*O*-isopropylidene-α-D-glucofuranose **(23β)**

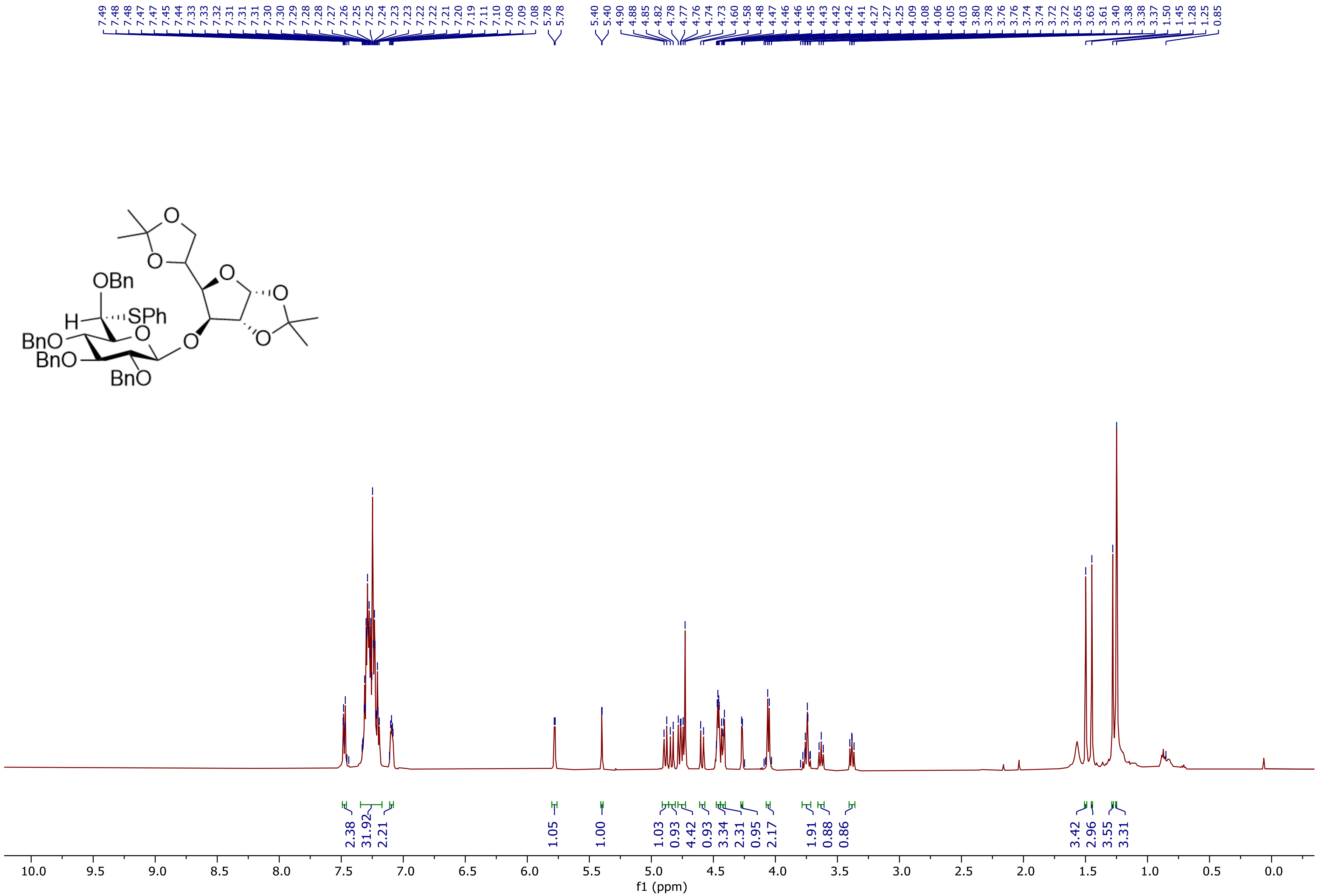

^{13}C{^{1}H} NMR (126 MHz, $CDCl_3$) Spectrum of (6*S*)-6-phenylthio-2,3,4,6-tetra-*O*-benzyl-*β*-D-glucopyranosyl-(1→3)-1,2:5,6-di-*O*-isopropylidene-α-D-glucofuranose **(23β)**

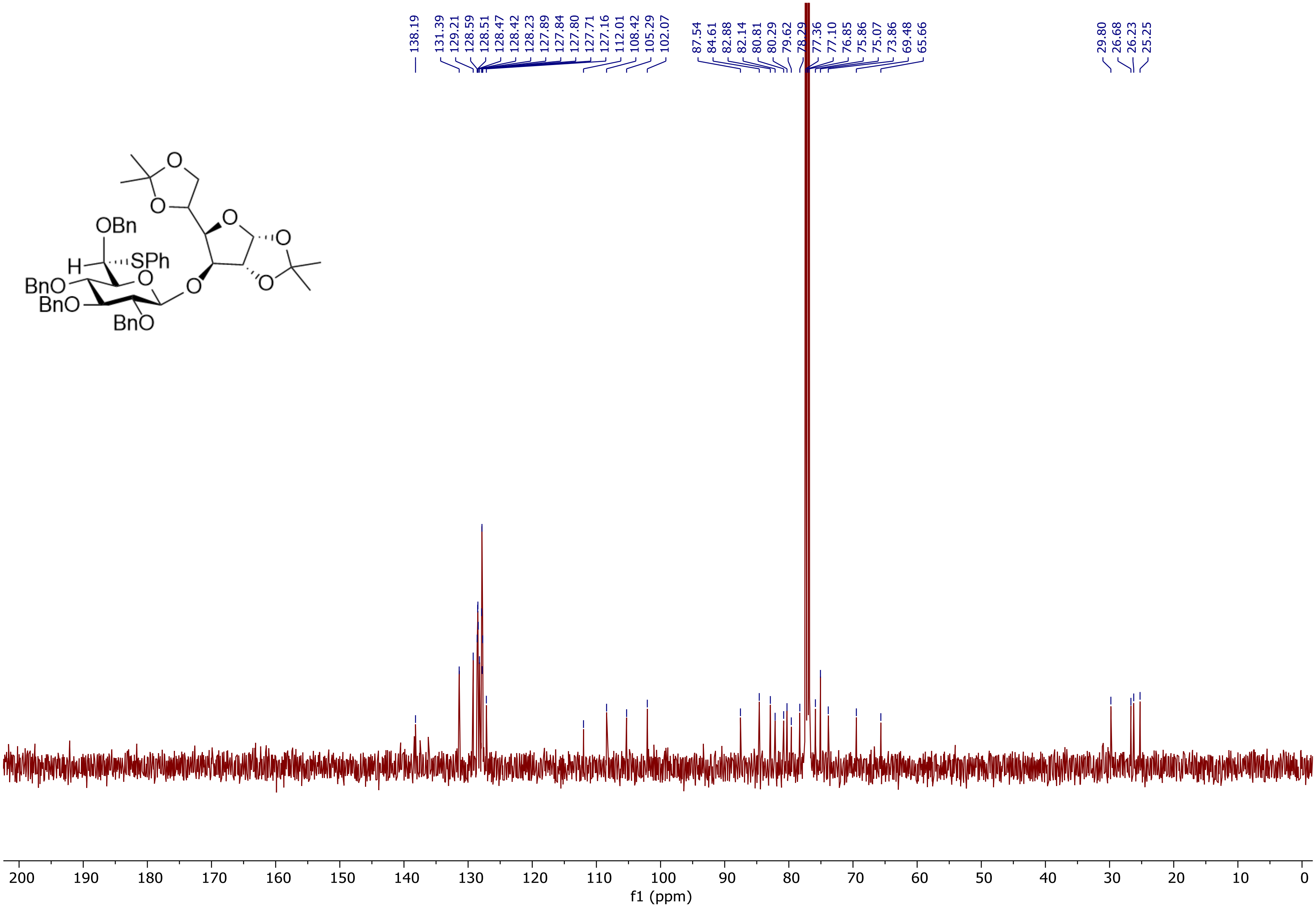

^{1}H NMR (500 MHz, CDCl$_3$) Spectrum of Methyl (6*S*)-6-phenylthio-2,3,4,6-tetra-*O*-benzyl-α-D-glucopyranosyl-(1→4)-2,3-O-isopropylidene-α-L-rhamnopyranoside. **(24α)**

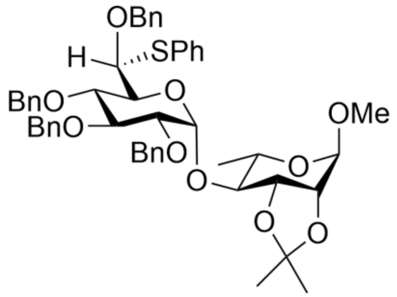

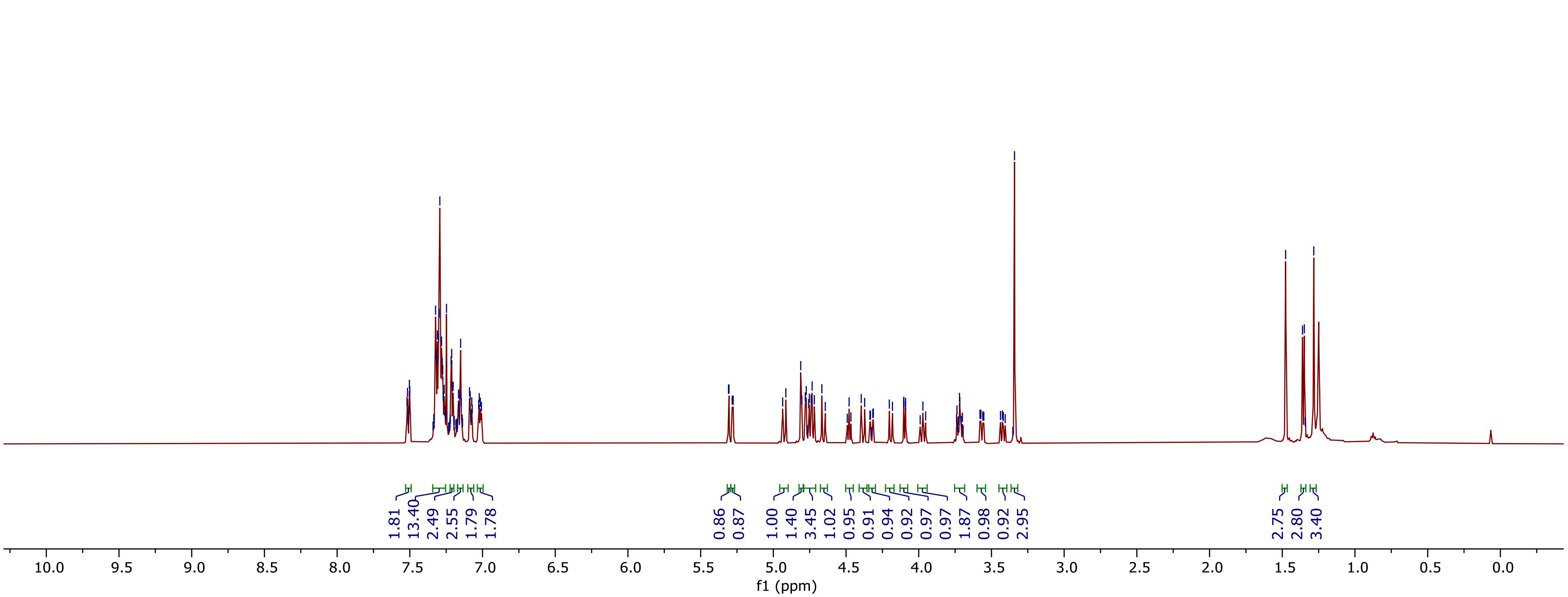

^{13}C{^{1}H} NMR (126 MHz, $CDCl_3$) Spectrum of Methyl (6*S*)-6-phenylthio-2,3,4,6-tetra-*O*-benzyl-*α*-D-glucopyranosyl-(1→4)-2,3-O-isopropylidene-α-L-rhamnopyranoside. **(24α)**

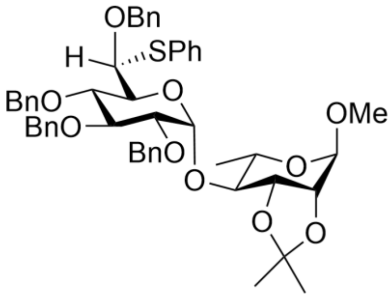

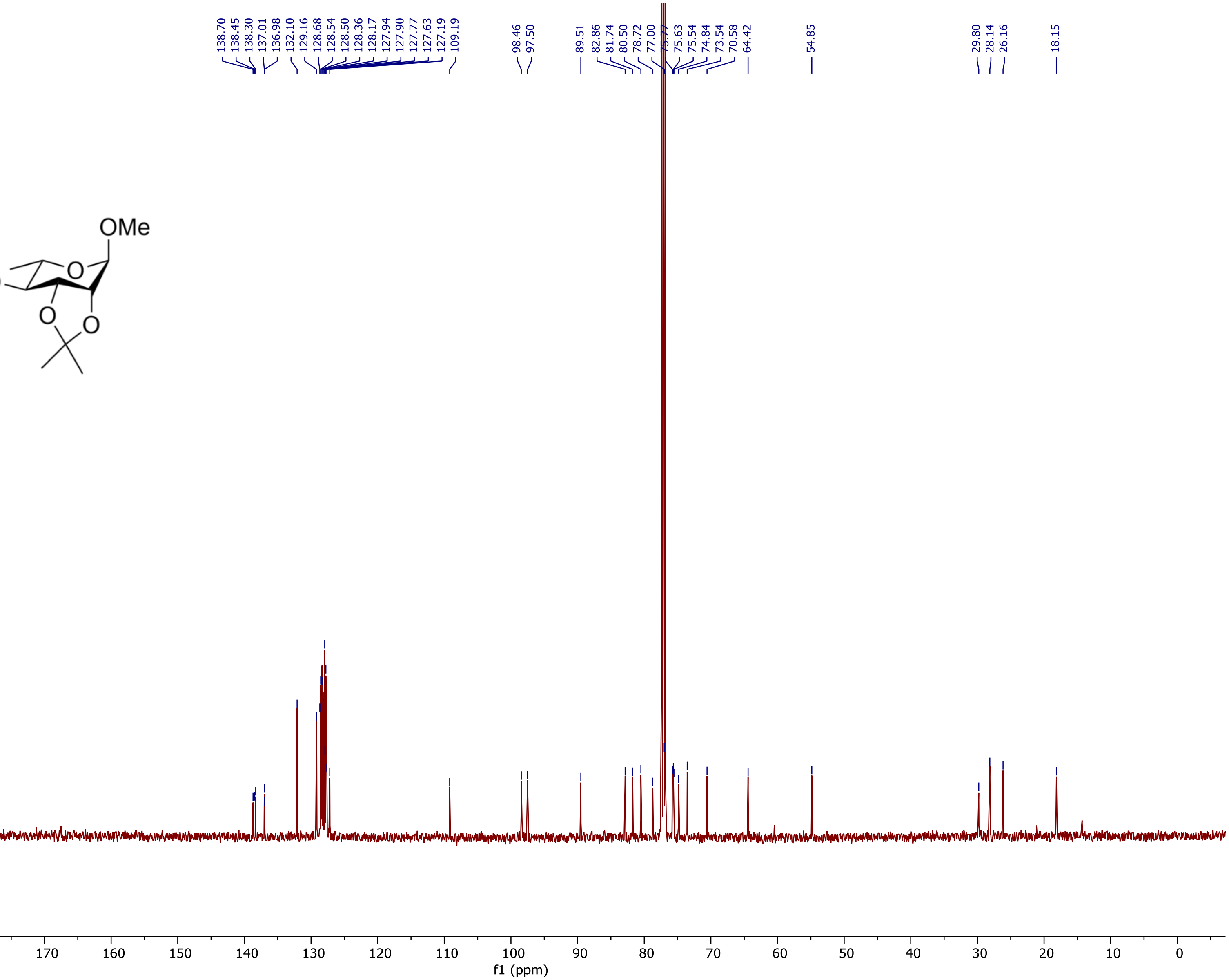

^{1}H NMR (500 MHz, CDCl$_3$) Spectrum of Methyl (6*S*)-6-phenylthio-2,3,4,6-tetra-*O*-benzyl-*β*-D-glucopyranosyl-(1→4)-2,3-O-isopropylidene-α-L-rhamnopyranoside. **(24β)**

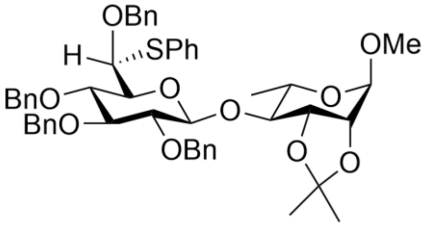

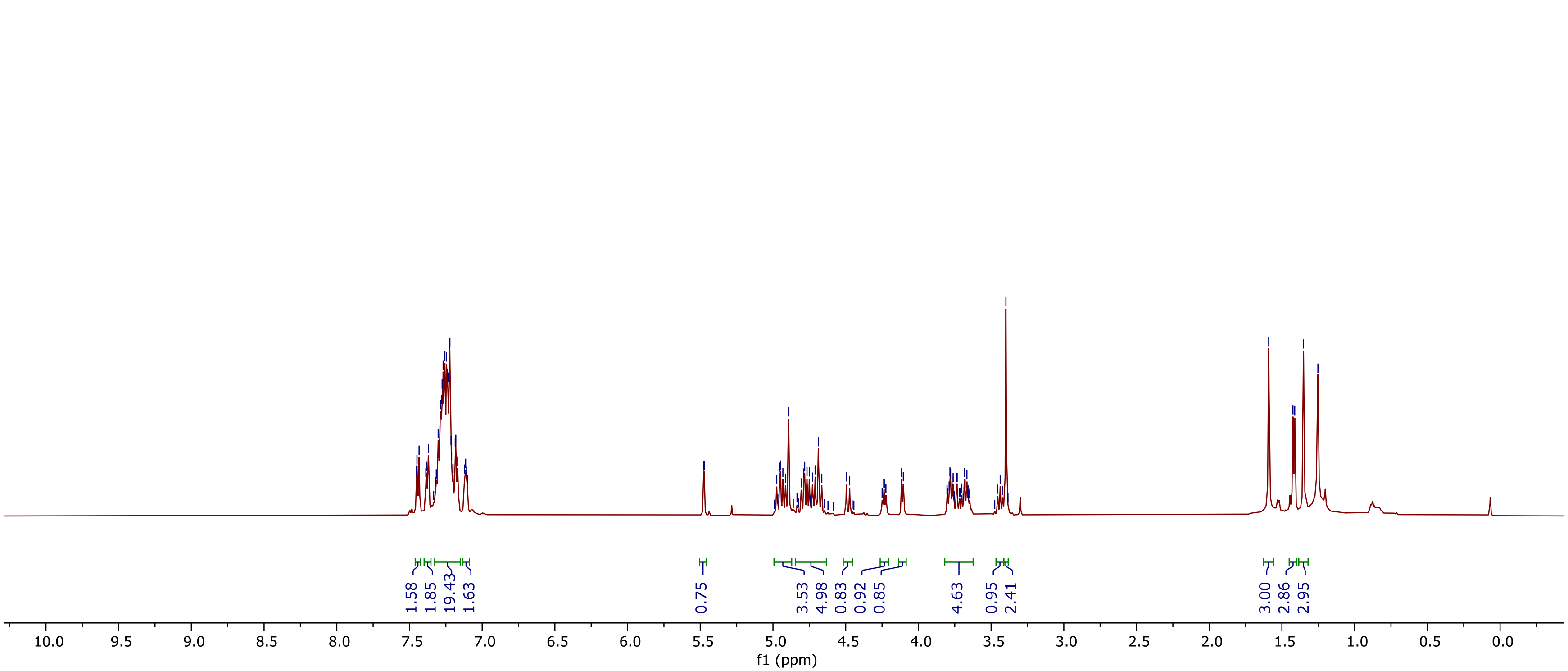

^{13}C{^{1}H} NMR (126 MHz, $CDCl_3$) Spectrum of Methyl (6*S*)-6-phenylthio-2,3,4,6-tetra-*O*-benzyl-*β*-D-glucopyranosyl-(1→4)-2,3-O-isopropylidene-α-L-rhamnopyranoside. **(24β)**

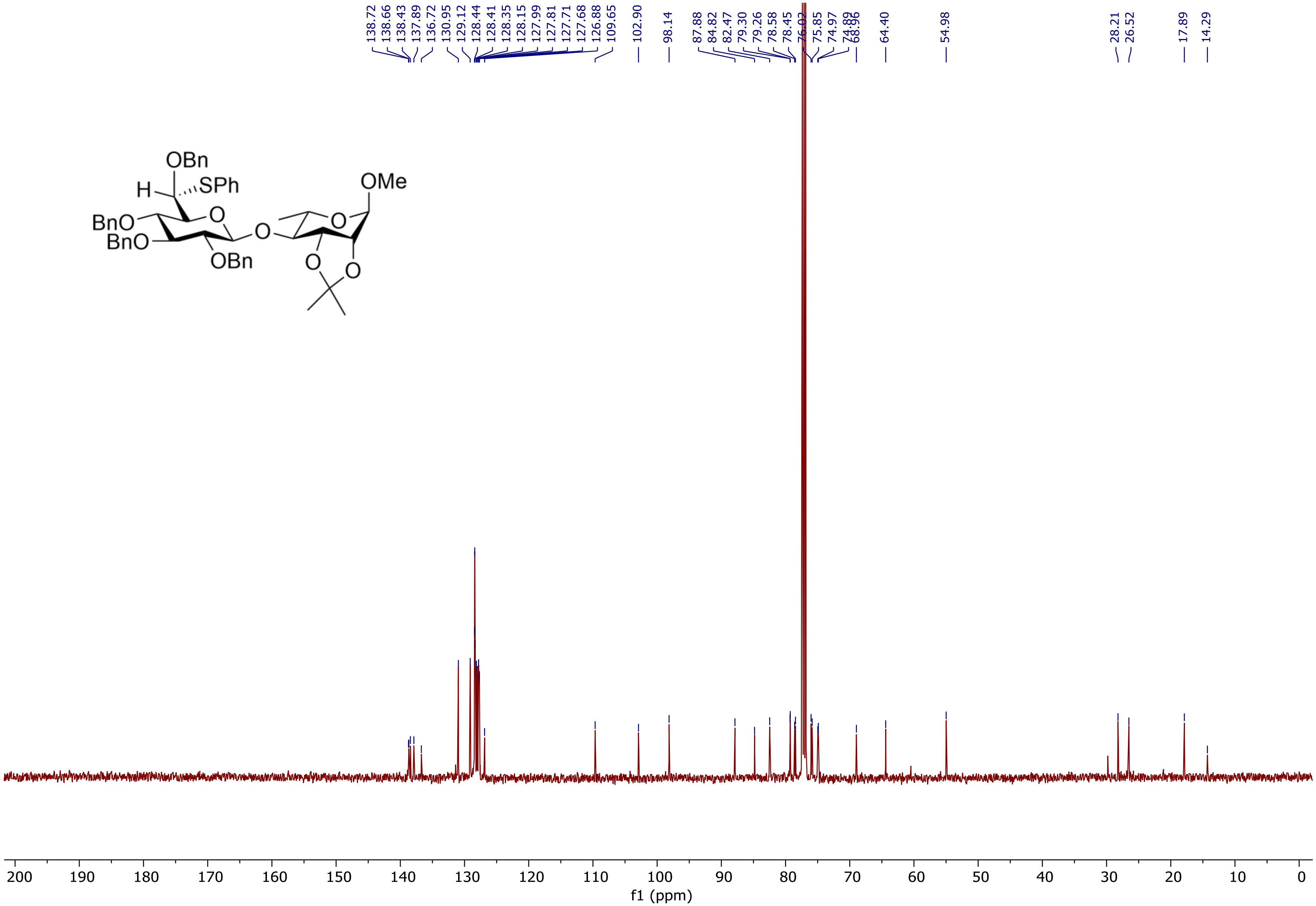

^{1}H NMR (500 MHz, $CDCl_3$) Spectrum of Methyl (6*S*)-6-phenylthio-2,3,4,6-tetra-*O*-benzyl-*α*-D-glucopyranosyl-(1→4)-2,3,6-tri-*O*-benzyl-α-D-glucopyranoside **(25α)**

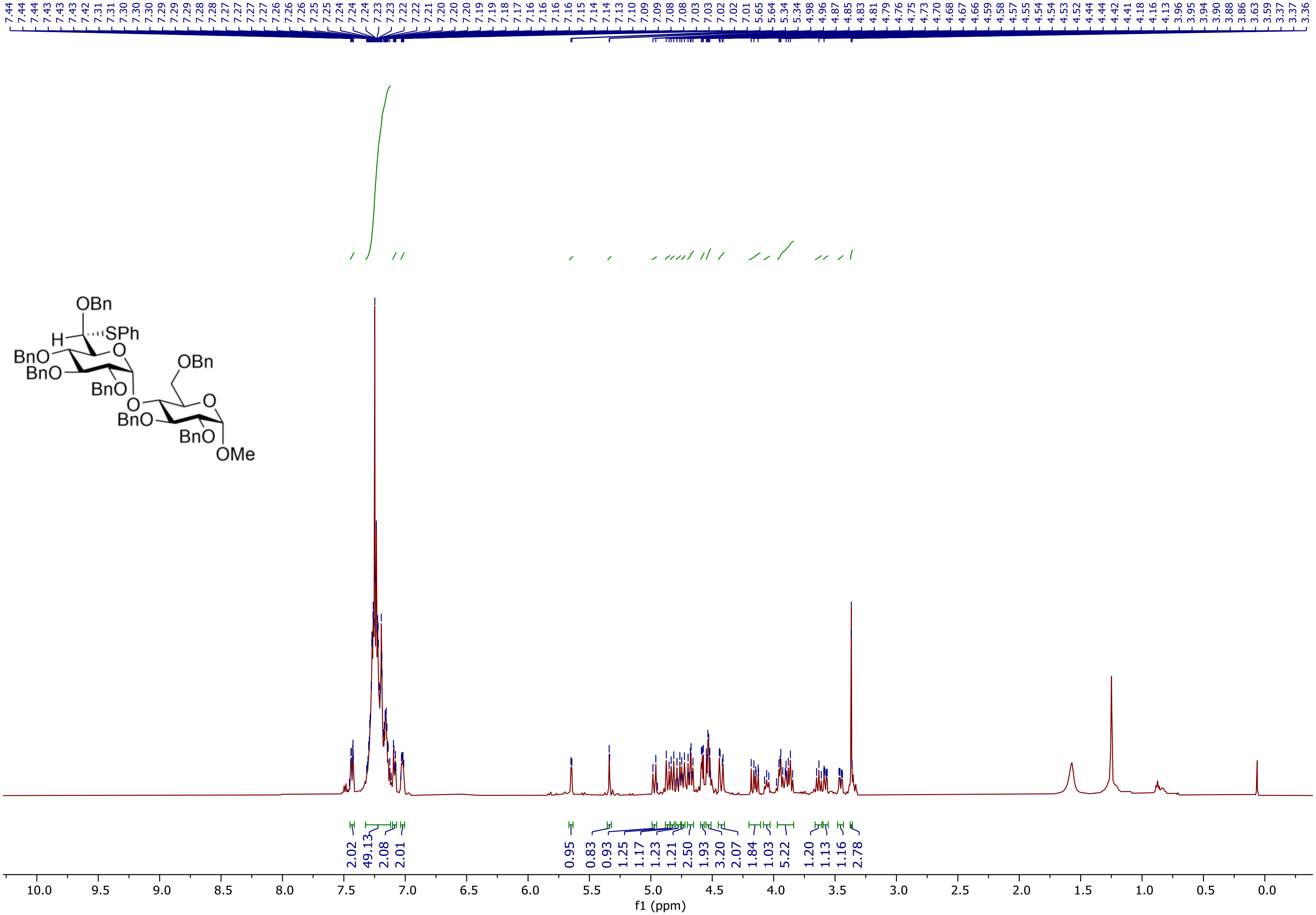

^{13}C{^{1}H} NMR (126 MHz, $CDCl_3$) Spectrum of Methyl (6*S*)-6-phenylthio-2,3,4,6-tetra-*O*-benzyl-*α*-D-glucopyranosyl-(1→4)-2,3,6-tri-*O*-benzyl-α-D-glucopyranoside **(25α)**

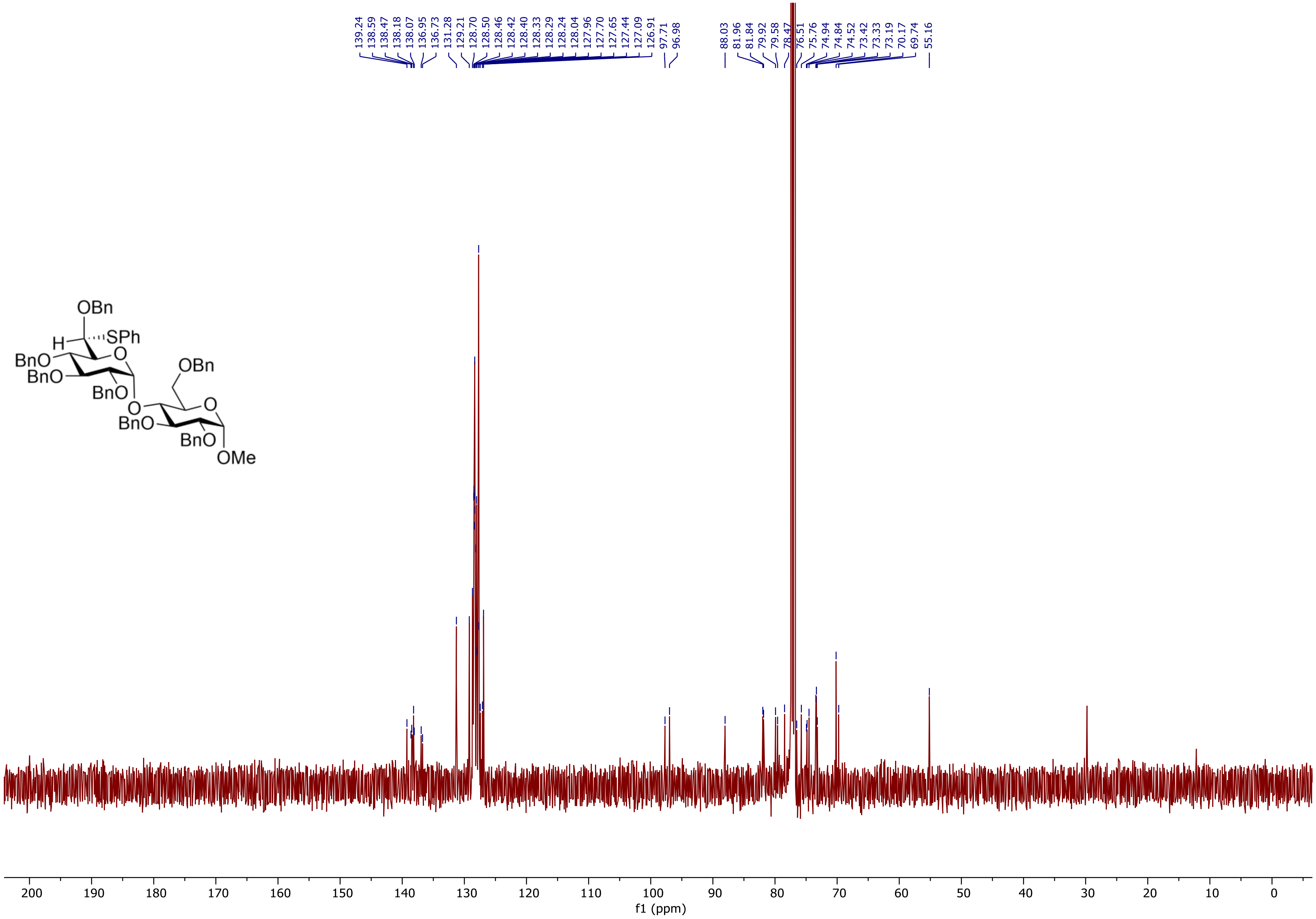

^{1}H NMR (500 MHz, $CDCl_3$) Spectrum of **25α** and **25β** enriched in **25β** Methyl (6*S*)-6-phenylthio-2,3,4,6-tetra-*O*-benzyl-*β*-D-glucopyranosyl-(1→4)-2,3,6-tri-*O*-benzyl-α-D-glucopyranoside.

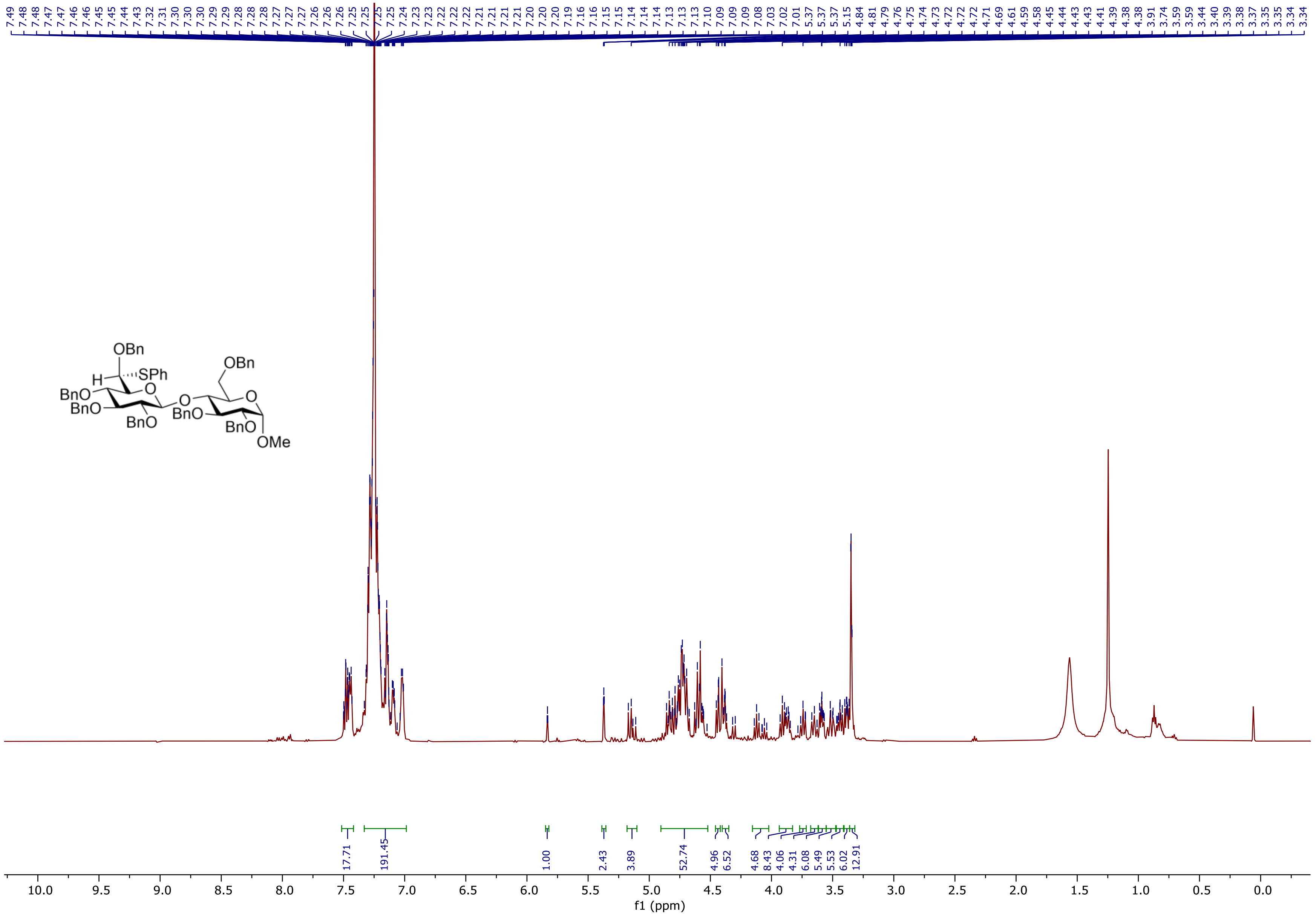

¹H NMR (500 MHz, $CDCl_3$) Spectrum of (6*R*)-6-phenylthio-2,3,4,6-tetra-*O*-benzyl-*α*-D-glucopyranosyl-(1→6)-1,2:3,4-*O*-diisopropylidene-α-D-galactopyranose **(26α)**

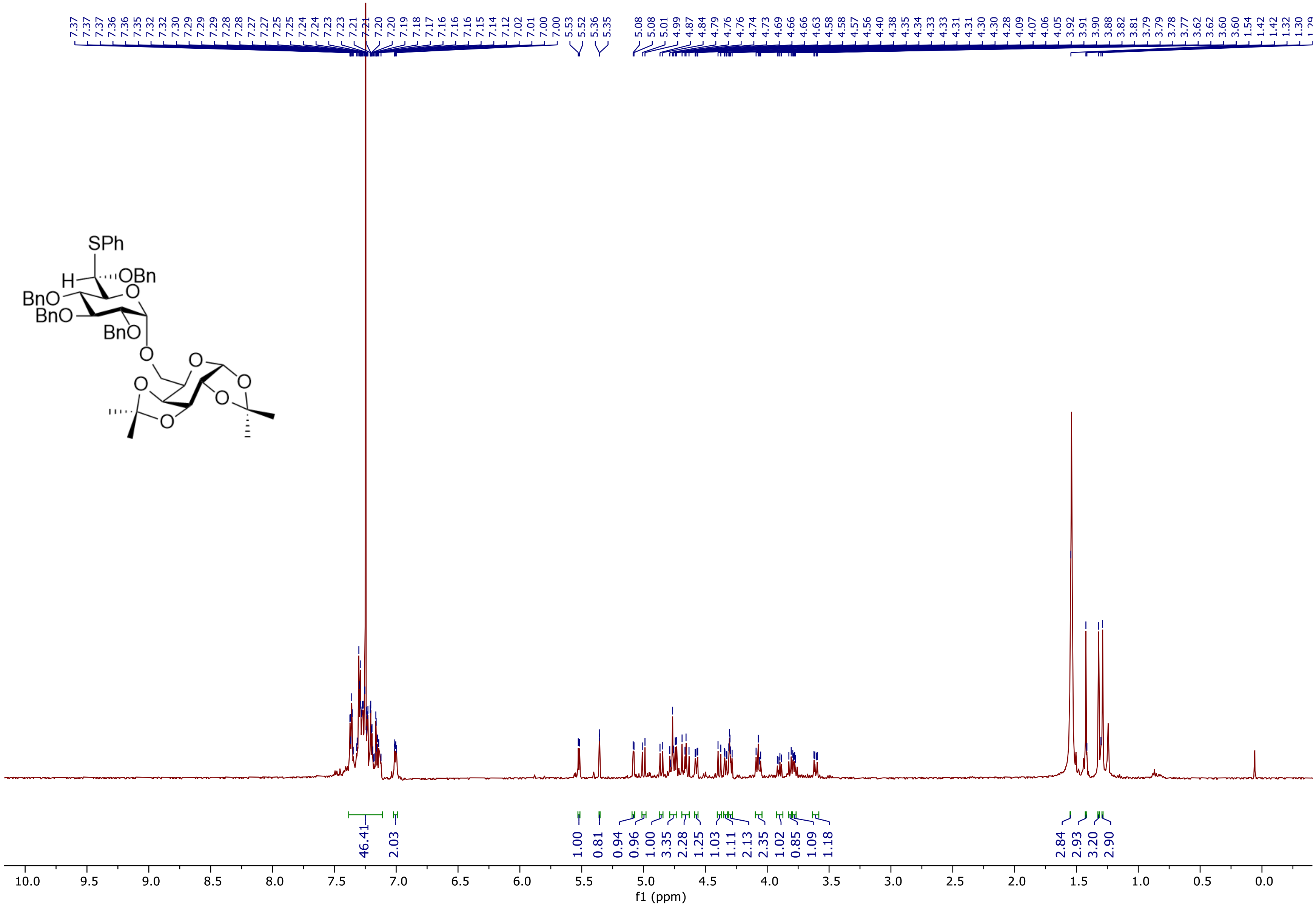

$^{13}C\{^1H\}$ NMR (126 MHz, $CDCl_3$) Spectrum of (6*R*)-6-phenylthio-2,3,4,6-tetra-*O*-benzyl-*α*-D-glucopyranosyl-(1→6)-1,2:3,4-*O*-diisopropylidene-α-D-galactopyranose **(26α)**

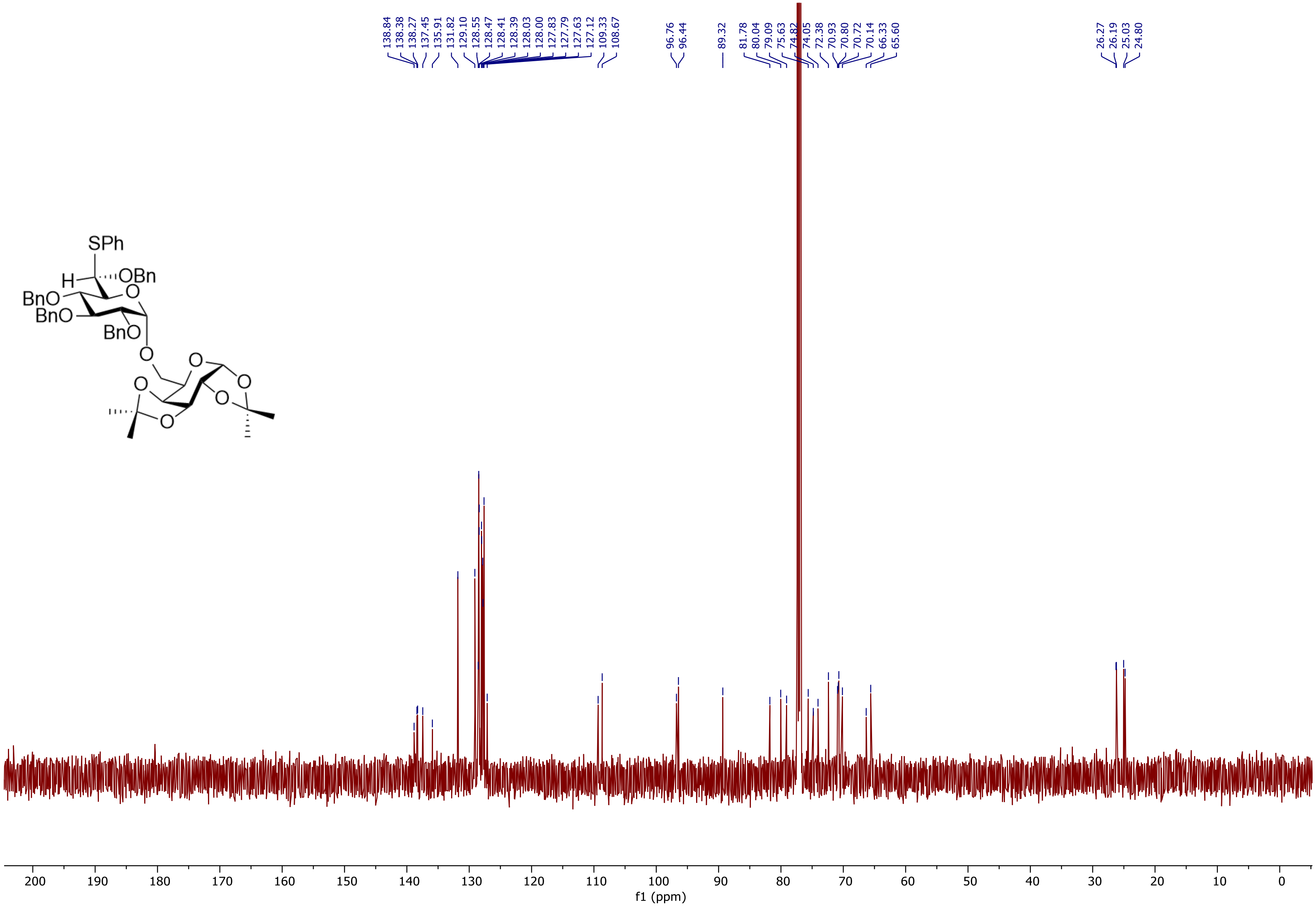

^{1}H NMR (500 MHz, $CDCl_3$) Spectrum of (6*R*)-6-phenylthio-2,3,4,6-tetra-*O*-benzyl-*β*-D-glucopyranosyl-(1→6)-1,2:3,4-*O*-diisopropylidene-α-D-galactopyranose **(26β)**

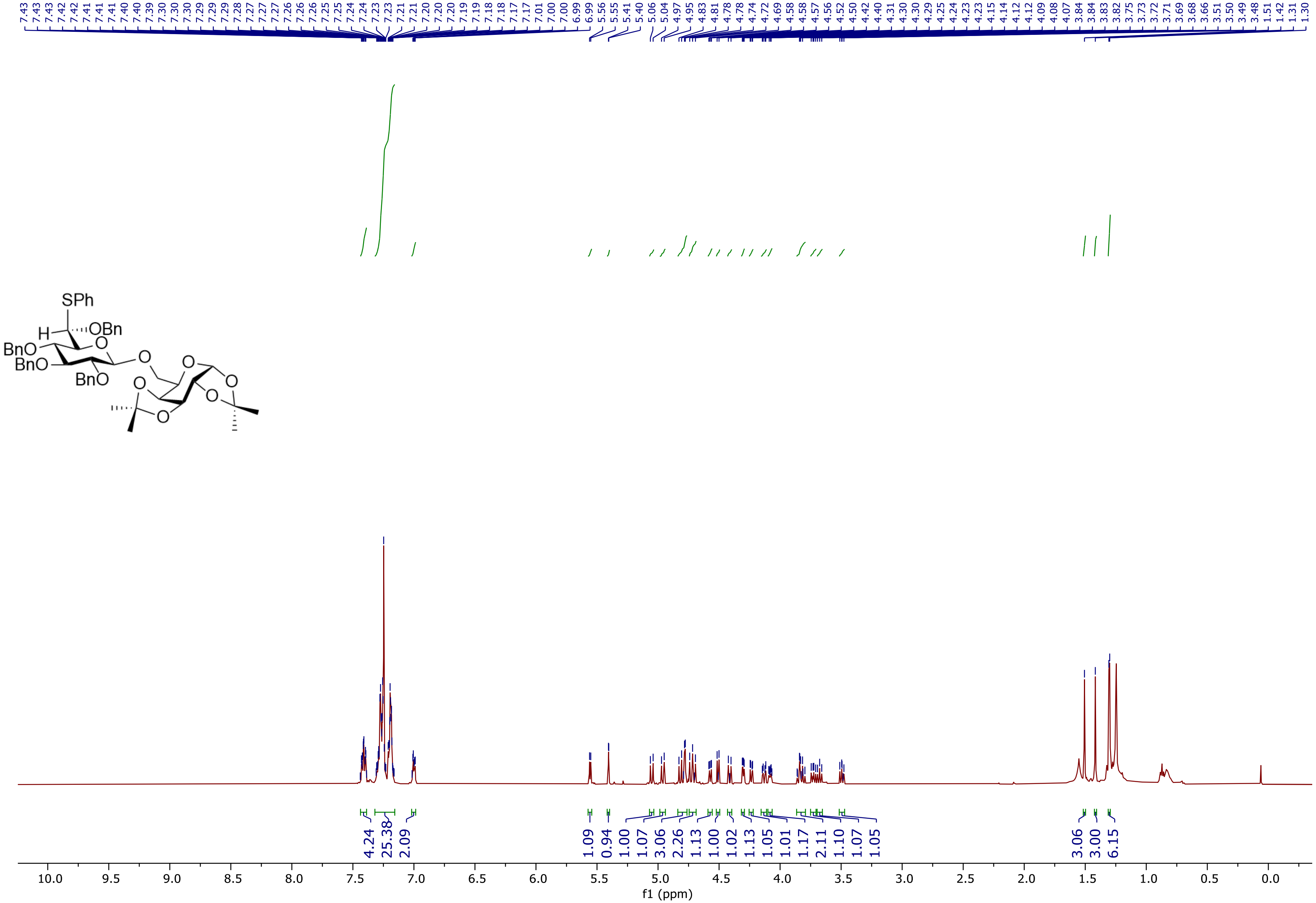

^{13}C{^{1}H} NMR (126 MHz, $CDCl_3$) Spectrum of (6*R*)-6-phenylthio-2,3,4,6-tetra-*O*-benzyl-*β*-D-glucopyranosyl-(1→6)-1,2:3,4-*O*-diisopropylidene-α-D-galactopyranose **(26β)**

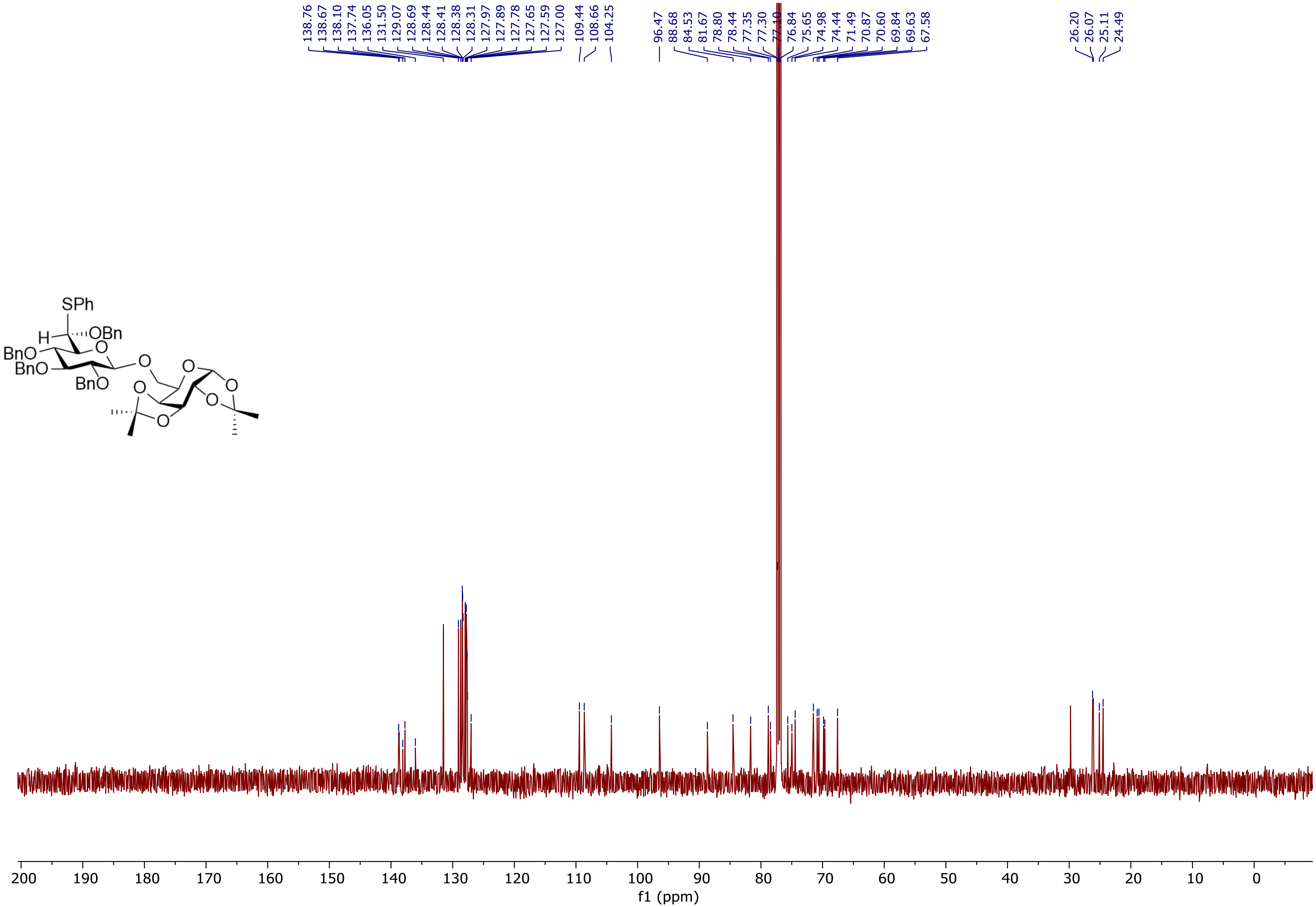

^{1}H NMR (500 MHz, CDCl$_3$) Spectrum of Adamantyl (6*R*)-6-phenylthio-2,3,4,6-tetra-*O*-benzyl-*α*-D-glucopyranoside. **(27α)**

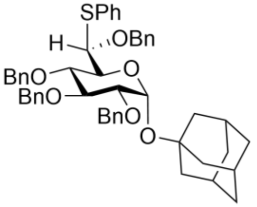

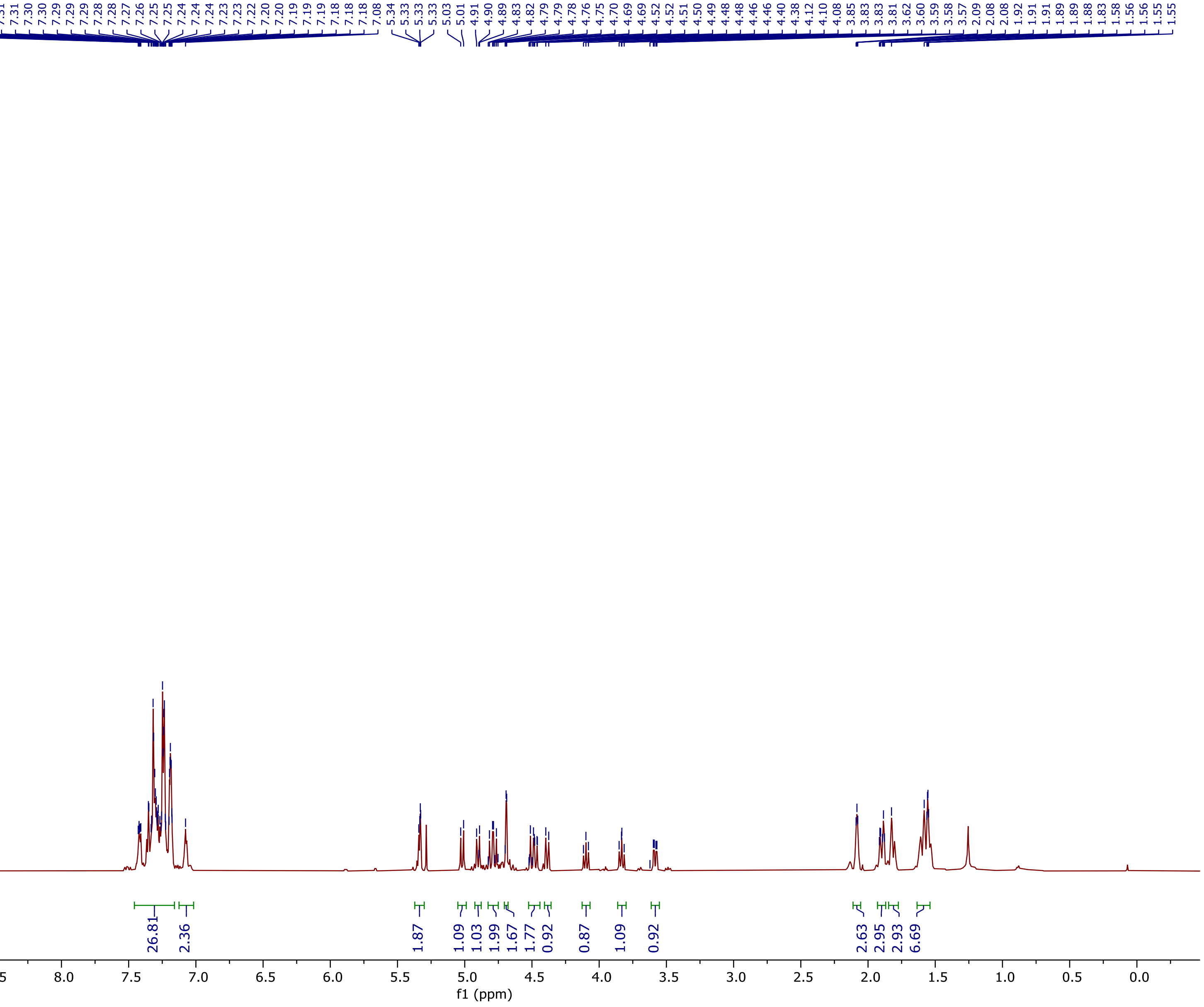

$^{13}C\{^{1}H\}$ **NMR** (126 MHz, $CDCl_3$) Spectrum of Adamantyl (6*R*)-6-phenylthio-2,3,4,6-tetra-*O*-benzyl-*α*-D-glucopyranoside. **(27α)**

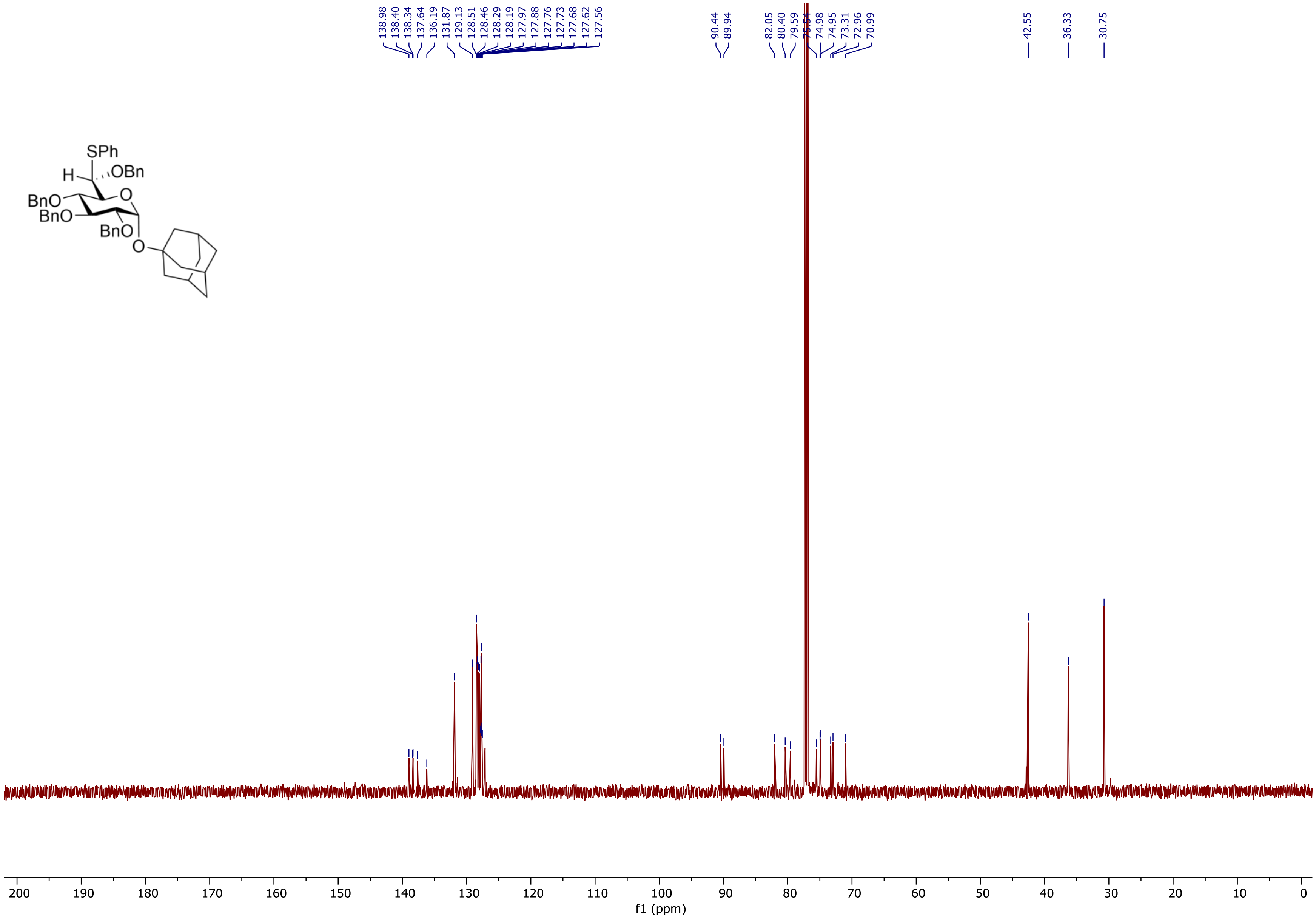

^{1}H NMR (500 MHz, CDCl$_3$) Spectrum of **27α** and **27β** enriched in **27β** Adamantyl (6*R*)-6-phenylthio-2,3,4,6-tetra-*O*-benzyl-*β*-D-glucopyranoside. from crude sample

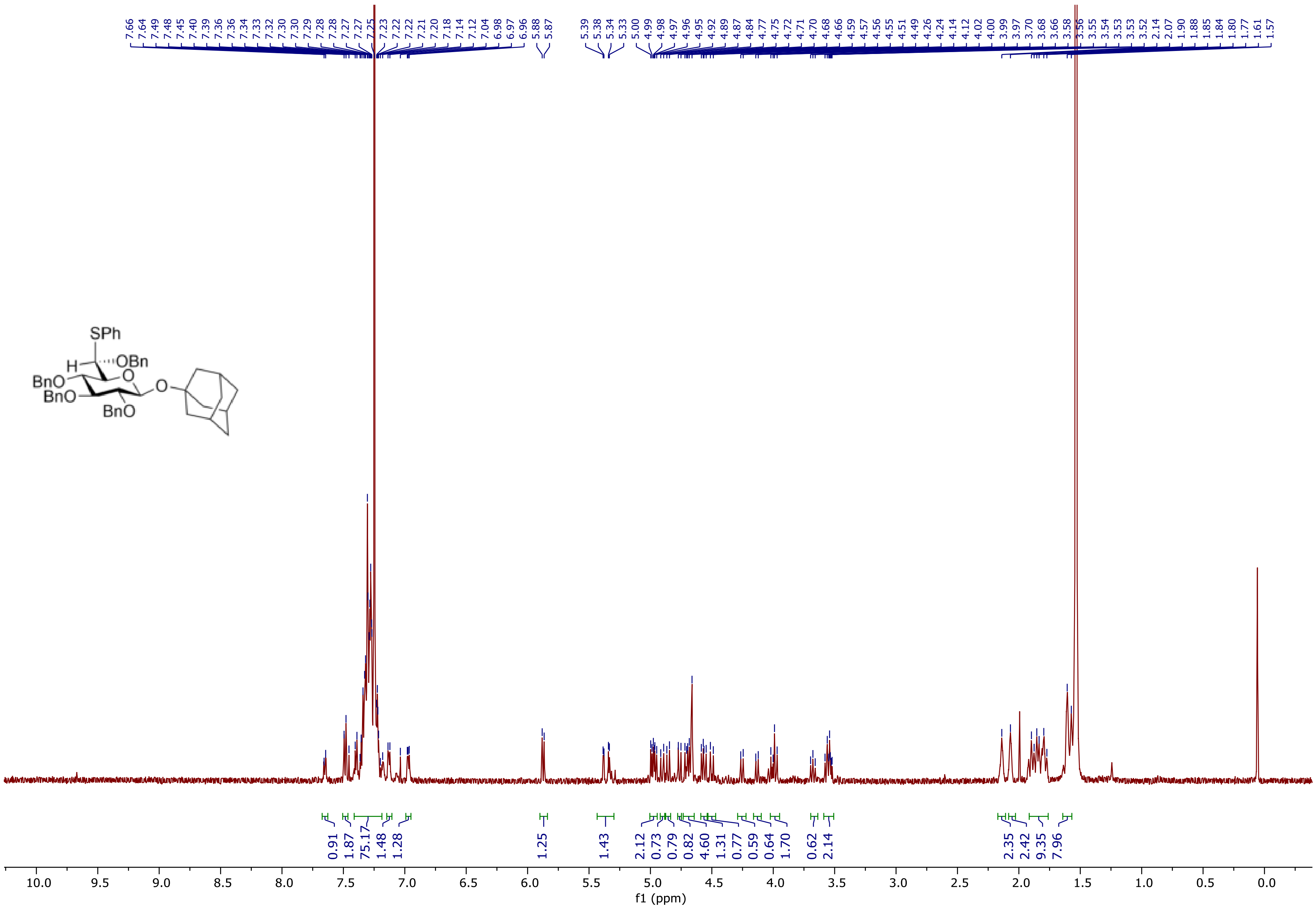

^{1}H NMR (500 MHz, CDCl$_3$) Spectrum of Methyl (6*R*)-6-phenylthio-2,3,4,6-tetra-*O*-benzyl-*α*-D-glucopyranosyl-(1→4)-2,3-*O*-isopropylidene-α-L-rhamnopyranoside. **(28α)**

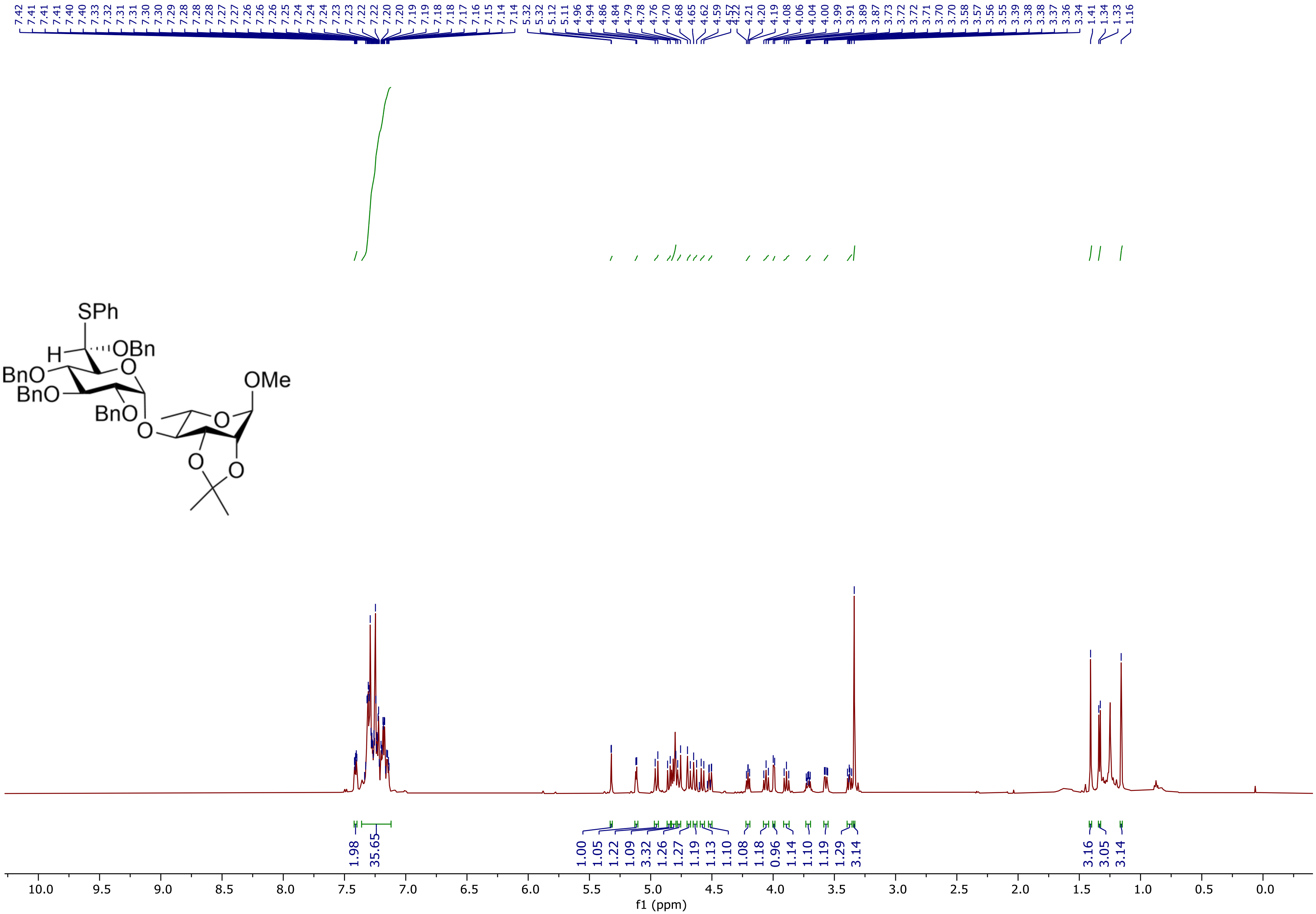

^{13}C{^{1}H} NMR (126 MHz, $CDCl_3$) Spectrum of Methyl (6*R*)-6-phenylthio-2,3,4,6-tetra-*O*-benzyl-*α*-D-glucopyranosyl-(1→4)-2,3-*O*-isopropylidene-α-L-rhamnopyranoside. **(28α)**

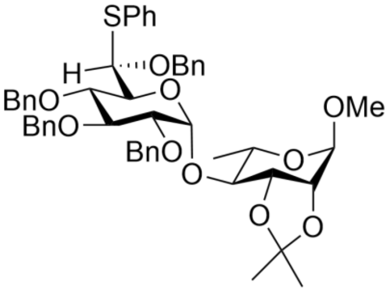

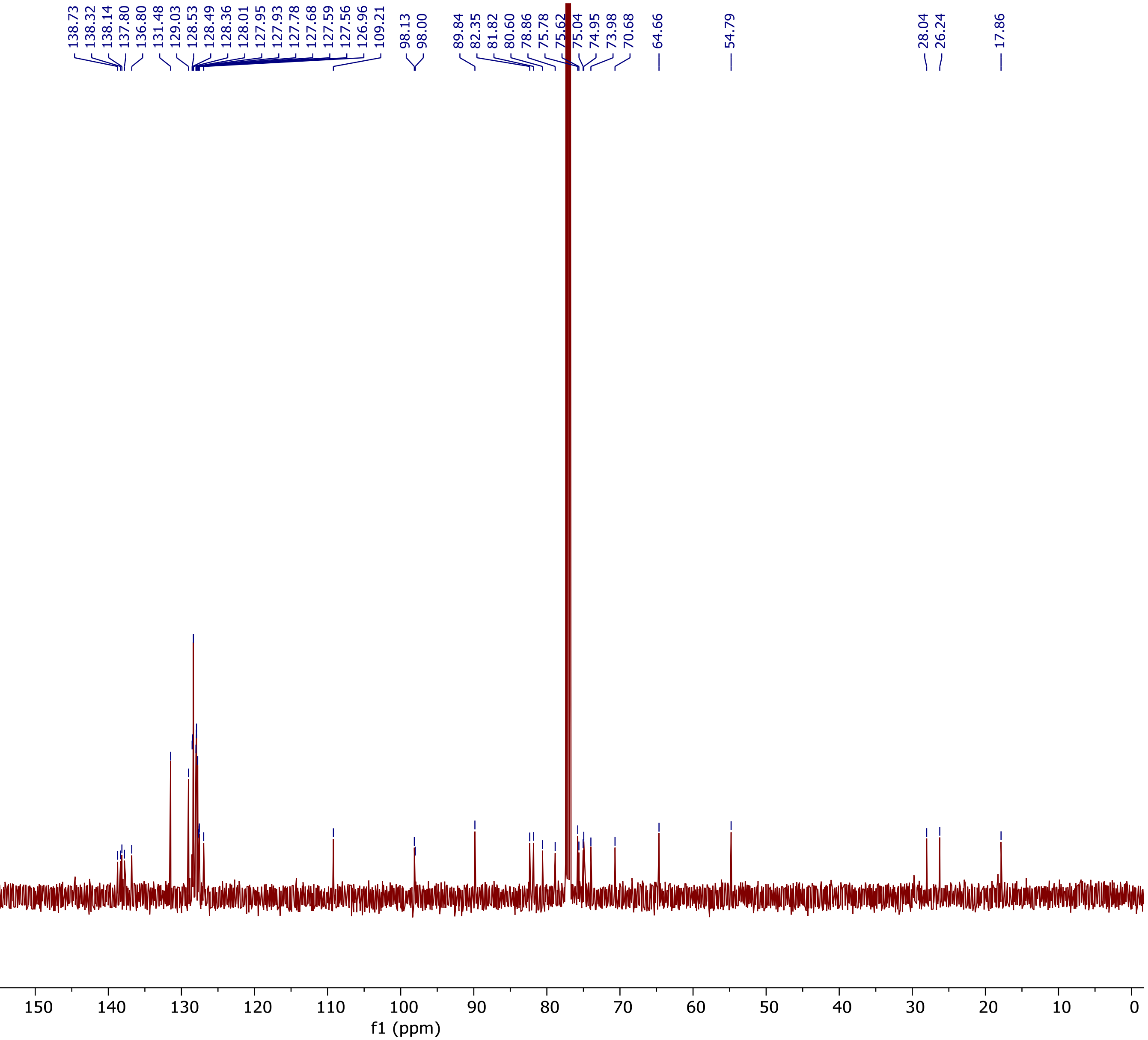

^{1}H NMR (500 MHz, CDCl$_3$) Spectrum of **28α** and **28β** enricched in **28β** Methyl (6*R*)-6-phenylthio-2,3,4,6-tetra-*O*-benzyl-*β*-D-glucopyranosyl-(1→4)-2,3-*O*-isopropylidene-α-L-rhamnopyranoside.

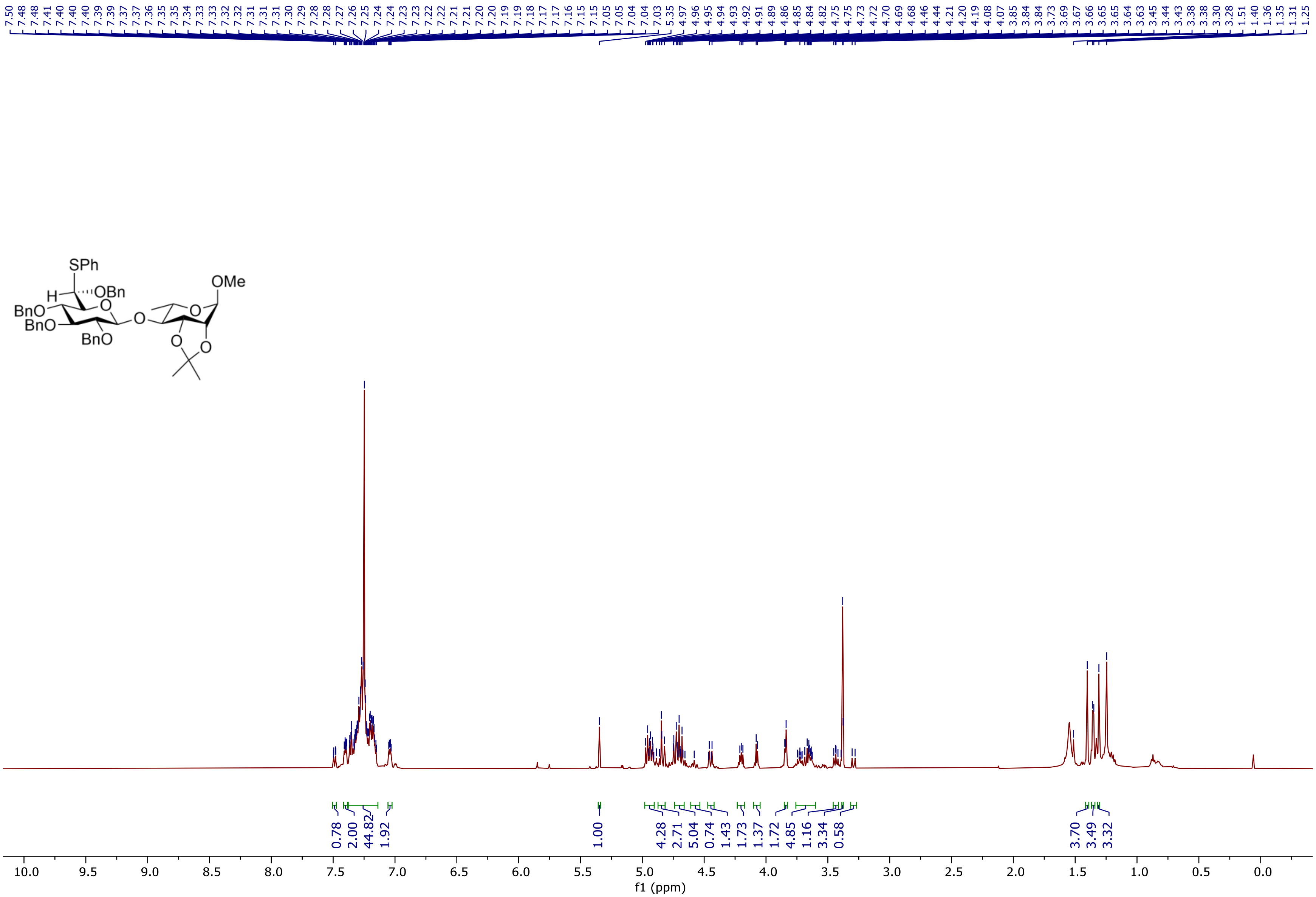

^{1}H NMR (500 MHz, CD_3OD) Spectrum of Methyl *β*-D-galactopyranosyl-(1→6)-1,2:3,4-*O*-diisopropylidene-*α*-D-galactopyranose **(29)**

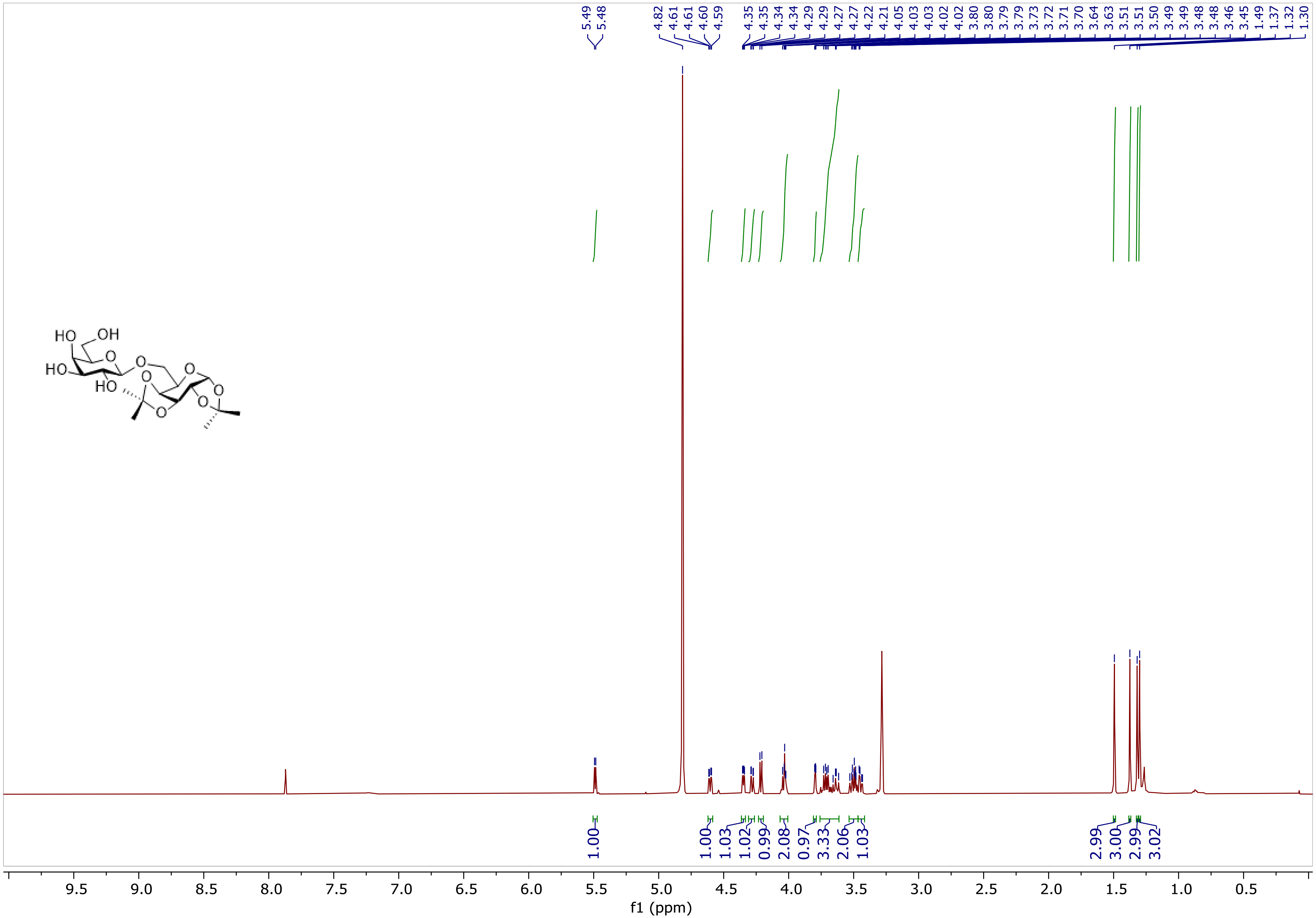

^{13}C {^{1}H} NMR (126 MHz, CD_3OD) Spectrum of Methyl *β*-D-galactopyranosyl-(1→6)-1,2:3,4-*O*-diisopropylidene-*α*-D-galactopyranose **(29)**

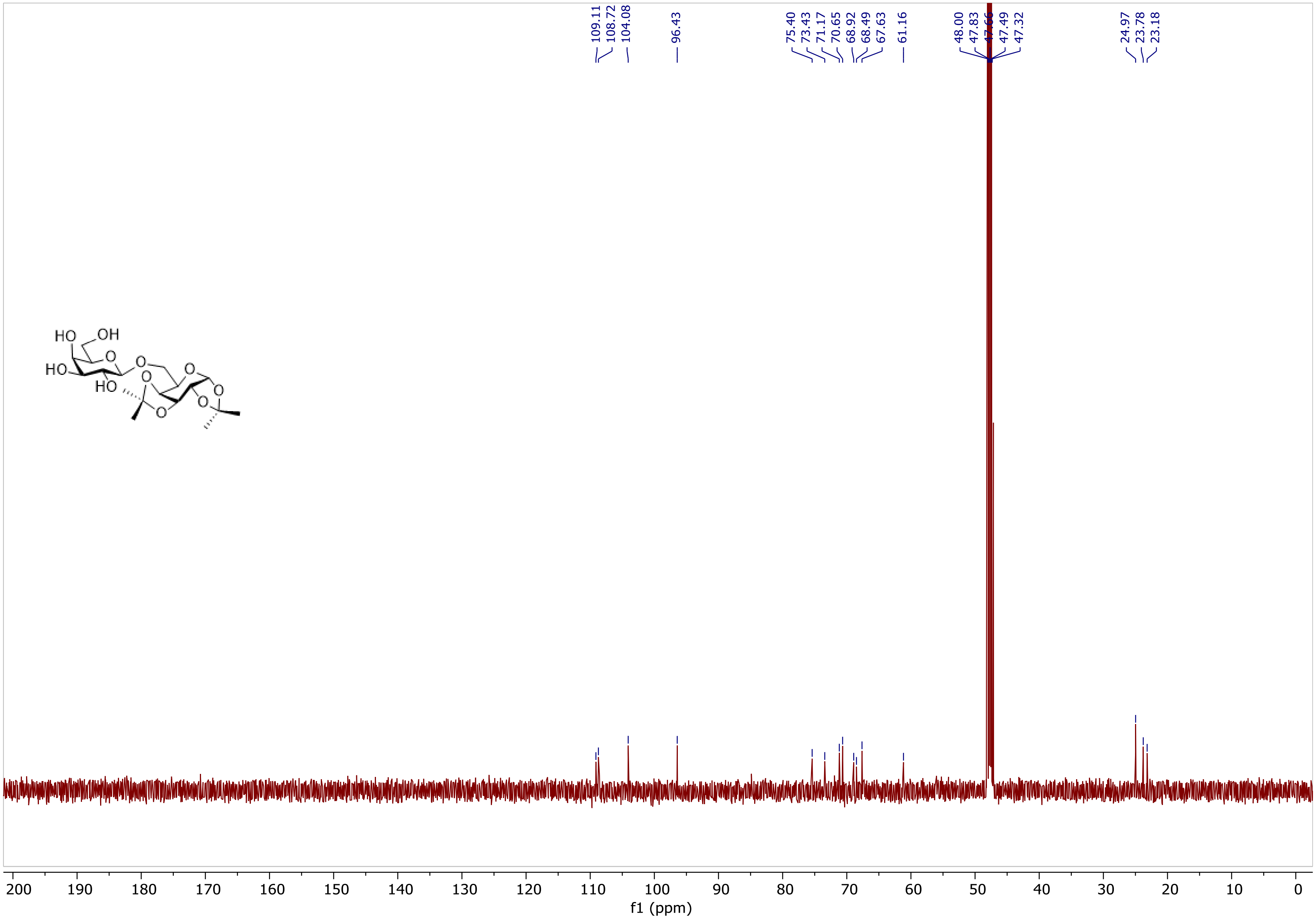

^{1}H NMR (500 MHz, CD_3OD) Spectrum of Adamantyl *β*-D-galactopyranoside **(30)**

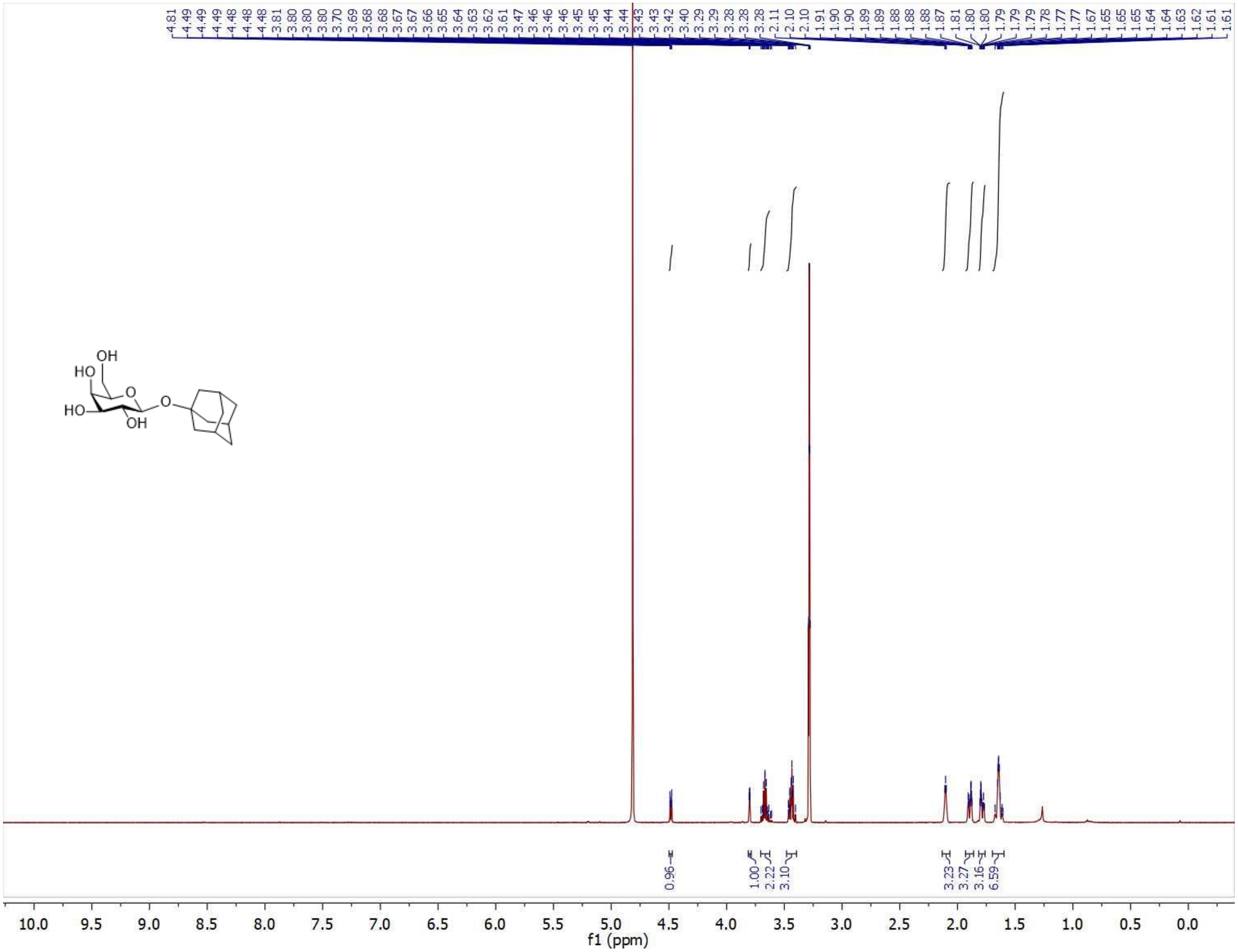

^{13}C {^{1}H} NMR (126 MHz, CD_3OD) Spectrum of Adamantyl *β*-D-galactopyranoside **(30)**

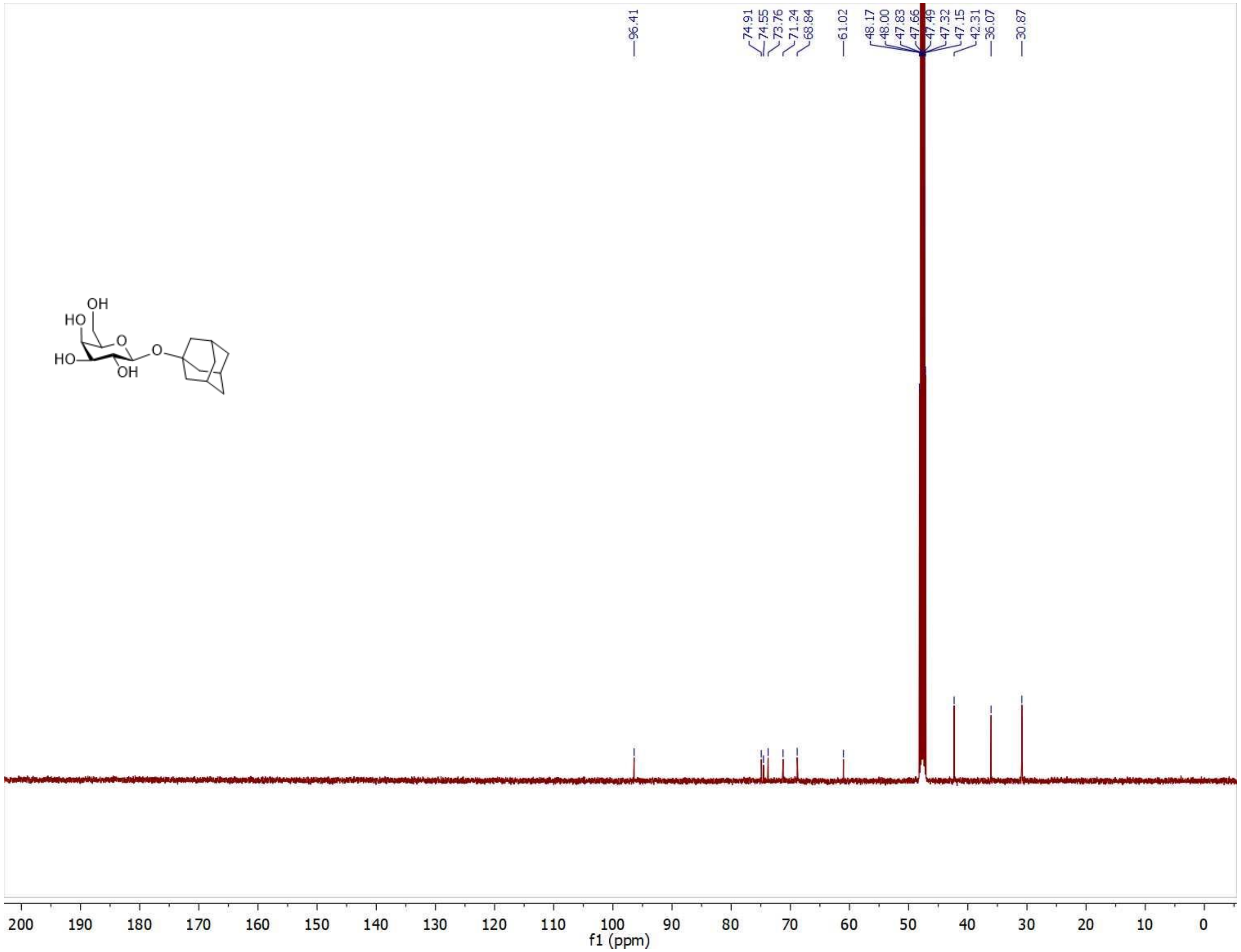

^{1}H NMR (500 MHz, CD_3OD) Spectrum of Methyl *β*-D-galactopyranosyl-(1→4)-2,3-*O*-isopropylidene-*α*-L-rhamnopyranoside **(31)**

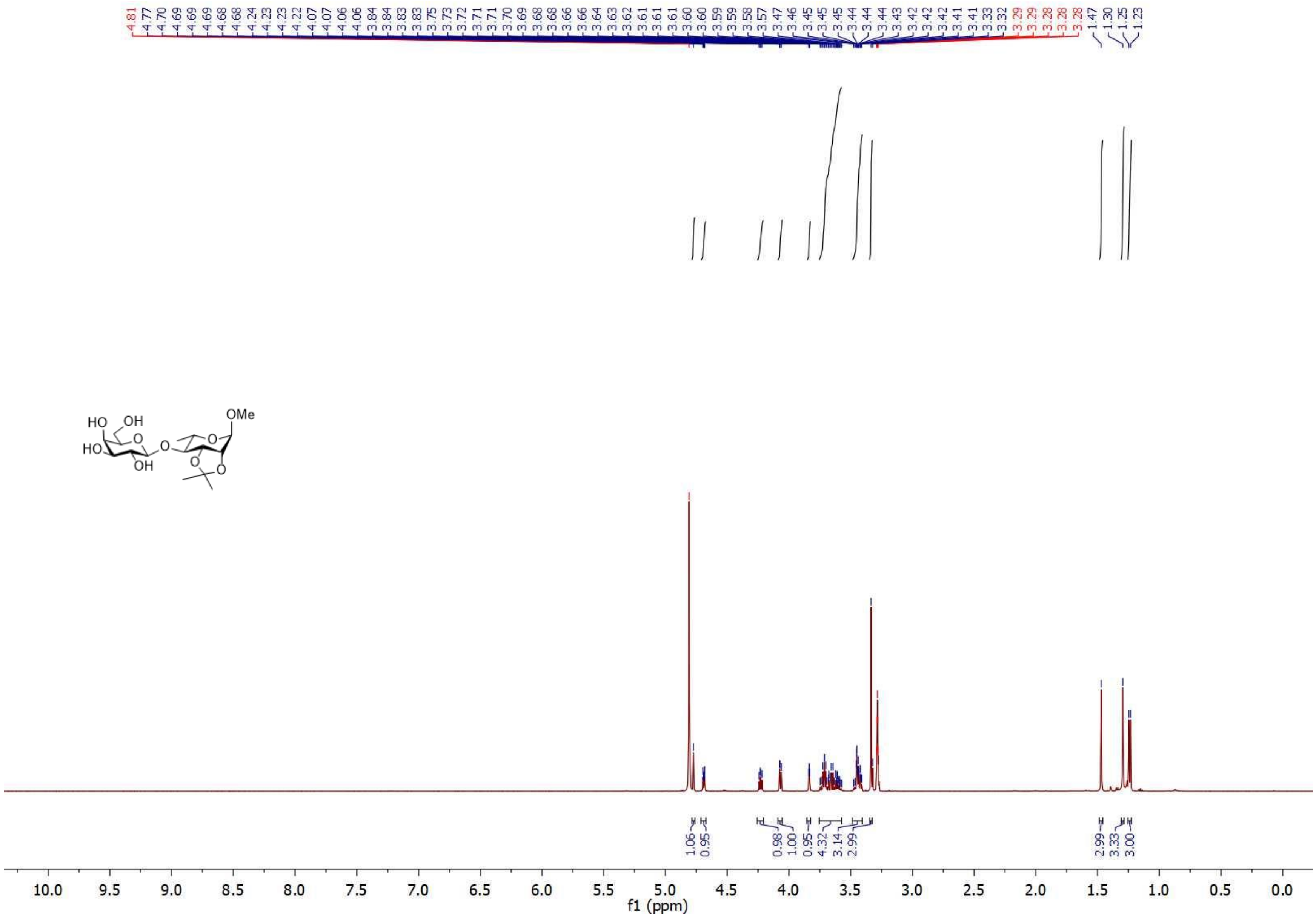

^{13}**C {^{1}H} NMR** (126 MHz, CD_3OD) Spectrum of Methyl *β*-D-galactopyranosyl-(1→4)-2,3-*O*-isopropylidene-*α*-L-rhamnopyranoside **(31)**

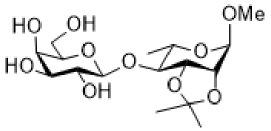

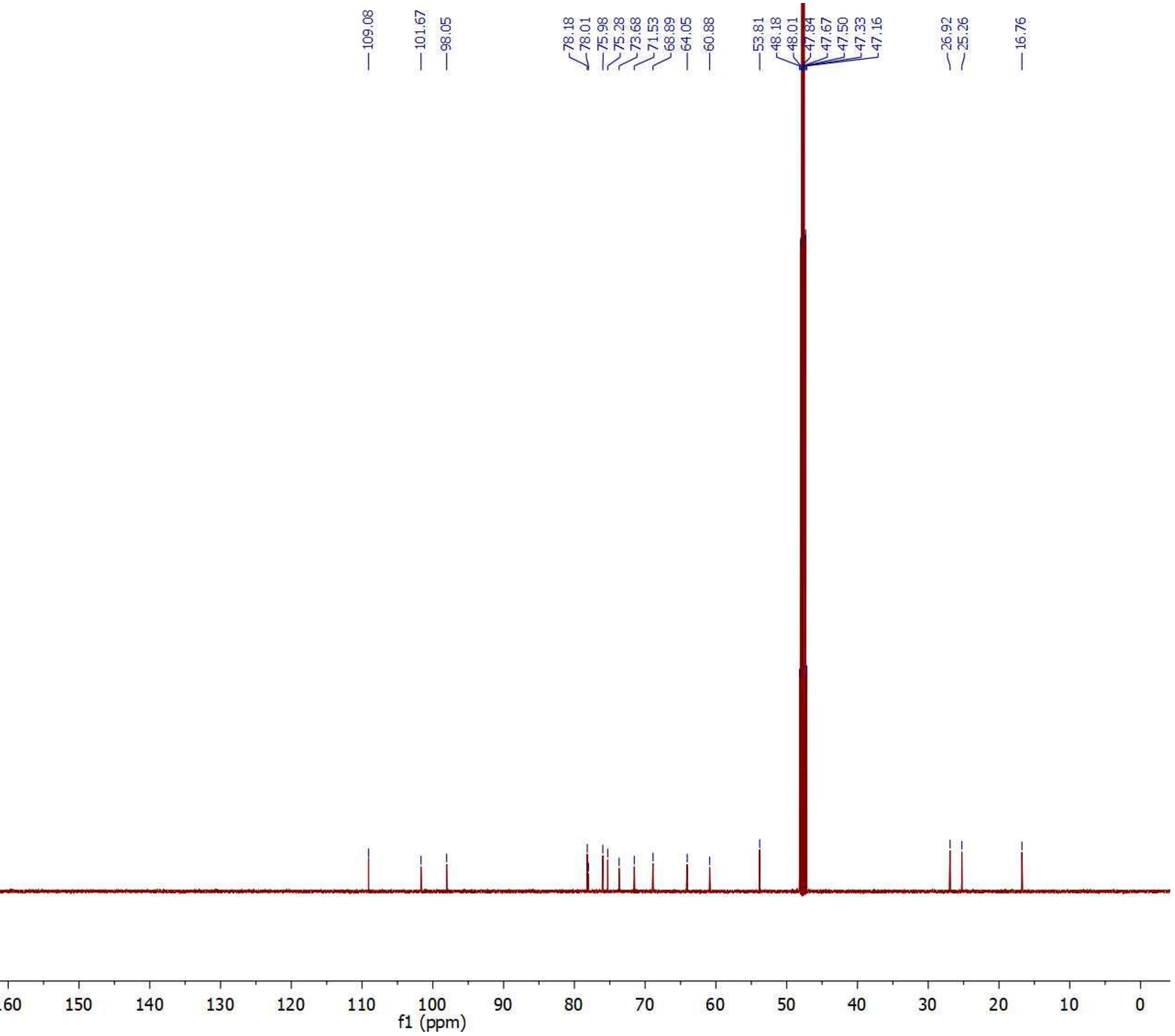

¹H NMR (500 MHz, CD_3OD) Spectrum of *β*-D-galactopyranosyl-(1→3)-1,2:5,6-di-*O*-isopropylidene-*α*-D-glucofuranose **(32)**

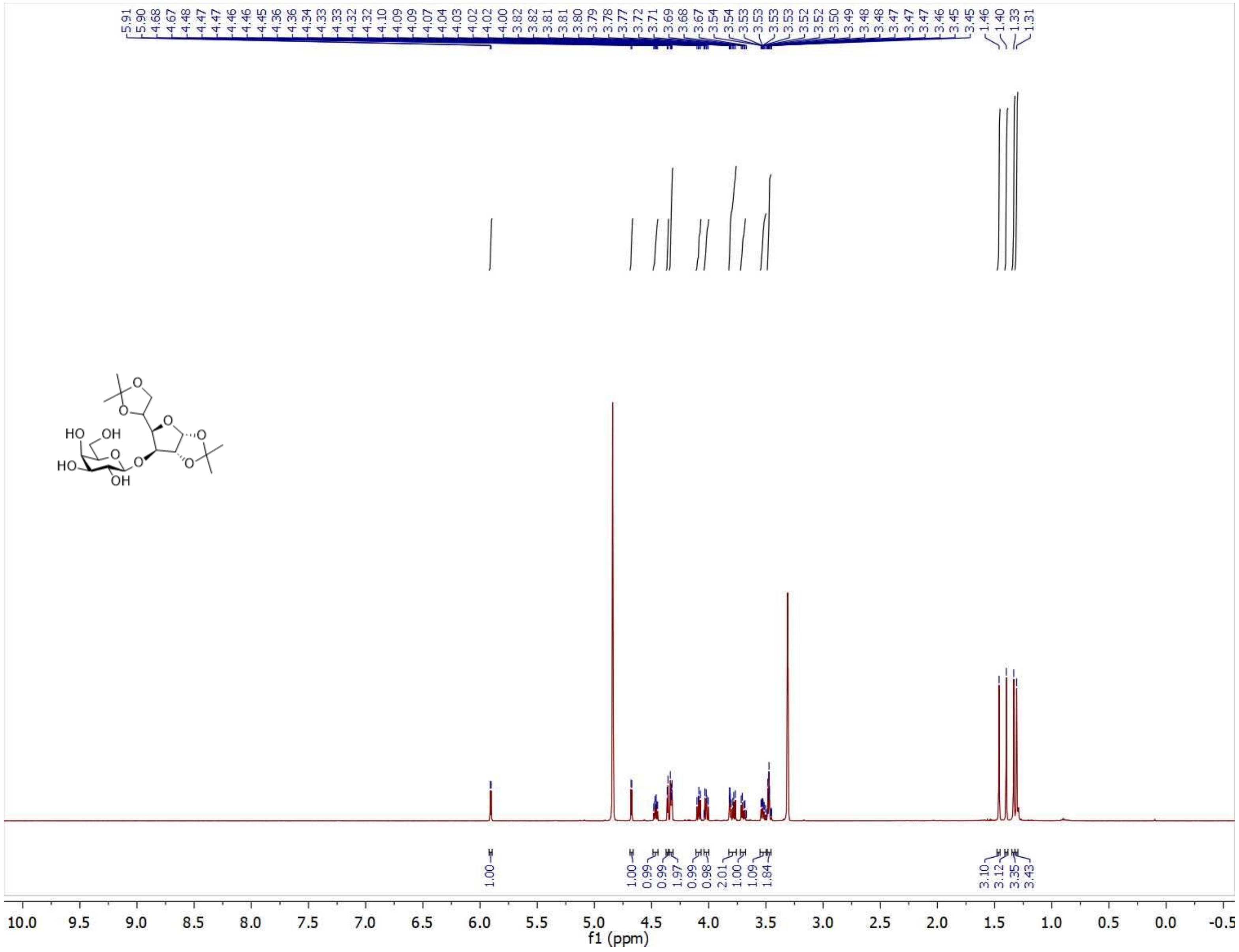

^{13}C {^{1}H} NMR (126 MHz, CD_3OD) Spectrum of β-D-galactopyranosyl-(1→3)-1,2:5,6-di-*O*-isopropylidene-α-D-glucofuranose **(32)**

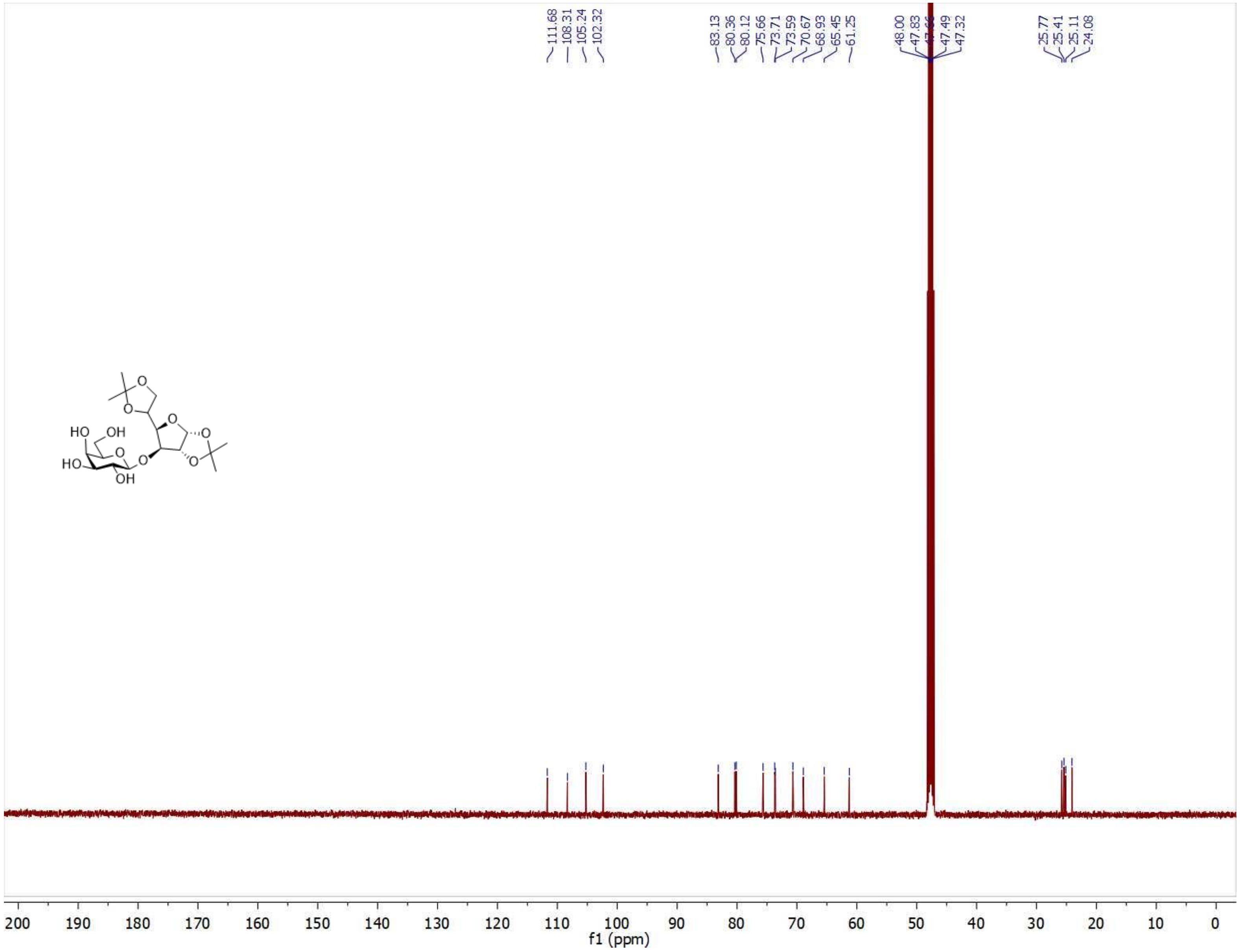

^{1}H NMR (600 MHz, D_2O) Spectrum of Methyl *α*-D-galactopyranosyl-(l→4)-*α*-D-glucopyranoside **(33)**

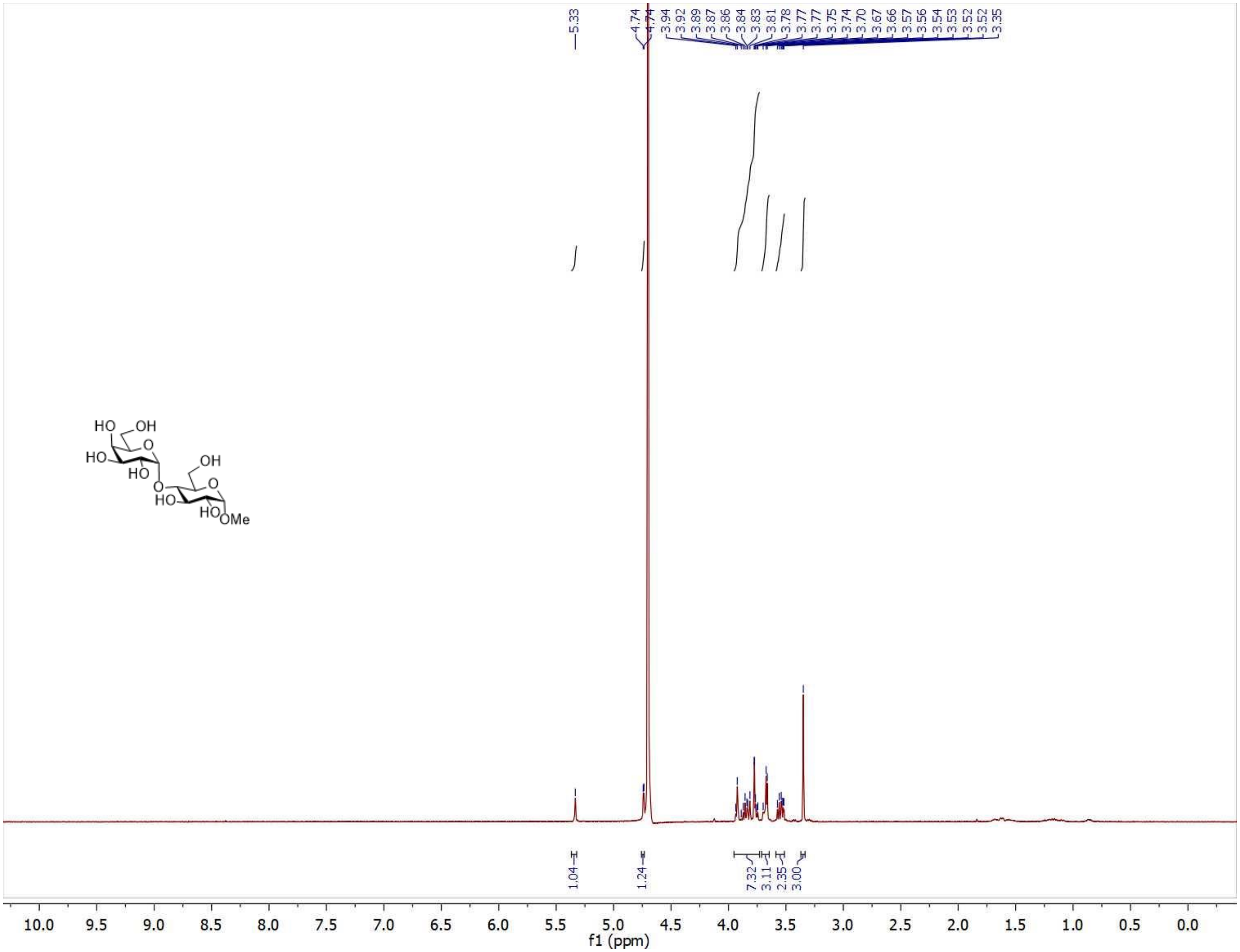

^{13}C {^{1}H} NMR (151 MHz, D_2O) Spectrum of Methyl *α*-D-galactopyranosyl-(l→4)-*α*-D-glucopyranoside **(33)**

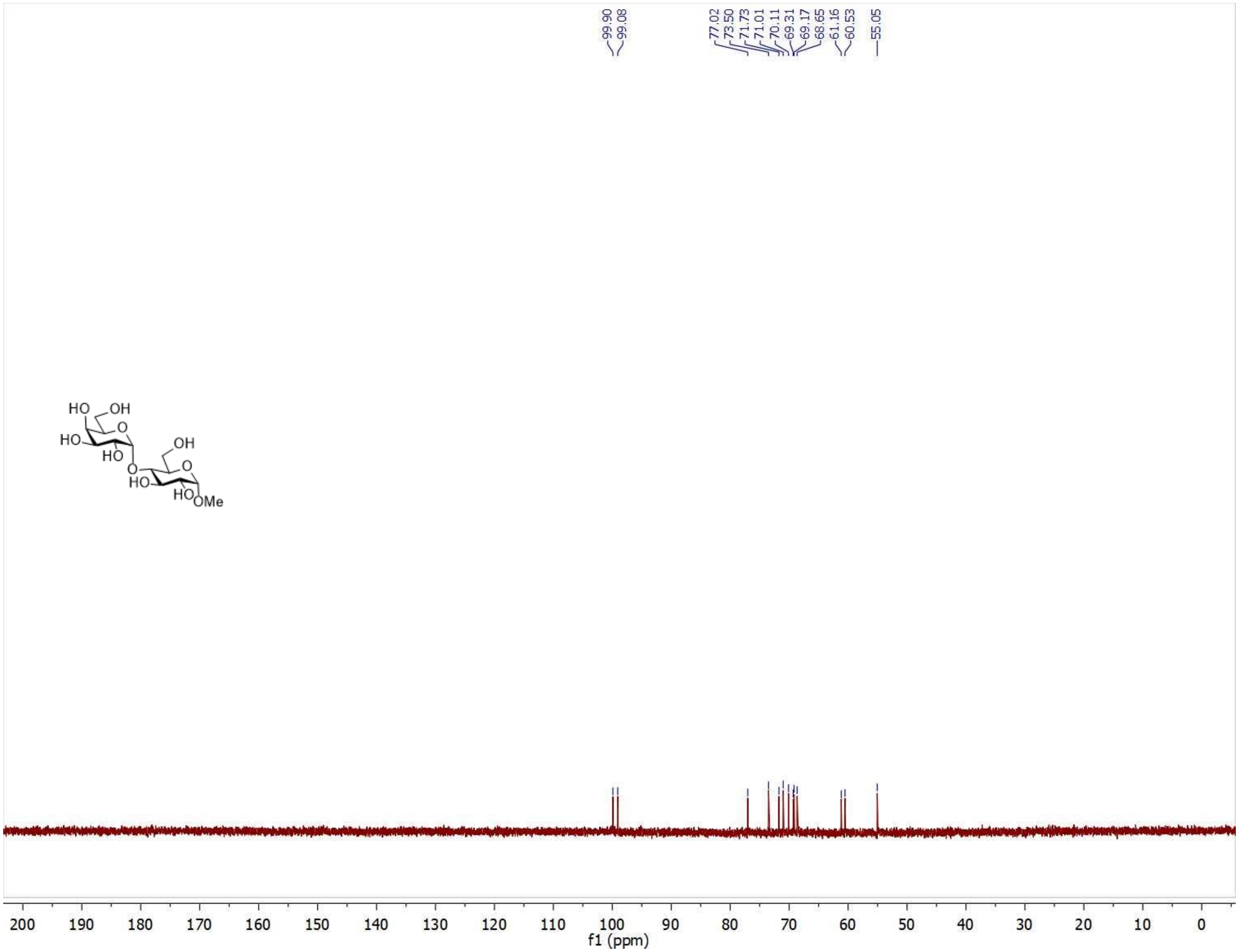

^{1}H NMR (500 MHz, D_2O) Spectrum of Methyl *β*-D-galactopyranosyl-(l→4)-*α*-D-glucopyranoside **(34)**

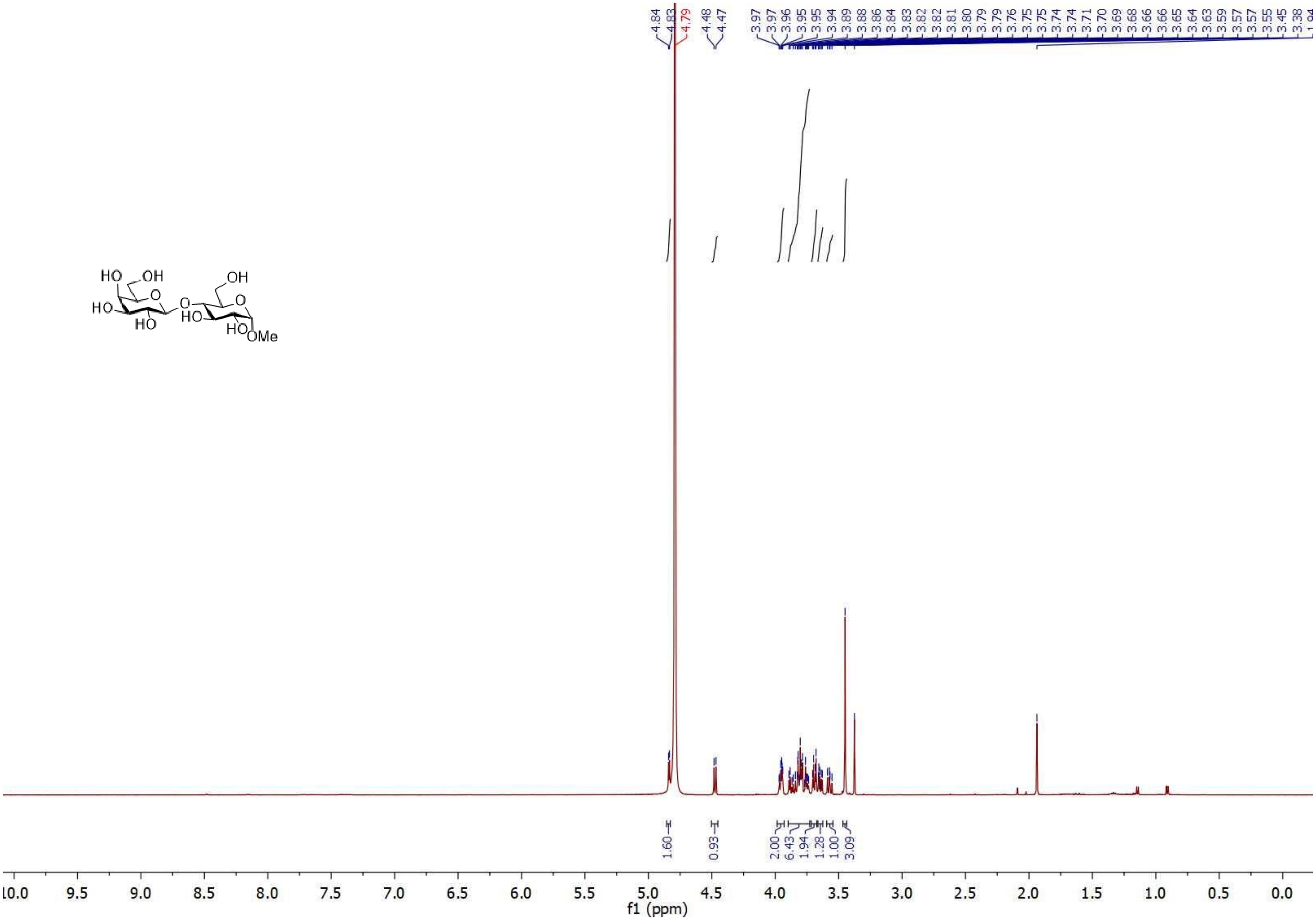

^{13}C {^{1}H} NMR (126 MHz, D_2O) Spectrum of Methyl *β*-D-galactopyranosyl-(l→4)-*α*-D-glucopyranoside **(34)**

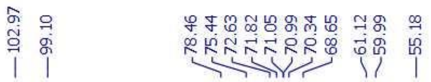

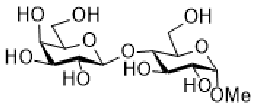

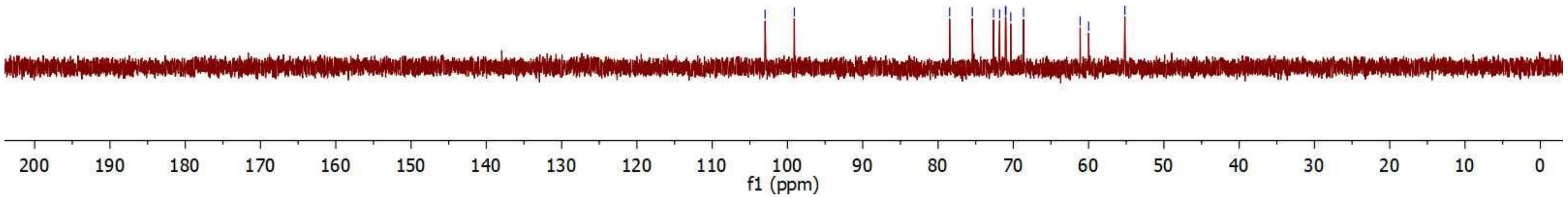

^{1}H NMR (500 MHz, CD_3OD) Spectrum of Methyl 4-*O*-(α-D-galactopyranosyl)-2,3-*O*-isopropylidene-α-L-rhamnopyranoside **(35)**

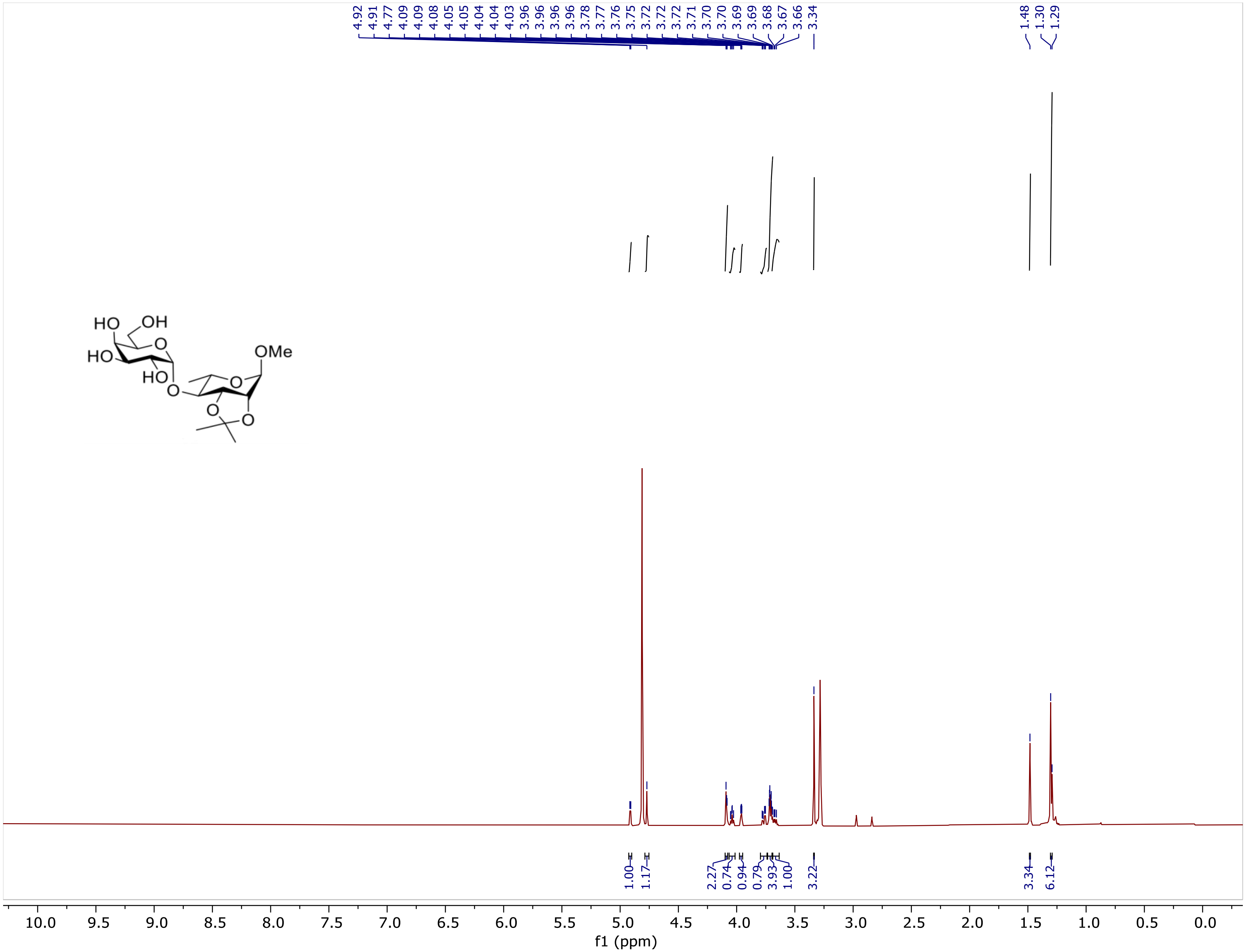

^{13}C {^{1}H} NMR (126 MHz, CD_3OD) Spectrum of Methyl 4-*O*-(*α*-D-galactopyranosyl)-2,3-*O*-isopropylidene-*α*-L-rhamnopyranoside **(35)**

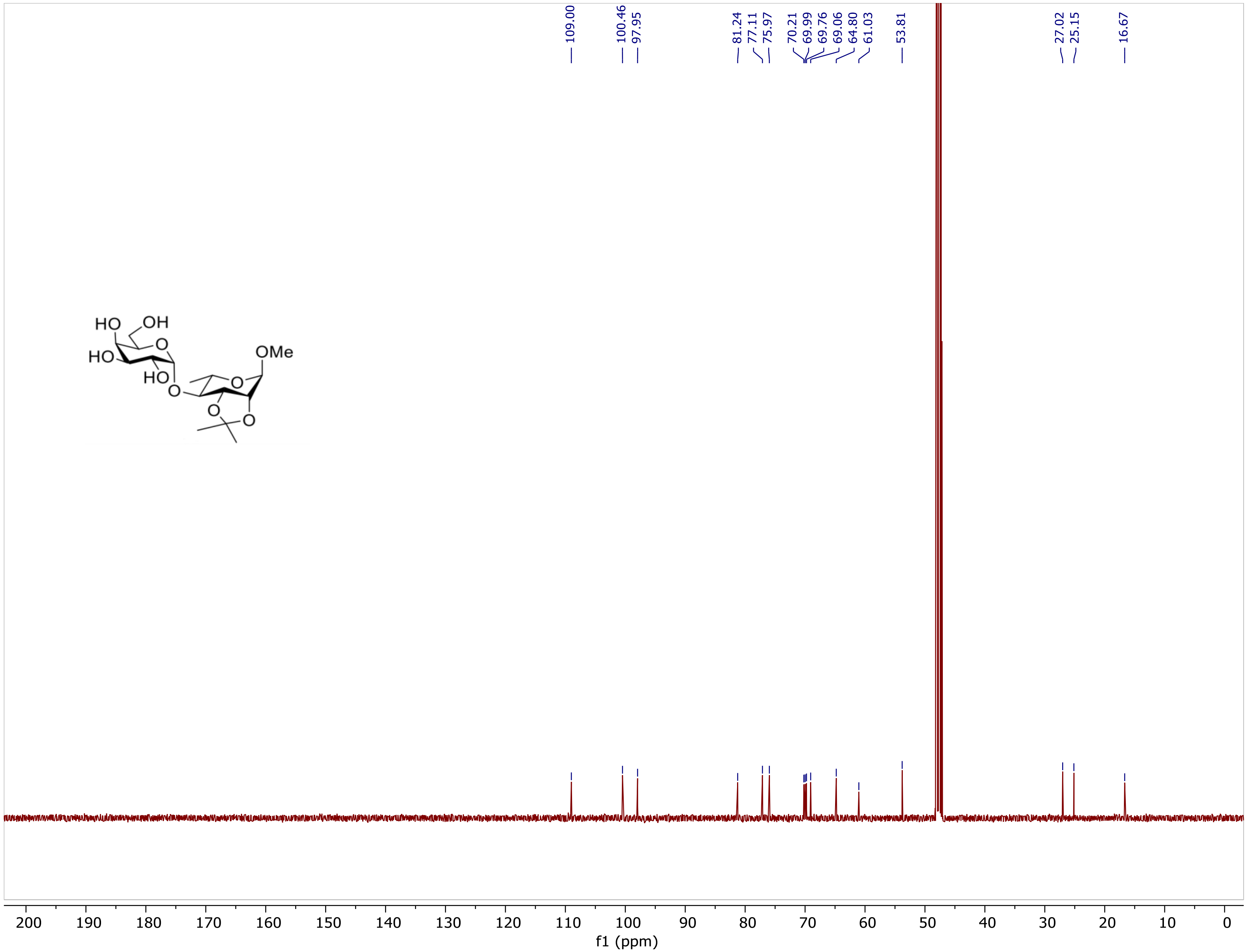

¹H NMR (500 MHz, CD₃CN) Spectrum of 6-*O*-β-D-Glucopyranosyl-1,2:3,4-*O*-diisopropylidene-α-D-galactopyranose. **(36)**

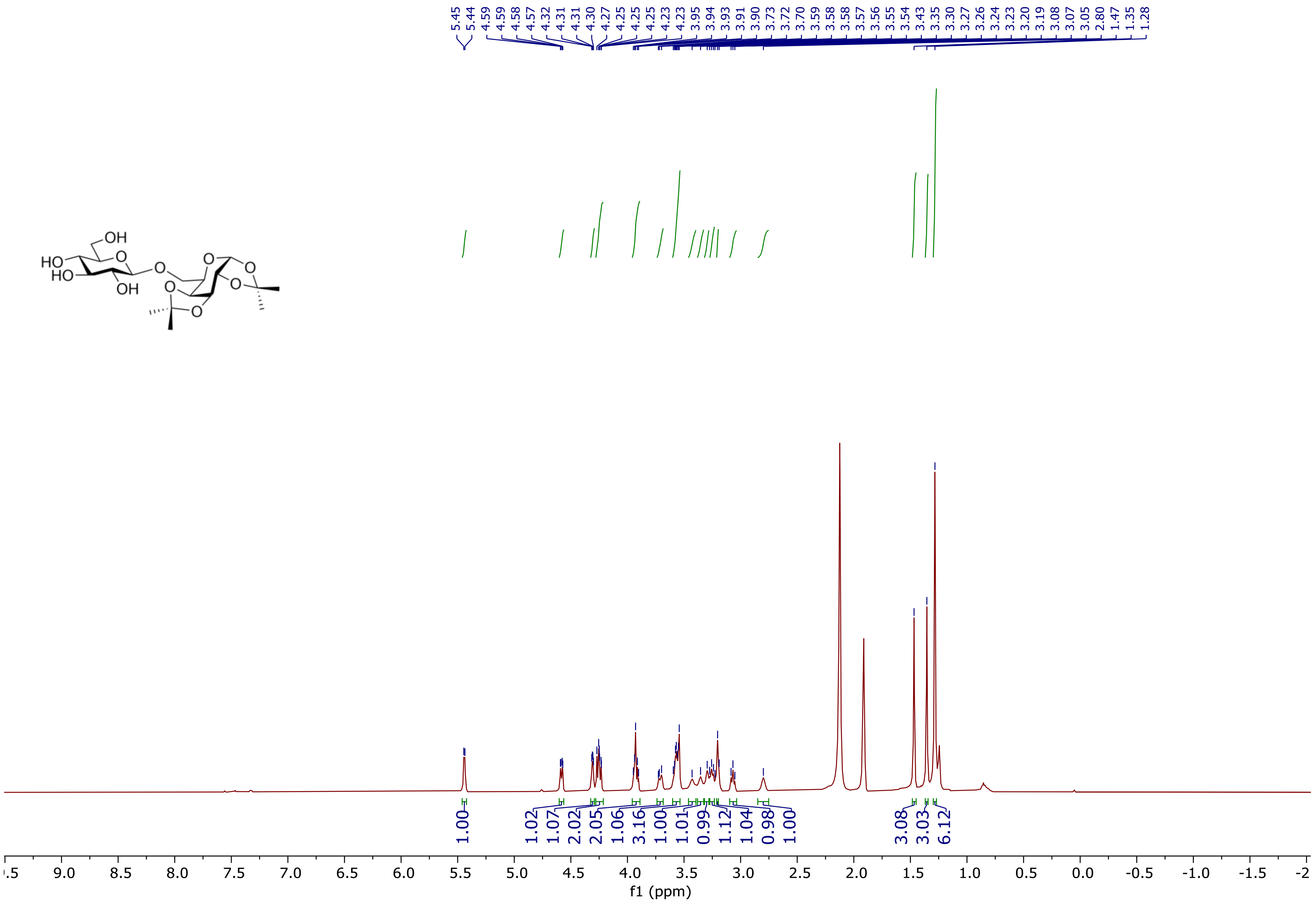

^{13}C{^{1}H} NMR (126 MHz, CD_3CN) Spectrum of 6-*O*-β-D-*gluco*pyranosyl-1,2:3,4-*O*-diisopropylidene-α-D-galactopyranose. **(36)**

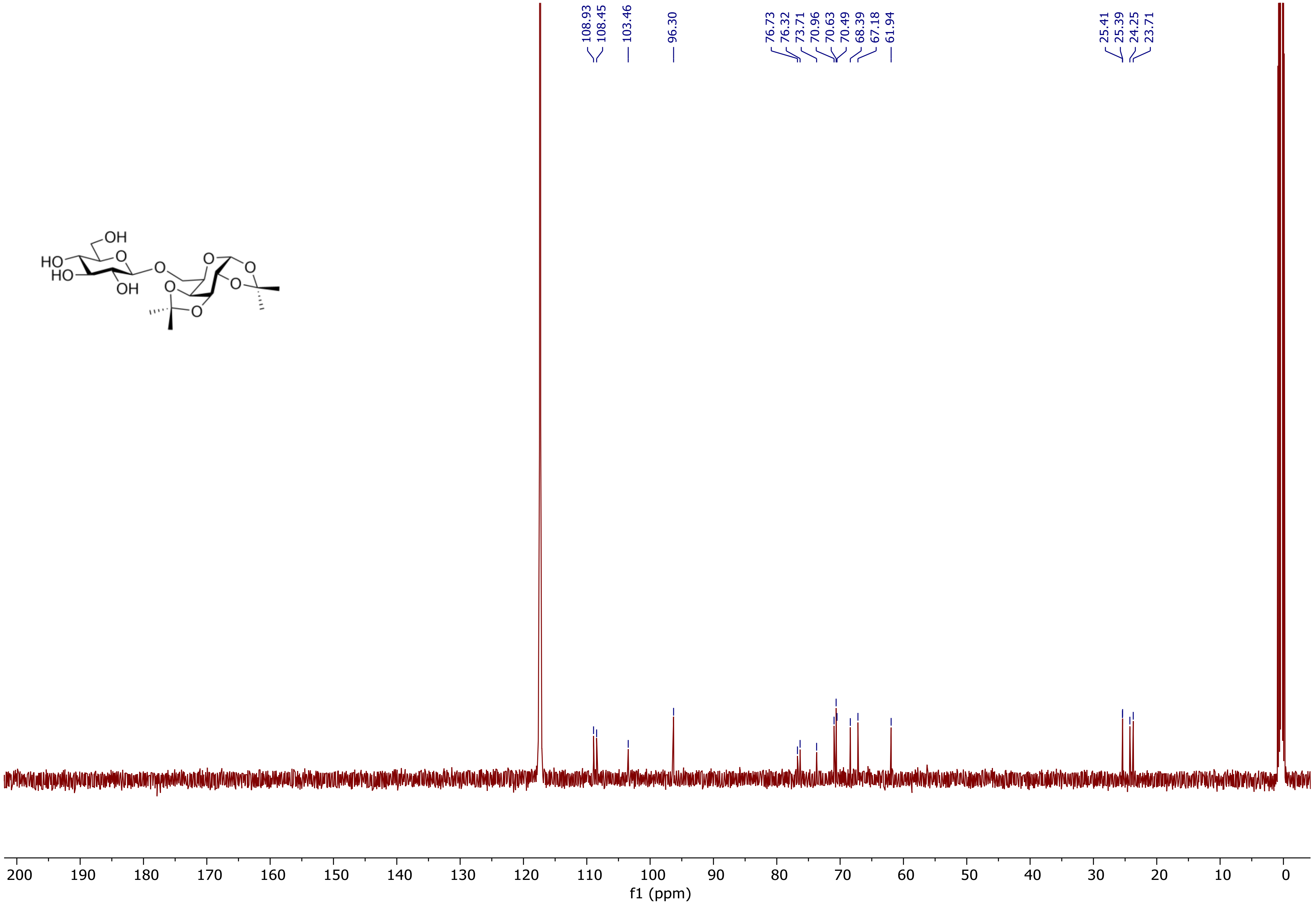

^{1}H NMR (500 MHz, CD_3OD) Spectrum of Adamantyl β-D-glucopyranose. **(37)**

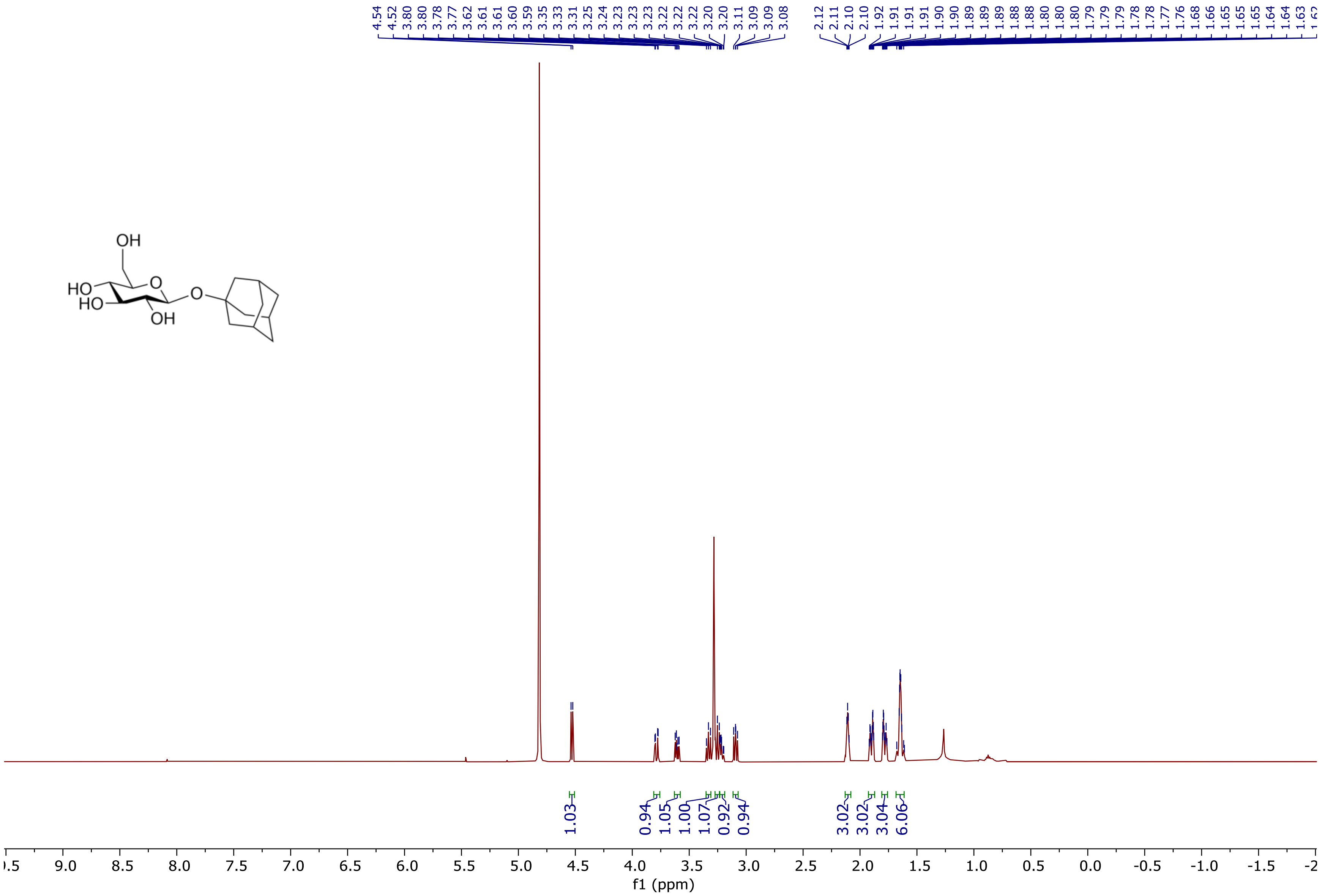

$^{13}C\{^1H\}$ **NMR** (126 MHz, CD_3OD) Spectrum of Adamantyl *β*-D-glucopyranose. **(37)**

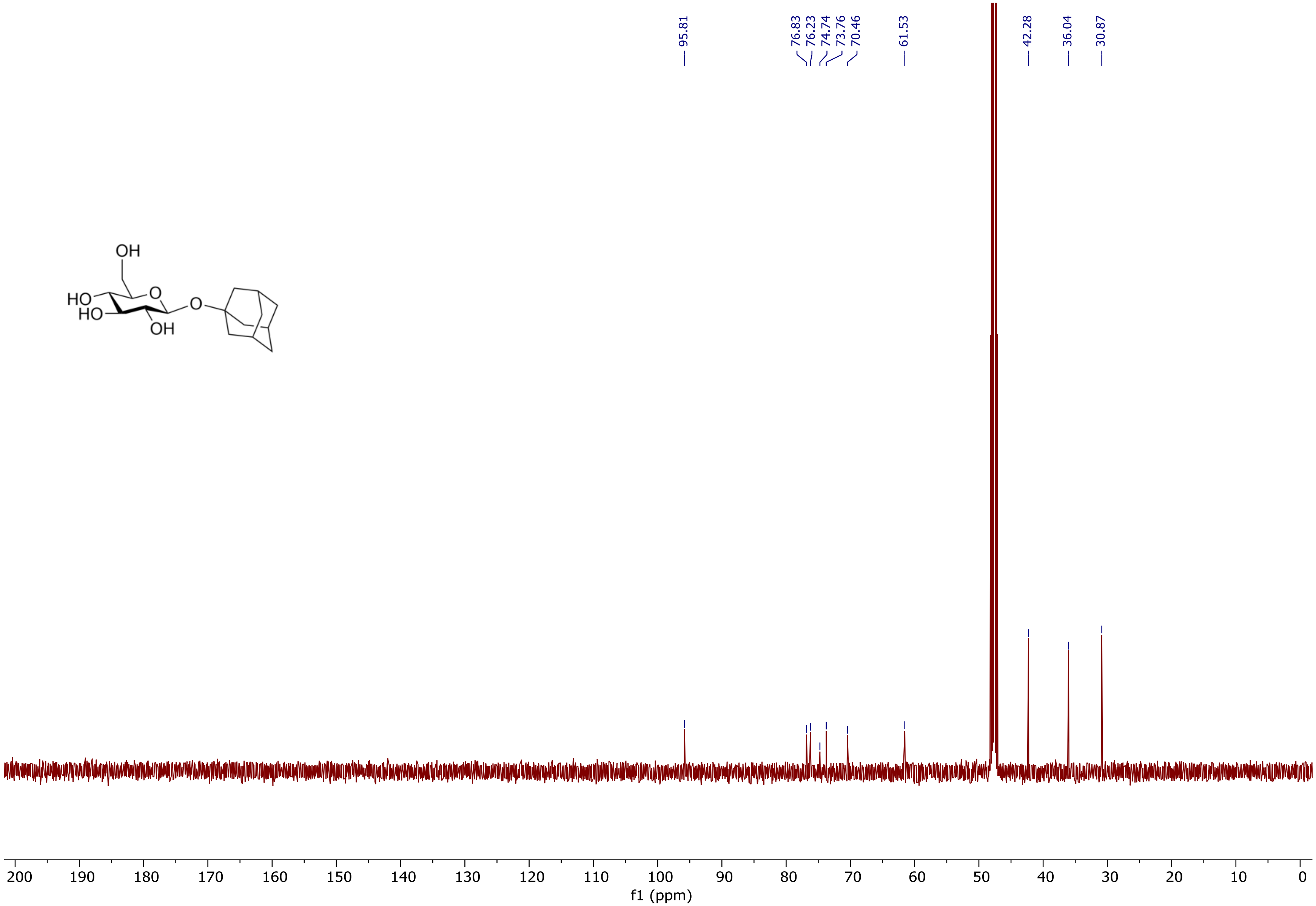

^{1}H NMR (500 MHz, CD$_3$OD) Spectrum of Methyl 4-*O*-*α*-D-glucopyranosyl-2,3-*O*-isopropylidene-α-L-rhamnopyranose. **(38)**

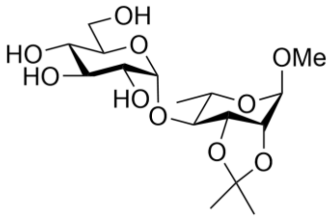

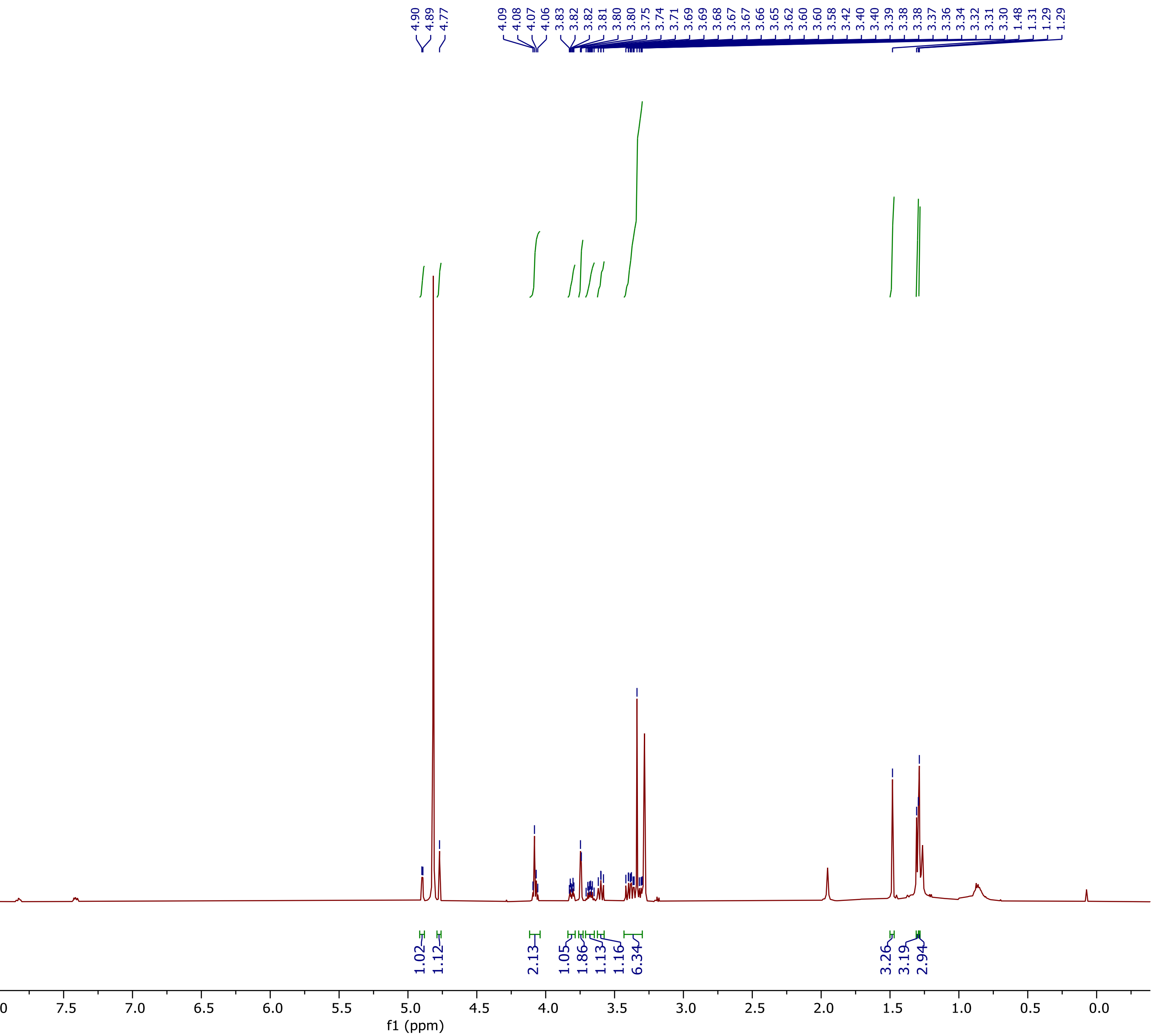

^{13}C{^{1}H} NMR (126 MHz, CD_3OD) Spectrum of Methyl 4-*O*-*α*-D-glucopyranosyl-2,3-*O*-isopropylidene-α-L-rhamnopyranose. **(38)**

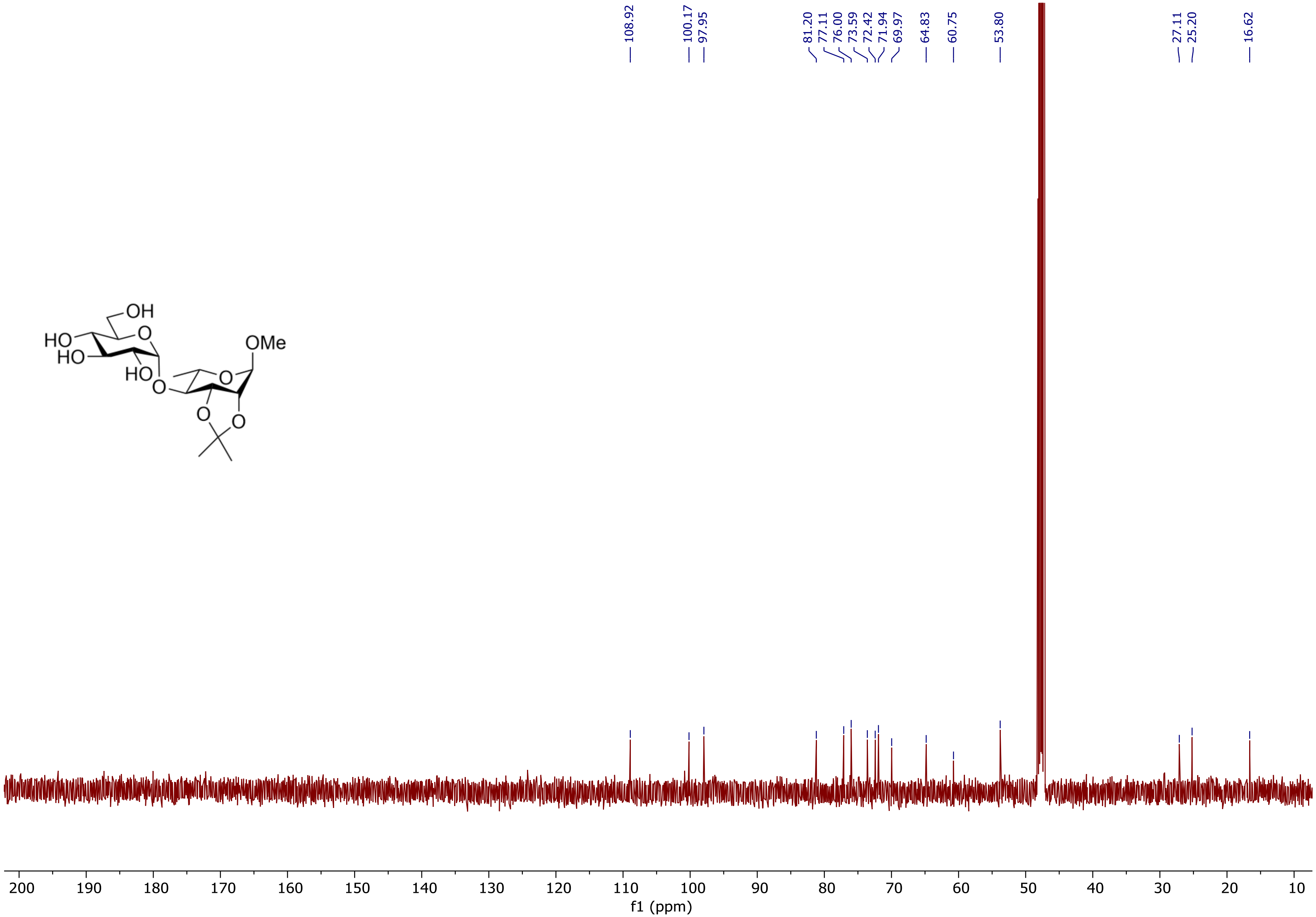

^{1}H NMR (500 MHz, CD_3OD) Spectrum of Adamantyl α-D-glucopyranose. **(39)**

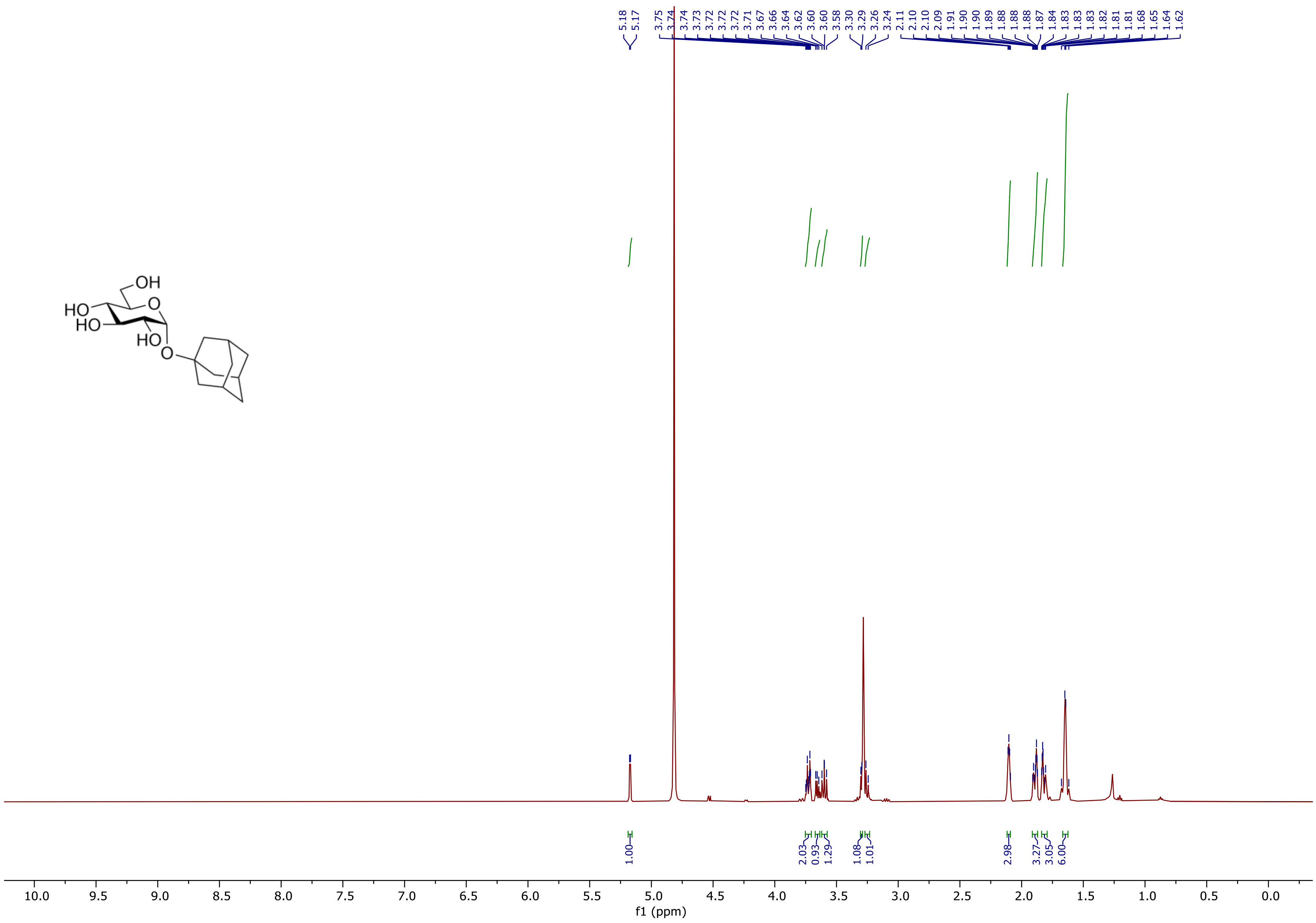

^{13}C{^{1}H} NMR (126 MHz, CD_3OD) Spectrum of Adamantyl α-D-glucopyranose. **(39)**

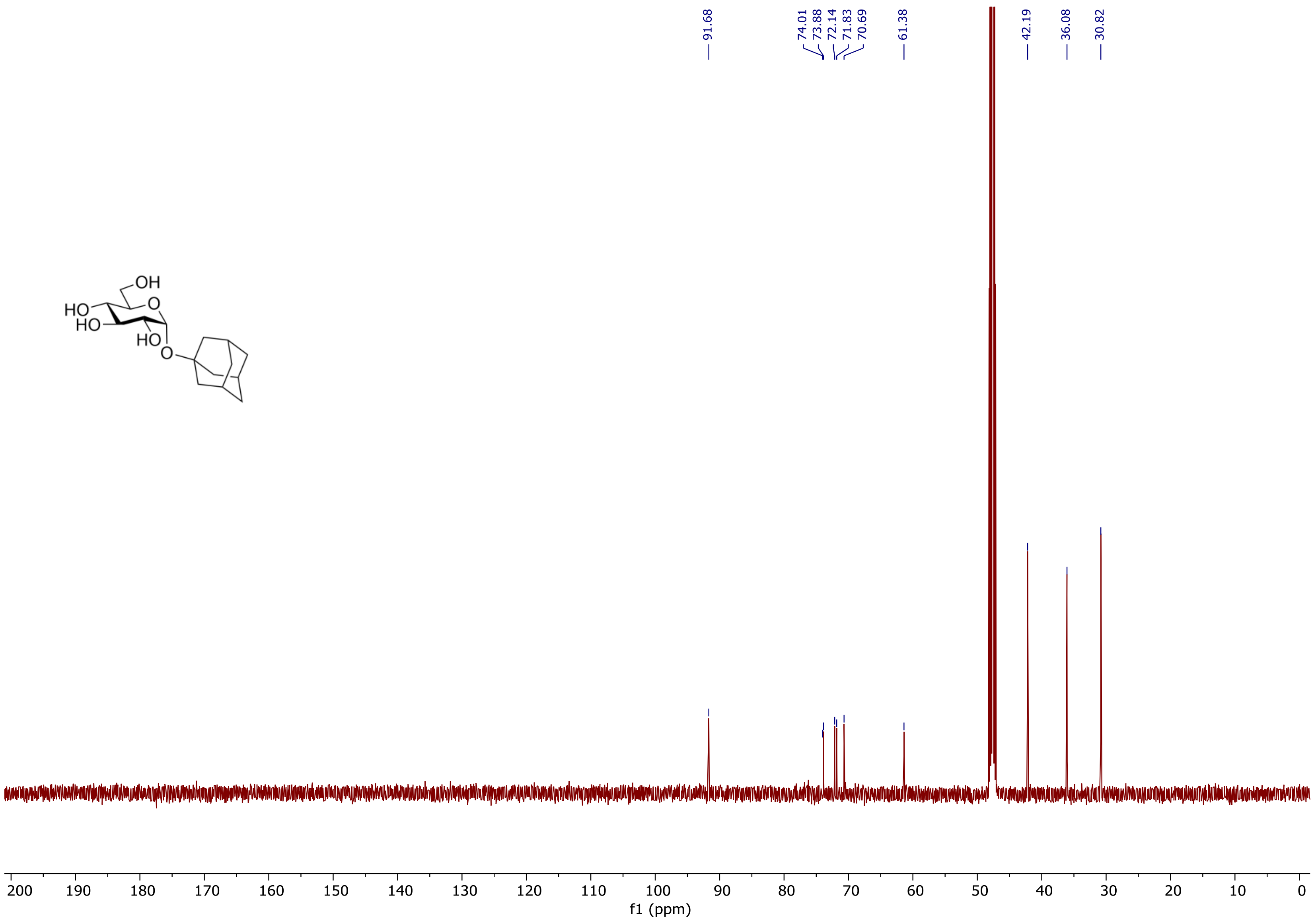

Supplement: Supplementary file 1 — jo2c02889_si_001.pdf [file jo2c02889_si_001.pdf]
